# Competition between N and O: use of diazine *N*-oxides as a test case for the Marcus theory rationale for ambident reactivity†

**DOI:** 10.1039/d0sc02834g

**Published:** 2020-07-23

**Authors:** Kevin J. Sheehy, Lorraine M. Bateman, Niko T. Flosbach, Martin Breugst, Peter A. Byrne

**Affiliations:** School of Chemistry, Analytical and Biological Chemistry Research Facility, University College Cork College Road Cork Ireland peter.byrne@ucc.ie; School of Pharmacy, University College Cork College Road Ireland; Department für Chemie, Universität zu Köln Greinstraße 4 50939 Köln Germany mbreugst@uni-koeln.de; SSPC (Synthesis and Solid State Pharmaceutical Centre) Cork Ireland

## Abstract

The preferred site of alkylation of diazine *N*-oxides by representative hard and soft alkylating agents was established conclusively using the ^1^H–^15^N HMBC NMR technique in combination with other NMR spectroscopic methods. Alkylation of pyrazine *N*-oxides (**1** and **2**) occurs preferentially on nitrogen regardless of the alkylating agent employed, while *O*-methylation of pyrimidine *N*-oxide (**3**) is favoured in its reaction with MeOTf. As these outcomes cannot be explained in the context of the hard/soft acid/base (HSAB) principle, we have instead turned to Marcus theory to rationalise these results. Marcus intrinsic barriers (Δ*G*^‡^_0_) and Δ_r_*G*° values were calculated at the DLPNO-CCSD(T)/def2-TZVPPD/SMD//M06-2X-D3/6-311+G(d,p)/SMD level of theory for methylation reactions of **1** and **3** by MeI and MeOTf, and used to derive Gibbs energies of activation (Δ*G*^‡^) for the processes of *N*- and *O*-methylation, respectively. These values, as well as those derived directly from the DFT calculations, closely reproduce the observed experimental *N*- vs. *O*-alkylation selectivities for methylation reactions of **1** and **3**, indicating that Marcus theory can be used in a semi-quantitative manner to understand how the activation barriers for these reactions are constructed. It was found that *N*-alkylation of **1** is favoured due to the dominant contribution of Δ_r_*G*° to the activation barrier in this case, while *O*-alkylation of **3** is favoured due to the dominant contribution of the intrinsic barrier (Δ*G*^‡^_0_) for this process. These results are of profound significance in understanding the outcomes of reactions of ambident reactants in general.

## Abstract 2

Marcus theory enables rationalisation and quantification of selectivities in reactions of ambident nucleophiles for which the HSAB principle cannot operate.

## Graphical abstract

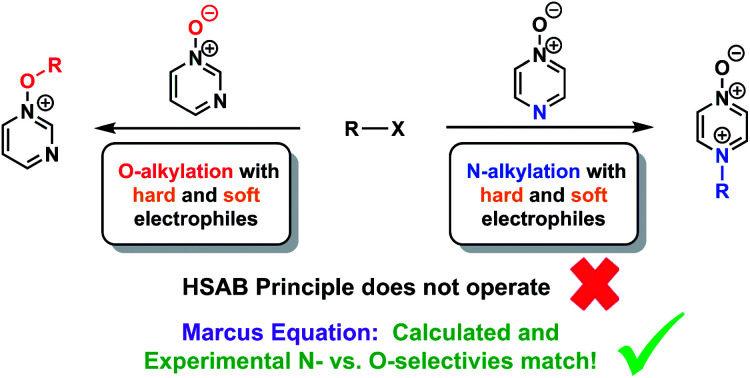

## Introduction

### Selectivity in reactions of ambident nucleophiles

A fundamental goal in organic chemistry is to be able to understand and rationalise why chemical processes occur as they do. Naturally, therefore, an understanding of the factors that govern regioselectivity in chemical reactions is of paramount importance – *i.e.* if a compound contains more than one reactive site, which one is preferred, and why? Reliably accounting for the regioselectivity observed in reactions of ambident nucleophiles and electrophiles is a challenge laden with difficulties and potential pitfalls. By far the most popular rationale for this purpose^1^ makes use of the principle of hard and soft acids and bases (the HSAB principle),^2^ and the related concept of charge *vs.* orbital control.^3^ The difficulty inherent in accounting for the selectivities observed in reactions of ambident nucleophiles is exemplified by the fact that the HSAB principle *predicts the incorrect product* in a very large number of cases, as has been reviewed in detail by Mayr and co-workers.^4^ The data in this review call starkly into question whether the principle adequately explains the observed selectivity in reactions of ambident nucleophiles in which the expected outcome (based on HSAB theory) does match the experimental outcome.^5^

Mayr and co-workers have suggested employing Marcus theory (described below) as an alternative method of accounting qualitatively for the selectivities of reactions of ambident reactants.^4^

Recently, Wang, Barnes and co-workers conducted computational investigations to establish a theoretical basis for applying the HSAB principle in rationalising ambident reactivity, and used this, along with Marcus theory, to explain the results of their calculations on gas phase reactions of amide anions.^6^ However, so far, the Marcus theory-based approach has not been adopted by the wider research community, and in fact the HSAB rationale continues to be cited in cases in which the experimental results do align, perhaps arbitrarily, with expectations based on this principle.^5^ Furthermore, the elements of the intuitively alluring HSAB rationale pervade all discussions of ambident reactivity in undergraduate chemistry courses, and in the most comprehensive organic chemistry textbooks.^1^ Given the clear deficiencies of the HSAB rationale in the context of ambident reactivity, it now behoves organic chemists to test Mayr's approach and other alternatives on their capacity to account for the outcomes of reactions of ambident reactants.

**Scheme 1:**
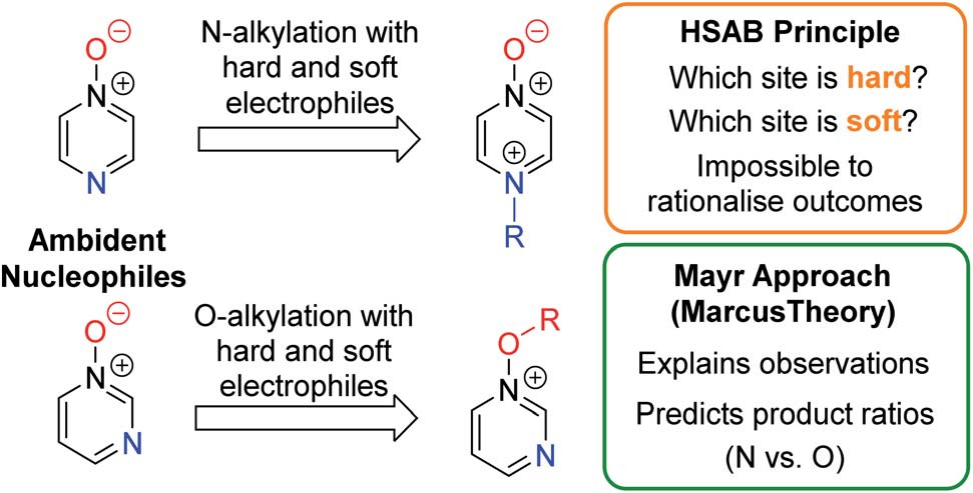
Approaches for rationalising selectivity in reactions of diazine *N*-oxides as representative ambident nucleophiles.

**Fig. 1:**
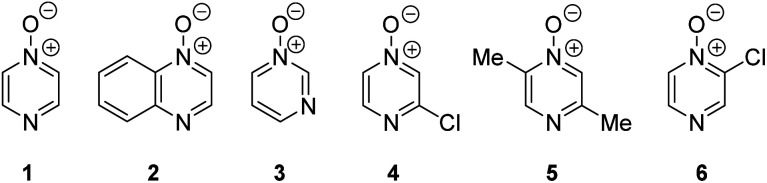
Representative diazine *N*-oxides.

Herein, we focus on the notoriously difficult problem of competition between N and O nucleophilic sites (Scheme 1).^4,5*c*,6,7–14^ We chose diazine *N*-oxides **1**, **2** and **3** (Fig. 1) as test substrates in reactions with various representative hard and soft electrophiles because, although these reactions show very high site-selectivity (*i.e.* for *N*- or *O*-alkylation),^7^ their outcomes are intractable to rationalisation using the HSAB principle (Scheme 1), as will be discussed in the next section. An additional contributing factor that confounds any attempt to analyse the reactions of these species using the HSAB rationale is that it is not possible to unambiguously identify which nucleophilic site of a diazine *N*-oxide is the hard site, and which is the soft site (see later).^15^

In this work, we will show that the approach of Mayr and co-workers enables accurate prediction of the preferred site of alkylation of ambident nucleophiles **1–3**. Furthermore, we will also show that it is even possible to calculate the ratio of the selectivities for the different nucleophilic sites in these compounds (N *vs.* O) with an impressive degree of accuracy (Scheme 1).^16^ Our results bolster the applicability of the Marcus theory-based approach and establish, for the first time, its capacity to semi-quantitatively account for the ratios of site-selectivities in reactions of ambident nucleophiles.

It should be noted that the limitations of the HSAB principle were highlighted by its developer (Pearson),^2*d*,*f*^ and that in its original formulation,^2*a*,*b*^ it was not derived with the intention of rationalising the selectivities of reactions of ambident reactants. However, thereafter, it has been^2*c*^ and continues to be applied in this manner.^1,5^ In recent years, a theoretical grounding demonstrating the applicability of the “global” HSAB principle (which does not apply to ambident reactants) has been developed.^17,18^ Despite the authors' inclusion in the articles on this topic of precise statements such as “*The local HSAB principle, which makes predictions about ambident acids and bases, is on much shakier theoretical ground, so experimental evidence against it is not surprising*”,^15*a*,17*b*^ these papers are nonetheless cited in other articles in support of application of the HSAB principle to the analysis of reactions of ambident nucleophiles.^5*c*^ This is illustrative of the continued application of the HSAB principle to rationalisation of ambident reactivity in the wider chemistry community despite the large body of evidence demonstrating that it does not apply in such instances.

### Competition between N and O nucleophilic sites

Numerous examples of reactions of ambident nucleophiles containing competing O and N nucleophilic sites exist in the literature.^6,10–14,19–32^ Compounds **1–3** are particularly suitable for the present investigation for the following reasons: (i) unlike the reactions of many other ambident nucleophiles containing N and O nucleophilic sites,^6,14,20–31^ reactions of **1–3** are not influenced by the presence of a counter-cation,^33^ and (ii) their alkylation products do not undergo secondary reactions (*cf.* amide alkylations).^19*d*,*e*^

**Scheme 2:**
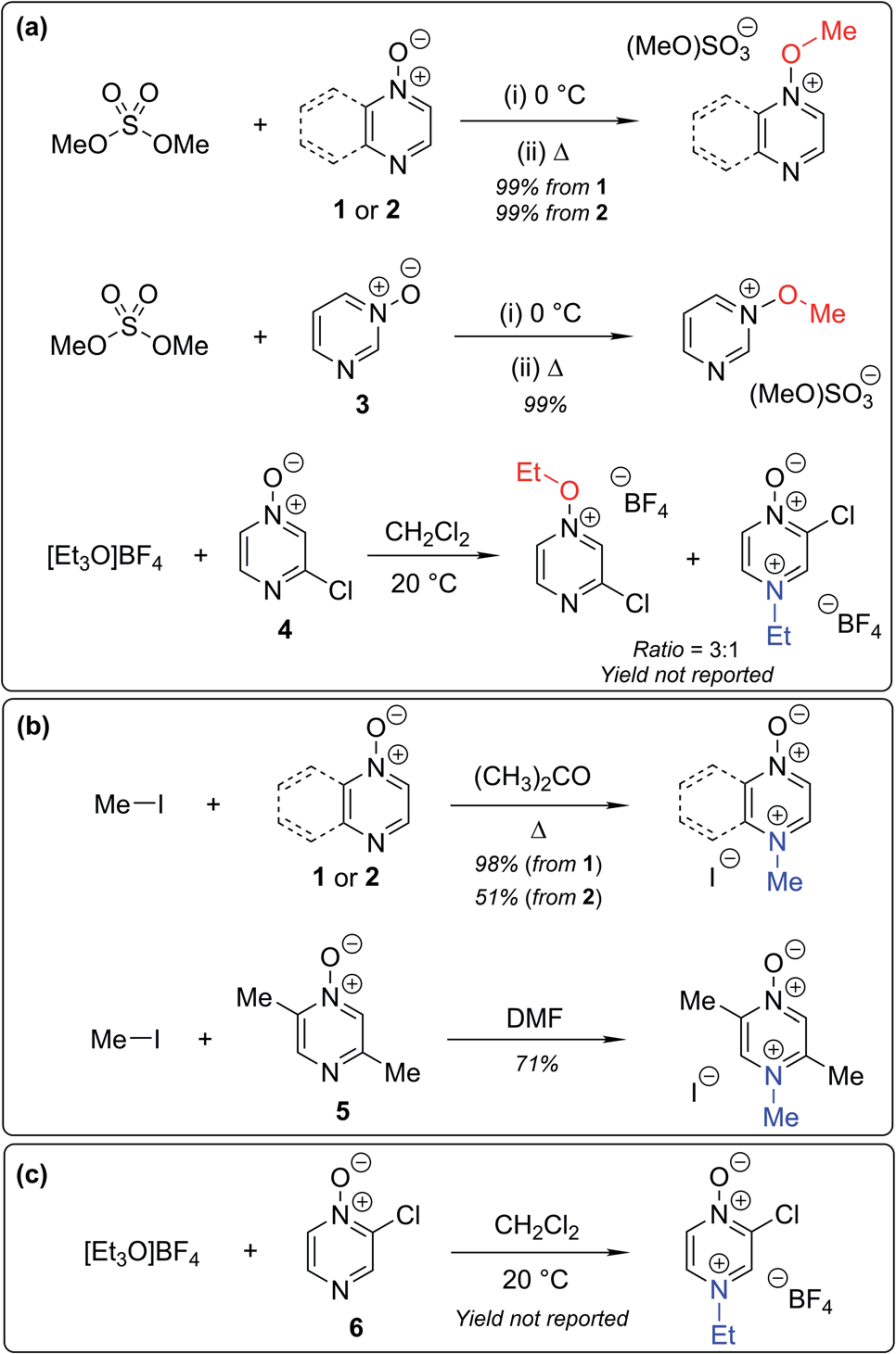
Alkylation of diazine *N*-oxides **1–6** using various hard and soft electrophiles. (a) *O*-alkylation using hard electrophiles,^7,10^ (b) *N*-alkylation using soft electrophiles,^11,12^ (c) *N*-alkylation using a hard electrophile.^10^

There exist several literature precedents of relevance to the ambident nucleophilicity of diazine *N*-oxides. Exclusive *O*-alkylation has been reported to occur in reactions of pyrazine *N*-oxide (**1**), quinoxaline *N*-oxide (**2**) and pyrimidine *N*-oxide (**3**) with hard alkylating agent dimethylsulfate,^7^ and predominant *O*-ethylation has been reported to occur in the reaction of compound **4** with hard electrophile [Et_3_O]BF_4_ (Scheme 2a).^10^ Reactions of **1**, of **2** and of **5** with soft electrophile methyl iodide have been reported to yield *N*-alkylated adducts (Scheme 2b),^11,12^ as has the reaction of **5** with benzyl chloride.^12*c*^ In contrast, compound **6** undergoes exclusive *N*-ethylation on reaction with hard electrophile [Et_3_O]BF_4_ (Scheme 2c).^10^ Notwithstanding the ambiguity inherent in assigning hard and soft sites in these diazine *N*-oxides, it is clear that these results cannot all simultaneously be consistent with the HSAB principle.

An additional fundamental difficulty exists in the context of reactions of diazine *N*-oxides: the act of establishing the structure of the product is itself fraught with ambiguity. The spectral features of the products of *O*-alkylation and *N*-alkylation of a particular diazine *N*-oxide are not necessarily readily distinguishable. Most instances in the literature in which product structures have been assigned have been based on the results of chemical derivatisations,^12^ prior to the development of modern spectroscopic methods. In only one instance (involving two compounds) have modern two-dimensional NMR spectroscopic techniques been used to establish the precise structures of alkylation products of diazine *N*-oxides.^10,34^ Hence, even in instances in which structural assignments have been made, it is not certain that the correct product structures have been identified.

To unambiguously establish the ratios of N *vs.* O selectivity for the alkylation reactions of **1–3**, we took advantage of the technique of indirect detection natural abundance ^1^H–^15^N HMBC NMR spectroscopy.^34–38^ This is an extremely useful diagnostic tool but, is very notably under-exploited – to our knowledge, there are only a handful of examples of its use to establish the site of attachment of an alkyl electrophile to an ambident reactant.^10,31,34,37^ We have also conducted high level quantum chemical calculations to help us in understanding the outcomes of these experiments.

### Background data and reference *δ*_N_ values

**Scheme 3:**
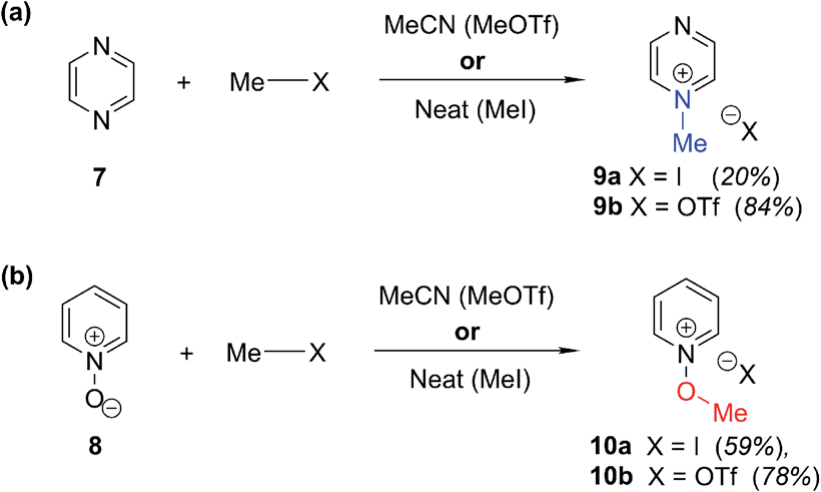
Examples of use of hard and soft methylating agents to effect (a) *N*-methylation of **7**; (b) *O*-methylation of **8**. X = I or OTf throughout. Isolated yields are shown in parentheses.

In order to be able to employ ^1^H–^15^N HMBC NMR spectral data in a diagnostic manner to establish the site of alkylation of ambident nucleophiles **1–3**, we have made use of a set of results described in our recent publication.^39^ In this preliminary study, we carried out various alkylations of representative diazines and azine *N*-oxides (see examples shown in Scheme 3, involving *N*-methylation of **7** and *O*-methylation of **8**), and monitored the change in the ^15^N NMR chemical shifts (referred to as Δ(*δ*_N_) values) of each nitrogen atom in the *N*-alkylated product relative to its *δ*_N_ value in the starting material using ^1^H–^15^N HMBC NMR spectroscopy. We consistently observed that upon *N*-alkylation of diazines, a large upfield shift of the *δ*_N_ value of the alkylated nitrogen atom occurs (*i.e.* Δ(*δ*_N_) ≪ 0 ppm).^40^ In fact, across a total of 22 examples from the chemical literature and our own work, involving *N*-methylation or ethylation of pyridines, diazines, diazine *N*-oxides, quinolines, and isoquinolines, the average upfield Δ(*δ*_N_) value of the alkylated nitrogen atom is −115 ppm.^10,41^ Similarly, the average upfield Δ(*δ*_N_) value associated with *N*-benzhydrylation was −91 ppm (3 examples). In contrast, the shift upfield in the *N*-oxide nitrogen *δ*_N_ value upon *O*-alkylation is significantly smaller – across 7 examples involving *N*-methylation or ethylation, the average upfield Δ(*δ*_N_) value was determined to be only −40 ppm, while for *O*-benzhydrylation the average Δ(*δ*_N_) value was −45 ppm. That the upfield signal in each case belongs to the alkylated nitrogen atom is shown by the existence of a correlation in the ^1^H–^15^N HMBC NMR spectrum of the product between the upfield ^15^N signal and the proton(s) of the *N*- or *O*-alkyl group.

From the above, we can conclude that there is a characteristic Δ(*δ*_N_) value associated with *N*-alkylation of an aromatic N-heterocycle, distinct from (and significantly larger than) the Δ(*δ*_N_) value associated with *O*-alkylation of an aromatic *N*-oxide. Analogous observations have been made in an ^15^N NMR spectroscopic studies of protonation of pyridine and 4-methylpyridine *N*-oxide, which induces Δ(*δ*_N_) values of −113.3 ppm^41*a*^ and −50.1 ppm,^41*b*^ respectively. Furthermore, complexation of aromatic *N*-heterocycles to metals has been shown to result in upfield Δ(*δ*_N_) values of *ca.* −100 ppm.^42^

Our previous investigation also allowed us to determine that in the ^1^H–^13^C HMBC NMR spectra of *N*-alkylated products, three-bond correlations exist between the *N*-alkyl group carbons and hydrogens and the *ortho* carbons and hydrogens of the aromatic moiety.^39^ No correlations were observed in the ^1^H–^13^C HMBC NMR spectra of *O*-alkylated products between the *O*-alkyl group carbons and hydrogens and the *ortho* carbons and hydrogens. Furthermore, these unambiguous NMR spectroscopic correlation methods also allowed us to establish definitive diagnostic trends in the ^13^C NMR chemical shifts of the alkyl group carbons immediately bound to aromatic nitrogen or aromatic *N*-oxide oxygen. For example, the *N*-methyl carbon of the adduct of *N*-methylation of an aromatic nitrogen nucleophile was shown to typically have a *δ*_C_ value in the range 36–53 ppm, while the *O*-methyl carbon of the adduct of aromatic *N*-oxide methylation typically exhibits a *δ*_C_ value in the range 62–75 ppm.^39^ Consequently, it should be possible to employ a combination of Δ(*δ*_N_) values (obtained from ^1^H–^15^N HMBC NMR spectra) together with ^1^H–^13^C HMBC and ^13^C{^1^H} NMR spectroscopic data to distinguish between *N*- and *O*-alkylated diazine *N*-oxides.

## Results

### Site of alkylation of diazine *N*-oxides

The data discussed above show that natural abundance ^1^H–^15^N HMBC is a highly useful diagnostic tool to determine whether or not the site of attachment of an alkyl electrophile is at a nitrogen atom. We will now describe how we have employed the ^1^H–^15^N HMBC NMR technique, in tandem with information from ^13^C{^1^H} and ^1^H–^13^C HSQC and HMBC NMR spectra, to establish the site of alkylation of ambident nucleophiles **1–3** in reactions with representative hard and soft alkylating agents.

**Scheme 4:**
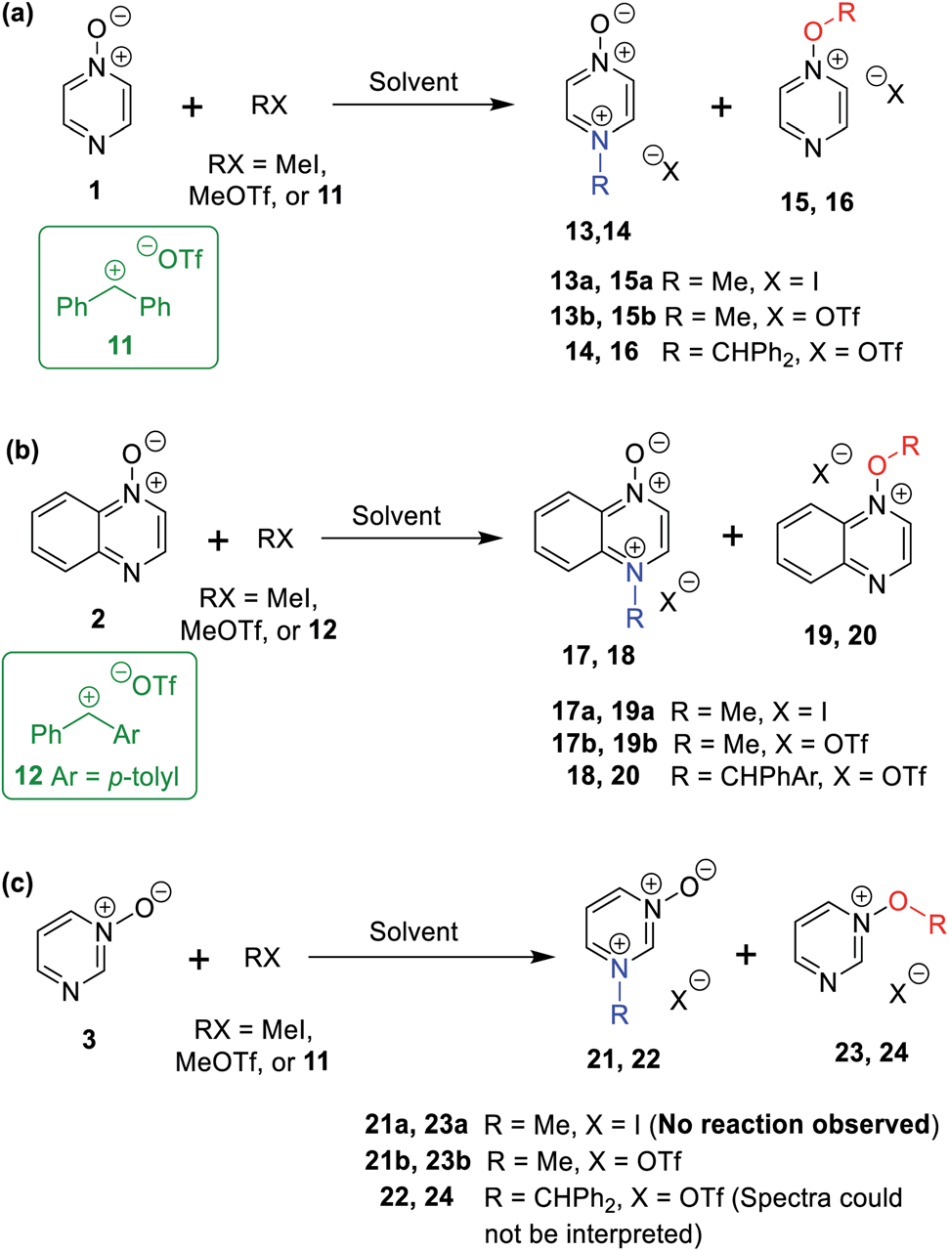
*N*- and *O*-alkylation reactions of ambident nucleophiles **1–3**. Methylation reactions (using MeI or MeOTf) were conducted in (CD_3_)_2_SO, CD_3_CN, or CH_3_CN. Upon completion of reactions in CD_3_CN or CH_3_CN, the solvent was removed, and (CD_3_)_2_SO was added. Benzhydrylation reactions were conducted in CD_2_Cl_2_.^43^ See Table 1 for details of conversions and yields.

Reactions of ambident nucleophiles **1** and **2** with electrophiles MeI, MeOTf, and benzhydrylium triflates **11** and **12** were carried out using the conditions shown in Scheme 4 and Table 1.^44–46^ The reaction of **1** with MeI in CD_3_CN or CH_3_CN resulted in formation of a single product, albeit with low conversion and yield – *i.e*. the process of alkylation was completely selective for one site (N or O) – see Table 1 entry (i). We did not observe any product formation in our ^1^H NMR spectra of the reaction of **2** + MeI in CD_3_CN. Product formation was only observed when the reagents were mixed together in the absence of solvent (neat); the data in Table 1 entry (v) refer to the reaction run under these conditions. As in the case of **1** + MeI, only a single product was observed by ^1^H NMR spectroscopy. Attempted reactions of **3** with MeI in CD_3_CN or MeCN did not yield any products, *i.e.* neither **21a** nor **23a** were observed (Scheme 4c).

**Table 1:** Alkylation reactions of diazine *N*-oxides **1**, **2** and **3** (as per Scheme 4) resulting in formation of *O*- and *N*-alkylated products.^a^ Note that the ^1^H NMR spectra of the reaction mixtures on their own do not show which product (*O vs*. *N*-alkylation) is favoured in each case, only the product ratio

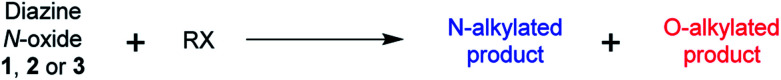								
Diazine *N*-oxide	#	Reaction solvent^a^	R	X	Products		Conversion (isolated% yield)^b^	*N*/*O* product ratio^c^
					*N*-methyl	*O*-methyl		
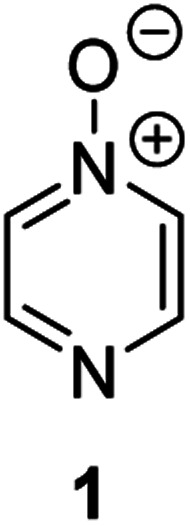	(i)	CD_3_CN or no solvent	Me	I	**13a**	**15a**	Reaction in CD_3_CN: 24% (solvent-free reaction 26%)	>99 : 1
	(ii)	CD_3_CN	Me	OTf	**13b**	**15b**	Quantitative (68% yield of **13b**)^a^	95 : 5
	(iii)	(CD_3_)_2_SO	Me	OTf	**13b**	**15b**	87%	>99 : 1
	(iv)^a^	CD_3_CN or CH_2_Cl_2_^a^	CH_2_Ph	OTf	**14**	**16**	Quantitative^a^	>99 : 1

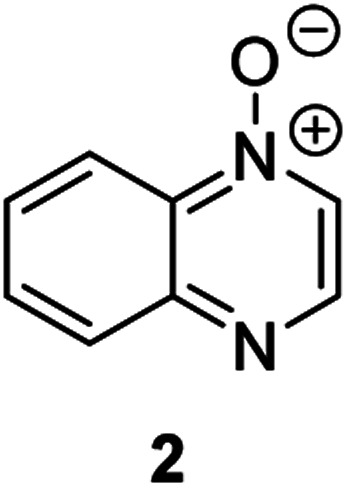	(v)	No solvent	Me	I	**17a**	**19a**	(Yield = 16%)^d^	>99 : 1
	(vi)	CD_3_CN	Me	OTf	**17b**	**19b**	Quantitative (57% yield of **17b**)^a^	89 : 11
	(vii)	(CD_3_)_2_SO	Me	OTf	**17b**	**19b**	78%	>99 : 1
	(viii)	CD_2_Cl_2_	CHPhAr^e^	OTf	**18**	**20**	93%	91 : 9

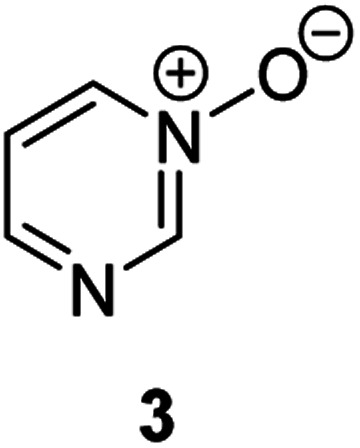	(ix)	CD_3_CN	Me	I	**21a**	**23a**	No products formed	—
	(x)	CD_3_CN	Me	OTf	**21b**	**23b**	Quantitative^a^	7 : 93
	(xi)	(CD_3_)_2_SO	Me	OTf	**21b**	**23b**	76%	7 : 93
	(xii)	CD_2_Cl_2_	CHPhAr^e^	OTf	**22**	**24**	Spectra could not be interpreted	—

a See ESI for experimental conditions employed and details of conversion calculations and yields.^44^

b Conversions represent the combined amount of *N*- and *O*-alkylated product formed relative to the amount added of the alkylating agent (always the limiting reagent). These were determined using integrations of appropriate signals in the ^1^H NMR spectra. For entry (viii), the deviation from quantitative conversion was due to hydrolysis of the alkylating agent. Percentage yields (where applicable) of isolated products were determined from separate reactions run on larger scale using MeCN solvent, or with no solvent (neat reagents) for entries (i) and (v). Products **14**, **18**, **20**, **21b** and **23b** (entries (iv), (viii) and (x), respectively) decompose upon attempted isolation, and hence no isolated yields could be obtained in these cases.

c The identities of the products cannot be determined directly from the ^1^H NMR spectra. Information from other spectra is needed to establish which product is *N*-alkylated and which is *O*-alkylated, and hence to establish the *N*/*O* ratio. See main text for full details.

d **2** + MeI were reacted together without solvent. The product was purified prior to NMR spectral characterisation, so the conversion was not determined for this reaction. However, the low isolated yield shown above is indicative of low conversion in this reaction.

e Ar = *para*-tolyl.

The reaction of **1** with benzhydrylium triflate **11** in CH_2_Cl_2_ or CD_3_CN also resulted in formation of single products (Table 1 entry (iv)).^43^ The ^1^H NMR spectrum of the reaction of **2** + **13** in CD_2_Cl_2_ (Scheme 4b) shows formation of two products in a 91 : 9 ratio (combined conversion = 93%; the remaining 7% was accounted for by hydrolysis product; see (Table 1 entry (viii)). Reaction of **3** with **11** gave ^1^H NMR and ^1^H–^15^N HMBC NMR spectra that we could not interpret,^47^ containing broad and unusually shaped signals – *i.e.* we could not detect formation of **22** or **24** (Scheme 4). We ascribe this to the very low Lewis basicity of **3**, *i.e.* the reaction of **3** + **11** is reversible, and thermodynamically disfavoured.

The reactions of **1–3** with MeOTf in CD_3_CN yielded mixtures of *O*- and *N*-methylation products (Table 1 entries (ii), (vi), and (x)). Addition of MeOTf to (CD_3_)_2_SO solutions of **1** and **2** resulted in formation of a single product in each case (Table 1 entries (iii) and (vii)), while the corresponding reaction of **3** gave two products (Table 1 entry (xi)). The rates of these reactions differed greatly depending on the solvent used. Product formation was rapid for reactions in CD_3_CN (*i.e.* complete within minutes), but was exceptionally slow in (CD_3_)_2_SO, requiring weeks for high conversions to be obtained. It is highly likely that the active methylating agent in (CD_3_)_2_SO was the methoxysulfonium salt [(CD_3_)_2_S(OMe)]OTf,^48–50^ and that this electrophile is much less reactive than MeOTf in MeCN.

Many of the initial products of the reactions of Scheme 4 and Table 1 do not survive attempts at isolation. Hence, all reactions were conducted on small scale, and the entirety of each reaction mixture was transferred (under inert atmosphere) to a NMR tube for analysis by NMR spectroscopy. In instances in which stable, isolable products were formed, the final (stable) products were isolated from separate reactions, conducted on larger scale. The adducts of benzhydrylation of **1** and **2** are hydrolytically unstable and could not be isolated. The adduct of **2** + MeI was formed in very low conversion,^51^ and the adduct of **3** + MeOTf became contaminated with multiple decomposition products;^52^ hence neither adduct could be isolated in pure form. In addition, for the reactions of **1–3** with MeOTf in MeCN or CD_3_CN solvent, decomposition of the minor product (detected in ^1^H NMR spectra in CD_3_CN) occurred upon removal of the MeCN/CD_3_CN solvent under vacuum, resulting in the observation of the signals of the major product only in the ^1^H NMR spectrum of the mixture upon dissolution in (CD_3_)_2_SO.^53^

In all cases shown in Table 1, it was impossible to distinguish the site of attachment of the alkyl group unambiguously using standard ^1^H- or ^13^C-based one or two-dimensional NMR techniques. That is, the identity of the product(s) in each case could not be reliably assigned as *O*-alkylated or *N*-alkylated. In the instances in which mixtures of *O*- and *N*-methylation products were obtained, product ratios could be determined using the integrations of signals in ^1^H NMR spectra, but which product was favoured was not clear. The product ratios determined in this way are shown in Table 1.

In order to determine which site (N or O) of each of the ambident nucleophiles **1–3** is favoured in the alkylation reactions shown in Scheme 4 and Table 1, we made use of the indirect detection natural abundance ^1^H–^15^N HMBC NMR spectroscopic technique described above. The ^15^N NMR chemical shifts of starting compounds **1–3** and of the observed alkylation adducts are shown in Table 2. The Δ(*δ*_N_) values associated with these reactions (also shown in Table 2) show the extent to which the chemical shifts of the ^15^N nuclei of the alkylation product(s) differ from the chemical shifts of the corresponding ^15^N nuclei in the starting materials **1–3**. As above, a negative value of Δ(*δ*_N_) indicates an upfield shift of the *δ*_N_ value of an ^15^N environment upon alkylation, while a positive value indicates a downfield shift. In several instances (all described above), only one product was formed in the alkylation reactions of **1–3**, while in others, the minor product did not survive the process of removal of the MeCN or CD_3_CN reaction solvent and replacement with (CD_3_)_2_SO.^53^ Hence, in almost all cases, only one product could be characterized using the ^1^H–^15^N HMBC NMR technique. In the ^1^H–^15^N HMBC spectrum of the reaction of **2** + **12**, no correlations were observed to the small signals of the minor product that was shown to be present by the ^1^H NMR spectrum. The only instance in which it was possible to determine the *δ*_N_ values of both the major and minor alkylation products involved methylation of **3** in (CD_3_)_2_SO using MeOTf (Scheme 4c; through methoxysulfonium triflate).

**Table 2:** *δ*_N_ and Δ(*δ*_N_) values associated with *N*- and *O*-alkylation reactions of diazine *N*-oxides **1–3** (as per Scheme 4)^a^

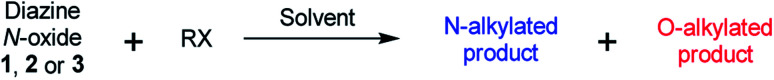										
Diazine *N*-oxide	#	Products	R	X	Reaction solvent/NMR solvent^a^	*δ*_N_ of starting compound (ppm)	*N*-alkylation		*O*-alkylation	
							*δ*_N_ of product (ppm)	Δ(*δ*_N_) (ppm)	*δ*_N_ of product (ppm)	Δ(*δ*_N_) (ppm)
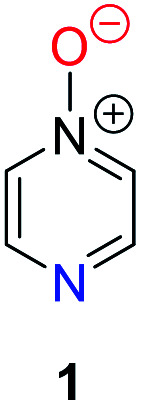	(i)	**13a**, **15a**	Me	I	MeCN/(CD_3_)_2_SO	309.3	322.3	+13.0	Product (**15a**) not formed	
						303.9^b^	187.1	−116.8		
	(ii)	**13b**, **15b**	Me	OTf	MeCN/(CD_3_)_2_SO	309.3	322.9	+13.6	Product (**15b**) decomposed during solvent exchange	
						303.9^b^	187.8	−116.1		
	(iii)	**13b**, **15b**	Me	OTf	(CD_3_)_2_SO	309.3	322.9	+13.6	Product (**15b**) not formed	
						303.9^b^	187.7	−116.2		
	(iv)	**14**, **16**	CH_2_Ph	OTf	CD_2_Cl_2_	311.0	325.0	+14.0	Product (**16**) not formed	
						303.5	201.6	−101.9		

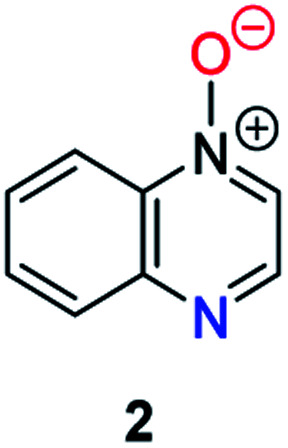	(v)	**17a**, **19a**	Me	I	MeCN/(CD_3_)_2_SO	303.2	314.4	+11.2	Product (**19a**) not formed	
						299.3^c^^,^^d^	178.0	−121.3		
	(vi)	**17b**, **19b**	Me	OTf	MeCN/(CD_3_)_2_SO	303.2	314.4	+11.2	Product (**19b**) decomposed during solvent exchange	
						299.3^c^^,^^d^	177.6	−121.7		
	(vii)	**17b**, **19b**	Me	OTf	(CD_3_)_2_SO	303.2	314.4	+11.2	Product (**19b**) not formed	
						299.3^c^^,^^d^	177.9	−121.4		
	(viii)	**18**, **20**	CHPhAr^e^	OTf	CD_2_Cl_2_	302.0	317.6	+14.4	Signal of **20** not detected in ^1^H–^15^N HMBC	
						300.3	190.5	−108.8		

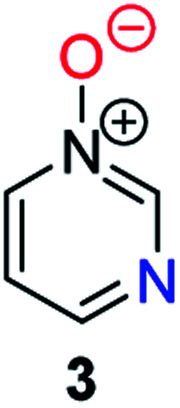	(ix)	**21b**, **23b**	Me	OTf	CD_3_CN/(CD_3_)_2_SO	301.3	Product (**21b**) decomposed during solvent exchange		303.4	+2.1
						291.7			249.4	−42.3
	(x)	**21b**, **23b**	Me	OTf	(CD_3_)_2_SO	301.3	293.6	−7.7	303.1	+1.8
						291.7	205.2	−86.5	249.0	−42.7

a See ESI for experimental conditions employed.^45^

b Literature *δ*_N_ values: 309.33, 303.85 ((CD_3_)_2_SO, referenced to nitromethane at 380 ppm; equivalent to ammonia at 0 ppm).^54^

c These values were reported in ref. 55 as *δ*_N_ −76.8 and −80.7 ppm (referenced to nitromethane at 0 ppm).

d The reported *δ*_N_ values for these signals was from a spectrum referenced to nitromethane at 0.0 ppm. Since our ^1^H–^15^N HMBC spectra were referenced to ammonia at 0 ppm, the literature *δ*_N_ value has been re-calculated here relative to ammonia at 0 ppm.

e Ar = *para*-tolyl.

**Fig. 2:**
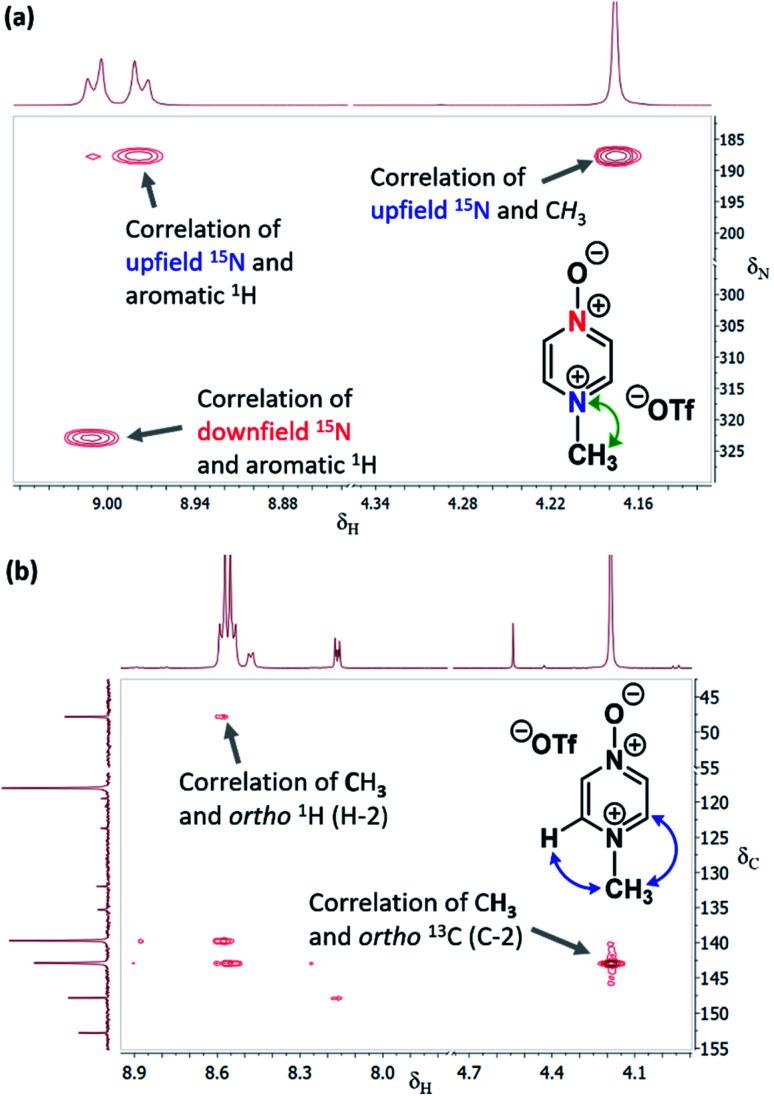
(a) Section of the ^1^H–^15^N HMBC NMR spectrum of **13b** in (CD_3_)_2_SO (from reaction of Table 2 entry (ii)) showing correlation of *N*-methyl ^1^H signal with upfield ^15^N signal, (b) section of the ^1^H–^13^C HMBC NMR spectrum of **13b** in CD_3_CN (from reaction of Table 2 entry (ii)) showing correlations between (i) *N*-methyl ^1^H signal and *ortho*-^13^C signals, and (ii) *ortho*-^1^H signals and *N*-methyl group ^13^C signal.

The ^1^H–^15^N HMBC NMR spectra of the major or exclusive products formed in the reactions of **1** or **2** with electrophiles MeI, MeOTf, and benzhydrylium **11** and **12** (Scheme 4a and b) all show that the *δ*_N_ values of the upfield nitrogen nuclei are shifted upfield by over 100 ppm relative to the *δ*_N_ values of the corresponding nitrogen NMR environments in the starting materials, *i.e.* Δ(*δ*_N_) > −100 ppm in each case (see Table 2 entries (i), (ii), (iii), (v), (vi) and (vii) for methylations and entries (iv) and (viii) for benzhydrylation reactions).^56^ That the upfield signal in the ^15^N dimension belongs to the alkylated nitrogen is confirmed by the existence of a correlation in the ^1^H–^15^N HMBC NMR spectrum of this signal with the ^1^H signal of the *N*-alkyl proton(s) (see example spectrum from the reaction of **1** + MeOTf in Fig. 2a).

In the ^1^H–^13^C HMBC NMR spectra of each of the major products of the reactions of **1** and **2**, a correlation is shown to exist between the alkyl group (aliphatic) proton(s) and the carbons *ortho* to the upfield nitrogen for all alkylation adducts (see example in Fig. 2b). A correlation between the alkyl group aliphatic carbon and the protons *ortho* to the upfield nitrogen is also evident in these spectra. The large upfield Δ(*δ*_N_) values and correlation data associated with the alkylation reactions of **1** and **2** are consistent with the preferential (and in some cases exclusive) occurrence of *N*-alkylation in these reactions.

In support of this conclusion, the ^13^C{^1^H} NMR chemical shifts of the methyl group carbon in the major products of the methylation reactions of **1** and **2** are, respectively, 44.1 and 46.6 ppm.^57^ These values lie in the middle of the range of *δ*_C_ values identified in our previous work as being characteristic of *N*-methylation of aromatic N-heterocycles (*vide supra*).^39^ The *δ*_C_ values of the minor products of these methylation reactions were, respectively, 68.9 and 70.2 ppm. These values appear in the middle of the *δ*_C_ range that is indicative of adducts of *O*-methylated aromatic *N*-oxides.^39,57^ The *δ*_C_ values of the benzhydryl group aliphatic carbons (Ar_2_*C*H) in the products of the benzhydrylation reactions of **1** and **2** were, respectively, 77.2 and 73.2 ppm.^57^ These values are characteristic of *N*-benzhydrylated products, based on our previous work.^39^ The above data are all consistent with the conclusion that the major products formed are *N*-alkylation adducts **13**, **14**, **17** and **18** (Scheme 4a and b). These are formed in preference to *O*-alkylation adducts **15**, **16**, **19** and **20**.

The ^1^H–^15^N HMBC NMR spectrum of the reaction mixture produced by adding MeOTf to a (CD_3_)_2_SO solution of **3** (Scheme 4c) showed signals for the major product at *δ*_N_ 303.1 and 249.0 ppm (Table 2, entry (x)).^58^ The upfield ^15^N NMR signal showed a correlation with the methyl group C*H*_3_ protons, indicating that this belongs to the alkylated nitrogen. However, no correlation existed in the ^1^H–^13^C HMBC NMR spectrum for the signal of the methyl protons with the signal of the carbons *ortho* to the upfield nitrogen, nor for the signal of the methyl carbon with the signal of the protons *ortho* to upfield nitrogen. Based on the *δ*_N_ value of the upfield nitrogen signal, the *δ*_C_ value of the methyl group carbon of 70.2 ppm (characteristic of a N^+^–O–*C*H_3_^13^C NMR signal of a *N*-methoxypyridinium ion),^39^ and the features of the ^1^H–^13^C HMBC NMR spectrum, the spectral characteristics of the major product are very similar to those of compound **10** (the *O*-methylated adduct of pyridine *N*-oxide (**8**); Scheme 3b), and other aromatic *N*-oxide *O*-methylation adducts.^39^

We therefore conclude that the major product of this reaction is *O*-methylation adduct **23b** (Scheme 4c). The upfield signal (*δ*_N_ = 249.1 ppm) is assigned to the *N*–OMe nitrogen atom, and hence has a Δ(*δ*_N_) value of −42.7 ppm relative to the signal of the *N*-oxide nitrogen atom of **3** (at *δ*_N_ = 291.7 ppm; see Table 2 entry (x)), while the downfield signal has Δ(*δ*_N_) = +1.8 ppm relative to the corresponding signal of **3** (*δ*_N_ = 301.3 ppm). The upfield Δ(*δ*_N_) value of −42.7 ppm for this reaction is very similar to the Δ(*δ*_N_) values observed in formation of methoxypyridinium salts during *O*-methylation reactions of *N*-oxides (*e.g.* Δ(*δ*_N_) = −43.6 ppm for formation of **10** from **8** + MeOTf; Scheme 3b).^39^

The Δ(*δ*_N_) value associated with formation of the minor product of the reaction of pyrimidine *N*-oxide (**3**) + [(CD_3_)_2_S(OMe)]OTf in (CD_3_)_2_SO is considerably larger than the Δ(*δ*_N_) value for *O*-alkylation (Table 2 entry (x); Δ(*δ*_N_) = −86.5 *vs.* −42.7 ppm). In addition, the ^1^H–^13^C HMBC NMR spectrum exhibits multiple bond correlations between the *N*-methyl group and *ortho* aromatic ^1^H and ^13^C signals.^59^ The *δ*_C_ value of the methyl group carbon of the minor product was 46.6 ppm,^57^ which is characteristic of an aromatic N^+^–*C*H_3_ carbon (*vide supra*).^39^ These data are consistent with the minor product being *N*-methylation adduct **21b** (Scheme 4c). Our spectral data on the reaction of **3** + MeOTf in CD_3_CN (or MeCN) also show that **23b** is the major product formed in this solvent.^54^ Although **21b** is formed in the reaction (as shown by ^1^H NMR spectral analysis), it does not survive the process of solvent removal and dissolution in (CD_3_)_2_SO (*vide supra*).

Based on the above data, we can conclude that the *N*- *vs. O*-methylation ratios in the reactions of **3** with MeOTf (in CD_3_CN) and [(CD_3_)_2_S(OMe)]OTf in (CD_3_)_2_SO are both 7 : 93 (in favour of *O*-methylation; see Table 1 entries (x) and (xi)).

### Crossover experiments

The *N*- *vs. O*-alkylation ratios observed in the reactions of **1–3** did not change over time in the absence of perturbation. In order to establish whether or not these reactions occurred under kinetic control, we carried out several crossover experiments involving reactions of MeOTf with **1–3** (and of MeI with **1**) in CD_3_CN followed by addition of a second nucleophile.^60^ An internal standard (1,3,5-trimethoxybenzene) was added to the reaction mixture to allow the amounts of the products present to be quantified (using integrations of ^1^H NMR spectral signals of the products) before and after addition of the second nucleophile, and to enable quantification of the amount of crossover product formed. Nucleophiles **7** and **25** were selected as second nucleophiles because they have been shown in separate studies to be considerably stronger Lewis bases than compounds **1–3**,^61^ and hence are expected to out-compete **1–3** for any free alkylating agent present due to (i) their stronger nucleophilicity and (ii) the fact that they are present in considerable excess over **1–3** under the conditions of the crossover experiment.

**Scheme 5:**
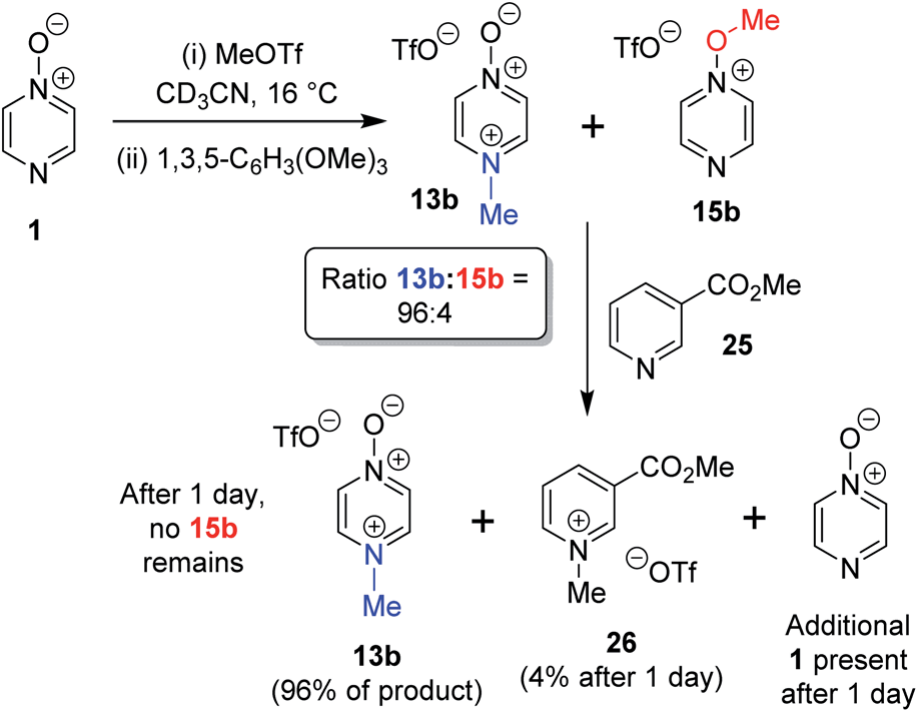
Crossover experiment investigating reversibility of reaction of **1** + MeOTf using 1,3,5-trimethoxybenzene as internal standard, and “crossover nucleophile” **25**. The crossover product is compound **26**.^62^

We observed that the amount of major product formed in the methylation reactions of each of **1** and **2** remained constant with respect to the internal standard during the crossover experiments, *i.e.* the formation of the major product in each case is irreversible (*i.e.***13a**, **13b**, and **17b** respectively). For example, the amount of **13b** formed in the reaction of **1** + MeOTf in CD_3_CN at 16 °C is invariant at 96% of methylation product throughout the experiment (Scheme 5). In the reactions of **1** and **2** with MeOTf (using **25** or **7** as the second nucleophile), crossover product formed at the expense of the minor product (*O*-methylation adducts **15b** and **19b**) with commensurate production of starting diazine *N*-oxide (**1** or **2**). Although crossover product (**9b** or **26**) is formed from the minor products in these experiments, we conclude in each case that this is a consequence of the occurrence of an S_N_2 reaction between the second nucleophile (**7** or **25**) and the minor product. If this were not the case, then repeated observations of the *N*/*O*-methylation ratios over time in alkylation reactions of **1** and **2** should show this ratio changing (to favour the major product), since formation of the major product is irreversible in each case. Consequently, we conclude that *O*-methylation of **1** and **2** are also irreversible processes in CD_3_CN solvent at ambient temperatures. Thus, *N*-methylation of each of **1** and **2** is observed this is the kinetically favoured process in each instance.

A similar crossover experiment involving the reaction of pyrimidine *N*-oxide (**3**) + MeOTf in CD_3_CN (with an internal standard added) and pyrazine (**7**) as 2nd nucleophile also showed formation of crossover product **9b**. In ^1^H NMR spectra of this reaction mixture recorded early in the reaction, the crossover product (**9b**) was observed to form primarily at the expense of *N*-methylation product **21b** (minor product of this reaction), but some *O*-methylation product (**23b**) was also consumed.^62^ An amount of **3** formed that was commensurate with the amount of **9b** produced. After several days, further crossover product was observed to form at the expense of major product **23b**.^62^ It is not clear from these experiments whether formation of **21b** and **23b** from **3** + MeOTf is reversible, *i.e.* whether **7** reacts with MeOTf formed by reversal of **21b** and/or **23b** to **3** + MeOTf, or whether crossover product **9b** is formed by direct S_N_2 reactions of **7** with **21b** and/or **23b**.

### Computational investigations

Our experimental investigations indicate that ambident nucleophiles pyrazine *N*-oxide (**1**) and quinoxaline *N*-oxide (**2**) (with competing N and O nucleophilic sites) undergo preferential alkylation on nitrogen regardless of the nature of the alkylating agent used, *i.e*. independent of whether the electrophile is hard or soft. Ambident nucleophile pyrimidine *N*-oxide (**3**), by contrast, has been shown to undergo preferential *O*-methylation by MeOTf. In order to be able to understand and rationalise the outcomes of the reactions described above, high level quantum chemical calculations at the DLPNO-CCSD(T)/def2-TZVPPD/SMD(CH_3_CN)//M06-2X-D3/6-311+G(d,p)/SMD(CH_3_CN) level of theory were carried out to determine the relative Gibbs energies of the reactants, transition states and products of the reactions of each of compounds **1**, **3**, **7** (pyrazine), and **8** (pyridine *N*-oxide) (structures shown in Fig. 1 and Scheme 3) with MeI and MeOTf.^63^ The reactions of pyrimidine (**27**) and pyridine (**28**) with MeI and MeOTf were also investigated in the same manner. The computational results can be used to estimate the Gibbs energy of activation (Δ*G*^‡^) and standard enthalpy and Gibbs energy of reaction (Δ_r_*H*° and Δ_r_*G*°, respectively) for each process. The accuracy and predictive capability of this computational method have been verified by the close agreement of the Δ*G*^‡^ values determined experimentally and computationally for the reaction of pyrazine *N*-oxide (**1**) with MeI (*vide infra*). The results of the computational investigations of the methylation reactions of **7**, **8**, **27** and **28** are presented in Table 3 (left side). Compounds **7**, **27** and **28** undergo *N*-methylation, and compound **8** undergoes *O*-methylation. These results allow us to see representative values of Δ*G*^‡^, Δ_r_*H*° and Δ_r_*G*° for *N*- and *O*-methylation reactions in which there is no ambiguity over the site of methylation.

**Table 3:** Calculated Δ*G*^‡^, Δ_r_*H*° and Δ_r_*G*° values for methylation of nucleophiles **1**, **3**, **7**, **8**, **27**, and **28** by MeI and MeOTf in CH_3_CN^a^^,^^b^

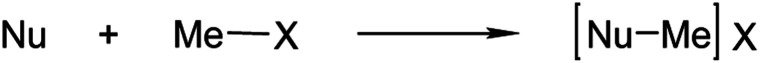															
Nucleophiles with single alkylation site^c^								Ambident nucleophiles							
#	Nu	X	Product & number		Δ*G*^‡^	Δ_r_*G*°	Δ_r_*H*°^b^	#	Nu	X	Product & number		Δ*G*^‡^	Δ_r_*G*°	Δ_r_*H*°^b^
(i)	**7**	I	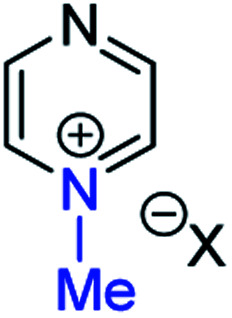	**9a**	+131	−21	−37	(ix)	**1**	I	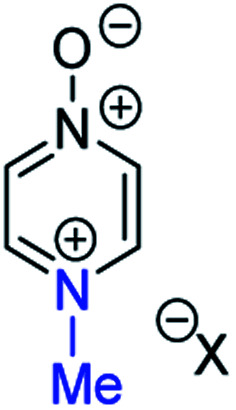	**13a**	+133	−20	−37
(ii)	**7**	OTf		**9b**	+107	−90	−90	(x)	**1**	OTf		**13b**	+108	−88	−90
(iii)	**8**	I	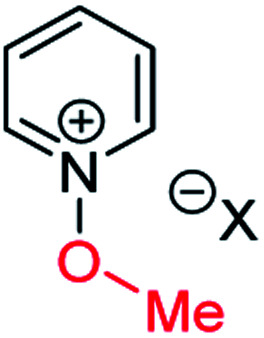	**10a**	+123	−7	−24	(xi)	**1**	I	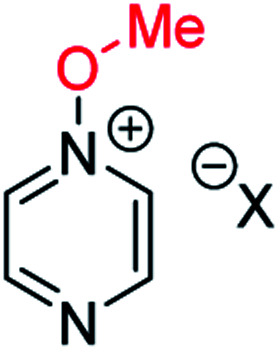	**15a**	+140	+31	+14
(iv)	**8**	OTf		**10b**	+97	−75	−76	(xii)	**1**	OTf		**15b**	+115	−38	−38
(v)	**27**	I	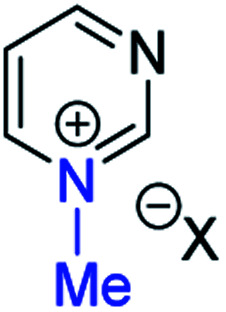	**29a**	+130	−23	−39	(xiii)	**3**	I	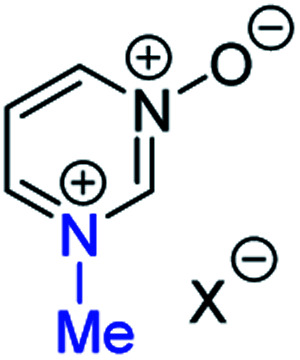	**21a**	+138	+4	−13
(vi)	**27**	OTf		**29b**	+106	−91	−91	(xiv)	**3**	OTf		**21b**	+113	−64	−66
(vii)	**28**	I	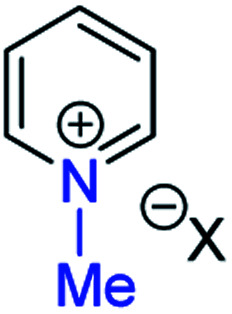	**30a**	+120	−48	−64	(xv)	**3**	I	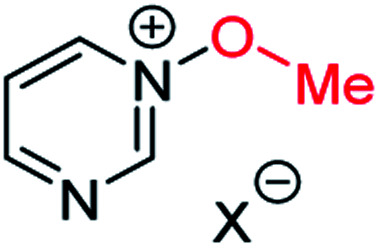	**23a**	+127	+21	+3
(viii)	**28**	OTf		**30b**	+96	−117	−117	(xvi)	**3**	OTf		**23b**	+103	−48	−49

a Enthalpies and Gibbs energy values (in kJ mol^−1^) were calculated at the DLPNO-CCSD(T)/def2-TZVPPD/SMD(CH_3_CN)//M06-2X-D3/6-311+G(d,p)/SMD(CH_3_CN) level of theory.

b Δ_r_*S*° values calculated for these reactions were similar across all reactions of MeI (Δ_r_*S*° = −55 ± 2 J K^−1^ mol^−1^), and across all reactions of MeOTf (Δ_r_*S*° = −2 ± 2 J K^−1^ mol^−1^). These data are included in Tables S1–S3 in the ESI, along with calculated Δ*H*^‡^ and Δ*S*^‡^ values for these reactions.^68^

c Pyrazine (**7**) and pyrimidine (**27**) clearly have two possible alkylation sites, but the sites are identical by symmetry.

Unsurprisingly, the reactions involving MeOTf have systematically smaller calculated Δ*G*^‡^ values and are more exergonic than the reactions involving MeI. The values of Δ*G*^‡^ and Δ_r_*G*° for methylation of **7** by MeI are very similar to the corresponding values for **27** (Table 3 entries (i) and (v)). The Δ*G*^‡^ and Δ_r_*G*° values for the reactions of **7** and **27** with MeOTf are also very similar (Table 3 entries (ii) and (vi)). This suggests that the nucleophilicities and Lewis basicities of **7** and **27** are very similar. The reactions involving pyridine (**28**; Table 3 entries (vii) and (viii)) were found computationally to be both more kinetically and thermodynamically favourable than the corresponding reactions of **7** and **27** with the two methylating agents.^64^ Our calculations indicate that the *O*-methylation reactions of **8** are more kinetically favourable than the corresponding reactions of **7** and **27**, despite being less thermodynamically favourable than those reactions (compare Table 3 entry (iii) with entries (i) and (v), and entry (iv) with entries (ii) and (vi)).

The reaction of pyrazine *N*-oxide (**1**) with MeOTf was found computationally to result in kinetically and thermodynamically preferred *N*-methylation (compare Table 3 entries (x) and (xii)). This calculation indicates that methylation of **1** by MeOTf is an irreversible process at room temperature (regardless of the site of methylation), in agreement with the results of our crossover experiments (see above). The relative magnitudes of Δ*G*^‡^(N) and Δ*G*^‡^(O) calculated for this reaction suggest that a small amount of *O*-methylated product (*ca.* 5–7%) should be produced, as is observed experimentally (*N*/*O* methylation ratio = 95 : 5 for reaction at 20 °C; see Table 2 entry (ii)).^65^

The reaction of **1** with MeI was also found to result in kinetically and thermodynamically preferred *N*-methylation (compare Table 3 entries (ix) and (xi)), which is consistent with the results of our crossover experiments. This reaction has been observed experimentally to be very slow. Only a small amount of conversion had occurred after several days, consistent with the high activation barrier found computationally (shown in Table 3) and determined through a kinetic investigation (described below). In contrast to the reaction of **1** with MeOTf (above), *O*-methylation of **1** by MeI was found computationally to be thermodynamically disfavoured and therefore reversible (Table 3 entry (xi)). No *O*-methyl adduct (**17a**) was observed experimentally for this reaction, which is consistent with kinetically disfavoured and reversible *O*-methylation.

The Δ*G*^‡^(N) and Δ_r_*G*°(N) values for *N*-methylation of **1** (by MeOTf or MeI) are similar to the corresponding values for diazines **7** and **27** (compare Table 3 entry (x) with entries (ii) and (vi), and entry (ix) with entries (i) and (v)). In contrast, the Δ*G*^‡^(O) and Δ_r_*G*°(O) values for *O*-methylation of **1** (by MeOTf or MeI) are significantly less favourable than the corresponding reactions of *N*-oxide **8** (compare Table 3 entry (xii) with entry (iv), and entry (xi) with entry (iii)). The implication of this is that the oxygen site of **1** is deactivated relative to the oxygen site of **8**, both as a nucleophile and as a Lewis base.^66^

Our calculations on the reaction of pyrimidine *N*-oxide (**3**) with MeOTf indicate that, despite the fact that *N*-methylation (formation of **21b**) is thermodynamically favoured over *O*-methylation (formation of **23b**), the kinetically preferred process in this reaction is *O*-methylation (compare Table 3 entries (xiv) and (xvi)). The difference between the calculated values of Δ*G*^‡^(N) and Δ*G*^‡^(O) suggests that a small amount of *N*-methylation (*ca.* 1–3%) should occur. These results are in quite close agreement with the experimental observations – *O*-methylation is indeed favoured, and approximately 7% of the product formed is *N*-methylation adduct **21b** (in CD_3_CN or (CD_3_)_2_SO; see Table 2 entries (ix) and (x)).^67^ These calculations indicate that both reactions are essentially irreversible (however, see the results of our crossover experiment involving **3** + MeOTf above).^63^ Our calculations on the reaction of **3** with MeI indicate that both *O*- and *N*-methylation (formation of **23a** and **21a**, respectively) are reversible. *O*-Methylation was found to be kinetically preferred, again despite the fact that this process is less thermodynamically favourable than *N*-methylation (compare Table 3 entries (xiii) and (xv)). As no product formation was observed experimentally when this reaction was attempted in CD_3_CN or MeCN, it is not possible to verify the applicability of these particular computational results.

**Scheme 6:**
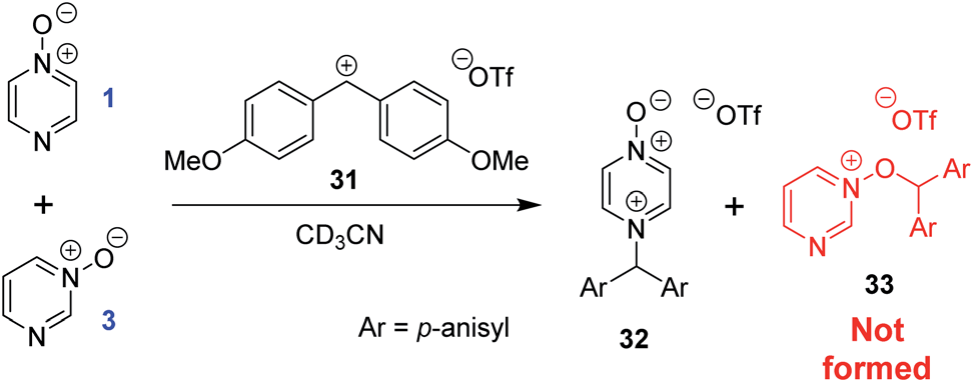
Competition experiment between reversible reactions of **1** and **3** with benzhydrylium ion **31**.^44^

The calculated Gibbs energies of activation for *N*- and *O*-methylation of pyrimidine *N*-oxide (**3**) by MeI or MeOTf, while higher than the Δ*G*^‡^ values for comparable reactions of similar compounds (*e.g.* pyrazine *N*-oxide (**1**), pyrazine (**7**), pyridine *N*-oxide (**8**) and pyridine (**27**)), are not especially different to those Δ*G*^‡^ values (compare Table 3 entry (xiv) with entries (ii) and (vi), entry (xiii) with entries (i) and (v), entry (xvi) with entry (iv), and entry (xv) with entry (iii)). However, comparison of the Δ_r_*G*° values for the same reactions indicates that both *O*- and *N*-methylation reactions of pyrimidine *N*-oxide (**3**) are far less thermodynamically favourable than the corresponding reactions of **1**, **7**, **8** and **27**. This computational observation has been verified experimentally through a thermodynamic competition experiment in which product **32** (derived from pyrazine *N*-oxide (**1**) in a reversible reaction) is formed to the complete exclusion of **33** (derived from pyrimidine *N*-oxide (**3**)) when **1**, **3** and benzhydrylium ion **31** are mixed in CD_3_CN (Scheme 6). It seems that the O and N nucleophilic/Lewis basic sites of **3** are deactivated in a similar manner to the O site of **1**.^66^

According to our computational data, *N*-methylation of both **1** and **3** results in a minor shortening of the *N*-oxide N–O bond. The calculated N–O bond lengths of diazine *N*-oxides **1** and **3** and *N*-methyldiazinium cations **13** and **21** are, respectively, 1.27 Å, 1.29 Å, 1.25 Å and 1.27 Å.^63^*O*-Methylation of **1** and **3** results in a lengthening of the N–O bond (to 1.36 Å for each of **15** and **23**, the *O*-methylated cationic derivatives of **1** and **3**).^63^*O*-methylation of **1** or **3** removes the favourable electrostatic interaction between N and O, and also diminishes the partial resonance of the *N*-oxide with the aromatic system, thereby removing resonance stabilisation effects that may help to stabilise the positive charge in the product. This may contribute to making *N*-methylation of **1** and **3** more thermodynamically favourable than *O*-methylation.

Finally, for completeness, we will comment on the values of the other thermodynamic functions associated with the above reactions. Computationally determined values of Δ_r_*S*° do not differ greatly from each other across all reactions of MeI with **1**, **3**, **7**, **8**, **27** and **28**, or across all reactions of MeOTf with the same nucleophiles, regardless of whether *N*- or *O*-methylation is occurring.^68^ Across all reactions of MeI in Table 3, Δ_r_*S*° remains constant around −55 ± 2 J K^−1^ mol^−1^, while a value of −2 ± 2 J K^−1^ mol^−1^ was observed across the reactions of MeOTf (using 99% confidence intervals).^68^ Therefore, the computational data suggest that enthalpy changes are primarily responsible for dictating the differences between the Δ_r_*G*° values in the various reactions in Table 3. It is not possible to unambiguously ascribe the differences in Δ_r_*H*° to specific effects, and hence we refrain from doing so.

### Activation barrier calculations using Marcus theory

Noting the deficiencies of the HSAB principle, Mayr and co-workers have advanced Marcus theory for rationalising the outcomes of reactions of ambident nucleophiles.^4^ The Marcus equation (eqn (1)) allows Δ*G*^‡^ to be separated out into its contributions from Δ_r_*G*° (the standard Gibbs energy of reaction) and Δ*G*^‡^_0_, the Marcus intrinsic barrier.^69–71^1
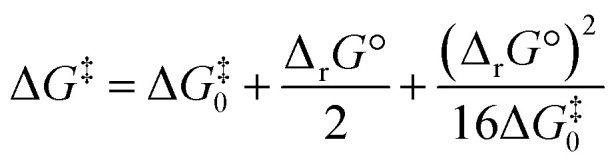


In reactions of ambident nucleophiles with competing sites of differing nucleophilicity, the different nucleophilic sites have different values of each of Δ*G*^‡^_0_ and Δ_r_*G*°. Mayr and co-workers have suggested that the selectivities in such reactions can be rationalised through an appraisal of the factors that influence the values of the two parameters in the Marcus equation (Δ*G*^‡^_0_ and Δ_r_*G*°).^4^ They have employed this approach to qualitatively rationalise the outcomes of reactions of a variety of ambident nucleophiles.^4,72^ In order to build up a more comprehensive understanding of the factors that influence selectivity in reactions of **1–3**, we have calculated values of Δ*G*^‡^_0_ and Δ_r_*G*° for these reactions, and used them to construct values of the activation barriers (Δ*G*^‡^) using the Marcus equation.

Using the procedure described in detail in the ESI,†^73^ values of the intrinsic barrier (Δ*G*^‡^_0_) were calculated for each of the reactions of compounds **1** and **3** with MeI and MeOTf. The Δ*G*^‡^_0_ values for reactions of **1** and **3** are shown in Table 4.^74^ It is noteworthy that, for both ambident nucleophiles **1** and **3**, the intrinsic barrier for methyl transfer to oxygen (Δ*G*^‡^_0_(O)) is lower than that for methylation of nitrogen (Δ*G*^‡^_0_(N)) – *e.g.* compare Table 4 entries (iii) and (i), and entries (vii) and (v). Hoz and co-workers previously established through computational investigations that the Δ*G*^‡^_0_ values associated with reactions of nucleophiles centred on 2nd row elements depend on the identity of the element at the nucleophilic site, with Δ*G*^‡^_0_ decreasing in the order C > N > O > F, *i.e.* from left to right across the periodic table.^75^ The lower intrinsic barriers (intrinsic preference) for *O*-alkylation over *N*-alkylation we observe for **1** and **3** are in line with this general trend.

**Table 4:** Values of intrinsic barriers (Δ*G*^‡^_0_) and derived values of Δ*G*^‡^ for methylation reactions of nucleophiles **1**, **3**, **7**, **8**, **27**, and **28** in CH_3_CN, calculated using the Marcus equation (eqn (1)) using values of Δ_r_*G*° from Table 3 (reproduced here)^a^^,^^b^

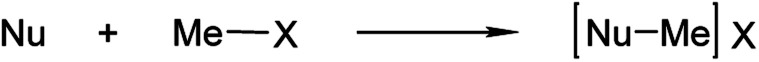						
Nucleophile	#	X	Δ*G*^‡^_0_	Δ_r_*G*°	DFT Δ*G*^‡^	Marcus Δ*G*^‡^
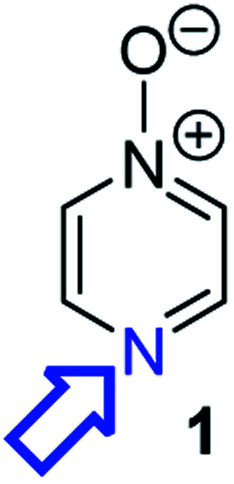	(i)	OTf	+149.5	−88	+108.0	+108.7
	(ii)	I	+144.0	−20	+133.0	+134.2
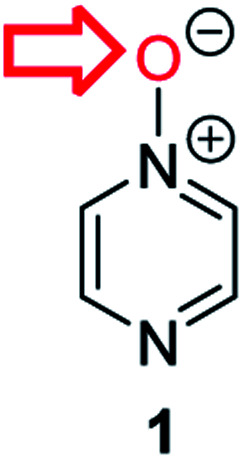	(iii)	OTf	+132.5	−38	+115.0	+114.3
	(iv)	I	+127.0	+31	+140.0	+143.0
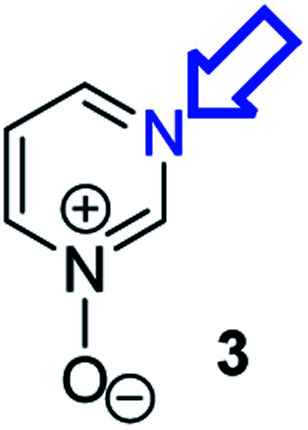	(v)	OTf	+145.0	−64	+113.0	+114.8
	(vi)	I	+139.5	+4	+138.0	+141.5
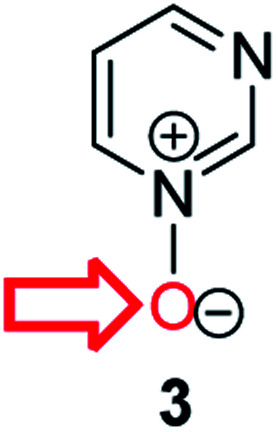	(vii)	OTf	+124.0	−48	+103.0	+101.2
	(viii)	I	+118.5	+21	+127.0	+129.2

a The site of methylation of each nucleophile is indicated by an arrow. The Gibbs energy values have units of kJ mol^−1^.

b Δ_r_*G*° and Δ*G*^‡^ (DFT Δ*G*^‡^) values here are reproduced from Table 3.

Substitution of the calculated Δ*G*^‡^_0_ values into eqn (1) (the Marcus equation) along with the values of Δ_r_*G*° calculated as described above (Table 3 and associated discussion; these Δ_r_*G*° values are reproduced in Table 4 to aid the understanding of the reader) allows values of Δ*G*^‡^ to be calculated using the Marcus equation. Comparison of the Δ*G*^‡^ values obtained using the Marcus equation (shown in Marcus Δ*G*^‡^ column in Table 4) with the Δ*G*^‡^ values directly calculated as described above (values from Table 3, labelled DFT Δ*G*^‡^, are reproduced in Table 4) shows a close correspondence between the two methods. Importantly, the experimentally observed N *vs.* O selectivities for the reactions of the ambident nucleophiles **1** and **3** are reproduced quite closely by both methods of calculation.^18^ Analysing how the factors that contribute to the Gibbs energy of activation for a reaction influence its magnitude (*i.e.* how the interplay between Δ*G*^‡^_0_ and Δ_r_*G*° influences Δ*G*^‡^) provides a very useful means of understanding the origins of the differences between the rates of different reactions. Nowhere is this more apposite than in understanding which nucleophilic site of an ambident nucleophile is kinetically preferred. A full analysis of this kind for the reactions of **1** and **3** will be described in detail below.

The applicability of Marcus theory has been challenged in recent years,^76^ and alternatives have been suggested.^77,78^ However, such alternatives also incorporate in some manner an intrinsic barrier or a proxy thereof. In addition to using the Marcus equation, we have also used an adaptation of the Zhu equation (see the ESI†)^79^ to calculate Δ*G*^‡^ values for the methylation reactions of nucleophiles **1** and **3**. The Δ*G*^‡^ values calculated using the adapted Zhu equation are very similar to the values calculated using eqn (1) (see Table S5 in the ESI†).^73^

The experimentally observed ratio of *N*- to *O*-methylation for the reaction of **1** + MeOTf was 95 : 5 (Table 2). Direct calculation of the Δ*G*^‡^ values at the DLPNO-CCSD(T)/def2-TZVPPD/SMD(CH_3_CN)//M06-2X-D3/6-311+G(d,p)/SMD(CH_3_CN) level of theory indicated a N/O ratio of 94 : 6 for this reaction, while calculation of the N/O ratio using the Marcus equation gave a ratio of 90 : 10 (compare Table 4 entries (i) and (iii)). Use of the Zhu equation gave a N/O ratio of 96 : 4.^73^ The experimentally observed ratio of *N*- to *O*-methylation for the reaction of **3** + MeOTf was 7 : 93. Our calculations indicated a ratio of 2 : 98 for this reaction, while calculation of the N/O ratio using the Marcus equation gave a ratio of 0.4 : 99.6, (compare Table 4 entries (v) and (vii)) and calculation using the Zhu equation gave a ratio of 0.5 : 99.5.^73^ That the experimental selectivities (in *N*- *vs. O*-methylations of **1** and **3** by MeOTf) are reproduced quite closely using the Marcus and Zhu equations^73^ and direct computation indicates that these methods are highly useful in understanding the factors that control Gibbs energies of activation in nucleophilic substitution reactions.

### Experimental verification of accuracy of calculated Δ*G*^‡^

**Scheme 7:**
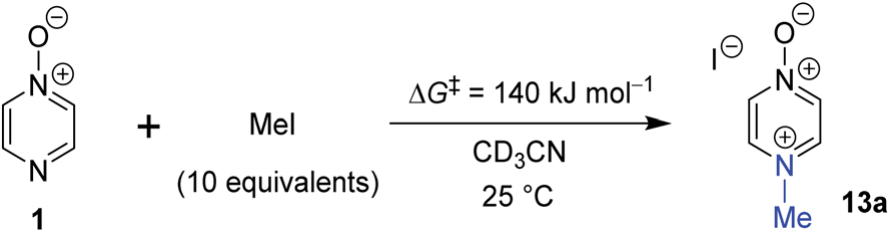
The reaction of **1** + MeI in CD_3_CN at 25 °C under pseudo-first order conditions (excess MeI) was monitored by ^1^H NMR spectroscopy to enable determination of an approximate Δ*G*^‡^ value for the reaction at 25 °C.

In order to verify the applicability of the computational methods discussed above to determine the magnitudes of activation barriers, we conducted a kinetic investigation on the reaction of pyrazine *N*-oxide (**1**) with MeI in CD_3_CN at 25 °C using ^1^H NMR spectroscopy to determine the concentrations of the reactants and product (**13a**). The experiment was conducted under pseudo-first order conditions, with MeI present in ten-fold excess over **1**. Using the method described in detail in the ESI,†^80^ we determined an approximate Δ*G*^‡^ value for this reaction of 1.4 × 10^2^ kJ mol^−1^. This value is within 5% of the Δ*G*^‡^ values predicted for this reaction using the Marcus equation (134.2 kJ mol^−1^), and using direct application of the DLPNO-CCSD(T)/def2-TZVPPD/SMD(CH_3_CN)//M06-2X-D3/6-311+G(d,p)/SMD(CH_3_CN) method (133 kJ mol^−1^). This striking agreement between computational theory and experiment demonstrates that these computational methods are capable of modelling kinetic phenomena of this type rather accurately (Scheme 7).

## Discussion

### Rationalisation of experimental N *vs.* O selectivities

The kinetic preference of compound pyrazine *N*-oxide (**1**) for *N*-methylation by soft electrophile MeI (forming compound **13a**) and by hard electrophile MeOTf (forming compound **13b**) has been demonstrated experimentally and computationally. The alkylation reactions of quinoxaline *N*-oxide (**2**) by MeI, MeOTf and benzhydrylium triflates (**11** or **12**) and of **1** by **11** or **12** are all also almost certainly irreversible, and all yield *N*-alkylated products preferentially or exclusively. The reaction of pyrimidine *N*-oxide (**3**) + MeOTf gives *O*-methylated product (**23b**) predominantly, and our computational investigations indicate that this is due to the kinetic favourability of formation of **23b**. Although no product formation is observed in the reaction of **3** + soft electrophile MeI (due to the formation of products **21a** and **23a** being thermodynamically disfavoured and hence reversible), our computational results indicate that *O*-methylation (formation of **23a**) is the kinetically favoured process in this reaction (see Table 4 entries (vi) and (viii)).

It is evident from these results that each nucleophile exhibits a preferred site of alkylation which is independent of the nature of the electrophile used (N for **1** and **2**, and O for **3**), *i.e.* these outcomes cannot be dictated by hard/soft acid/base interactions. A fundamentally different set of factors must dictate the observed selectivities in these reactions. We discuss an alternative rationale to account for these observations later in this article.

Although the above evidence clearly shows that the HSAB principle does not apply in this set of reactions, and thereby renders unnecessary the identification of which nucleophilic site of each of **1–3** is “harder” and which is “softer”, it is nonetheless appropriate at this point to discuss the difficulty and ambiguity inherent in attempts at such identifications. The features that are employed to determine whether a reactant is hard or soft are charge (charge density), size, polarizability and electronegativity.^2*a*,*b*,*g*,18*b*,*c*^ For hard bases, the donor atom is typically negatively charged and/or has a local excess of electron density, and is of small size, low polarizability and high electronegativity. For soft bases, the donor atom typically does not bear a formal negative charge and exhibits low negative charge density, and is of large size, high polarizability and low electronegativity. Derivation of functions that reliably indicate the “local hardness” and “local softness” of sites in a molecule (such as an ambident nucleophile) has proved a difficult endeavour.^15^ At present, such approaches cannot be applied without ambiguity.

On the basis that oxygen is more electronegative than nitrogen, one could perhaps anticipate that the oxygen site of a diazine *N*-oxide such as **1–3** should be harder than the nitrogen site. However, although there is a formal negative charge on the *N*-oxide oxygen atoms in these compounds, it is not clear which nucleophilic site in each ambident nucleophile should have the highest negative charge density, thereby potentially complicating the issue. To probe this question, we calculated the charge distribution for the ambident *N*-oxides with a variety of methods (ChelpG, Merz–Singh–Kollman, natural bond order (NBO), and atoms in molecules (AIM)),^81^ but found that there was no uniform agreement between methods on which site bears the highest negative charge density in compounds **1** and **3**. Full details of this are given in the ESI.†^81^

We now present an alternative rationale, based on Marcus theory, to explain these results (see eqn (1) above). In the following discussion, the intrinsic barriers for alkylation at oxygen and nitrogen are referred to, respectively, as Δ*G*^‡^_0_(O) and Δ*G*^‡^_0_(N). The standard Gibbs energies of reaction for *O*- and *N*-alkylation are referred to, respectively, as Δ_r_*G*°(O) and Δ_r_*G*°(N).

Although *O*-methylation is intrinsically preferred over *N*-methylation (for diazine *N*-oxides, and in general; *vide supra*),^75^ in reactions of **1** and **2**, the intrinsic preference for *O*-alkylation is modest. Δ*G*^‡^_0_(O) is calculated to be only 17 kJ mol^−1^ lower than Δ*G*^‡^_0_(N) for the reactions of **1** with MeI or MeOTf (Table 4 entry (i) *vs.* (iii), and entry (ii) *vs.* (iv)). The Δ_r_*G*°(N) values for these reactions are substantially more favourable than the corresponding Δ_r_*G*°(O) values. Consequently, the very favourable contribution of Δ_r_*G*°(N) to Δ*G*^‡^(N) supersedes the favourable contribution of Δ*G*^‡^_0_(O) to Δ*G*^‡^(O), such that Δ*G*^‡^(N) is much lower than Δ*G*^‡^(O) for alkylations of **1** and **2**. That is, the intrinsic favourability of *O*-alkylation is outweighed by the thermodynamic favourability of *N*-alkylation, so in these irreversible reactions, *N*-alkylation is kinetically preferred.^82^

In the reaction of pyrimidine *N*-oxide (**3**) with MeOTf, the value of Δ_r_*G*°(N) is much less favourable with respect to Δ_r_*G*°(O) than is the case for the corresponding reaction of pyrazine *N*-oxide (**1**). Δ*G*^‡^_0_(O) is calculated to be 21 kJ mol^−1^ lower than Δ*G*^‡^_0_(N) for both MeOTf and MeI (compare Table 4 entry (vii) with entry (v), and entry (viii) with entry (vi)), so *O*-methylation of **3** is intrinsically preferred. Since the thermodynamic favourability of *N*-methylation of **3** is diminished (relative to the corresponding reactions of **1**), and *O*-methylation is intrinsically favoured, Δ*G*^‡^(O) is lower than Δ*G*^‡^(N), and hence *O*-methylation of **3** is the kinetically dominant reaction. Instances in which *N*-alkylation is likely to have been “deactivated” due to steric interactions, resulting in preferential *O*-alkylation, have been reported previously.^4,22*b*,*c*,*d*,*e*,31^ In this case, it seems likely that the free nitrogen Lewis basic site of **3** is deactivated due to an electronic effect. This Lewis basic site is connected through a network of π-bonds to an *N*-oxide group in a *meta* position relative to it, which may act as an electron withdrawing group, thereby diminishing the Lewis basicity (electron donor capacity) of the free nitrogen atom.

The reaction of **3** with MeI was calculated to be thermodynamically unfavourable (Δ_r_*G*° > 0 for both *O*- and *N*-methylation by MeI), and therefore reversible. This is consistent with our experimental observation that no product was formed in this reaction. However, our calculations do indicate that *O*-methylation (formation of **23a**) is kinetically favoured over *N*-methylation. A similar rationale to that presented above for the reaction of **3** + MeOTf applies in this case – *i.e. O*-methylation is intrinsically preferred (Δ*G*^‡^_0_(O) < Δ*G*^‡^_0_(N)) and the thermodynamic advantage of *N*-methylation over *O*-methylation is small, and consequently *O*-methylation is the kinetically favoured process (see Table 4 entries (vi) and (viii)).

As discussed above, the Δ_r_*G*° values calculated for *N*- and *O*-methylations of **3** by both MeI and MeOTf are much less favourable than the Δ_r_*G*° values of methylation reactions of other, similar compounds (*e.g.***1**, **7**, **8** and **27**; *vide supra*). In the context of our analysis based on the Marcus equation, we can make use of this information to rationalise the relatively high Δ*G*^‡^(O) and Δ*G*^‡^(N) values calculated for the methylation reactions of **3**. The less favourable Δ_r_*G*° values for *O*- and *N*-methylations of **3** influence the magnitudes of the Δ*G*^‡^ values for these reactions, causing them to be higher than the Δ*G*^‡^ values of reactions of similar nucleophiles.

As is described in detail in the ESI,†^73^ operationally, the value of the intrinsic barrier (Δ*G*^‡^_0_) for a reaction is accessed as the average of two identity reactions. Since there is no leaving group formed in the addition of a nucleophile to carbenium ions such as **11** and **12** (structures in Scheme 4 above), only one identity reaction of the required two can be identified to model such processes using Marcus theory. Hence, the straightforward method described in the ESI†^73^ for accessing values of intrinsic barriers cannot be employed for reactions involving carbenium ions. Alternative methods for estimating the magnitudes of the intrinsic barriers for such reactions or analogues thereof have been reported,^83^ but these do not allow quantitative determinations of the type performed above for reactions involving electrophiles from which leaving groups become cleaved. Hence only a qualitative appraisal of the outcomes of the reactions of **1** and **2** with benzhydrylium ions is possible, which we give below.

We consider that the observation of strongly preferred or exclusive *N*-benzhydrylation of nucleophiles pyrazine *N*-oxide (**1**) and quinoxaline *N*-oxide (**2**) in their reactions with benzhydrylium ions (**11** or **12**) arises as a consequence of the same factors that dictate the outcomes of the reactions of these nucleophiles with MeI or MeOTf. That is, in each case, *O*-benzhydrylation is intrinsically favoured (Δ*G*^‡^_0_(O) is smaller than Δ*G*^‡^_0_(N)) but the influence of Δ_r_*G*°(N) on Δ*G*^‡^(N) outweighs the influence of Δ*G*^‡^_0_(O) on Δ*G*^‡^(O), and consequently *N*-benzhydrylation is the kinetically preferred process. As discussed above, it was not possible to determine what occurred in the reaction of **3** + benzhydrylium ion **11**, so further comment on this is not warranted.

### Literature examples of *N vs. O* alkylation

We have noted in passing above that, due to the ambiguity that has up until now been inherent in determining which product is formed predominantly in reactions of ambident nucleophiles containing N and O nucleophilic sites, there exist notable cases in the literature in which the products of such reactions may have been misidentified.^8,9,84^

**Scheme 8:**
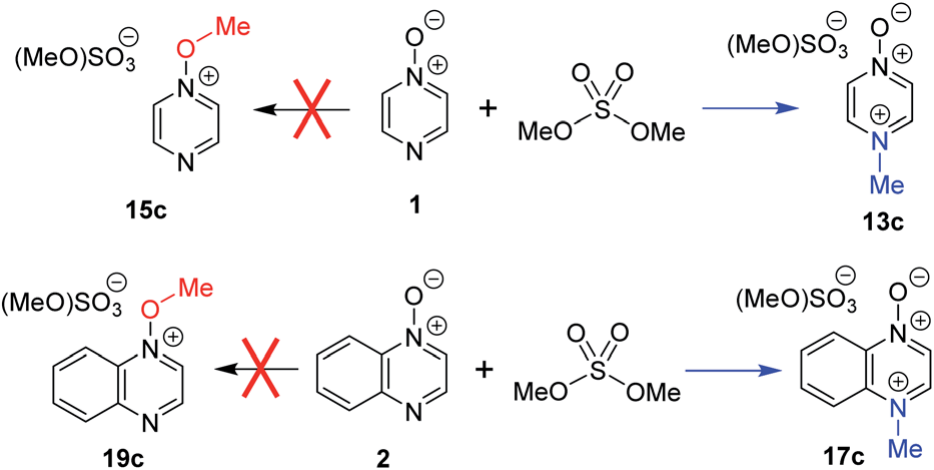
Reactions of compounds **1** and **2** with dimethylsulfate have been reported to give *O*-methylated products **15c** and **19c**.^7^ Our data indicate that *N*-methylated adducts **13c** and **17c** are likely to be the major products.

Comparison of the ^1^H NMR spectrum of *N*-methylated product **13b** (from reactions of MeOTf with **1**; Scheme 4a) with the ^1^H NMR spectra assigned to *O*-methylation adduct **15c** (Scheme 8) in ref. 7 shows that the spectra are essentially identical. A similar observation can also be made on comparison of the ^1^H NMR spectrum of *N*-methylated product **17b** (from **2** + MeOTf; Scheme 4b) and that assigned to *O*-methylated adduct **19c** in ref. 7. We have identified a distinct set of signals belonging to the *O*-methylated adducts **15b** and **19b** that appear at different chemical shifts to the *N*-methylated adducts **13b** and **17b** (*vide supra*). Furthermore, the ^13^C NMR chemical shifts reported for the methyl group carbons (either N–*C*H_3_ or O–*C*H_3_) of the products are 47.2 and 44.5 ppm, respectively.^7^ These *δ*_C_ values are indicative of formation of *N*-methylation products **13c** and **17c** (*vide supra*). Hence, our data indicate that it is highly unlikely that **1** and **2** undergo preferential *O*-methylation in reactions with dimethylsulfate, a close analogue of MeOTf. The methodology reported in ref. 7 was predicated on the use of *N*-methoxypyridinium salts. That this otherwise highly successful methodology did not work for these compounds can be explained by the fact that *N*-methylated compounds **13c** and **17c** were almost certainly employed rather than the intended *O*-methylated compounds **15c** and **19c**. Problems of this type are illustrative of the need for a much more rigorous understanding of the factors that dictate the outcomes in reactions of ambident nucleophiles such as diazine *N*-oxides.

## Conclusions

If one must verify on a case-by-case basis whether the predictive capabilities of a theory apply or not, then those predictive capabilities must be seriously called into question. For this reason, the continued use of the HSAB principle in rationalising the selectivities of ambident reactants in research articles and undergraduate courses and textbooks should be ceased. It appears to us that the approach of Mayr and co-workers, based around Marcus theory, is able to account for the behaviour of ambident reactants in a manner in which the HSAB principle cannot. We hope through this study to have contributed to a more general understanding of ambident reactivity, to have developed upon the approach of Mayr and co-workers to show that it can be applied to semi-quantitatively rationalise product ratios in reactions of ambident nucleophiles, and to have demonstrated the utility of ^1^H–^15^N HMBC NMR spectroscopy in establishing the site of attachment in reactions of nitrogen-containing compounds.

In the cases we have investigated here, calculation of Δ*G*^‡^ values using the equations of Marcus or Zhu yields values that reproduce closely the experimental *N*/*O* methylation ratios for reactions of ambident nucleophiles pyrazine *N*-oxide (**1**) and pyrimidine *N*-oxide (**3**). Based on this, it is reasonable to expect that calculations based on Marcus theory will allow semi-quantitative predictions of the nucleophilic site-selectivities in reactions of other ambident nucleophiles – not just those involving competition between N and O nucleophilic sites. The close agreement between the reaction selectivities determined experimentally and those calculated using the Marcus and Zhu equations (see Table 4 and associated discussion) is demonstrative of the utility of the concept of the intrinsic barrier.

The intrinsic barrier (Δ*G*^‡^_0_) associated with an alkylation reaction of a nucleophile can be considered a property of the compounds involved in the reaction. The interplay between this quantity and the thermodynamic favourability of the reaction (quantified through Δ_r_*G*°) dictates the magnitude of the activation barrier for the reaction (Δ*G*^‡^). Having established herein a computational method that stands up to the stern test posed by modelling of the disparate behaviour of diazine *N*-oxides **1** and **3**, we intend in future publications to determine the magnitudes of intrinsic barriers for reactions of a wide variety of other nucleophiles, and hence establish systematic trends in intrinsic barriers (developing upon the work of Hoz).^75^ This will allow the factors that control intrinsic barriers to be understood, and hence deepen our understanding of activation barriers in general.

## Details on computational methodology

The conformational space for each structure was explored with the OPLS-2005 force field^85^ and a modified Monte Carlo search algorithm implemented in Macromodel.^86^ An energy cut-off of 84 kJ mol^−1^ was employed for the conformational analysis, and structures with heavy-atom root-mean-square deviations (RMSD) up to 0.5 Å after the force field optimizations where considered to be the same conformer. All remaining structures were subsequently optimized with the dispersion-corrected M06-2X functional^87^ with Grimme's dispersion correction D3 (zero-damping),^88^ the triple-ζ basis set 6-311+G(d,p), and SMD solvation model^89^ for acetonitrile. An ultrafine grid was used throughout this study for the numerical integration of the density. Vibrational analysis verified that each structure was a minimum or a transition state and for the latter, following the intrinsic reaction coordinates (IRC) confirmed that all transition states connected the corresponding reactants and products on the potential energy surface. Thermal corrections were obtained from unscaled harmonic vibrational frequencies at the same level of theory for a standard state of 1 mol L^−1^ and 298.15 K. Entropic contributions to free energies were obtained from partition functions evaluated with Grimme's quasi-harmonic approximation.^90^ This method employs the free-rotor approximation for all frequencies below 100 cm^−1^, the rigid-rotor-harmonic-oscillator (RRHO) approximation for all frequencies above 100 cm^−1^, and a damping function to interpolate between the two expressions. Similar results were obtained from partition functions evaluated with Cramer's and Truhlar's quasiharmonic approximation.^91^ This method uses the same approximations as the usual harmonic oscillator approximation, except that all vibrational frequencies lower than 100 cm^−1^ are set equal to 100 cm^−1^. Electronic energies were subsequently obtained from single point calculations of the M06-2X-D3 geometries employing Neese's domain-based local pair-natural orbital (DLPNO) approach to the CCSD(T) method [DLPNO-CCSD(T)] with the default normalPNO settings,^92–94^ the triple-ζ def2-TZVPPD^95,96^ in combination with the corresponding auxiliary basis set^97^ and the SMD continuum model for acetonitrile.^89^ All density functional theory calculations were performed with Gaussian 16,^98^ while the DLPNO-CCSD(T) calculations were performed with ORCA 4.^99^

## Conflicts of interest

There are no conflicts to declare.

## Supplementary Material

SC-011-D0SC02834G-s001

SC-011-D0SC02834G-s002

SC-011-D0SC02834G-s003

SC-011-D0SC02834G-s004

SC-011-D0SC02834G-s005

SC-011-D0SC02834G-s006

SC-011-D0SC02834G-s007

SC-011-D0SC02834G-s008

SC-011-D0SC02834G-s009

SC-011-D0SC02834G-s010

SC-011-D0SC02834G-s011

SC-011-D0SC02834G-s012

SC-011-D0SC02834G-s013

SC-011-D0SC02834G-s014

SC-011-D0SC02834G-s015

SC-011-D0SC02834G-s016

SC-011-D0SC02834G-s017

SC-011-D0SC02834G-s018

SC-011-D0SC02834G-s019

SC-011-D0SC02834G-s020

SC-011-D0SC02834G-s021

SC-011-D0SC02834G-s022

SC-011-D0SC02834G-s023

SC-011-D0SC02834G-s024

SC-011-D0SC02834G-s025

SC-011-D0SC02834G-s026

SC-011-D0SC02834G-s027

SC-011-D0SC02834G-s028

SC-011-D0SC02834G-s029

SC-011-D0SC02834G-s030

SC-011-D0SC02834G-s031

SC-011-D0SC02834G-s032

SC-011-D0SC02834G-s033

SC-011-D0SC02834G-s034

SC-011-D0SC02834G-s035

SC-011-D0SC02834G-s036

SC-011-D0SC02834G-s037

SC-011-D0SC02834G-s038

SC-011-D0SC02834G-s039

SC-011-D0SC02834G-s040

SC-011-D0SC02834G-s041

SC-011-D0SC02834G-s042

SC-011-D0SC02834G-s043

SC-011-D0SC02834G-s044

SC-011-D0SC02834G-s045

SC-011-D0SC02834G-s046

SC-011-D0SC02834G-s047

SC-011-D0SC02834G-s048

SC-011-D0SC02834G-s049

SC-011-D0SC02834G-s050

SC-011-D0SC02834G-s051

SC-011-D0SC02834G-s052

SC-011-D0SC02834G-s053

SC-011-D0SC02834G-s054

SC-011-D0SC02834G-s055

SC-011-D0SC02834G-s056

SC-011-D0SC02834G-s057

SC-011-D0SC02834G-s058

SC-011-D0SC02834G-s059

SC-011-D0SC02834G-s060

SC-011-D0SC02834G-s061

SC-011-D0SC02834G-s062

SC-011-D0SC02834G-s063

SC-011-D0SC02834G-s064
