# Supplementary material for: Competition between N and O: use of diazine N-oxides as a test case for the Marcus theory rationale for ambident reactivity
Source: Chem Sci. 2020 Jul 23;11(35):9630–47. doi: 10.1039/d0sc02834g (PMC8162281; doi:10.1039/d0sc02834g)
Supplement: SC-011-D0SC02834G-s001 [file SC-011-D0SC02834G-s001.pdf]

Electronic Supplementary Material (ESI) for Chemical Science.
This journal is © The Royal Society of Chemistry 2020

Competition Between N and O: Use of Diazine *N*-Oxides as a Test Case for the Marcus Theory Rationale for Ambident Reactivity

Kevin J. Sheehy,[a] Lorraine M. Bateman,[a,b,d] Niko T. Flosbach,[c] Martin Breugst,*[c] Peter A. Byrne,*[a,d]

[a] School of Chemistry, Analytical and Biological Chemistry Research Facility, University College Cork, College Road, Cork, Ireland.

[b] School of Pharmacy, University College Cork, College Road, Ireland

[c] Department für Chemie, Universität zu Köln, Greinstraße 4, 50939 Köln, Germany.

[d] SSPC (Synthesis and Solid State Pharmaceutical Centre), Cork, Ireland.

E-mail: peter.byrne@ucc.ie

mbreugst@uni-koeln.de

Contents

1. General Experimental ………………………………………………………….…….. S3 – S4

2. Preparation and ^{1}H-^{15}N HMBC NMR spectra of diazine *N*-oxides **1**–**3**……………… S4 – S6

3. Synthesis of 4-methylbenzhydryl chloride…….………..…………………………... S7

4. Reactions of Diazines and *N*-oxides with MeI, MeOTf and benzhydrylium ions......... S8 – S38
   - 4.1 General Procedures ……………………………………………………………... S8
   - 4.2 Reactions of Pyrazine *N*-Oxide (**1**)……………………….....………………… S9 – S17
   - 4.3 Reactions of Quinoxaline *N*-Oxide (**2**)…………..………......………………… S18 – S27
   - 4.4 Reactions of Pyrimidine *N*-Oxide (**3**)………………...………....................... S28 – S37

5. Crossover experiments of **1**, **2**, **3** and **25** with a competing nucleophile………....... S38 – S52
   - 5.1 Crossover experiment of pyrazine *N*-oxide (**1**) with MeOTf and methyl nicotinate (**25**) …………………………………………………………….. S38 – S41
   - 5.2 Crossover experiment of quinoxaline *N*-oxide (**2**) with MeOTf and pyrazine (**7**) S42 – S45
   - 5.3 Crossover experiment of pyrimidine *N*-oxide (**3**) with MeOTf and pyrazine (**7**)… S46 – S50
   - 5.4 Crossover experiment of 4-Methylpyrazinium-*N*-oxide iodide (**13a**) with MeOTf and methyl nicotinate (**25**) ……... S50 – S52

6. Competition experiment: Pyrazine *N*-oxide (**1**) vs Pyrimidine *N*-oxide (**3**)………… S53 – S54

7. Full Spectra for compounds produced in Sections 4 – 6 …………..………………… S55 – S82

8. Calculations of Thermodynamic and Activation Parameter Values …………...……… S83 – S85

9. Calculation of Marcus Intrinsic Barriers …...……..…………………………...…….. S86 – S91

10. Charge Density Calculations ……………………………………………..……….. S92

11. Calculation of Activation Barriers for Methyl Transfer Identity Reactions……....…. S93 – S99

12. Calculations on Reactions with Methyl Iodide and Methyl Triflate ………..………….. S100 – S110

13. Determination of 2nd Order Rate Constant……………..……..…………………...….. S111 – S116

14. Additional Literature References from Main Article……………..………………...….. S117

15. Supporting Information References…………………………………........................ S118

1. General Experimental

Commercial diazines and alkylating agents were obtained from Fluorochem, Sigma-Aldrich and Alfa Aesar.

CH_2Cl_2, CH_3CN, CD_3CN, $(CD_3)_2SO$ and $(CH_3)_2SO$ were dried over activated 3 Å molecular sieves and stored under an atmosphere of nitrogen in flasks with grease-free J. Young's valves (this is a modification of the method of Williams and Lawton).[1] Molecular sieves (10 weight percent per unit volume of compound to be dried) were activated by flame drying in the storage flask(s) for 5 – 10 minutes (depending on quantity of sieves to be dried). After flame-drying, the storage flask was immediately connected to a Schlenk line, subjected to vacuum (between 2 and 5×10^{-3} mbar), and allowed to stand until the sieves had cooled. The flask was then subjected to several vacuum/refill cycles to establish a nitrogen atmosphere inside, and the solvent/compound to be dried was then added against a flow of nitrogen.

Solvents that were used in relative bulk (CH_3CN, CH_2Cl_2) were stored in a specialised flask with two J. Young's valves, one of which was modified to facilitate easy access of a needle to the body of the flask through the side-arm of the valve. When accessing the dry solvent, the angled side-arm was sealed with a rubber septum, and the small volume contained between the septum and the sealed tap of the J. Young's valve was flushed with a stream of nitrogen gas for a minimum of five minutes prior to opening the valve. The solvent required several days after commencing drying to reach maximal dryness (according to analysis by Karl Fischer titration), but was dry enough for most purposes after one day. CH_3CN and THF stored in this manner was found to retain water contents of less than 10 ppm for more than one year.

For all reactions conducted using Schlenk glassware, the Schlenk flask was dried in an oven, then attached to vacuum via Schlenk manifold and placed under vacuum ($\leq 5 \times 10^{-3}$ mbar). The flask was then filled with nitrogen gas by the pump and fill technique (three repeats of the following cycle: evacuation to $\leq 5 \times 10^{-3}$ mbar, re-fill with nitrogen gas).[2] Solids and reagents were then introduced to the flasks under fast nitrogen flow.

NMR spectra were recorded on Bruker Avance III 600, Bruker Avance III 500, Bruker Avance I 400 and Bruker Avance III 300 NMR spectrometers. ^{1}H and ^{13}C NMR chemical shifts were referenced to tetramethylsilane (TMS). ^{1}H NMR spectra (proton coupled mode, 600 MHz, 400 MHz and 300 MHz respectively) ^{13}C{^{1}H} NMR spectra (proton decoupled mode; 150 MHz,100 MHz and 75 MHz, respectively), HSQC NMR spectra, HMBC NMR spectra and COSY NMR spectra were acquired at 300 K on the 300 and 600 MHz instruments and 293 K on the 400 MHz instrument. ^{1}H NMR spectra on the 500 MHz instrument (equipped with a 5 mm QNP probe) were recorded at 298 K. ^{1}H NMR spectra were acquired using a 30° pulse (Bruker zg pulse programme), an acquisition time of 2.65 seconds, and a time domain data size of 32768 or 65536 points. A relaxation delay of 5 seconds was used in most instances; exceptions to this are noted where applicable below. Signal assignments in the ^{1}H and ^{13}C NMR spectra were made with reference to information contained in the two-dimensional NMR spectra. ^{1}H-^{15}N HMBC spectra were recorded at 300 K on a Bruker Avance III 600 NMR spectrometer [600 MHz (^{1}H), 60.8 MHz (^{15}N)], equipped with Bruker BBFO cryoprobe (coil temperature 16 K) and referenced externally to ammonia, the value of which was uncorrected. ^{1}H-^{15}N HMBC spectra were acquired using the Bruker

hmbcqpndqf pulse program (2D H-1/X correlation via heteronuclear zero and double quantum coherence optimised on long range couplings), with 4 scans and spectral width of 600–650 ppm. All ^{1}H-^{15}N HMBC NMR spectra shown below were processed (post-acquisition) by application of t1 noise reduction. All spectra were run at University College Cork. Spectra recorded in non-deuterated solvents were acquired using the Bruker NOESY presat (noesygppr) solvent suppression pulse sequence, using presaturation during the mixing time and relaxation delay. Chemical shifts (δ) are expressed as parts per million (ppm), positive shift being downfield from TMS; coupling constants (*J*) are expressed in Hertz (Hz). Splitting patterns in ^{1}H-NMR spectra are designated as: s (singlet), bs (broad singlet), d (doublet), dd (doublet of doublets), ddd (doublet of doublets of doublets), t (triplet), td (triplet of doublets), q (quartet), quin (quintet) and m (multiplet). Infrared spectra were measured using a FTIR UATR2 spectrometer as thin films in acetonitrile. Data are represented as follows: frequency of absorption (cm^{-1}), intensity of absorption (s = strong, m = medium, w = weak, br = broad). High resolution (precise) mass spectra (HRMS) were recorded on a Waters LCT Premier TOF LC–MS instrument using electrospray ionization in positive ionization mode (ESI+) using 50 % acetonitrile/water containing 0.1 % formic acid as eluent. Samples were made up at a concentration of approximately 1 mg ml^{-1}.

2. Preparation and ^{1}H-^{15}N HMBC NMR spectra of diazine *N*-oxides 1 – 3

Preparations of diazine *N*-oxides were achieved with modifications of established literature procedures.[3,4]

We recommend the use of a slight excess of diazine (relative to the amount of 3-chloroperbenzoic acid) in order to remove the need to use quenching agents (e.g. Ph_3P, Na_2SO_3) in these reactions.

(i) Pyrazine *N*-oxide (1) [4]

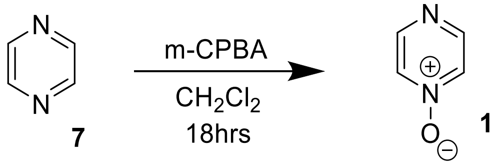

Pyrazine (**7**) (1.12 g, 14.0 mmol) was dissolved in CH_2Cl_2 (70 ml). 3-Chloroperbenzoic acid (3.08 g, 13.8 mmol) was added in one portion, and the solution was stirred for 18 hrs, turning a cloudy white colour (due to precipitated 3-chlorobenzoic acid). The reaction mixture was washed twice with saturated sodium sulfite solution (*ca.* 40 ml each) and once with a solution of brine (*ca.* 40 ml). The recovered organic phase was dried over Na_2SO_4, and the drying agent was removed by filtration. The solvent was then removed under reduced pressure. The residue was purified by column chromatography using 100% EtOAc, yielding a colourless, needle-like solid. (0.56 g, 5.8 mmol, 42%). This material was immediately transferred to a glove box upon isolation.

^{1}H NMR (300 MHz, $CDCl_3$) δ 8.52 – 8.44 (m, 2H), 8.15 – 8.08 (m, 2H). [5]

A further sample of **1** (0.080 g) was dissolved in CH_2Cl_2 (0.65 ml) and placed in an NMR tube by syringe under nitrogen, which was then sealed by a rubber septum cap and wrapped with PTFE tape. ^{1}H NMR and ^{1}H-^{15}N HMBC NMR spectra were recorded on this sample. The ^{15}N NMR chemical shift values reported below were attained from the ^{1}H-^{15}N HMBC NMR experiment. See the General Experimental for details on the solvent suppression protocol used during acquisition.

^{1}H NMR (600 MHz, CH_2Cl_2) δ 8.40 (app d, app *J* = 4.3 Hz, 2H), 8.08 – 8.03 (m, 2H).

^{15}N NMR (60.8 MHz, CH_2Cl_2): δ 311, 303.5

(ii) Quinoxaline *N*-oxide (2)

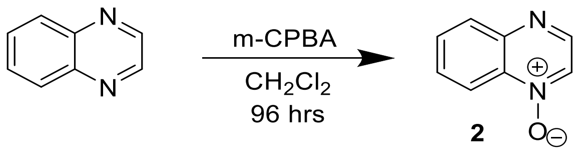

Quinoxaline (1.70 g, 13.1 mmol) was dissolved in 100 ml CH_2Cl_2. 3-Chloroperbenzoic acid (2.39 g, 13.8 mmol) was added in one portion, and the solution was stirred for 4 days. Precipitated 3-chlorobenzoic acid appeared in the reaction mixture after a few hours. The reaction mixture was washed twice with saturated sodium sulfite solution (*ca.* 40 ml each) and once with a solution of brine (*ca.* 40 ml). The recovered organic phase was dried over Na_2SO_4, and the drying agent was removed by filtration. The solvent was then removed under reduced pressure. The residue was purified by column chromatography in silica using 70:30 ethyl acetate/cyclohexane, yielding light tan-coloured solid (**2**). (1.27 g, 8.68 mmol, 66% yield). This material was immediately transferred to a glove box upon isolation.

^{1}H NMR (300 MHz, $CDCl_3$) δ 8.68 (d, *J* = 3.6 Hz, 1H), 8.59 (dd, *J* = 8.6, 1.4 Hz, 1H), 8.35 (d, *J* = 3.6 Hz, 1H), 8.19 – 8.11 (m, 1H), 7.88 – 7.72 (m, 2H). [6]

A sample of the product (0.055 g) was dissolved in CH_2Cl_2 (0.65 ml) and placed in an NMR tube by syringe under nitrogen, which was then sealed by a rubber septum cap and wrapped with PTFE tape. ^{1}H NMR and ^{1}H-^{15}N HMBC NMR spectra were recorded on this sample. The ^{15}N NMR chemical shift values reported below were attained from the ^{1}H-^{15}N HMBC NMR experiment. See the General Experimental for details on the solvent suppression protocol used during acquisition.

^{1}H NMR (600 MHz, CH_2Cl_2) δ 8.62 (d, *J* = 3.5 Hz, 1H), 8.50 (app d, app *J* = 8.6 Hz, 1H), 8.31 (d, *J* = 3.5 Hz, 1H), 8.09 (app d, app *J* = 8.4 Hz, 1H), 7.82 – 7.77 (m, 1H), 7.74 – 7.69 (m, 1H).

^{15}N NMR (60.8 MHz, CH_2Cl_2): δ 302, 300.3.

(iii) Preparation of Pyrimidine *N*-oxide

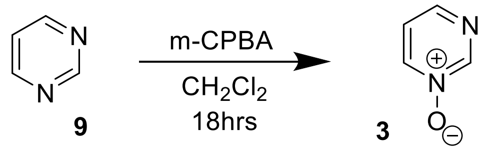

Pyrimidine (**9**) (1.74 g, 21.7 mmol) was dissolved in CH_2Cl_2 (110 ml). 3-Chloroperbenzoic acid (5.62 g, 32.6 mmol) was added in one portion, and the solution was stirred for 48 hrs, turning a cloudy white colour (due to precipitated 3-chlorobenzoic acid). PPh_3 (3.90 g, 14.9 mmol) was added, and the solution was stirred for 3 hours. The solvent was removed under reduced pressure. The residue was purified by column chromatography using 90 : 10 EtOAc/Cyclohexane, yielding a white crystalline solid (**3**). (0.993 g, 10.3 mmol, 48 %). The product is very hygroscopic and hence was transferred to a glove box immediately after isolation.

^{1}H NMR (300 MHz, $CDCl_3$) δ 9.01 (m (fine splitting not resolved), 1H), 8.42 – 8.35 (m, 1H), 8.25 (dd, *J* = 4.7, 1.4 Hz, 1H), 7.35 – 7.28 (m, 1H). [7]

Authors' Note: We recommend that PPh_3 should NOT be used for quenching purposes, as it was difficult to find chromatographic conditions allowing the product to be separated from triphenylphosphine oxide, and significant loss of product occurred due to co-elution with Ph_3PO.

A sample of the product (0.047 g) was dissolved in DMSO (0.65 ml) and placed in an NMR tube by syringe under nitrogen, which was then sealed by a rubber septum cap and wrapped with PTFE tape. ^{1}H NMR and ^{1}H-^{15}N HMBC NMR spectra were recorded on this sample. The ^{15}N NMR chemical shift values reported below were attained from the ^{1}H-^{15}N HMBC NMR experiment. See the General Experimental for details on the solvent suppression protocol used during acquisition.

^{1}H NMR (600 MHz, DMSO) δ 9.04 (s, 1H), 8.58–8.52 (m, 1H), 8.25 (dd, J = 4.7, 1.0 Hz, 1H), 7.55 – 7.49 (m, 1H).

^{15}N NMR (60.8 MHz, DMSO): δ 301.3, 291.7.

A further sample of **3** (0.047 g) was dissolved in CH_2Cl_2 (0.65 ml) and placed in an NMR tube by syringe under nitrogen, which was then sealed by a rubber septum cap and wrapped with PTFE tape. The product was analysed by ^{1}H-^{15}N HMBC NMR. See the General Experimental for details on the solvent suppression protocol used during acquisition.

^{1}H NMR (600 MHz, CH_2Cl_2) δ 8.90 (s, 1H), 8.32 (app d, app *J* = 6.6 Hz, 1H), 8.16 (app d, app *J* = 4.6 Hz, 1H), 7.30 – 7.24 (m, 1H).

^{15}N NMR (60.8 MHz, CH_2Cl_2): δ 299.6, 291.4.

3. Synthesis of 4-methylbenzhydryl chloride

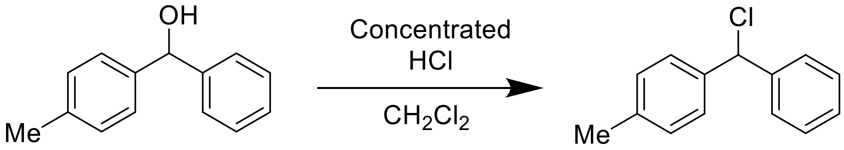

4-methylbenzhydrol (1.00 g, 5.04 mmol) was dissolved in dichloromethane (16 ml), and the resulting solution was cooled in an ice bath for 10 minutes. Over approximately 20 minutes, concentrated aqueous HCl (37%; 5 ml) was added dropwise from a Pasteur pipette into the solution of 4-methylbenzhydrol at 0 °C. The reaction was stirred at 0 °C for 1.5 hours, and then placed in a refrigerator overnight. The reaction was then transferred into a pre-chilled separating funnel (cooled in freezer in advance), and the dichloromethane phase was separated from the aqueous phase. The aqueous phase was extracted twice with cold dichloromethane (pre-chilled in an ice bath; *ca.* 5 ml per extraction), and the dichloromethane phases were combined and then dried over anhydrous $CaCl_2$. The $CaCl_2$ was removed by filtration. The dichloromethane phases were kept cold at all points by immersing the vessel(s) containing them in an ice bath.

Next, the solvent was removed from the filtrate under vacuum, giving a colourless oil (1.05 g, 4.85 mmol, 96%). The flask containing the product was maintained at room temperature during solvent removal, and a relatively high vacuum was used to remove the solvent as quickly as possible. A sample was removed and dissolved in $CDCl_3$, and a ^{1}H NMR spectrum was obtained.

^{1}H NMR (300 MHz, $CDCl_3$) δ 7.46 – 7.26 (m, overlaps with C*H*Cl_3 signal, contains 7H of tolyl and phenyl groups), 7.15 (app d, app *J* = 7.9 Hz, 2H), 6.11 (s, 1H, Ar_2C*H*), 2.34 (s, 3H, CH_3). [8]

An attempt was made to crystallise the product by triturating with *n*-pentane, and hence small signals of this solvent are present in the ^{1}H NMR spectrum recorded of the product.

The product was stored in a freezer, and remains stable at −18 °C for at least one year.

4. Reactions of Diazine *N*-oxides with MeI, MeOTf and benzhydrylium ions

4.1 General Procedures

General Procedure A: Removal of solvent without compromising inert atmosphere

The following procedure was used to remove the solvent (MeCN, CD_3CN or Et_2O) and volatile reagents (MeI or MeOTf) from a Schlenk flask containing a completed reaction mixture without exposing the product(s) to the ambient atmosphere, allowing the inert atmosphere in a reaction flask to be re-established after completion of removal of volatile materials. A second vacuum trap was attached to the Schlenk manifold on one arm and to the sealed reaction flask by the other. An inert atmosphere was established in the second trap and connective tubing by three pump and re-fill cycles.[2] The trap was then again placed under vacuum ($\leq 5 \times 10^{-3}$ mbar) and then immersed in liquid N_2 in a Dewar flask. At this point, the tap on the Schlenk flask is carefully opened and volatile reagents are removed and collected in the second trap. After approximately 30 minutes, the entirety of the trap and the Schlenk flask are re-filled with nitrogen gas through the Schlenk manifold, and the tap of the Schlenk flask is closed. The trap is removed and the Schlenk flask is re-attached directly to the Schlenk manifold.

General Procedure B: Preparation of NMR samples under inert atmosphere

The following procedure was used to place the products of the alkylation reactions (dissolved in an appropriate solvent) into NMR tubes while maintaining an inert atmosphere. The products were formed in an N_2-filled Schlenk flask using inert atmosphere techniques. The appropriate solvent was introduced to the Schlenk flask by syringe and *ca.* 10 mg of the product was dissolved. An empty NMR tube was placed in a long, tube shaped Schlenk flask, which was evacuated and re-filled with nitrogen ≥ 3 times by the pump and refill technique,[2] creating an inert atmosphere inside the flask. The solution to be examined (in DMSO or CH_2Cl_2) was added to the NMR tube by syringe under nitrogen. The NMR tube was then sealed by a rubber septum cap. The seal made by the rubber septum on the outside of the NMR tube was secured by wrapping it with PTFE tape and then a layer of Parafilm. The sealed NMR tube was then transferred to the appropriate spectrometer for analysis.

General Procedure C: Preparation of benzhydryl adducts of heterocycles and *N*-oxides

The appropriate benzhydryl chloride (1 equivalent) was weighed into a reaction vessel and transferred into a glove box containing a nitrogen atmosphere. Dry CD_2Cl_2,CH_2Cl_2 or CD_3CN (usually 0.85 ml) was added, followed by the heterocycle or *N*-oxide (1 equivalent). AgOTf (1.1 – 1.2 equivalents) was then added, causing the immediate precipitation of AgCl. The reaction vessel was sealed, and agitated (15 minutes for 4-methylbenzhydryl chloride, 60 minutes for benzhydryl chloride), and then filtered (removing AgCl) through a syringe filter into an NMR tube. The NMR tube was sealed using a rubber septum. The seal was then wrapped with PTFE tape and Parafilm. Finally, the NMR tube was placed in a long Schlenk flask and removed from the glove box and brought to the NMR spectrometer. All products underwent relatively rapid decomposition (hydrolysis) on exposure to moisture, and hence were only characterized by inert atmosphere NMR spectroscopy.

4.2 Reactions of Pyrazine *N*-Oxide (1)

Preparation of *N*-methylpyrazinium *N'*-oxide iodide (13a)

(a) Experiment Showing Isolated Yield of 13a (Solvent-Free Reaction) – Contains ^{15}N NMR data

Pyrazine *N*-oxide (**1**) (0.041 g, 0.43 mmol) was placed in a N_2-filled Schlenk flask. Methyl iodide (0.53 ml, 1.2 g, 8.5 mmol) was added by syringe to the flask. The flask was wrapped in foil and left in the dark for 48 hours, after which time the methyl iodide was removed under vacuum using General Procedure A. The resulting yellow solid (**13a**) was washed by addition of dry Et_2O, which was removed by cannula filtration (under inert atmosphere). Three aliquots of dry Et_2O (0.5 ml each) were used in this manner to wash the product, (yield = 0.026 g, 0.11 mmol, 26%) A sample of **13a** in dry $(CD_3)_2SO$ was then prepared for ^{1}H and ^{1}H-^{15}N HMBC NMR spectroscopic characterization by Procedure B

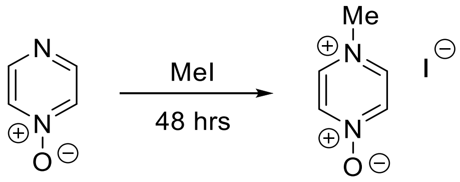

1 **13a**

^{1}H NMR (600 MHz, $(CD_3)_2SO$) δ 9.05 – 9.01 (m, 2H), 9.00 – 8.97 (m, 2H), 4.18 (s, 3H, C*H*$_3$). [9]

^{15}N NMR (60.8 MHz, $(CD_3)_2SO$): δ 322.3 (*N*—O), 187.1 (*N*$^+$—Me).

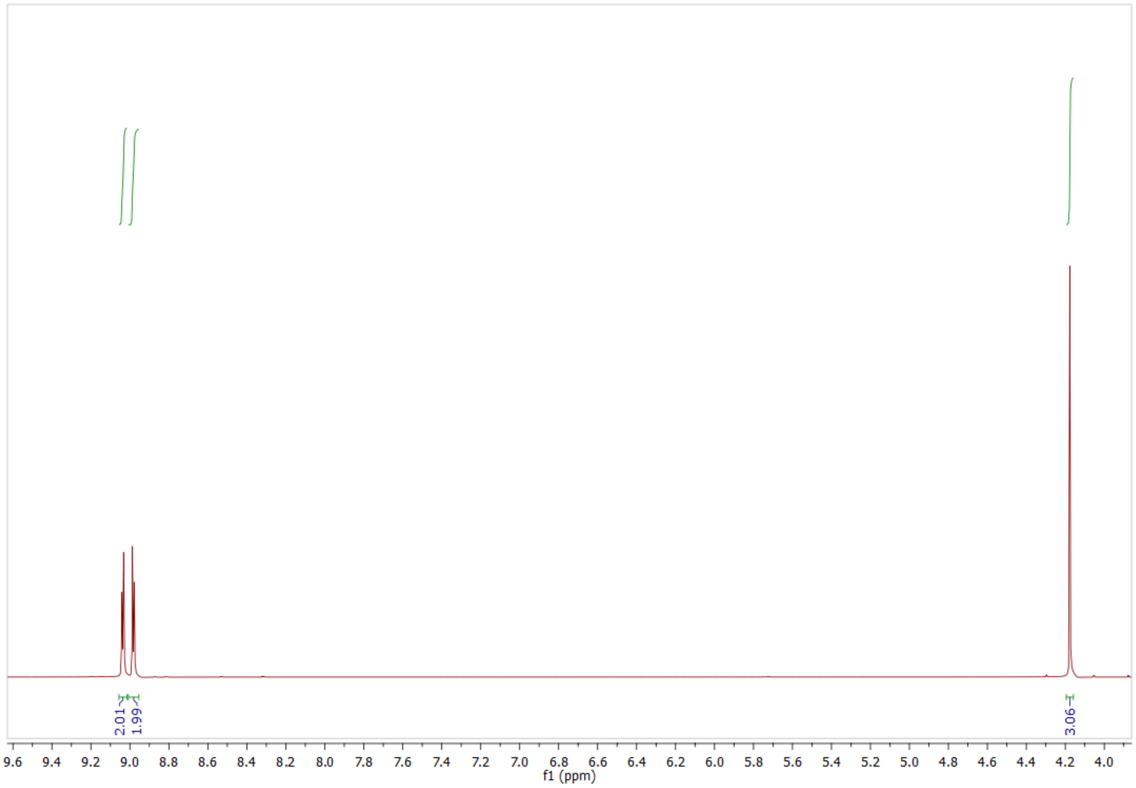

Figure S1: ^{1}H NMR spectrum in $(CD_3)_2SO$ of **13a**, showing no **15a**. The full spectrum is shown in Section 7.

(b) Experiment in CD_3CN Showing Low Conversion to 13a

In a glove box, pyrazine *N*-oxide (**1**) (0.019 g, 0.20 mmol) was dissolved in CD_3CN (0.65 ml). Methyl iodide (0.033 g, 0.23 mmol) was added dropwise by syringe to the solution of **1**. The reaction vessel was agitated throughout addition of MeI. After completion of addition of MeI, the entire reaction mixture was transferred to an NMR tube. The NMR tube was sealed with a rubber septum, and the seal was secured by wrapping with PTFE tape and then Parafilm. The NMR tube was take to the NMR spectrometer. A ^{1}H NMR spectrum recorded approximately 20 minutes after mixing of the reactants showed no conversion to **13a** (i.e. only signals of **1** and MeI were observed). After four days, a second ^{1}H NMR spectrum was obtained. This showed low conversion to **13a**. No signals of **15a** were observed.

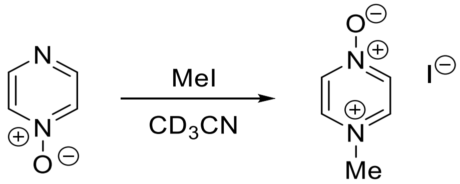

1 **13a**

^{1}H NMR (300 MHz, CD_3CN)

Assigned to **13a**: δ 8.74 – 8.66 (m, 2H), 8.61 – 8.51 (m, 2H), 4.20 (s, 3H, NC*H*$_3$).[9]

Assigned to **1**: δ 8.46 – 8.38 (m, 2H), 8.13 – 8.06 (m, 2H).

Relative to 1H of **13a**, 1H of **1** integrates for 3.1H. Therefore, the conversion to **13a** was 24%. A signal of H_2O is present in the second spectrum since due to ingress of into the NMR tube.

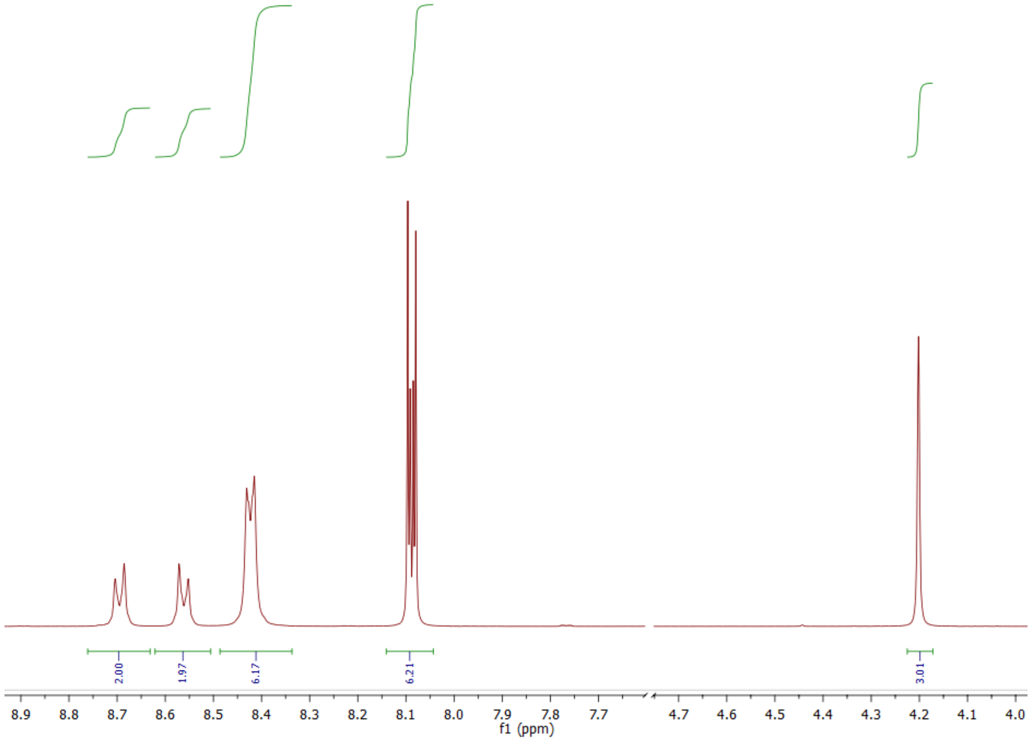

Figure S2: ^{1}H NMR spectrum of reaction of **1** + MeI in CD_3CN, forming **13a** in low conversion after 4 days, and showing that no **15a** is formed. The full spectrum is shown in Section 7.

Preparations of 13b and 15b

(a) Experiment Showing Isolated Yield of 13b

Pyrazine *N*-oxide (**1**) (0.166 g, 1.73 mmol) was dissolved in CH_3CN (5.0 ml) in a N_2-filled Schlenk flask. Methyl triflate (0.318 g, 1.94 mmol) was then added dropwise. After 96 hours, the CH_3CN was removed under vacuum using General Procedure A. The solid product (**13b**) was washed by addition of dry Et_2O, which was removed by cannula filtration (under inert atmosphere). Three aliquots of dry Et_2O (3 ml each) were used in this manner to wash the product (yield = 0.305 g, 1.17 mmol, 68%) A sample of **13b** in dry $(CD_3)_2SO$ was prepared using General Procedure B for ^{1}H NMR spectroscopic characterization.

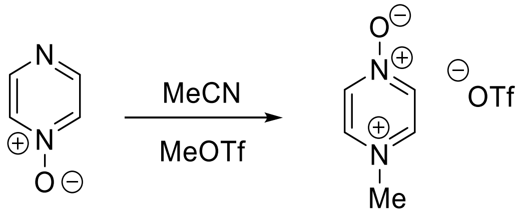

1 **13b**

^{1}H NMR (300 MHz, $(CD_3)_2SO$) δ 9.03 – 8.96 (m, 4H), 4.16 (s, 3H, C*H*$_3$). [9]

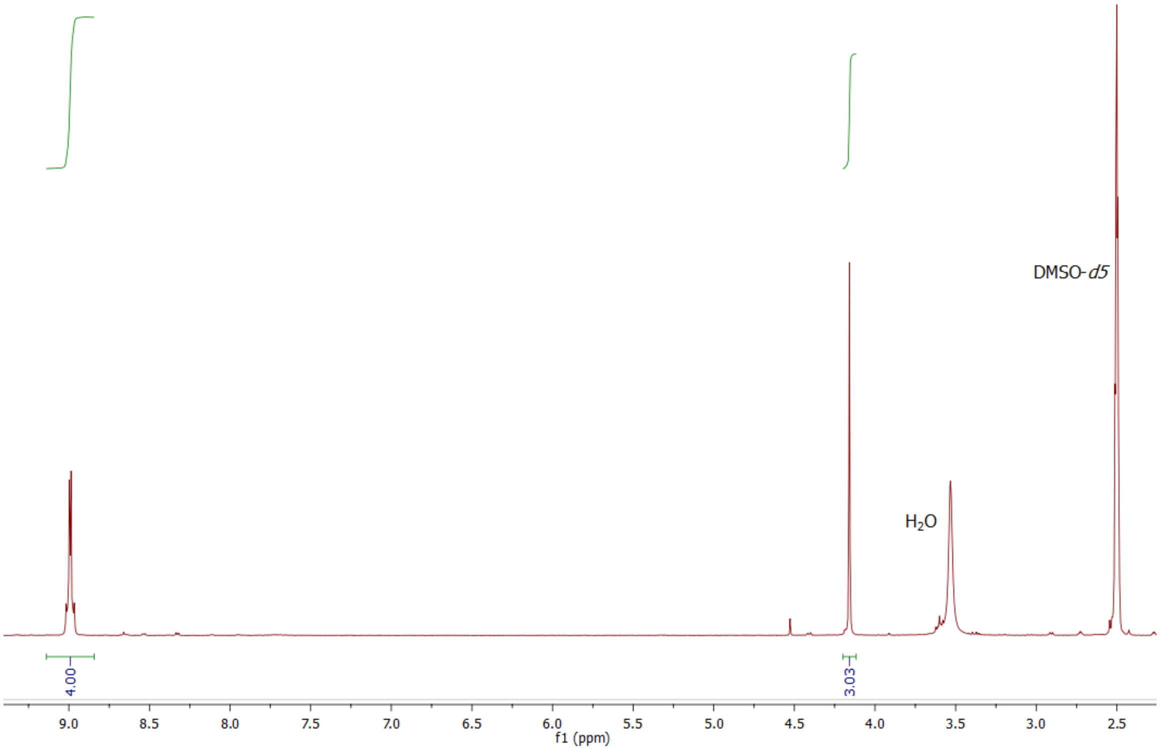

Figure S3: ^{1}H NMR spectrum of **13b** in $(CD_3)_2SO$. The full spectrum is shown in Section 7.

(b) Experiment Showing N- vs O-Alkylation Product Ratio (13b vs 15b) – Contains ^{15}N NMR Data

Pyrazine *N*-oxide (**1**) (0.031 g, 0.32 mmol) was dissolved in CD_3CN (0.65 ml) in a N_2-filled Schlenk flask. Methyl triflate (0.050 g, 0.030 mmol) was subsequently added dropwise. The reaction mixture was transferred to an NMR tube and analyzed by NMR spectroscopy using General Procedure B.

Note: Insufficient concentrations of **1** and **15b** in the spectra below meant that unambiguous assignments of ^{1}H and ^{13}C NMR signals to specific sites in the structures of these compounds was not possible.

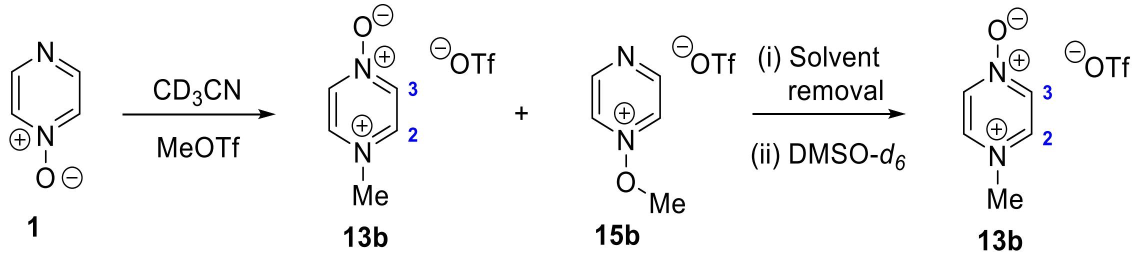

^{1}H NMR (300 MHz, CD_3CN)

Signals assigned to **13b**: δ 8.64 – 8.50 (m, 4H, H-2 and H-3), 4.19 (s, 3H, NCH_3).

Signals assigned to **15b**: δ 9.48 (dd, J = 3.3, 1.6 Hz, 2H), 9.13 (dd, J = 3.3, 1.6 Hz, 2H), 4.54 (s, 3H, OCH_3). Relative to 1H of **13b**, 1H of **15b** integrates for 0.05H.

Signals assigned to the starting material **1**: δ 8.48 (d, J = 4.8 Hz, 1H), 8.16 (dd, J = 3.6, 1.5 Hz, 1H). Relative to 1H of **13b**, 1H of **1** integrates for *ca.* 0.15H.

The signal at δ 8.64 – 8.50 ppm contains 4H of **13b** and 2H of the starting material **1**. The integration of this signal is slightly low with respect to the other signals of **1** and **13b**; this is likely to be due to a slow relaxation rate of one of the contributing protons.

^{13}C{^{1}H} NMR (75 MHz, CD_3CN)

Assigned to **13b**: δ 143.1 (C-2), 139.9 (C-3), 48.1 (NCH_3)

Assigned to **15b**: δ 153.1, 132.2, 69.1 (OCH_3)

Assigned to **1**: δ 148.1, 135.6.

Quantitative product formation can be concluded in this experiment on the basis of complete consumption of MeOTf (no signal of MeOTf present in the ^{1}H NMR spectrum). Ratio of N-alkylation and O-alkylation Products (from ^{1}H NMR spectrum):

4H of Compound **13b** = 4.00 – Therefore 1H = 1.00

2H of compound **15b** = 0.10 – Therefore 1H = 0.05

$$\text{Ratio} = \frac{1.00}{1.00 + 0.05} \times \ 100 = 95\% \text{ N alkylation}$$

The CD_3CN was removed using General Procedure A and the product mixture was re-dissolved in (CD_3)-$_2$SO to record a ^{1}H-^{15}N HMBC NMR spectrum. Product **15b** did not survive the solvent removal process.

^{1}H NMR (600 MHz, $(CD_3)_2SO$)

Signals assigned to **13b**: δ 9.02 – 8.99 (m, 2H), 8.99 – 8.96 (m, 2H), 4.17 (s, 3H, CH_3).

Signals assigned to **1**: δ 8.55 – 8.53 (m, 2H), 8.33 – 8.31 (m, 2H). Relative to 1H of **13b**, 1H of **1** integrates for 0.15H.

^{15}N NMR of **13b** (60.8 MHz, $(CD_3)_2SO$): δ 322.9 (*N*—O), 187.8 (*N*$^{+}$—Me).

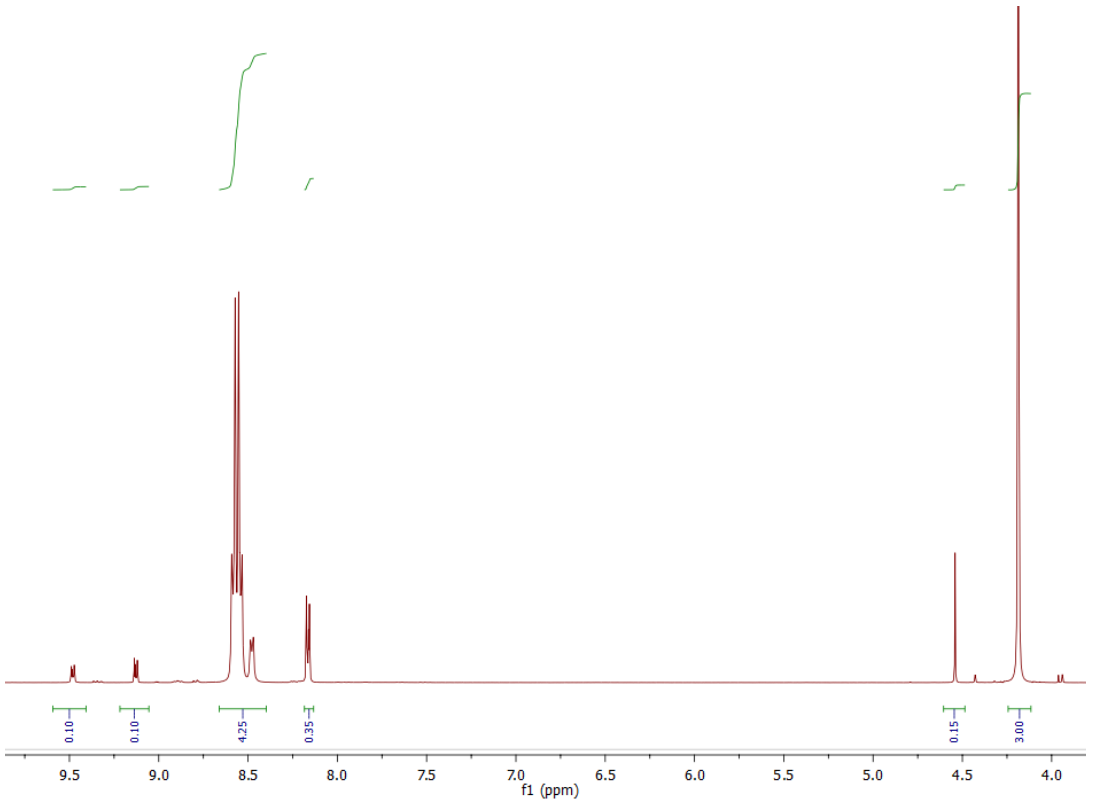

Figure S4: ^{1}H NMR spectrum of reaction mixture in CD_3CN, showing signals of **13b** (major product), some **15b** and starting material. The full spectrum is shown in Section 7.

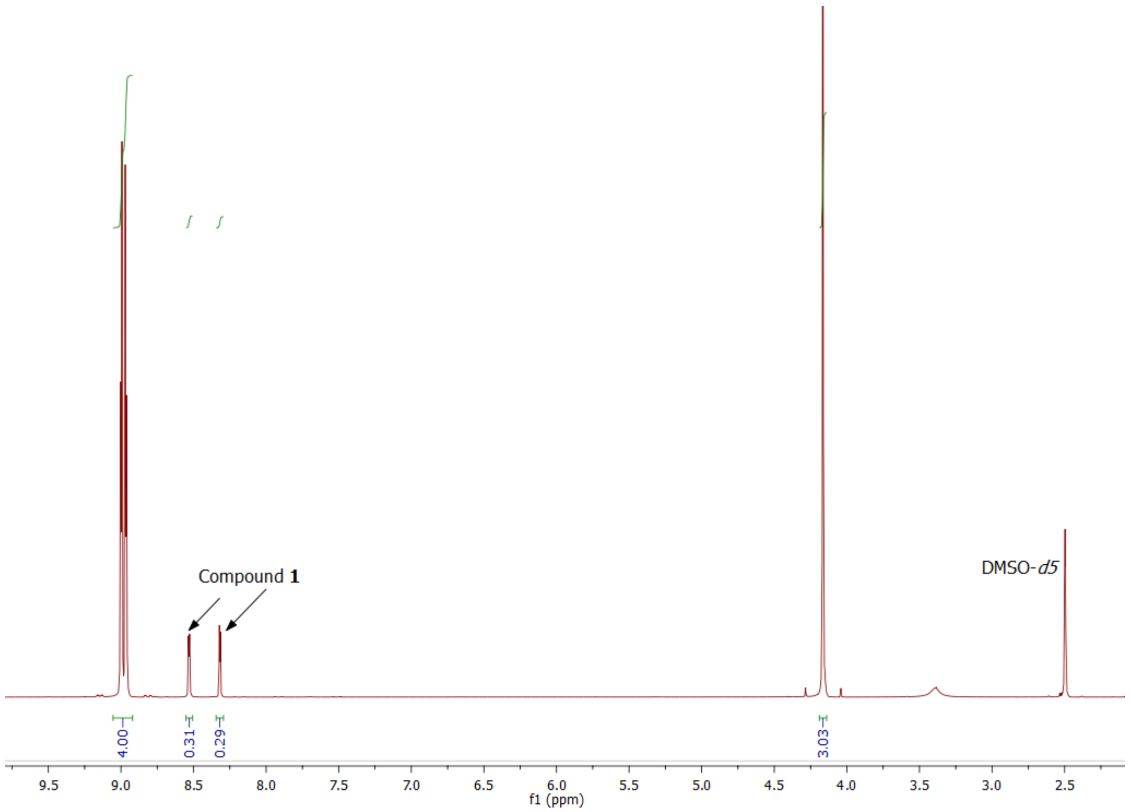

Figure S5: ^{1}H NMR spectrum of reaction mixture after removal of CD_3CN and addition of $(CD_3)_2SO$, showing signals of **13b** (major product) and starting material, but no **15b**. The full spectrum is shown in Section 7.

(c) <u>Experiment Showing Exclusive Formation of 13b in $(CD_3)_2SO$ – Contains ^{15}N and ^{13}C NMR Data</u>

Pyrazine *N*-oxide (**1**) (0.050 g, 0.52 mmol) was dissolved in $(CD_3)_2SO$ (0.65 ml) in a vial inside an inert atmosphere glove box. Methyl triflate (0.084 g, 0.51 mmol) was subsequently added dropwise. The reaction mixture was transferred to a NMR tube by syringe. The NMR tube was then sealed by a rubber septum cap and wrapped with PTFE tape. The septum was then covered with Parafilm and the tube transferred outside the glove box. The methoxydimethylsulfonium salt derived from $(CD_3)_2SO$ is likely to be the primary methylating agent in the reaction of **1** + MeOTf in $(CD_3)_2SO$.[10] As a consequence, the methylation of **1** is relatively slow. After 4 weeks the reaction mixture was subjected to ^1H and ^1H-^{15}N HMBC NMR spectroscopic characterization.

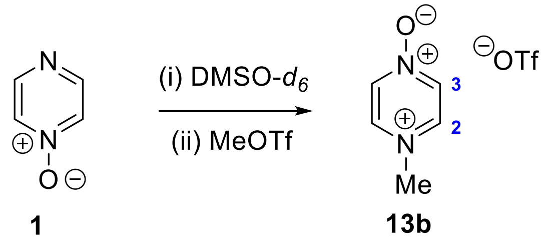

^1H NMR (600 MHz, $(CD_3)_2SO$)

Signals assigned to **13b**: δ 9.02 – 8.98 (m, 2H), 8.98 – 8.93 (m, 2H), 4.17 (s, 3H, CH_3).

Signals assigned to **1**: δ 8.55 – 8.51 (m, 2H), 8.34 – 8.30 (m, 2H). Relative to 1H of **13b**, 1H of **1** integrates for 0.23H.

A signal assigned to the methoxydimethylsulfonium salt of $(CD_3)_2SO$ is present at 3.98 ppm. Relative to 1H of **13b**, 1H of the salt integrates for 0.15H.

$^{13}C\{^1H\}$ NMR (150 MHz, $(CD_3)_2SO$)

Signals assigned to **13b**: 142.7 (C-2) , 138.7 (C-3), 120.7 (q, J = 322 Hz, triflate CF_3), 46.8.

Signals assigned to **1**: δ 148.2, 134.2.

Signals assigned to methoxydimethylsulfonium salt of $(CD_3)_2SO$: 62.2.

^{15}N NMR of **13b** (60.8 MHz, $(CD_3)_2SO$)

Signals assigned to **13b**: δ 322.9 (*N*—O), 187.7 (N^+—Me).

Signals assigned to **1**: δ 310.8, 303.7.

Conversion Calculation (based on consumption of the methoxydimethylsulfonium salt as the limiting reagent):

4H of Compound **13b** corresponds to 4.00, therefore 1H = 1.00

For the methoxydimethylsulfonium salt at 3.98 ppm, 3H = 0.46, therefore 1H = 0.15.

$$\text{Conversion} = \frac{1.00}{1.00 + 0.15} \times\ 100 = 87\%$$

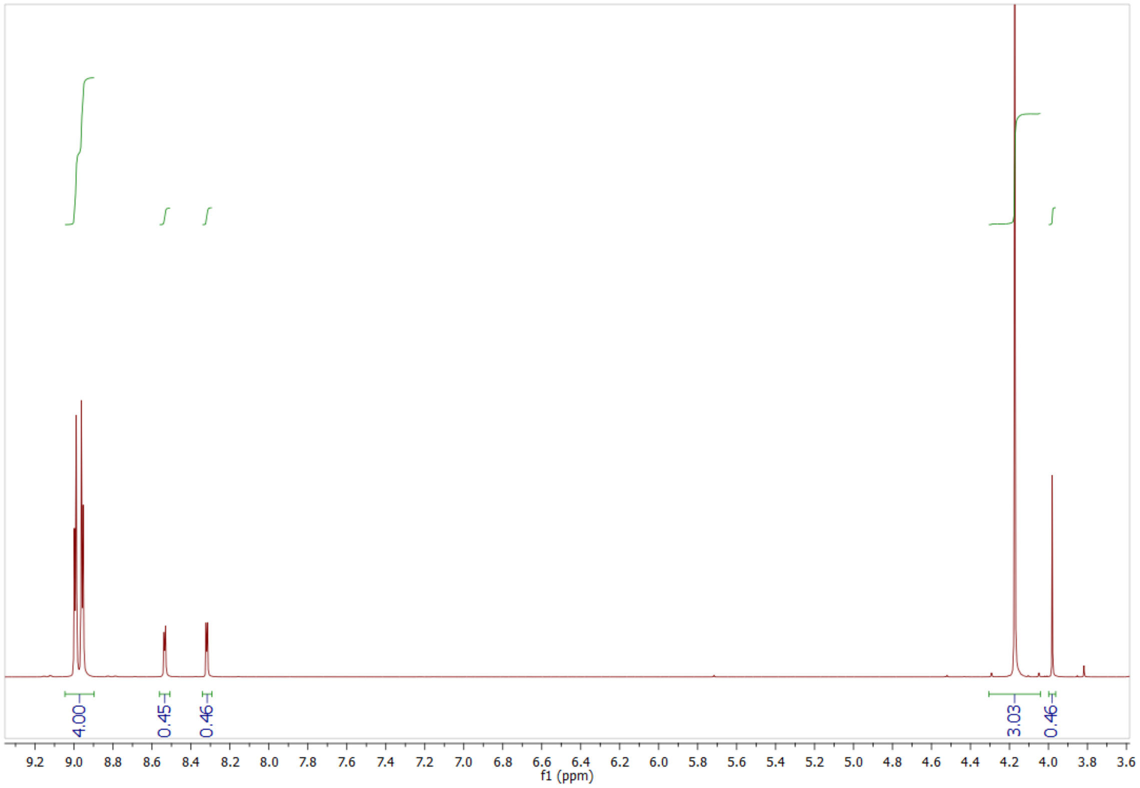

Figure S6: ^{1}H NMR spectrum showing product **13b** and **1** in $(CD_3)_2SO$. The full spectrum is shown in Section 7.

Preparation of 14

(a) <u>Experiment in CD_3CN – Quantitative Conversion to 14 – Contains ^{13}C NMR data</u>

The products of this reaction decompose upon exposure to moisture, and could not be isolated. The products were characterized by recording NMR spectra of the reaction mixture under inert atmosphere.

Pyrazine *N*-oxide (**1**) (0.016 g, 0.17 mmol), benzhydryl chloride (0.035 g, 0.17 mmol) and silver triflate (0.054 g, 0.21 mmol) were combined by the process described in General Procedure C to produce **14** in CD_3CN. NMR spectroscopic characterization of the product in CD_3CN was carried out. Quantitative conversion to **14** (based on consumption of the benzhydrylium ion) was observed.

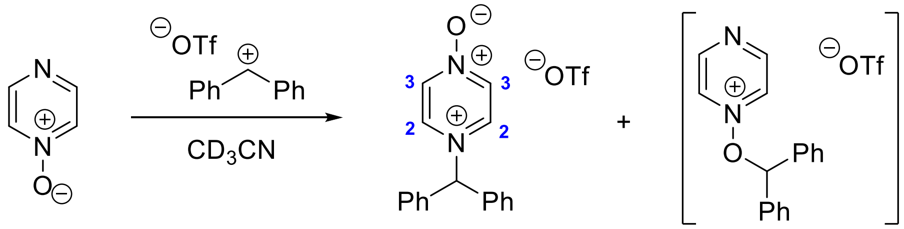

1 **14** **16** Not formed

^{1}H NMR (600 MHz, CD_3CN) δ 8.50 (app s,* 4H, H-2, H-3), 7.54 – 7.49 (m, 6H, Phenyl H-3, H-4 & H-5), 7.36 – 7.31 (m, 4H, Phenyl H-2 & H-6), 7.24 (s, 1H, C*H*Ph$_2$). Apparent singlet (app s) in ^{1}H NMR spectrum was appeared as two barely separated multiplets in other spectra of this compound.

$^{13}C\{^1H\}$ NMR (150 MHz, CD_3CN) δ 141.31 (C-3), 140.77 (C-2), 135.30 (Phenyl C-1), 131.05 (Phenyl C-4), 130.54 (Phenyl C-3 & C-5), 129.94 (Phenyl C-2 & C-6), 77.21 (*C*HPh$_2$).

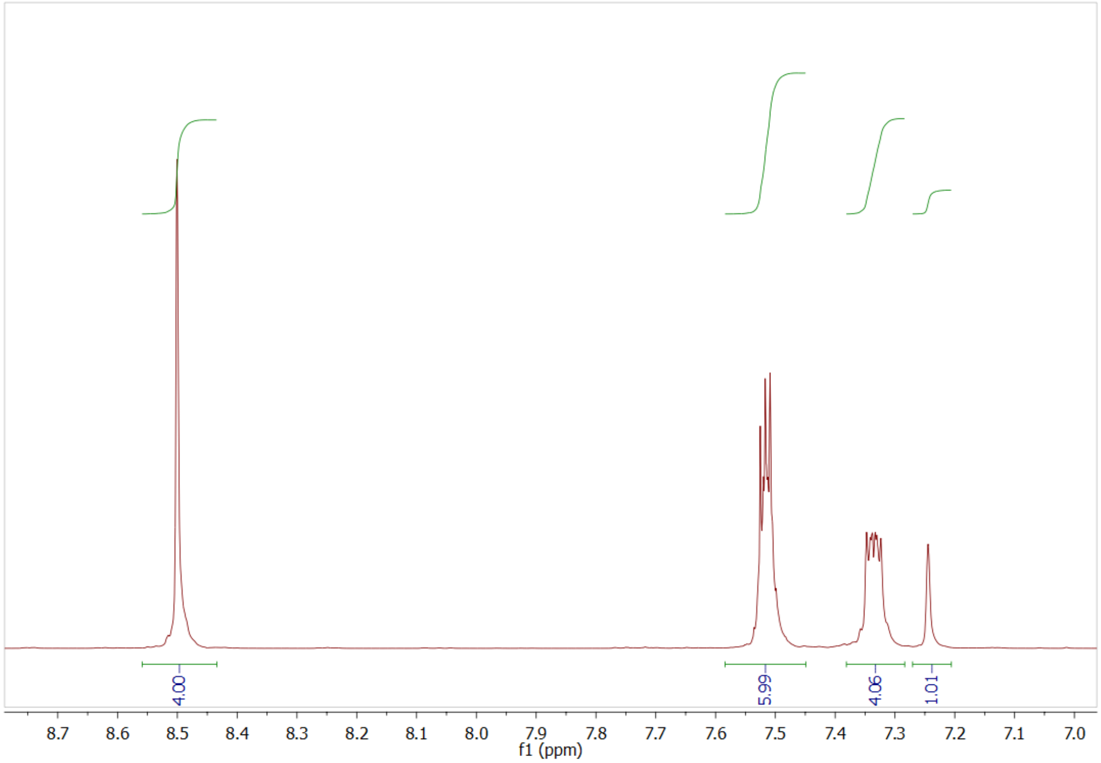

Figure S7: ^{1}H NMR spectrum in CD_2Cl_2 of **14**. The full spectrum is shown in Section 7.

(b) Experiment Showing Exclusive Formation of 14 in CH_2Cl_2 – Contains ^{15}N NMR Data

The products of this reaction decompose upon exposure to moisture, and could not be isolated. The products were characterized by recording NMR spectra of the reaction mixture under inert atmosphere.

Pyrazine *N*-oxide (**1**) (0.037 g, 0.39 mmol), benzhydryl chloride (0.077 g, 0.38 mmol) and silver triflate (0.113 g, 0.440 mmol) were combined by the process described in General Procedure C to produce **14** in CH_2Cl_2. The reaction mixture in CH_2Cl_2 was subjected to ^1H and ^1H-^{15}N HMBC NMR spectroscopic characterization using the solvent suppression protocol referred to in the General Experimental. No hydrolysis product can be definitively identified from the ^1H NMR spectrum, although a small amount of material not attributable to **14** is present. Conversion to **14** is estimated to be a minimum of 94% (based on integration of excess **1** relative to **14**).

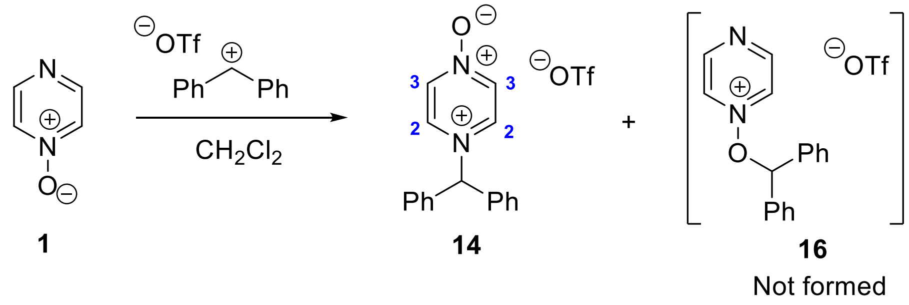

^1H NMR (600 MHz, CH_2Cl_2)

Assigned to **14**: δ 8.56 – 8.49 (m, 4H, H-2, H-3), 7.52 – 7.48 (m, 6H), 7.35 – 7.31 (m, 4H), 7.29 (s, 1H, Ph_2CH).

Assigned to **1**: δ 8.68 (app d, app J = 5.4 Hz, 2H), 8.48 (app d, app J = 5.5 Hz, 2H). Integration relative to 1H of **14** is 0.13H.

^{15}N NMR (60.8 MHz, CH_2Cl_2): δ 325.0 (*N*—O of **14**), 201.6 (N^+—Me of **14**).

The ^1H and ^1H-^{15}N HMBC NMR spectra are shown in Section 7.

4.3 Reactions of Quinoxaline *N*-Oxide (2)

Preparations of *N*-Methylquinoxalinium *N'*-oxide iodide (17a)

(a) Experiment Showing Formation of 17a in Low Yield

Quinoxaline *N*-oxide (**2**) (0.023 g, 0.16 mmol) was placed in a N_2-filled Schlenk flask. Methyl iodide (0.684 g, 4.82 mmol) was subsequently added dropwise *via* syringe. The flask was wrapped in foil and left in the dark for 48 hours, before the methyl iodide was removed under vacuum using General Procedure A. The flask was then opened and the red solid product (**17a**) was washed by addition of Et_2O, which was removed by cannula filtration. Three aliquots of dry Et_2O (3 ml each) were used in this manner to wash the product in very low yield (2 mg, 0.007 mmol, 4% yield). The recovered product (**17a**) was dissolved in $(CD_3)_2SO$ for ^{1}H NMR spectroscopic characterization. Some signals from residual Et_2O are present in the ^{1}H NMR spectrum.

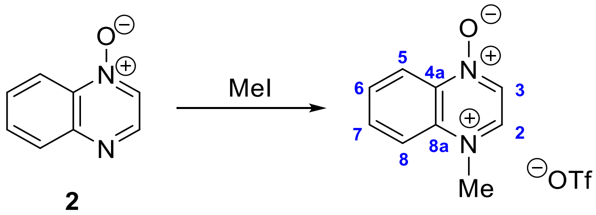

^{1}H NMR (600 MHz, $(CD_3)_2SO$) δ 9.46 (d, J = 5.2 Hz, 1H, H-2), 9.28 (d, J = 5.2 Hz, 1H, H-3), 8.61 (dd, J = 8.7, 1.2 Hz, 1H, H-8), 8.55 – 8.51 (m (app dd, signal resolution renders J values ambiguous), 1H, H-5), 8.34 (m, 1H, H-6), 8.21 – 8.15 (m, 1H, H-7), 4.49 (s, 3H, NCH_3).

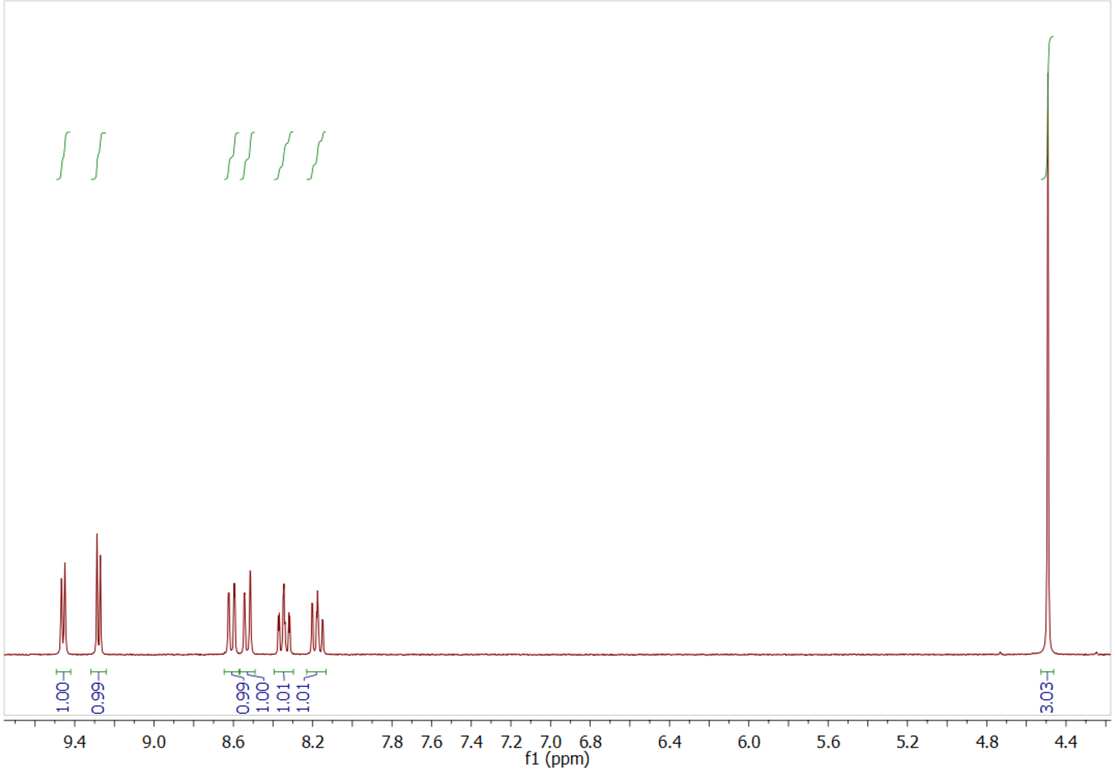

Figure S8: ^{1}H NMR spectrum of **17a** in $(CD_3)_2SO$. The full spectrum is shown in Section 7.

(b) Experiment Showing Formation of 17a in Low Yield – Contains ^{15}N NMR data

Quinoxaline *N*-oxide (**2**) (0.044 g, 0.30 mmol) was placed in a N_2-filled Schlenk flask. Methyl iodide (0.129 g, 0.91 mmol) was subsequently added dropwise *via* syringe. The MeI was removed under vacuum using General Procedure A after 18 hours and the solid product (**17a**) was washed by addition of dry Et_2O, which was removed by cannula filtration (under inert atmosphere). Three aliquots of dry Et_2O (0.4 ml each) were used in this manner to wash the product (yield = 0.014 g, 0.049 mmol, 16%). A sample of **17a** in dry $(CH_3)_2SO$ was then prepared for ^{1}H and ^{1}H-^{15}N HMBC NMR spectroscopic characterization using General Procedure B. Note: An initial attempt to dissolve the product in CH_2Cl_2 was unsuccessful, and a residual amount of this solvent can be seen in the spectrum.

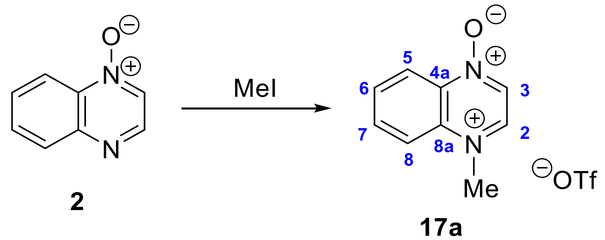

^{1}H NMR (600 MHz, $(CH_3)_2SO$) δ 9.42 (d, *J* = 5.2 Hz, 1H, H-2), 9.25 (d, *J* = 5.2 Hz, 1H, H-3), 8.59 – 8.56 (m (app dd, *J* values ambiguous), 1H, H-8), 8.51 – 8.48 (m (app dd, *J* values ambiguous), 1H, H-5), 8.34 – 8.29 (m, 1H, H-6), 8.16 – 8.12 (m, 1H, H-7), 4.46 (s, 3H, NC*H*$_3$).

^{15}N NMR (60.8 MHz, $(CH_3)_2SO$): δ 314.7 (*N*—O), 178.3 (*N*$^+$—Me).

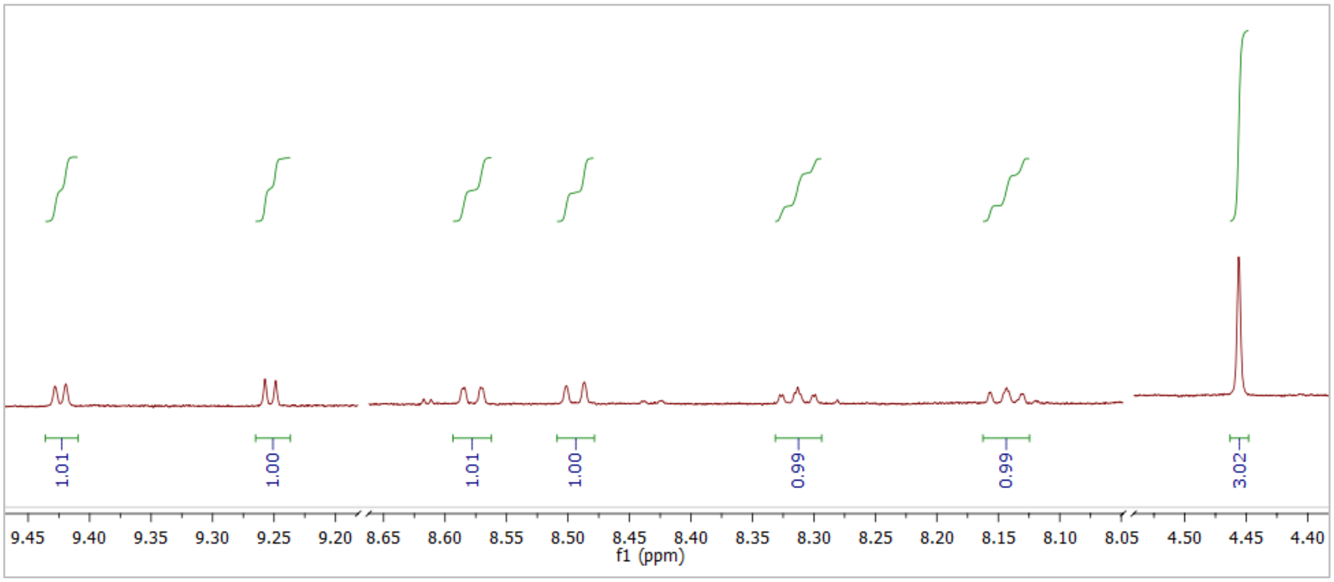

Figure S9: ^{1}H NMR spectrum of **17a** in $(CH_3)_2SO$. The full spectrum is shown in Section 7.

Note: This spectrum was recorded in non-deuterated solvent (using the solvent suppression protocol specified in the General Experimental above). Due to a combination of this and the low conversion to product that occurred in this reaction, the product signals are very small. However, the spectral details match well to the ^{1}H NMR spectrum obtained from another repetition of the same experiment, described in part (a), immediately above.

Preparations of 17b and 19b

(a) Experiment Showing Isolated Yield of 17b

Quinoxaline *N*-oxide (**2**) (0.323 g, 2.21 mmol) was dissolved in CH_3CN (10 ml) in a N_2-filled Schlenk flask. Methyl triflate (0.399 g, 2.43 mmol) was subsequently added dropwise. After 5 hours, the CH_3CN was removed under vacuum using General Procedure A, giving black crystals. The solid product (**17b**) was washed by addition of dry Et_2O, which was removed by cannula filtration (under inert atmosphere). Three aliquots of dry Et_2O (3 ml each) were used in this manner to wash the product (yield = 0.389 g, 1.25 mmol, 57%). A sample of **17b** in dry $(CD_3)_2SO$ was then prepared for ^1H and ^1H-^{13}C HMBC NMR spectroscopic characterization using General Procedure B.

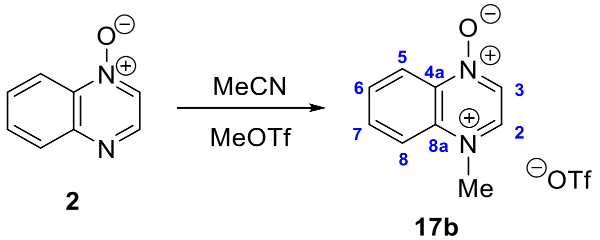

^{1}H NMR (300 MHz, $(CD_3)_2SO$) δ 9.45 (d, *J* = 5.2 Hz, 1H, H-2), 9.27 (d, *J* = 5.2 Hz, 1H, H-3), 8.61 (dd, *J* = 8.7, 1.2 Hz, 1H, H-8), 8.55 – 8.49 (m (app dd, signal resolution renders *J* values ambiguous), 1H, H-5), 8.39 – 8.30 (m, 1H, H-6), 8.17 (m, 1H, H-7), 4.49 (s, 3H, CH_3).

^{13}C{^{1}H} NMR (75 MHz, $(CD_3)_2SO$) δ 144.5 (C-2), 140.0 (C-4a), 136.5 (C-6), 135.9 (C-8a), 133.2 (C-7), 133.0 (C-3), 121.1 (C-5), 120.0 (C-8), 44.2 (CH_3)

IR (ATR-FTIR), cm^{-1}: 3115 (w), 3092 (w), 1629 (m), 1536 (m), 1408 (m), 1256 (s), 1029 (s), 638 (m).

HRMS-ESI+ (*m/z*): calculated for $[M]^+$ = $C_9H_9N_2O$ 161.0709; found 161.07069.

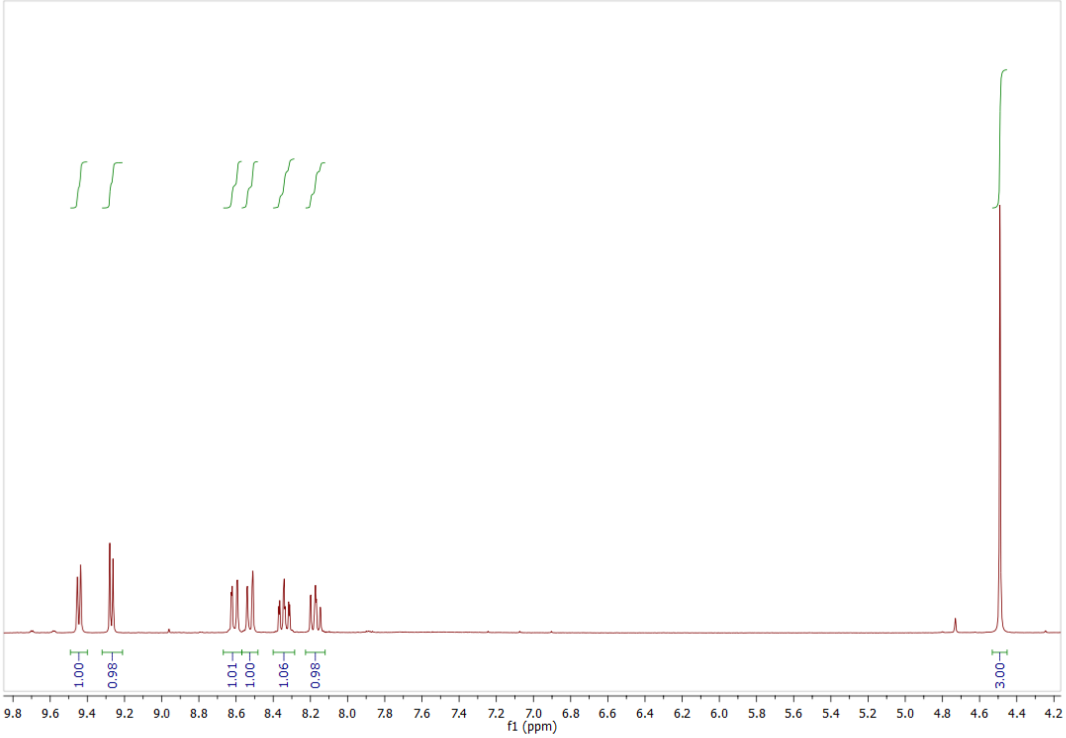

Figure S10: ^{1}H NMR spectrum in $(CD_3)_2SO$ of **17b**. The full spectrum is shown in Section 7.

(b) Experiment Showing N- vs O-Alkylation Product Ratio (17b vs 19b) – Contains ^{13}C & ^{15}N NMR Data

Quinoxaline *N*-oxide (**2**) (0.047 g, 0.32 mmol) was dissolved in CD_3CN (0.65 ml) in a N_2-filled Schlenk flask. Methyl triflate (0.045 g, 0.27 mmol) was subsequently added dropwise. The reaction mixture was transferred to an NMR tube and analyzed by NMR spectroscopy using General Procedure B.

Note: Insufficient concentrations of **2** and minor product **19b** in the following spectra meant that unambiguous assignment of hydrogen and carbon NMR signals to specific sites in the structures of these compounds was not possible.

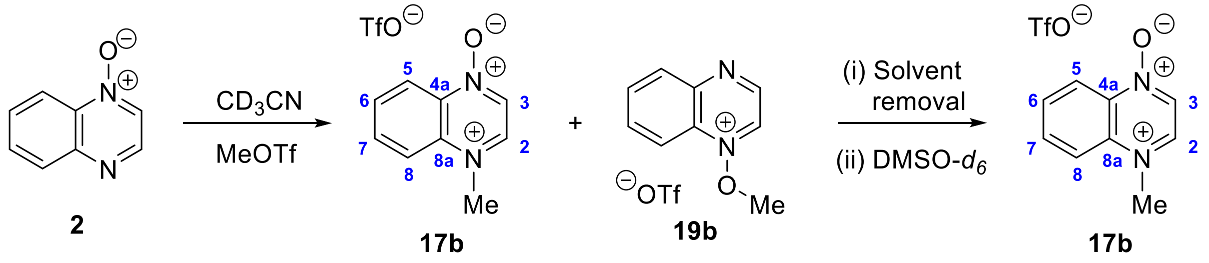

^{1}H NMR (400 MHz, CD_3CN)

Assigned to **17b**: δ 8.98 (d, *J* = 5.1 Hz, 1H, H-2), 8.79 (d, *J* = 5.2 Hz, 1H, H-3), 8.62 – 8.55 (m, 1H, H-5), 8.48 – 8.23 (m, 2H, H-5 and H-7), 8.16 – 8.06 (m, 1H, H-8), 4.47 (s, 3H, NCH_3).

Assigned to **19b**: δ 9.63 (d, *J* = 3.4 Hz, 1H), 9.56 (d, *J* = 3.2 Hz, 1H), 8.62 – 8.55 (m, 1H), 8.48 – 8.23 (m, 2H), 8.16 – 8.06 (m, 1H), 4.69 (s, 3H, OCH_3). Relative to 1H of **17b**, 1H of **19b** integrates for 0.12H.

Assigned to **2**: δ 8.69 (d, *J* = 3.6 Hz, 1H), 8.48 – 8.23 (m, 2H), 8.16 – 8.06 (m, 1H), 7.94 – 7.87 (m, 1H), 7.84 – 7.78 (m, 1H). Relative to 1H of **17b**, 1H of **2** integrates for approximately 0.33H.

The signal between 8.62 and 8.55 ppm contains a 1H signal from **17b** and a 1H signal from **19b**.

The signal between 8.48 and 8.23 ppm contains a 2H signal from **17b**, a 2H signal from **19b** and a 2H signal from **2**.

The signal between 8.16 and 8.06 ppm contains a 1H signal from **17b**, a 1H signal from **19b** and a 1H signal from **2**.

^{13}C{^{1}H} NMR (100 MHz, CD_3CN)

Assigned to **17b**: δ 143.2 (C-2), 140.2 (C-4a), 136.3 (C-6), 135.5 (C-8a), 132.9 (C-7), 132.1 (C-3), 120.1 (C-5), 119.7 (C-8), 44.1 (*C*H_3)

Assigned to **19b**: δ 147.2, 144.7, 140.2, 136.8, 134.7, 131.7, 129.4, 119.1, 116.3, 68.9.

Ratio of N-alkylation and O-alkylation Products (from integrations in ^{1}H NMR spectrum):

3H of Compound **17b** = 3.00 – Therefore 1H = 1.00

3H of compound **19b** = 0.36 – Therefore 1H = 0.12

$$\text{Ratio} = \frac{1.00}{1.00 + 0.12} \times 100 = 89\% \text{ N alkylation}$$

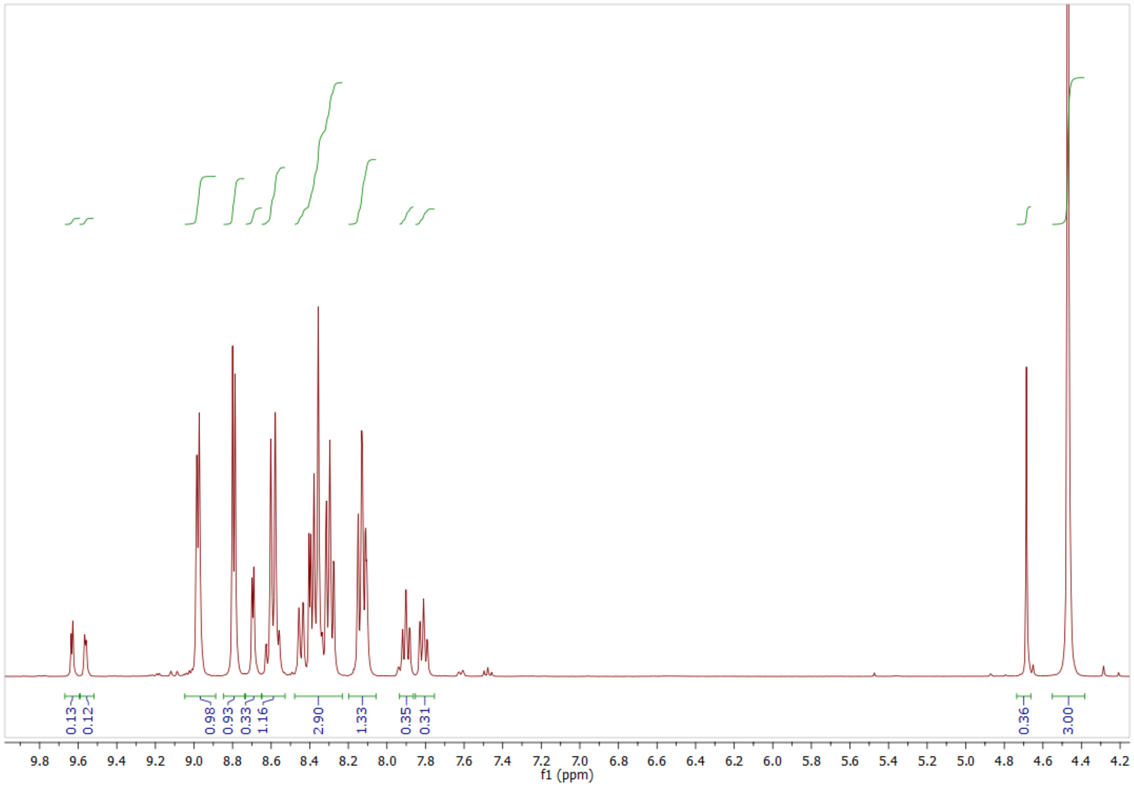

Figure S11: ^{1}H NMR Spectrum of **17b**, **19b** and **2** in CD_3CN. The full spectrum is shown in Section 7.

The CD_3CN was removed using General Procedure A and the product mixture was re-dissolved in $(CD_3)_2SO$ to allow a ^{1}H-^{15}N HMBC NMR spectrum to be recorded. Product **19b** did not survive the solvent removal process.

^{1}H NMR (600 MHz, $(CD_3)_2SO$)

Assigned to **17b**: δ 9.46 (d, *J* = 5.1 Hz, 1H, H-2), 9.29 (d, *J* = 5.1 Hz, 1H, H-3), 8.60 (app d,* app *J* = 8.6 Hz, 1H, overlaps partially with signal of **2**, H-8), 8.53 (app d,* app *J* = 8.8 Hz, 1H, H-5), 8.37 – 8.32 (m, 1H, H-6), 8.20–8.16 (m, overlaps with signal of **2**, 1H, H-7), 4.50 (s, 3H, NC*H*$_3$). See NMR spectra in experiments described above – these signals are not doublets; signal resolution in this particular spectrum is too low to observe the fine structure of these signals.

Assigned to **2**: δ 8.78 (d, *J* = 3.5 Hz, 1H), 8.63 (d, *J* = 3.5 Hz, 1H, overlaps partially with signal of **17b**), 8.46 – 8.42 (m (app d, app *J* = 8.5 Hz), 1H), 8.16 – 8.11 (m, overlaps with signal of **17b**, 1H), 7.96 – 7.92 (m, 1H), 7.88 – 7.84 (m, 1H). Relative to 1H of **17b**, 1H of **2** integrates for 0.26H.

The region between 8.64 and 8.45 ppm contains two 1H signals from **17b** and a 1H signal from **2**.

The region between 8.20 and 8.11 ppm contains a 1H signal from **17b** and a 1H signal from **2**.

^{15}N NMR (60.8 MHz, $(CD_3)_2SO$): δ 314.4 (*N*—O), 178.0 (*N*$^+$—Me).

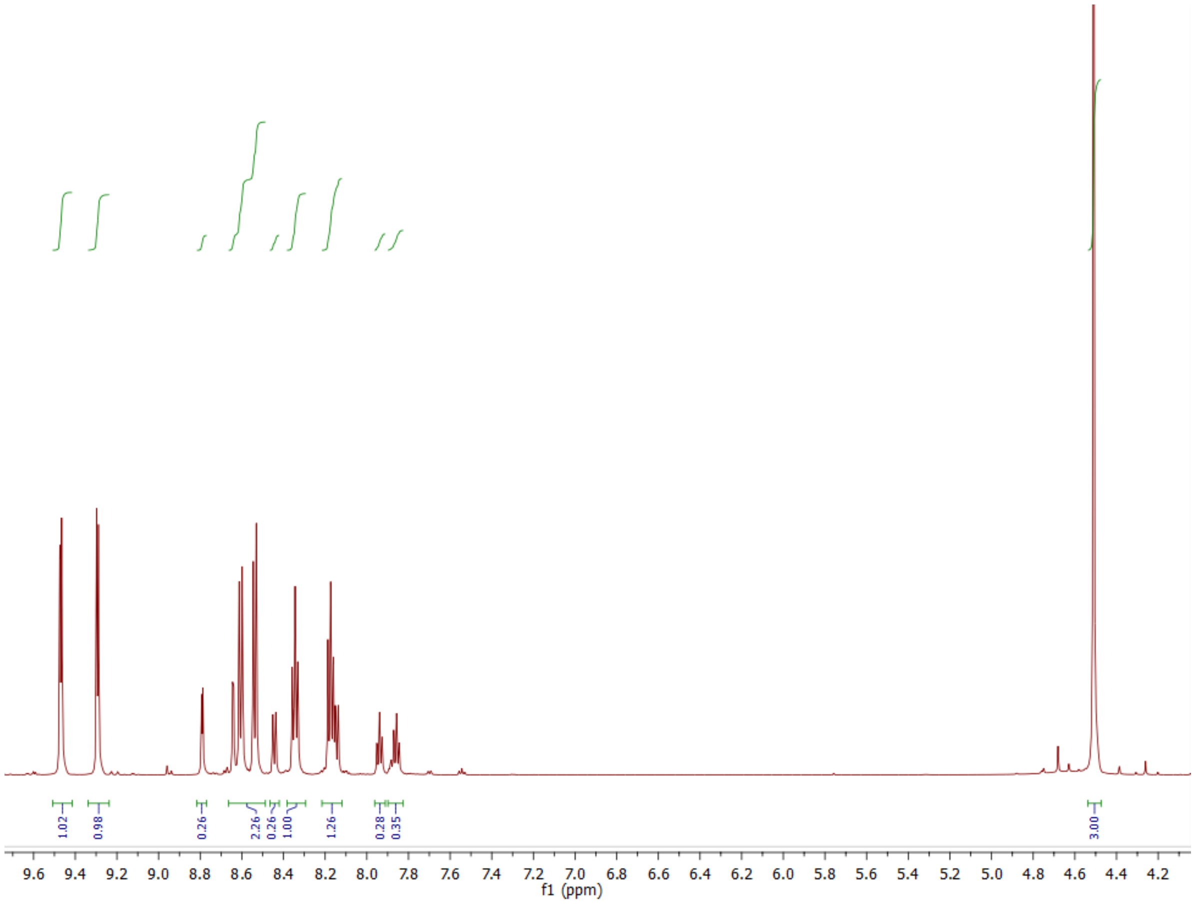

Figure S12: ^{1}H NMR Spectrum of **17b** and **2** in $(CD_3)_2SO$. Note the absence of signals assigned to **19b**. The full spectrum is shown in Section 7.

(c) **<u>Experiment Showing Exclusive Formation of 17b in $(CD_3)_2SO$ – Contains ^{15}N NMR Data</u>**

Quinoxaline *N*-oxide (**2**) (0.057 g, 0.39 mmol) was dissolved in $(CD_3)_2SO$ (0.8 ml) in a vial inside an inert atmosphere glove box. Methyl triflate (0.050 g, 0.31 mmol) was subsequently added dropwise. The reaction mixture was transferred to a NMR tube by syringe. The NMR tube was then sealed by a rubber septum cap and wrapped with PTFE tape. The septum was then covered with Parafilm and the tube transferred outside the glove box. $(CH_3)_2SO$ is known to react with methylating agents (e.g. dimethyl sulfate) to give methoxydimethylsulfonium salt.[10] The resulting methoxysulfonium salt acts as the primary methylating agent in the reaction of **2** + MeOTf in $(CD_3)_2SO$. As a consequence, the methylation of **2** is relatively slow. After 4 weeks the reaction mixture was subjected to ^1H and ^1H-^{15}N HMBC NMR spectroscopic characterization.

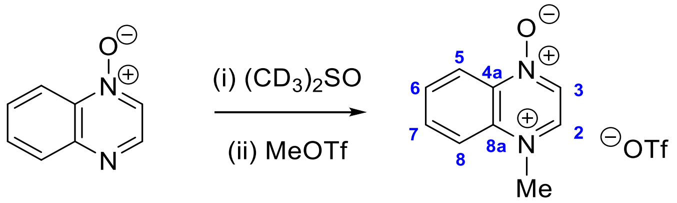

2 **17b**

^{1}H NMR (600 MHz, $(CD_3)_2SO$)

Assigned to **17b**: δ 9.46 (d, *J* = 5.1 Hz, 1H, H-2), 9.27 (d, *J* = 5.1 Hz, 1H, H-3), 8.58 (dd, *J* = 8.7, 1.1 Hz, 1H, H-8), 8.54 – 8.50 (m (app dd, signal resolution renders *J* values ambiguous), 1H, H-5), 8.34 – 8.30 (m, 1H, H-6), 8.18 – 8.14 (m, 1H, overlaps partially with signal of **2**, H-7), 4.51 (s, 3H, CH_3).

Assigned to **2**: δ 8.78 (d, *J* = 3.6 Hz, 1H), 8.62 (d, *J* = 3.6 Hz, 1H), 8.42 (dd, *J* = 8.6, 1.2 Hz, 1H), 8.14 – 8.10 (1H, overlaps partially with signal of **17b**), 7.95 – 7.89 (m, 1H), 7.87 – 7.81 (m, 1H). Relative to 1H of **17b**, 1H of **2** integrates for approximately 0.78H.

The region between 8.17 and 8.10 ppm contains a 1H signal from **17b** and a 1H signal from **2**.

A signal assigned to the methoxydimethylsulfonium salt of $(CD_3)_2SO$ is present at 3.99 ppm. Relative to 1H of **17b**, 1H of the salt integrates for 0.28H.

^{15}N NMR (60.8 MHz, $(CD_3)_2SO$)

Assigned to **17b**: δ 314.4 (*N*—O), 177.9 (N^+—Me).

Assigned to **2**: δ 302.3, 299.7.

<u>Conversion Calculation </u>(based on consumption of the methoxydimethylsulfonium salt as the limiting reagent):

For the methoxydimethylsulfonium salt at 3.99 ppm, 3H = 0.84 relative to 1H of **17b**, therefore 1H = 0.28.

$$\text{Conversion} = \frac{1.00}{1.00 + 0.28} \times\ 100 = 78\%$$

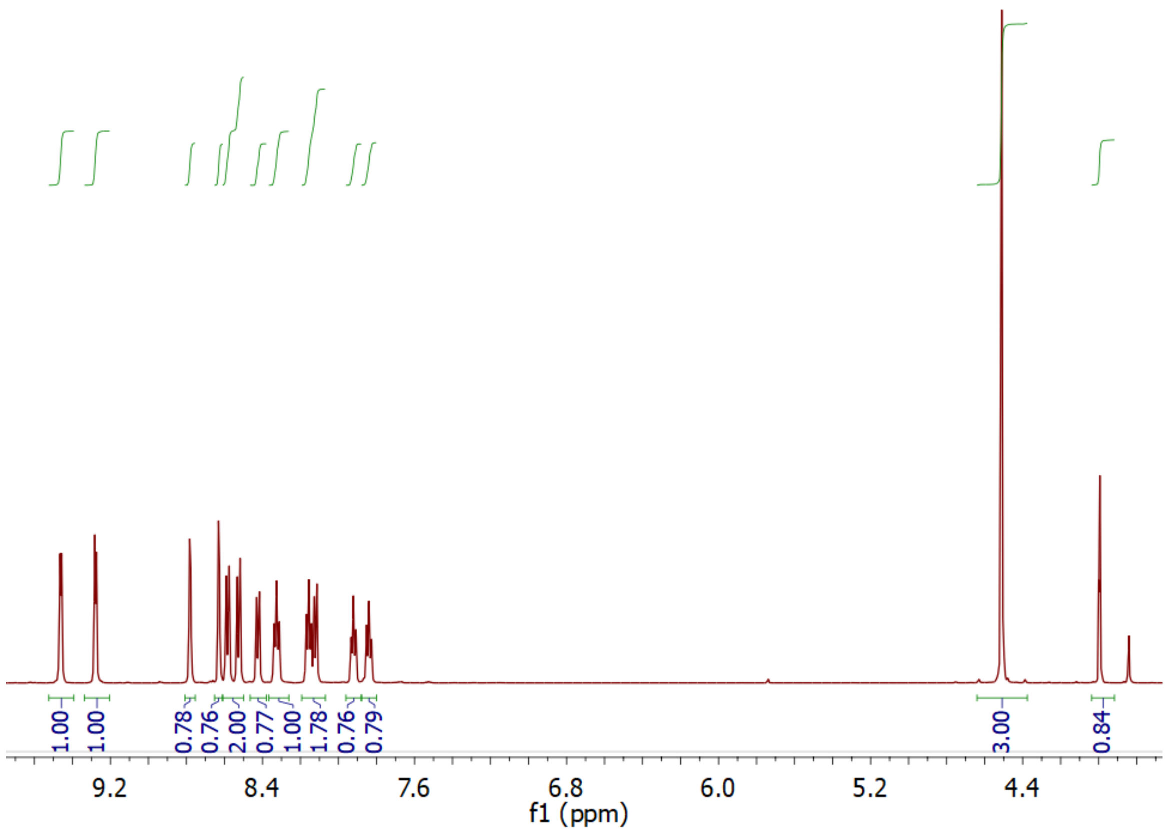

Figure S13: ^{1}H NMR Spectrum in $(CD_3)_2SO$ of **17b** and **2** in $(CD_3)_2SO$. The full spectrum is shown in Section 7.

Preparation of 18

The products of this reaction decompose upon exposure to moisture, and could not be isolated. Consequently, the products were characterized by recording NMR spectra of the reaction mixture under inert atmosphere.

Quinoxaline *N*-oxide (**2**) (0.026 g, 0.18 mmol), 4-methylbenzhydryl chloride (0.038 g, 0.18 mmol) and silver triflate (0.044 g, 0.17 mmol) were combined by the process described in Procedure C to produce **18** (major product) + **20** (minor product) in CD_2Cl_2. The reaction mixture in CD_2Cl_2 was then prepared for ^{1}H and ^{1}H-^{15}N HMBC NMR spectroscopic characterization using General Procedure B.

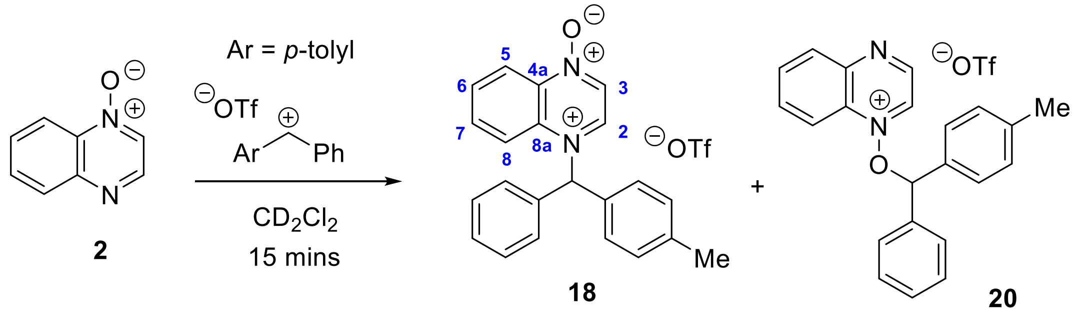

^{1}H NMR (600 MHz, CD_2Cl_2)

Signals assigned to **18**: δ 8.75 (d, *J* = 5.5 Hz, 1H, H-3), 8.60 (dd, *J* = 8.7, 1.1 Hz, 1H, H-8), 8.53 (d, *J* = 5.5 Hz, 1H, H-2), 8.42 (app d, app *J* = 8.9 Hz, 1H, H-5), 8.10 (m, 1H, H-7), 8.02 – 7.97 (m, 1H, H-6), 7.81 (s, 1H, C*H*PhTol), 7.51 – 7.46 (m, 3H, Phenyl H-3, H-4 & H-5), 7.39 – 7.22 (m, 6H, Phenyl H-2 & H-6, Tolyl H-2, H-3, H-5 & H-6), 2.37 (s, 3H, CH_3).

Signals assigned to **20**: δ 8.83 (d, *J* = 3.6 Hz, 1H), 8.28 (d, *J* = 8.4 Hz, 1H) and 7.87 (m, 1H). Relative to 1H of **18**, 1H of **20** integrates for 0.10H.

A peak assigned to a hydrolysis product is present at 5.38 ppm. The signals of the aromatic protons of this product also contribute to the integration of the multiplet at 7.39 – 7.22 ppm. Relative to 1H of **18**, 2H of the hydrolysis product integrates for 0.08H.

^{13}C{^{1}H} NMR (150 MHz, CD_2Cl_2)

Signals assigned to **18**: δ 141.4 (Tolyl C-4), 141.2 (C-4a), 140.7 (C-2), 137.5 (C-7), 135.6 (C-8a), 134.8 (Phenyl C-1), 133.5 (C-6), 133.1 (C-3), 130.8 (Tolyl C-3 & C-5), 130.2 (Phenyl C-4), 130.1 (Phenyl C-3 & C-5), 129.5 (Tolyl C-2 & C-6), 129.2 (Phenyl C-2 & C-6), 121.4 (C-5), 121.1 (C-8), 73.2 (*C*HPhTol), 21.3 (*C*H_3).

A ^{13}C NMR signal assigned to the $CF_3SO_3^-$ ion is present at δ 120.72 (q, *J* = 320 Hz).

Note: Low concentration of minor product **20** in the ^{13}C{^{1}H} NMR spectrum meant that assignment of the very small signals present in the spectrum to this compound could not be done unambiguously.

^{15}N NMR (60.8 MHz, CD_2Cl_2)

Signals assigned to **18**: δ 317.6 (*N*—O), 190.5 (N^+—Me).

No correlations were observed to the ^{1}H NMR signals of the minor product, **20**.

Ratio of N-alkylation and O-alkylation Products:

1H of compound **18** = 1.00

1H of compound **20** = 0.10

$$\text{Ratio} = \frac{1.00}{1.00 + 0.10} \times\ 100 = 91\%\ \text{N alkylation}$$

<u>Conversion Calculation</u> (based on consumption of the benzhydrylium ion as the limiting reagent):

1H of Compound **18** corresponds to 1.00.

For the hydrolysis product at 5.38ppm, 2H = 0.08. Therefore, since two equivalents of benzhydrylium ion are consumed in hydrolysis (formation of bis(benzhydryl) ether), the conversion was:

$$\text{Conversion} = \frac{1.00}{1.00 + 0.08} \times\ 100 = 93\%$$

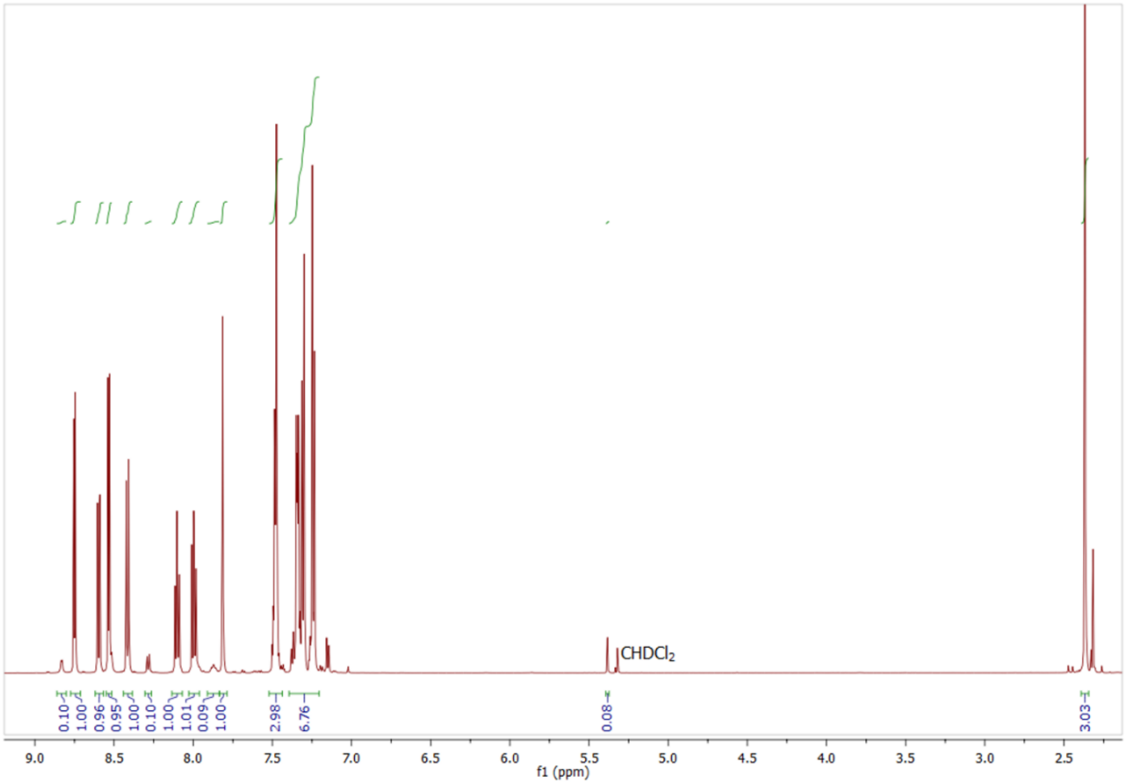

Figure S14: ^{1}H NMR spectrum in CD_2Cl_2 of **18**. The full spectrum is shown in Section 7.

4.4 Reactions of Pyrimidine *N*-Oxide (3)

Preparations of 21b and 23b

Removing the solvent from reaction mixtures containing **21b** and/or **23b** causes decomposition of **21b**. Formation of some quantity of degradation products was observed in all instances of reactions of **3** with MeOTf (see below), regardless of whether CD_3CN, MeCN or $(CD_3)_2SO$ were used as solvent *even if the solvent was not removed*. Taking steps to protect the reaction mixture from light also did not prevent the formation of these degradation products. It is not clear whether the degradation products observed directly in reaction mixtures by ^{1}H NMR spectroscopy (reactions in CD_3CN or $(CD_3)_2SO$ – see below) are derived from decomposition of **21b** or **23b** or both, or if some separate process leads to the formation of the decomposition products observed in the reaction mixtures. Although **23b** survives solvent removal, attempts to isolate it from the decomposition products through crystallization under inert atmosphere (in a Schlenk flask) were unsuccessful, resulting only in formation of further decomposition product(s). Since neither **21b** nor **23b** could be isolated, it was necessary to characterize these products in the reaction mixtures in which they formed by NMR spectroscopy under inert atmosphere. A high resolution mass spectrum of **23b** (sample maintained under inert atmosphere) was also obtained by subjecting a reaction mixture known (from NMR spectroscopic analysis) to contain only a small amount of decomposition product to electrosptray ionization mass spectrometric analysis (see below). This compound (with dimethylsulfate counter-ion rather than triflate) has been characterized previously.[11]

(a) <u>Experiment Showing Approximate Isolated Yield of 23b</u>

Pure samples of compounds **23b** and/or **21b** could not be obtained from this reaction for the reasons given at the start of section 4.4 (just above).

Pyrimidine *N*-oxide (**3**) (0.195 g, 2.03 mmol) was dispensed into a Schenk flask and sealed in a glove box. The flask was removed from the glove box and attached to a Schlenk line, and the solid was then dissolved in dry CH_3CN (3 ml). Methyl triflate (0.342 g, 2.08 mmol) was subsequently added dropwise. The flask was wrapped with aluminium foil and the reaction mixture was stirred for 24 hours.

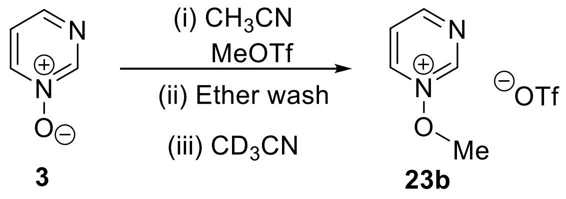

All operations and manipulations of the product were carried out under inert atmosphere – *i.e.* the product was kept in a Schlenk flask under an atmosphere of N_2 throughout. Dry Et_2O (3 ml) was then added to the reaction mixture, which caused the separation of a yellow oil from the reaction mixture. The supernatant (CH_3CN/Et_2O) was carefully removed by cannula. Two further aliquots of dry Et_2O (3 ml each) were then used to wash the yellow oil. In each case, the Et_2O supernatant was removed by cannula, as above. The product was dried by passing a stream of N_2 gas over the oil to avoid exposing the product to vacuum (for the reasons given at the beginning of section 4.4). The oil obtained contained small amounts of decomposition products seen in all experiments involving reaction of **3** with MeOTf (see

below). The amount of decomposition product present (based on ^{1}H NMR spectroscopic analysis of theis material) is sufficiently small to quote an approximate isolated yield for **23b** of 404 mg (1.55 mmol, 77% yield) from this experiment. All attempts to purify this material further (to obtain completely pure **23b**) resulted in decomposition of the product. A small sample of the product was dissolved in dry CD_3CN and analyzed by NMR spectroscopy using General Procedure B. A separate sample of **23b** was prepared in dry MeCN (approximately 1 mg ml^{-1}) and transferred to a mass spectrometry vial contained in a Schlenk flask under an atmosphere of nitrogen. The sample was maintained under inert atmosphere until directly prior to recording the mass spectrum.

^{1}H NMR (300 MHz, CD_3CN) Signals assigned to **23b**: δ 9.77 (dd, *J* = 2.3, 0.8 Hz, 1H), 9.43 – 9.34 (m, 2H), 8.23 (ddd, *J* = 6.8, 4.9, 0.9 Hz, 1H), 4.47 (s, 3H).

HRMS-ESI+ (*m/z*): Calculated for $[M]^+$ = $C_5H_7N_2O$ 111.0553; found 111.0550 (44%). Calculated for $[M + H + OTf]^+$ = $C_6H_8N_2O_4SF_3$ 261.0157; found 261.0150 (100%). We assign the second peak to the dicationic N-protonated adduct of **23b** associated with triflate to give an entity with a single net positive charge.

Note: The ^{1}H signal at δ 9.77 in compound **23b** has an extremely long relaxation time. A 30° pulse and a relaxation delay of 60 seconds were used during acquisition of the spectrum shown in Fig. S14, leading to a set of internally consistent integrations for the ^{1}H NMR signals of **23b**.

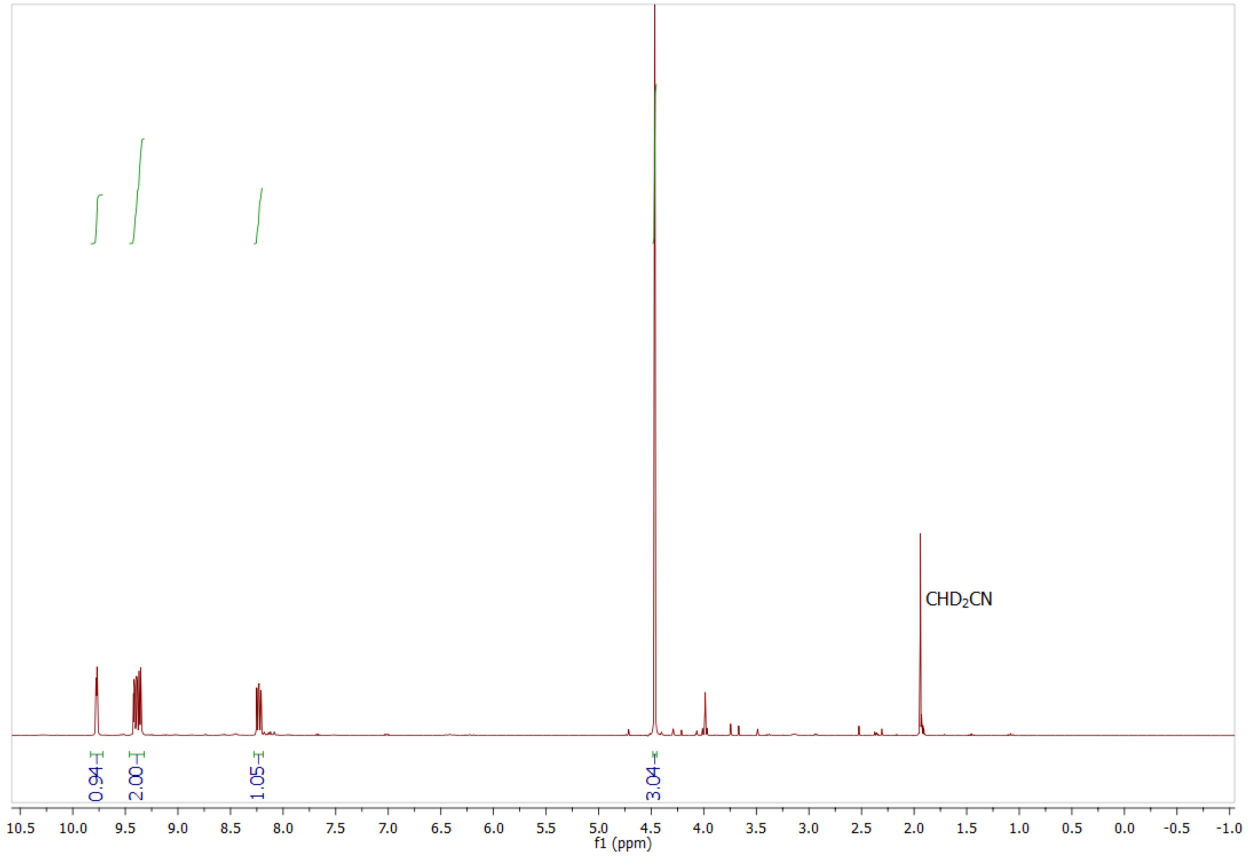

Figure S15: ^{1}H NMR spectrum in CD_3CN of **23b**. Small signals of decomposition products are present between 4.5 and 2.0 ppm.

(b) Reaction in MeCN – after solvent removal only 23b is observed – Contains ^{15}N NMR Data

Pure samples of compounds **23b** and/or **21b** could not be obtained from this reaction for the reasons given at the start of section 4.4.

Pyrimidine *N*-oxide (**3**) (0.046 g, 0.48 mmol) was dissolved in CH_3CN (5 ml) in a N_2-filled Schlenk flask. Methyl triflate (0.057 g, 0.35 mmol) was subsequently added dropwise. After *ca.* 20 minutes, the CH_3CN was removed under vacuum using General Procedure A and the solid product (**23b**) was washed by addition of dry Et_2O, which was removed by cannula filtration (under inert atmosphere). Three aliquots of dry Et_2O (2 ml each) were used in this manner to wash the product. A sample of **23b** in dry $(CH_3)_2SO$ was then prepared for ^{1}H and ^{1}H-^{15}N HMBC NMR spectroscopic characterization using General Procedure B.

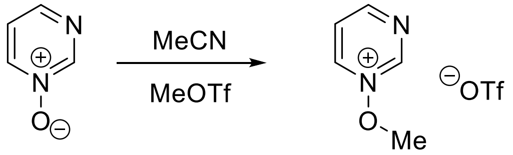

^{1}H NMR (600 MHz, $(CH_3)_2SO$)

Signals assigned to **23b**: δ 10.21 (app d, app *J* = 1.8 Hz, 1H), 9.90 – 9.83 (m, 1H), 9.44 (dd, *J* = 4.8, 1.5 Hz, 1H), 8.40 – 8.36 (m, 1H), 4.45 (s, 3H).[11]

Signals assigned to **3**: δ 9.07 (s, 1H, H-2), 8.61 – 8.56 (m, 1H, H-6), 8.33 – 8.29 (m, 1H, H-4), 7.58 – 7.52 (m, 1H, H-5).[11] Relative to 1H of **23b**, 1H of **3** integrates for approximately 0.71H.

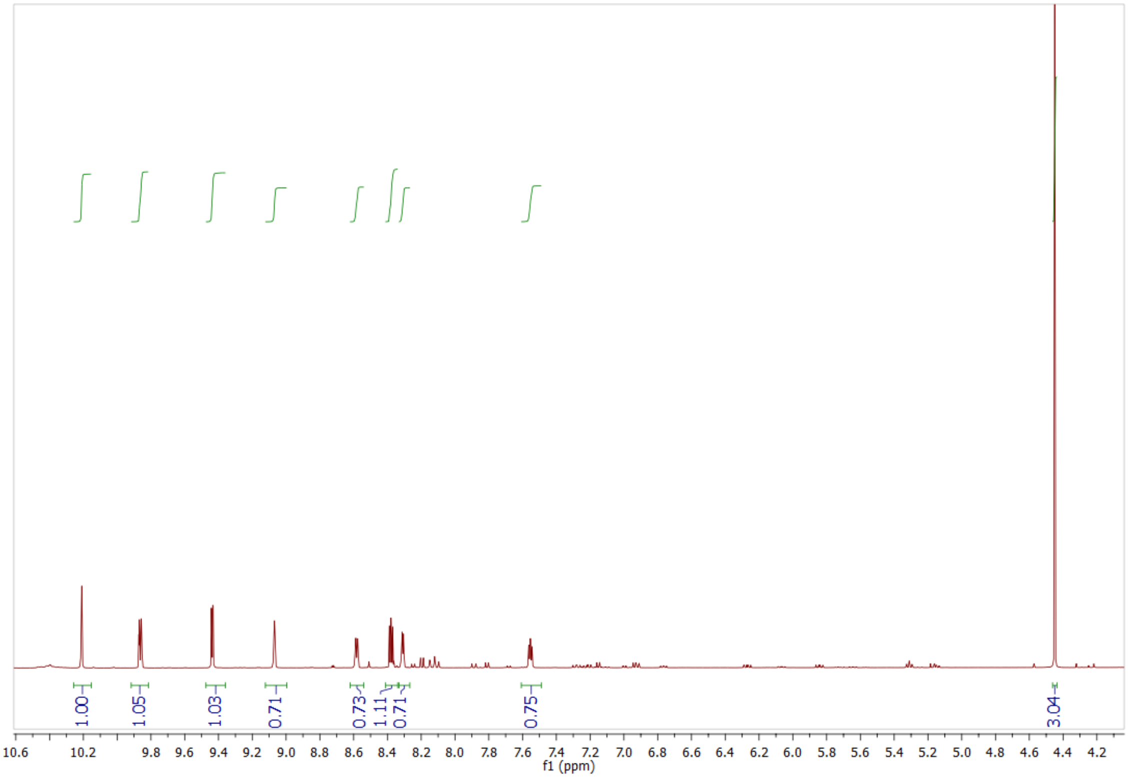

Figure S16: ^{1}H NMR spectrum in $(CH_3)_2SO$ of **23b**, containing signals assigned to **3**. The full spectrum is shown in Section 7

(c) Experiment Showing N- vs O-Alkylation Product Ratio (21b vs 23b) in CD_3CN – Contains ^{15}N and ^{13}C NMR Data

Pure samples of compounds **23b** and/or **21b** could not be obtained from this reaction for the reasons given at the start of section 4.4.

Pyrimidine *N*-oxide (**3**) (0.045 g, 0.47 mmol) was dissolved in CD_3CN (0.65 ml) in a N_2-filled Schlenk flask. Methyl triflate (0.067 g, 0.41 mmol) was then added dropwise. The reaction mixture was transferred to an NMR tube and analyzed by NMR spectroscopy using General Procedure B.

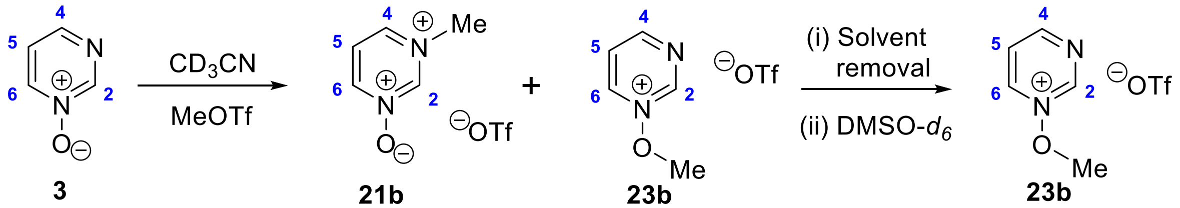

3 **21b** **23b** **23b**

^{1}H NMR (400 MHz, CD_3CN)

Signals assigned to **23b**: δ 9.81 (app d, app *J* = 1.5 Hz, 1H, H-2), 9.47 – 9.37 (m, 2H, H-4 and H-6), 8.31 – 8.24 (m, 1H, H-5), 4.51 (s, 3H, OC*H*$_3$).

Signals assigned to **21b**: δ 9.56 (s, 1H, H-2), 8.90 (app d, app *J* = 6.8 Hz, 1H), 8.58 (app d, app *J* = 6.0 Hz, 1H), 8.01 (app t, app *J* = 6.0 Hz, 1H), 4.32 (s, 3H, NC*H*$_3$). Relative to 1H of **23b**, 1H of **21b** integrates for 0.08H.

Signals assigned to starting material **3**: δ 8.98 (s, 1H, H-2), 8.50 – 8.42 (m, 1H, H-6), 8.39 – 8.33 (m, 1H, H-4), 7.55 – 7.46 (m, 1H, H-5).[11] Relative to 1H of **23b**, 1H of **3** integrates for 0.30H.

^{13}C{^{1}H} NMR (100 MHz, CD_3CN)

Signals assigned to **23b**: δ 163.6 (C-6), 150.0 (C-2), 148.1 (C-4), 125.0 (C-5), 70.2 (O*C*H$_3$).

Signals assigned to **21b**: δ 151.8, 149.1, 140.2, 124.0, 46.6 (N*C*H$_3$).

Signals assigned to starting material **3**: δ 149.1 (C-2), 145.8 (C-4), 144.5 (C-6), 121.9 (C-5).

A quartet from $CF_3SO_3^-$ is present at δ 120.6 (partially overlaps with other signals; *J* = *ca.* 320 Hz).

Ratio of N-alkylation and O-alkylation Products (from integrations in ^{1}H NMR spectrum):

2H of Compound **23b** = 2.00 – Therefore 1H = 1.00

1H of Compound **21b** = 0.08 – Therefore 1H = 0.08

$$\text{Ratio} = \frac{1.00}{1.00 + 0.08} \times 100 = 93\%\ \text{O alkylation}$$

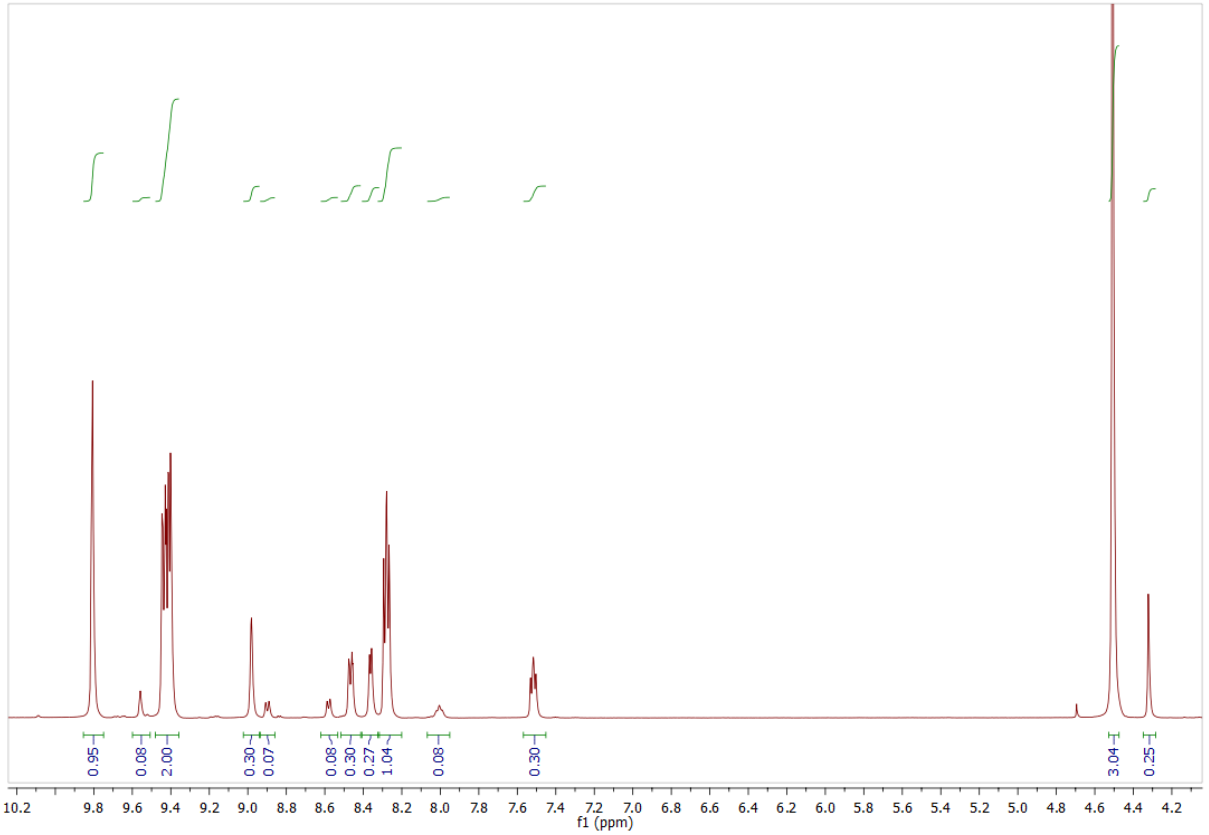

Figure S17: ^{1}H NMR spectrum in CD_3CN, containing signals assigned to **23b**, **21b** and **3**. The full spectrum is shown in Section 7.

The CD_3CN was removed and the product mixture was re-dissolved in $(CD_3)_2SO$ to allow a ^{1}H-^{15}N HMBC NMR spectrum to be measured. Product **21b** did not survive the solvent removal process.

^{1}H NMR (600 MHz, $(CD_3)_2SO$)

Signals assigned to **23b** δ 10.24 (dd, *J* = 2.2, 0.8 Hz, 1H, H-2), 9.89 (ddd, *J* = 6.8, 2.2, 1.6 Hz, 1H, H-6), 9.46 (dd, *J* = 4.8, 1.6 Hz, 1H, H-4), 8.40 (app. ddd, *J* = 6.8, 4.8, 0.8 Hz, 1H, H-5), 4.48 (s, 3H).

Signals assigned to starting material **3** δ 9.15 – 9.11 (m, 1H, H-2), 8.64 (ddd, *J* = 6.6, 2.0, 1.5 Hz, 1H, H-6), 8.38 (dd, *J* = 4.8, 1.5 Hz, 1H, H-4), 7.61 (ddd, *J* = 6.6, 4.8, 0.9 Hz, 1H, H-5).[11] Relative to 1H of **23b**, 1H of **3** integrates for approximately 1.80H.

^{15}N NMR (60.8 MHz, $(CD_3)_2SO$)

Signals assigned to **23b** δ 303.4 (free *N*), 249.4 (N^+—OMe)

Signals assigned to **3**: δ 300.9, 285.8.

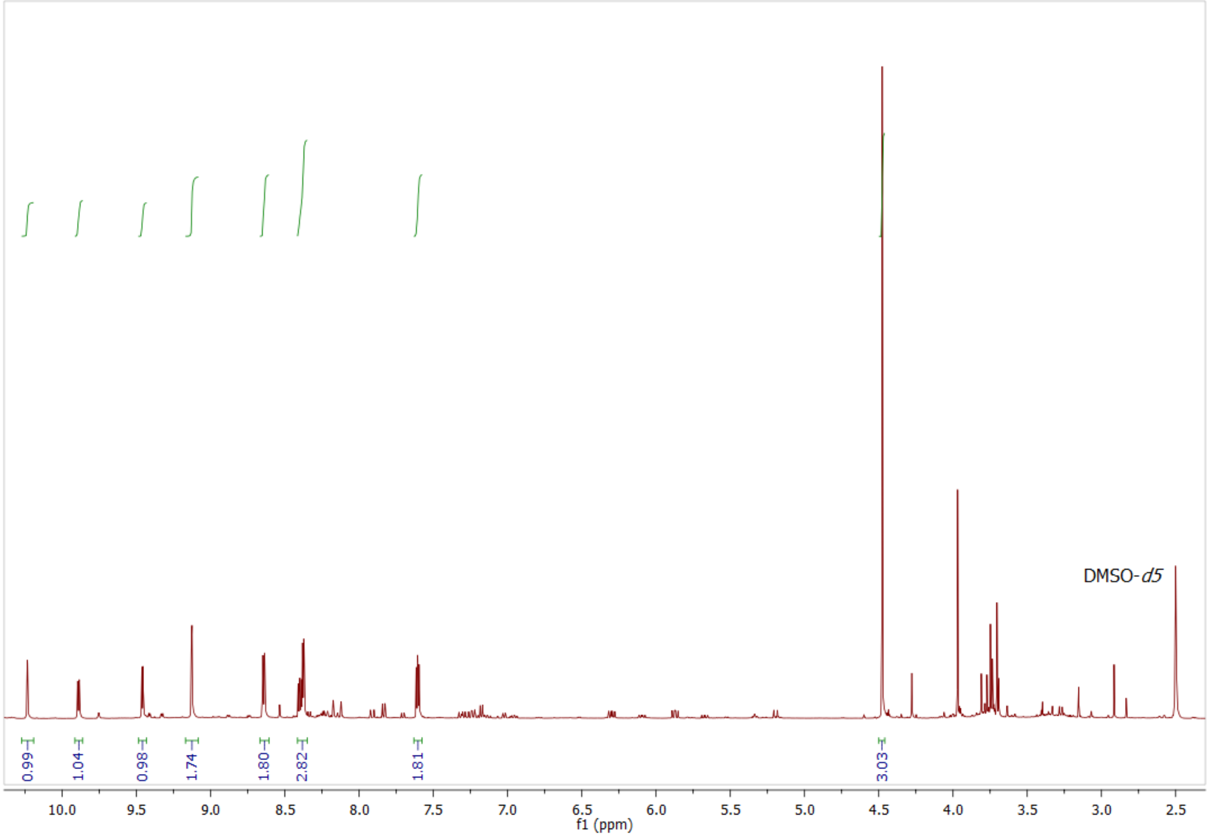

Figure S18: ^{1}H NMR spectrum in $(CD_3)_2SO$, containing signals assigned to **23b** and **3**. Signals assigned to **21b** are no longer present after solvent removal. Signals of a large amount of decomposition products are also present. The full spectrum is shown in Section 7.

(d) Experiment Showing N- vs O-Alkylation Product Ratio (21b vs 23b) in $(CD_3)_2SO$ – Contains ^{15}N and ^{13}C NMR Data

Pyrimidine *N*-oxide (**3**) (0.050 g, 0.52 mmol) was dissolved in $(CD_3)_2SO$ (0.8 ml) in a vial inside an inert atmosphere glove box. Methyl triflate (0.087 g, 0.53 mmol) was subsequently added dropwise. The reaction mixture was transferred to a NMR tube by syringe. The NMR tube was then sealed by a rubber septum cap and wrapped with PTFE tape. The septum was then covered with Parafilm and the tube transferred outside the glove box. ^{1}H NMR spectra were run periodically over the course of four weeks. Very slow consumption of **3** and growth of **21b** and **23b** was observed from these spectra. The integration of the ^{1}H NMR signal of the methylating agent (likely to be (methoxy)sulfonium triflate)[10] at δ 3.98 ppm also diminished during this time. After four weeks, ^{1}H, ^{13}C{^{1}H}, COSY, ^{1}H-^{13}C HSQC, ^{1}H-^{13}C HMBC and ^{1}H-^{15}N HMBC NMR spectra of the reaction mixture were recorded.

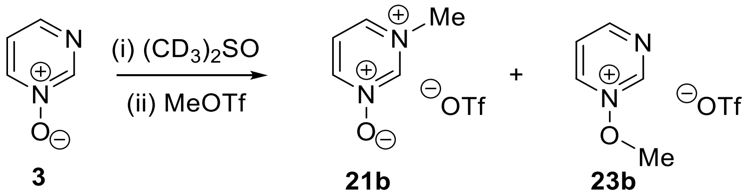

^{1}**H NMR** (600 MHz, $(CD_3)_2SO$)

Signals assigned to **23b**: δ 10.24 (dd, *J* = 2.2, 0.8 Hz, 1H, H-2, overlaps with signal of **21b**), 9.90 (ddd, *J* = 6.8, 2.2, 1.6 Hz, 1H, H-6), 9.47 (dd, *J* = 4.9, 1.6 Hz, 1H, H-4), 8.41 (ddd, *J* = 6.8, 4.9, 0.8 Hz, 1H, H-5, partially overlaps with signal of **3**), 4.48 (s, 3H, OCH_3).

Signals assigned to **21b**: δ 10.18 (s, 1H, overlaps with signal of **23b**), 9.21 – 9.17 (m, 1H), 8.89 (app d, app *J* = 6.1 Hz, 1H), 8.17 (app t, app *J* = 6.5 Hz, 1H), 4.26 (s, 3H, NCH_3). Relative to 1H of **23b**, 1H of **21b** integrates for 0.07H.

Signals assigned to **3**: δ 9.09 (m, 1H, H-2), 8.60 (ddd, *J* = 6.6, 2.0, 1.5 Hz, 1H, H-6), 8.33 (dd, *J* = 4.8, 1.5 Hz, 1H, H-4, partially overlaps with signal of **23b**), 7.58 (ddd, *J* = 6.6, 4.8, 1.0 Hz, 1H, H-5).[11] Relative to 1H of **23b**, 1H of **3** integrates for 0.61H.

A signal assigned to the methoxydimethylsulfonium salt of $(CD_3)_2SO$ is present at 3.98 ppm. Relative to 1H of **23b**, 1H of the salt integrates for approximately 0.33H.

Note: The singlet at δ 10.24 ppm has an extremely slow relaxation rate. A 30° pulse and a relaxation delay of 60 seconds were used during acquisition of the spectra above, leading to a set of internally consistent integrations for the ^{1}H NMR signals of **23b**. Use of a 90° pulse and a 60 second relaxation delay gave an integration of the signal at 10.24 ppm of 84% relative to the other 1H signals of **23b**.

^{13}**C{^{1}H} NMR** (151 MHz, $(CD_3)_2SO$)

Signals assigned to **23b**: δ 163.7 (C-6), 151.2 (C-2), 149.0 (C-4), 125.1 (C-5), 70.4 (OCH_3).

No signals in this ^{13}C NMR spectrum could be assigned to the small amount of **21b** present.

Signals assigned to **3**: δ 149.8 (C-2), 145.3 (C-4 or C-6), 145.2 (C-4 or C-6), 122.6 (C-5).

A signal at δ 121.1 (q, *J* = 322 Hz) is assigned to triflate ion ($^{-}OSO_2CF_3$). Small signal derived from decomposition products are also present (see spectra in section 7 and comment at start of section 4.4).

^{15}**N NMR** (60.8 MHz, $(CD_3)_2SO$)

Signals assigned to **23b**: δ 303.1 (free *N*), 249.0 (N^{+}—OMe)

Signals assigned to **21b**: δ 293.6 (*N*—O), 205.2 (N^{+}—Me)

Signals assigned to **3**: 300.7, 288.4.

<u>Conversion Calculation</u> (based on consumption of the methoxydimethylsulfonium salt as the limiting reagent):

3H of Compound **23b** corresponds to 3.00, therefore 1H = 1.00. 3H of compound **21b** corresponds to 0.21, therefore 1H = 0.07.

For the methoxydimethylsulfonium salt at 3.98 ppm, 3H = 1.00, therefore 1H = 0.33.

$$\text{Conversion} = \frac{1.00 + 0.07}{1.00 + 0.07 + 0.33} \times \ 100 = 76\%$$

Ratio of N-alkylation and O-alkylation Products (using integrations from ^{1}H NMR spectrum):

1H of Compound **23b** = 1.00

1H of Compound **21b** = 0.07

$$\text{Ratio} = \frac{1.00}{1.00 + 0.07} \times \ 100 = 93\% \text{ O alkylation}$$

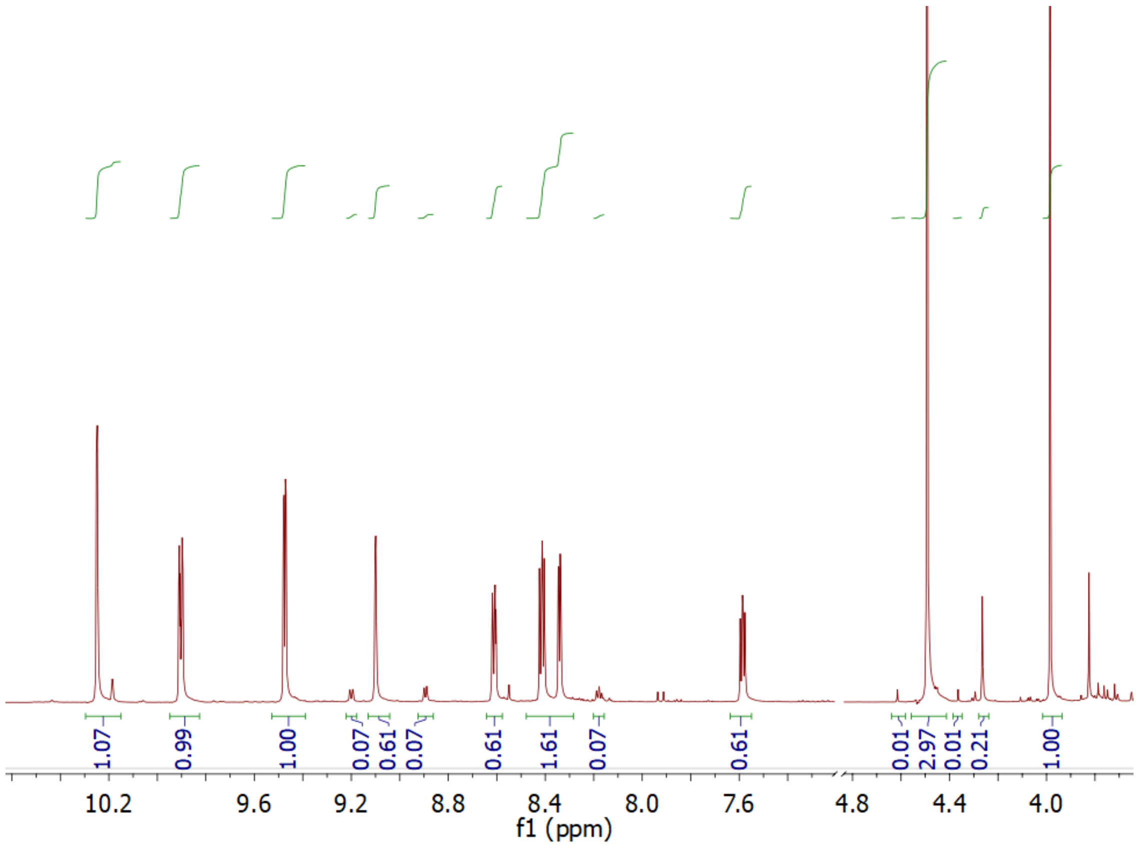

Figure S19: ^{1}H NMR spectrum in $(CD_3)_2SO$ of **23b**, containing signals assigned to **21b** and **3**. The full spectrum is shown in Section 7.

Attempted Preparation of 22 and 24

Pyrimidine *N*-oxide (**3**) (0.044 g, 0.46 mmol), benzhydryl chloride (0.093 g, 0.46 mmol) and silver triflate (0.132 g, 0.514 mmol) were combined by the process described in Procedure C in an attempt to produce **22** or **24** in CH_2Cl_2. The reaction mixture was analyzed by NMR spectroscopy using General Procedure B. As can be seen in the spectra shown below, the appearances of the signals are highly unusual, and none of these signals could be definitively assigned to any particular species. The identities of the entities formed in this process are not clear.

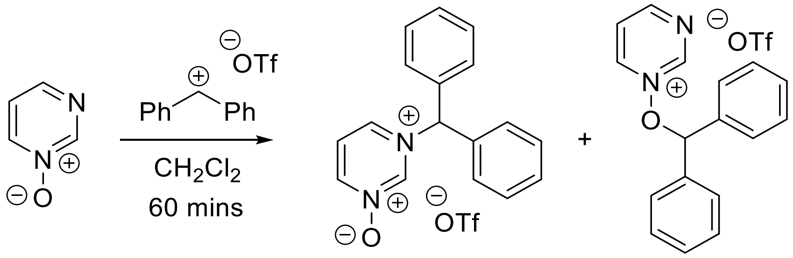

No evidence for product formation

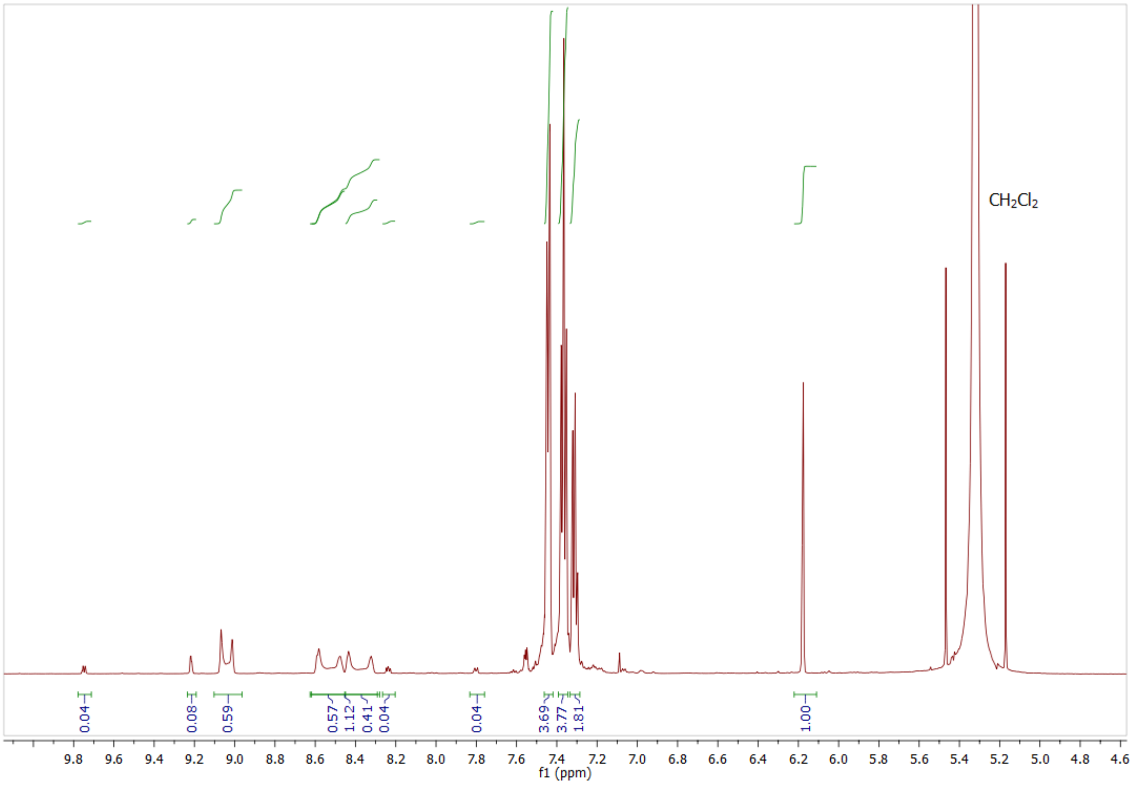

Figure S20: ^{1}H NMR spectrum in CH_2Cl_2 of the crude reaction mixture from the reaction above. Signals could not be definitively assigned to product **22** or **24**. The full spectrum is shown in Section 7.

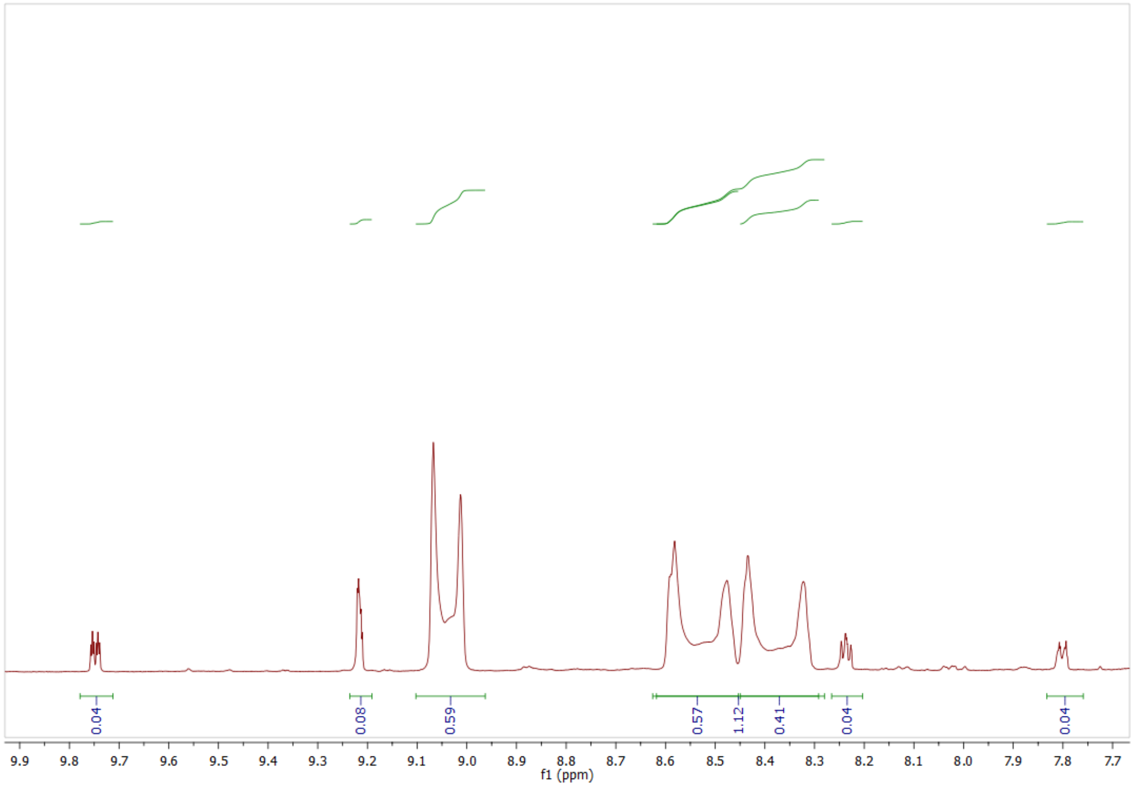

Figure S21: Expansion of the ^{1}H NMR spectrum of the crude reaction mixture from the reaction above in CH_2Cl_2, showing the broadness of the observed signals. Signals could not be definitively assigned to product **22** or **24**

5. Crossover Experiments

General Procedure D: Crossover experiments

The following procedure was used to establish whether reactions of diazine *N*-oxides occurred under kinetic control. In a glove box containing a nitrogen atmosphere, the appropriate diazine *N*-oxide (1 equivalent) was weighed into a vial. Dry CD_3CN (usually 0.65 ml) was added. An internal standard, 1,3,5-trimethoxybenzene was subsequently added (approx. 15 mol%). The mixture was then transferred into an NMR tube, which was sealed with a rubber septum. The seal was then wrapped with PTFE tape and Parafilm. Finally, the NMR tube was placed in a long Schlenk flask and removed from the glove box and brought to the NMR spectrometer. A ^{1}H NMR spectrum was measured and the tube was removed from the spectrometer. A solution of the crossover nucleophile in CD_3CN (amounts specified below) was then injected through the septum cap. A second ^{1}H NMR spectrum was recorded immediately, and an additional spectrum was obtained after allowing the reaction mixture to stand (in the NMR tube) for two days or more.

5.1 Crossover experiment – pyrazine *N*-oxide (1) with MeOTf and methyl nicotinate (25)

The following reagents were combined in the process described in General Procedure D. Pyrazine *N*-oxide (**1**) (0.018 g, 0.19 mmol) was dissolved in CD_3CN (0.65 ml) in a vial in a glove box. Methyl triflate (0.024 g, 0.15 mmol) was subsequently added dropwise. To this mixture was added 1,3,5-trimethoxybenzene (0.003 g, 0.02 mmol). The reaction mixture was transferred to an NMR tube and analyzed by NMR spectroscopy in CD_3CN (Spectrum A). The tube was removed from the spectrometer and methyl nicotinate (**25**) (0.032 g, 0.23 mmol) in CD_3CN (0.15 ml) was injected into the tube through the septum by syringe. The mixture was agitated and a second ^{1}H NMR spectrum was recorded. No change was observed in the ratio of **13b** and **15b** in this spectrum. An additional ^{1}H NMR spectrum was recorded after 1 day (Spectrum B).

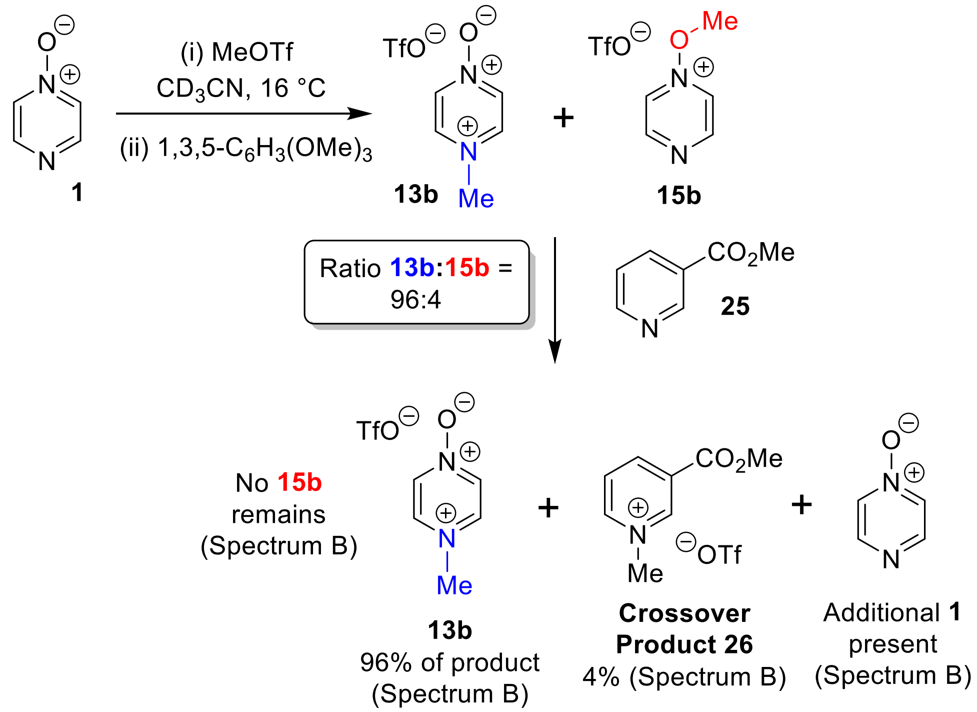

^{1}H NMR (300 MHz, CD_3CN, 15 second relaxation delay) Spectrum A:

Signals assigned to **13b**: δ 8.55 – 8.49 (m, 4H), 4.15 (s, 3H).

Signals assigned to **15b**: δ 9.45 (dd, J = 3.3, 1.6 Hz, 2H), 9.08 (dd, J = 3.3, 1.6 Hz, 2H), 4.51 (s, 3H). Relative to 1H of **13b**, 1H of **15b** integrates for 0.04H.

Signals assigned to starting material **1**: δ 8.47 – 8.40 (m, 2H), 8.13 (app dd, app J = 3.6, 1.5 Hz, 2H). Relative to 1H of **13b**, 1H of **1** integrates for approximately 0.28H.

Signals assigned to internal standard **trimethoxybenzene**: δ 6.09 (s, 3H), 3.74 (s, 9H). Relative to 1H of **23b**, 1H of **trimethoxybenzene** integrates for 0.14H.

Ratio of N-alkylation and O-alkylation Products:

3H of Compound **13b** = 3.00 – Therefore 1H = 1.00

2H of Compound **15b** = 0.08 – Therefore 1H = 0.04

$$\text{Ratio} = \frac{1.00}{1.00 + 0.04} \times \ 100 = 96\%\ \text{N alkylation}$$

Ratio of major product to internal standard:

3H of Compound **13b** = 3.00 – Therefore 1H = 1.00

3H of internal standard = 0.43 – Therefore 1H = 0.143

$$\text{Ratio} = \frac{1.00}{1.00 + 0.143} \times \ 100 = 87 : 13$$

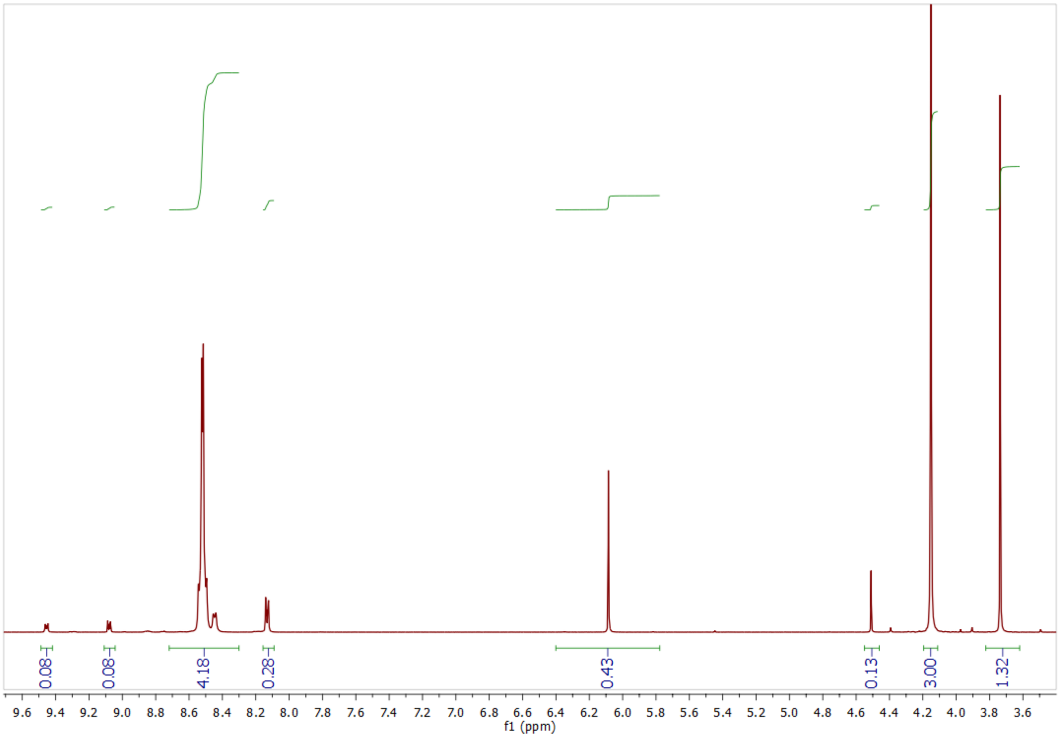

Figure S22: Spectrum A: ^{1}H NMR spectrum in CD_3CN containing signals assigned to **13b**, **15b** and **1**. Signals of the internal standard 1,3,5-trimethoxybenzene are also present. The full spectrum is shown in Section 7.

^{1}H NMR (300 MHz, CD_3CN, 15 second relaxation delay) Spectrum B:

Signals assigned to **13b**: δ 8.57 – 8.49 (m, 4H), 4.16 (s, 3H).

No signals assigned to **15b**

Signals assigned to starting material **1**: δ 8.46 – 8.38 (m, 2H), 8.08 (app dd, app *J* = 3.5, 1.5 Hz, 2H). Relative to 1H of **13b**, 1H of **1** integrates for approximately 0.39H.

Signals assigned to internal standard **trimethoxybenzene**: δ 6.08 (s, 1H), 3.73 (s, 1H). Relative to 1H of **13b**, 1H of **trimethoxybenzene** integrates for approximately 0.15H.

Signals assigned to **25**: δ 9.14 – 9.10 (m, 1H), 8.76 (dd, *J* = 4.9, 1.7 Hz, 1H), 8.30 – 8.25 (m, 1H), 7.47 (ddd, *J* = 8.0, 4.9, 0.9 Hz, 1H), 3.90 (s, 3H). Relative to 1H of **13b**, 1H of **3** integrates for 1.57H.

Signals assigned to **crossover product 26**: δ 9.22 (s, 1H), 8.92 (d, *J* = 8.1 Hz, 1H), 4.39 (s, 3H), 4.00 (s, 3H). Relative to 1H of **13b**, 1H of **26** integrates for 0.04H. [12]

Note: The singlets at 4.39 ppm and 4.16 ppm are overlapping with a minor side product, altering their integration values.

Ratio of **13b** to **crossover product 26**:
4H of Compound **13b** = 4.00 – Therefore 1H = 1.00

1H of **26** = 0.04 – Therefore 1H = 0.04

$$\text{Ratio} = \frac{1.00}{1.00 + 0.04} \times \ 100 = 96 : 4$$

Ratio of major product to internal standard:

4H of Compound **13b** = 4.00 – Therefore 1H = 1.00

3H of internal standard = 0.45 – Therefore 1H = 0.15

$$\text{Ratio} = \frac{1.00}{1.00 + 0.15} \times \ 100 = 87 : 13$$

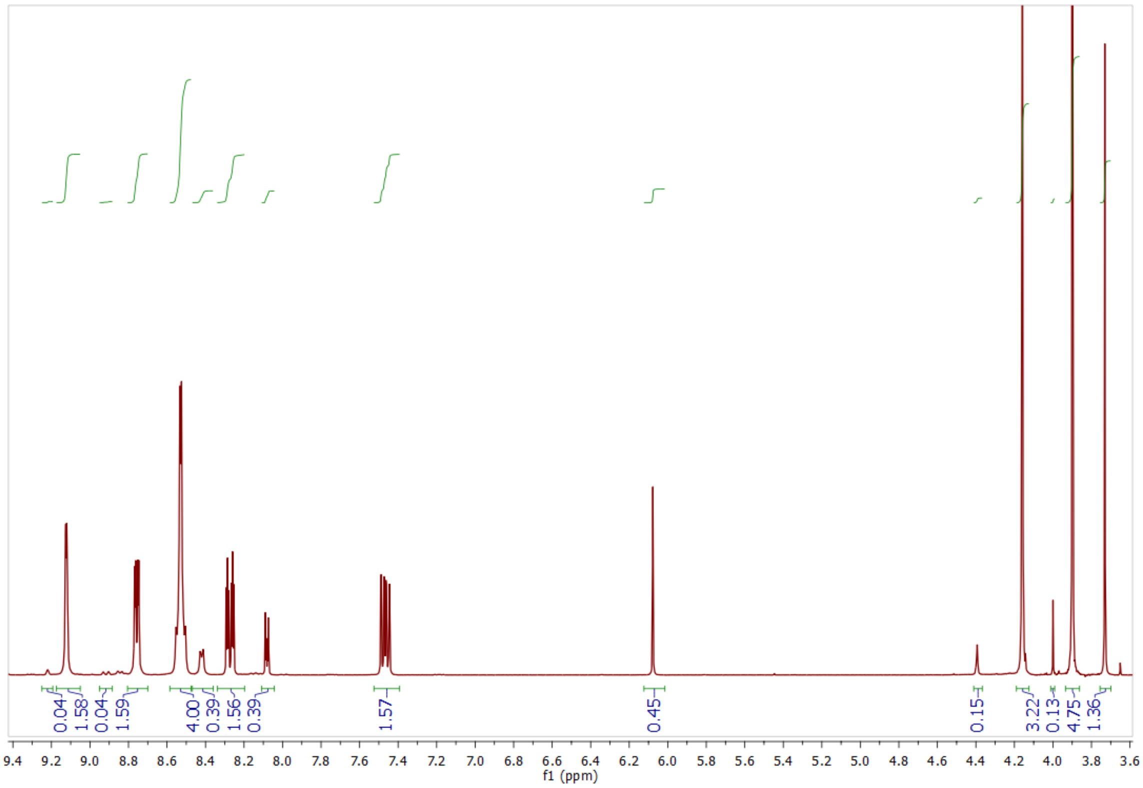

Figure S23: Spectrum B: ^{1}H NMR spectrum in CD_3CN containing signals assigned to **13b**, **25**, crossover product **26** and **1**. Signals of the internal standard 1,3,5-trimethoxybenzene are also present. No signals assigned to **15b** are observed. The full spectrum is shown in Section 7.

That the relative ratio of N-methylation product (**13b**) and the internal standard (1,3,5-trimethoxybenzene) remains constant after addition of 2nd nucleophile (**25**) demonstrates that the formation of **13b** from **1** + MeOTf is irreversible under the reaction conditions employed.

We conclude that formation of crossover product (**26**) derived from O-methylation product **15b** occurs by S_N2 reaction of **15b** + 2nd nucleophile **25**, and that **15b** does not undergo reversal to **1** + MeOTf in CD_3CN at *ca.* 20 °C (i.e. **15b** is formed irreversibly). If this were not the case, then a mixture of **13b** + **15b** should eventually convert entirely to **13b**, since **13b** is formed irreversibly. The ratio of **13b** to **15b** remains invariant with time unless a second nucleophile is added to the reaction mixture.

5.2 Crossover experiment – quinoxaline *N*-oxide (2) with MeOTf and pyrazine (7)

The following reagents were combined in the process described in General Procedure D. Quinoxaline *N*-oxide (**2**) (0.018 g, 0.12 mmol) was dissolved in CD_3CN (0.65 ml) in a vial in a glove box. Methyl triflate (0.019 g, 0.12 mmol) was subsequently added dropwise. To this mixture was added 1,3,5-trimethoxybenzene (5 mg, 0.03 mmol). The reaction mixture was transferred to an NMR tube and analyzed by NMR spectroscopy in CD_3CN (Spectrum A). The tube was removed from the spectrometer and a solution of **7** (8 mg, 0.010 mmol) in CD_3CN (0.20 ml) was injected into the tube through the rubber septum by syringe. The mixture was agitated and a second ^1H NMR spectrum was recorded. No change was observed in the ratio of **17b** and **19b** in this spectrum. After 1 day, and additional ^1H NMR spectrum was recorded (Spectrum B).

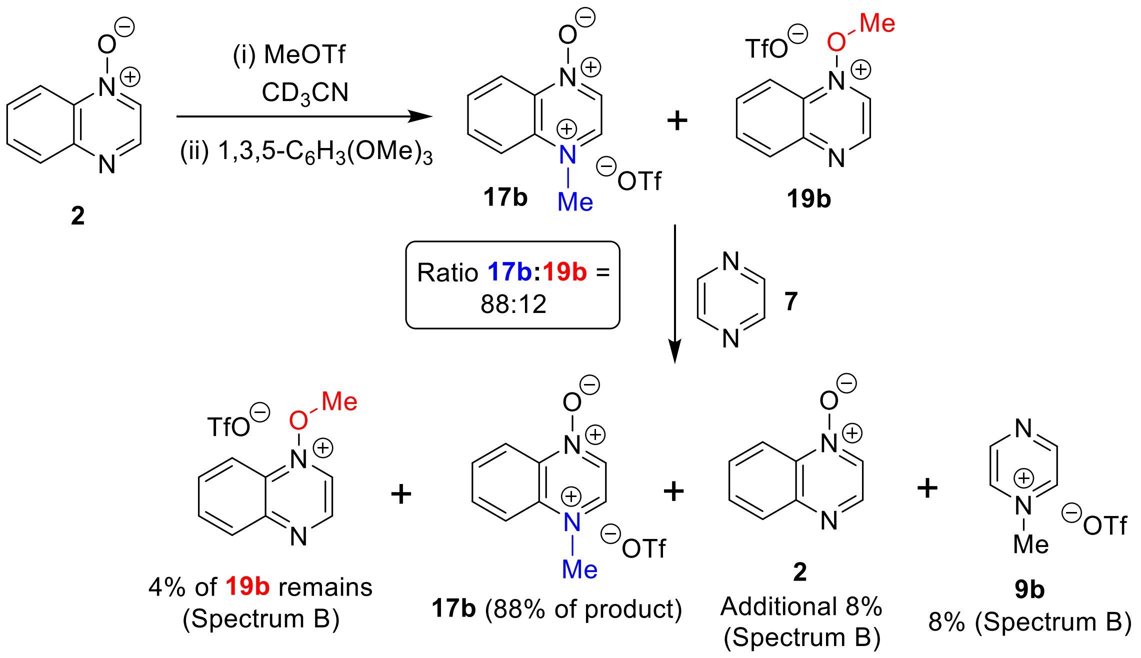

^{1}H NMR (300 MHz, CD_3CN, 15 second relaxation delay) Spectrum A:

Signals assigned to **17b**: δ 8.92 (d, *J* = 5.3 Hz, 1H), 8.75 (d, *J* = 5.3 Hz, 1H), 8.62 – 8.55 (m, overlaps with 1H of **19b**, contains 1H of **17b**), 4.43 (s, 3H).

Signals assigned to **19b**: δ 9.60 (d, *J* = 3.4 Hz, 1H), 9.49 (d, *J* = 3.4 Hz, 1H), 8.56 – 8.52 (overlaps with 1H of **17b**, contains 1H of **19b**), 4.65 (s, 3H). Relative to 1H of **17b**, 1H of **19b** integrates for 0.13H.

Signals assigned to starting material **2**: δ 8.67 (d, *J* = 3.6 Hz, 1H), 8.46 (dd, *J* = 8.6, 1.4 Hz, 1H), 8.38 – 8.23 (m, 1H), 7.92–7.84 (m, 1H), 7.83 – 7.75 (m, 1H). Relative to 1H of **17b**, 1H of **2** integrates for approximately 0.38H.

Signals assigned to internal standard **trimethoxybenzene**: δ 6.06 (s, 3H), 3.73 (s, 9H). Relative to 1H of **23b**, 1H of **trimethoxybenzene** integrates for 0.33H.

The section of the spectrum at 8.62 – 8.53 ppm contains 1H each of **17b** and **19b**. The section at 8.38 – 8.23 ppm contains a 2H signal from **17b**, a 2H signal from **19b** and a 1H signal from **2**. The section at 8.15 – 8.07 ppm contains a 1H signal from **17b,** a 1H signal from **19b** and a 1H signal from **2**.

Ratio of N-alkylation and O-alkylation Products:

1H of Compound **17b** = 1.00

1H of Compound **19b** = 0.13

$$\text{Ratio} = \frac{1.00}{1.00 + 0.13} \times\ 100 = 88\%\ \text{N alkylation}$$

Ratio of major product to internal standard:

1H of Compound **17b** = 1.00

3H of internal standard = 1.00 – Therefore 1H = 0.33

$$\text{Ratio} = \frac{1.00}{1.00 + 0.33} \times\ 100 = 75 : 25$$

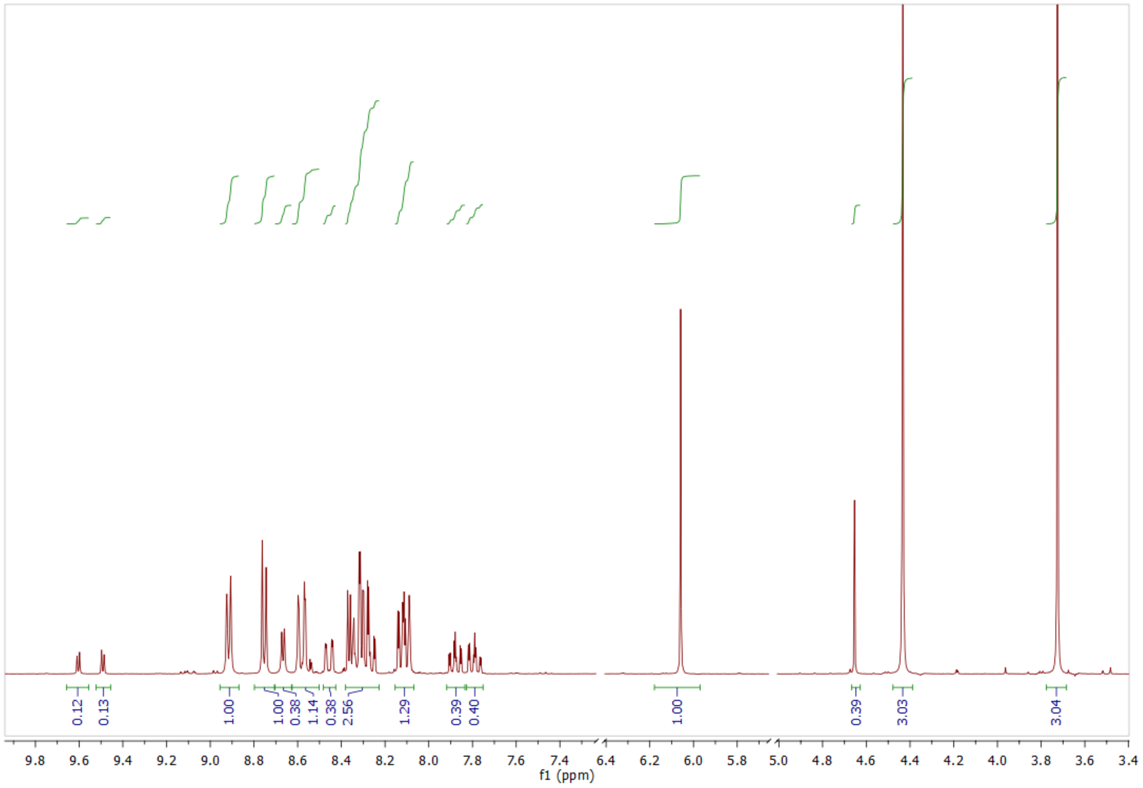

Figure S24: Spectrum A: ^{1}H NMR spectrum in CD_3CN containing signals assigned to **17b**, **19b** and **2**. Signals of the internal standard 1,3,5-trimethoxybenzene are also present. The full spectrum is shown in Section 7.

^{1}H NMR (300 MHz, CD_3CN, 15 second relaxation delay) Spectrum B:

Signals assigned to **17b**: δ 8.91 (d, *J* = 5.3 Hz, 1H), 8.76 (d, *J* = 5.3 Hz, overlaps with signal of **9b**, contains 1H of **17b**), 4.43 (s, 3H).

Signals assigned to **19b**: δ 9.61 (d, *J* = 3.4 Hz, 1H), 9.49 (d, *J* = 3.4 Hz, 1H), 8.58 (app d, app *J* = 7.3 Hz, 1H), 4.65 (s, 3H). Relative to 1H of **17b**, 1H of **19b** integrates for 0.05H.

Signals assigned to starting material **2**: δ 8.66 (d, *J* = 3.6 Hz, 1H), 8.46 (dd, *J* = 8.6, 1.4 Hz, 1H), 7.91–7.83 (m, 1H), 7.82 – 7.74 (m, 1H). Relative to 1H of **17b**, 1H of **2** integrates for approximately 0.55H.

Signals assigned to internal standard **trimethoxybenzene**: δ 6.06 (s, 3H), 3.73 (s, 9H). Relative to 1H of **17b**, 1H of **trimethoxybenzene** integrates for approximately 0.33H.

Signals assigned to **7**: δ 8.57 (s, overlaps with 1H signal of **17b**, contains 4H of **7** (relative integration = 4.05 – 1.00 = 3.05)). Relative to 1H of **17b**, 1H of **7** integrates for (3.05/4) = 0.76H.

Signals assigned to **crossover product 9b**: δ 9.41 – 9.35 (m, 2H), 8.77 – 8.73 (m, 2H, overlaps with signal of **17b**), 4.39 (s, 3H). Relative to 1H of **17b**, 1H of **9b** integrates for approximately 0.07H.

The section of the spectrum between 8.38 and 8.23 ppm contains a 2H signal from **17b**, a 2H signal from **19b** and a 1H signal from **2**. The section at 8.15 – 8.07 ppm contains a 1H signal from **17b,** a 1H signal from **19b** and a 1H signal from **2**. The signal between 8.77 and 8.71 ppm contains a 1H signal from **17b** and a 1H signal from **19b**.

Ratio of **17b** to **crossover product 9b**:

1H of Compound **17b** = 1.00

2H of crossover product = 0.14 – Therefore 1H = 0.07

$$\text{Ratio} = \frac{1.00}{1.00 + 0.07} \times \ 100 = 93 : 7$$

Ratio of major product to internal standard:

1H of Compound **17b** = 1.02

3H of internal standard = 1.00 – Therefore 1H = 0.33

$$\text{Ratio} = \frac{1.02}{1.02 + 0.33} \times \ 100 = 76 : 24$$

That the relative ratio of N-methylation product (**17b**) and the internal standard (1,3,5-trimethoxybenzene) remains constant after addition of 2nd nucleophile (**7**) demonstrates that the formation of **17b** from **2** + MeOTf is irreversible under the reaction conditions employed.

We conclude that formation of crossover product (**9b**) derived from O-methylation product **19b** occurs by S_N2 reaction of **19b** + 2nd nucleophile **7**, and that **19b** does not undergo reversal to **2** + MeOTf in CD_3CN at *ca.* 20 °C (i.e. **19b** is formed irreversibly). If this were not the case, then a mixture of **17b** + **19b** should eventually convert entirely to **17b**, since **17b** is formed irreversibly. The ratio of **17b** to **19b** remains invariant with time unless a second nucleophile is added to the reaction mixture.

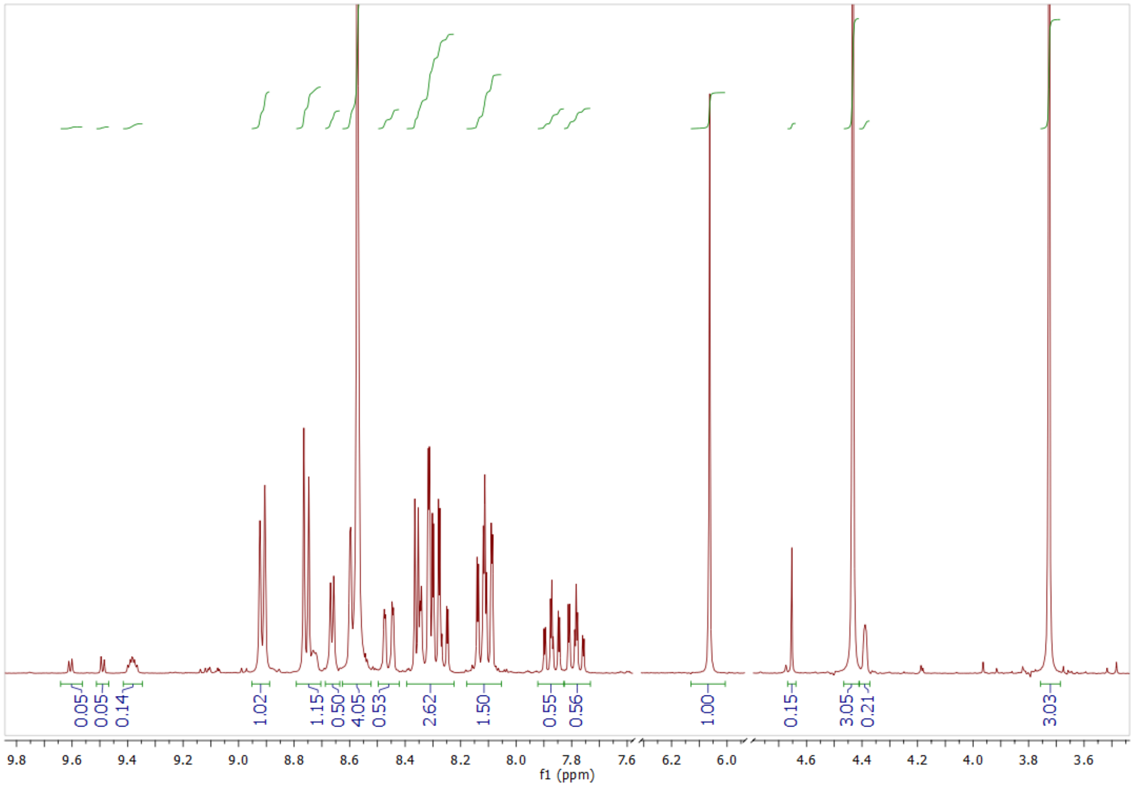

Figure S25: Spectrum B: ^{1}H NMR spectrum in CD_3CN containing signals assigned to **17b**, **7**, crossover product **9b** and **2**. Signals of the internal standard 1,3,5-trimethoxybenzene are also present. A lower proportion of signals assigned to **19b** are observed. The full spectrum is shown in Section 7.

5.3 Crossover experiment – pyrimidine *N*-oxide (3) with MeOTf and pyrazine (7)

The following reagents were combined in the process described in General Procedure D. Pyrimidine *N*-oxide (**3**) (0.014 g, 0.15 mmol) was dissolved in CD_3CN (0.65 ml) in a vial in a glove box. Methyl triflate (0.021 g, 0.13 mmol) was subsequently added dropwise. To this mixture was added 1,3,5-trimethoxybenzene (4 mg, 0.02 mmol). The reaction mixture was transferred to an NMR tube and analyzed by NMR spectroscopy in CD_3CN (Spectrum A). The tube was removed from the spectrometer and a 1.33 mol L^{-1} solution of **7** in CD_3CN (0.25 ml, 0.33 mmol) was injected into the tube through the rubber septum by syringe. The mixture was agitated and a second ^{1}H NMR spectrum was recorded – no change was observed in the ratio of **21b** and **23b** in this spectrum. After 1 day, the mixture was re-analyzed by ^{1}H NMR spectroscopy (Spectrum B). In spectrum B, only signals of starting material **3**, crossover product **9b**, **7** and **trimethoxybenzene** were observed. After two weeks, the mixture was analyzed again by ^{1}H NMR spectroscopy (Spectrum C). This showed that no **21b** or **23b** remained, and substantial formation of crossover product **9b** along with starting material **3** and a variety of decomposition products (the latter of which have been observed in all other experiments involving formation of **21b** and **23b** – see above).

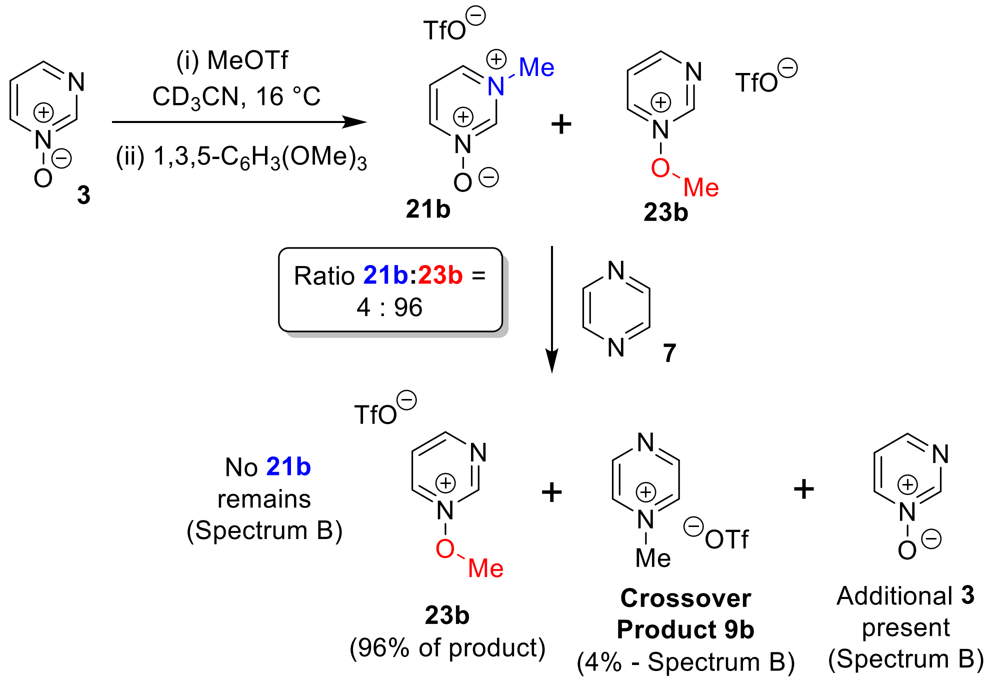

^{1}H NMR (300 MHz, CD_3CN, 20 second relaxation delay) <u>Spectrum A</u>:

Signals assigned to **21b**: δ 9.50 – 9.36 (m, 1H), 8.98 – 8.85 (m, 1H), 8.53 (d, *J* = 6.0 Hz, 1H), 8.13 (s, 1H), 4.30 (s, 3H).

Signals assigned to **23b**: δ 9.78 – 9.77 (m, 1H), 9.43 – 9.36 (m, 2H), 8.33 – 8.17 (m, 1H), 4.51 (s, 3H). Relative to 1H of **23b**, 1H of **21b** integrates for 0.03H.

Signals assigned to starting material **3**: δ 8.95 (s, 1H), 8.41 (m, 1H), 8.34 – 8.23 (m, 1H), 7.51 – 7.43 (m, 1H). Relative to 1H of **23b**, 1H of **3** integrates for 0.31H.

Signals assigned to internal standard **trimethoxybenzene**: δ 6.11 (s, 3H), 3.76 (s, 9H). Relative to 1H of **23b**, 1H of **trimethoxybenzene** integrates for 0.18H.

The section of the spectrum between 9.50 and 9.36 ppm contains a 1H signal from **23b** and a 2H signal from **21b**. The section between 8.98 and 8.85 ppm contains a 1H signal from **21b** and a 1H signal from **3**. The section between 8.41 and 8.23 ppm contains a 1H signal from **23b** and two 1H signals from **3**.

Ratio of N-alkylation and O-alkylation Products:

3H of Compound **23b** = 3.00 – Therefore 1H = 1.00

1H of Compound **21b** = 0.03

$$\text{Ratio} = \frac{1.00}{1.00 + 0.03} \times \ 100 = 97\% \text{ O alkylation}$$

Ratio of major product to internal standard:

3H of Compound **23b** = 3.00 – Therefore 1H = 1.00

3H of internal standard = 0.54 – Therefore 1H = 0.18

$$\text{Ratio} = \frac{1.00}{1.00 + 0.18} \times \ 100 = 85 : 15$$

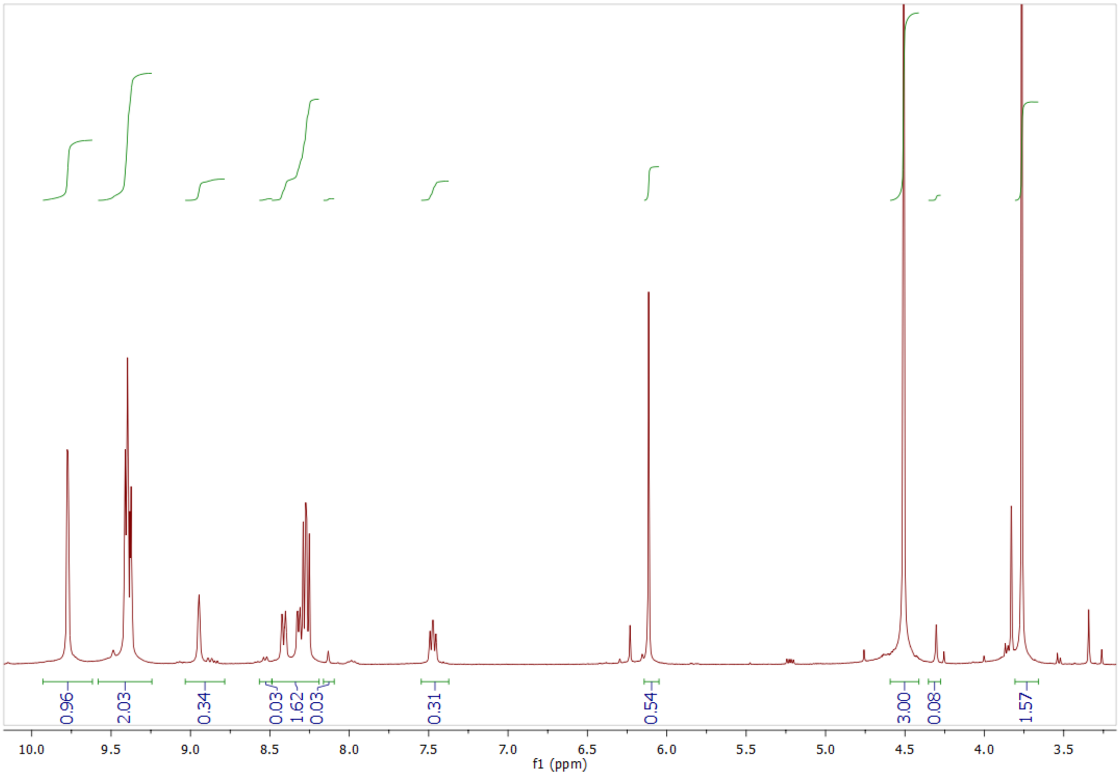

Figure S26: Spectrum A: ^{1}H NMR spectrum in CD_3CN containing signals assigned to **21b**, **23b** and **3**. Signals of the internal standard 1,3,5-trimethoxybenzene are also present. The full spectrum is shown in Section 7.

[1]**H NMR** (300 MHz, CD_3CN, 20 second relaxation delay) Spectrum B:

No signals assigned to **21b**.

Signals assigned to **23b**: δ 9.78 (app dd, app *J* = 2.2, 0.7 Hz, 1H), 9.43 – 9.37 (m, 2H), 8.31 – 8.25 (m, 1H), 4.51 (s, 3H).

Signals assigned to starting material **3**: δ 8.93 (s, 1H), 8.42 – 8.36 (m, 1H), 8.31 – 8.25 (m, 1H), 7.48 – 7.40 (m, 1H). Relative to 1H of **23b**, 1H of **3** integrates for 0.43H.

Signals assigned to internal standard **trimethoxybenzene**: δ 6.11 (s, 3H), 3.76 (s, 9H). Relative to 1H of **23b**, 1H of **trimethoxybenzene** integrates for 0.18H.

Signals assigned to **7**: δ 8.60 (s, 4H). Relative to 1H of **23b**, 1H of **7** integrates for (11.32/4) = 2.83H.

Signals assigned to **crossover product 9b**: δ 9.43 – 9.37 (m, 2H), 8.76 (d, *J* = 3.1 Hz, 2H), 4.42 (s, 3H). Relative to 1H of **23b**, 1H of **9b** integrates for 0.10H.

The signal between 9.50 and 9.36 ppm contains a 1H signal from **21b** and a 2H signal from **23b**. The signal between 8.98 and 8.85 ppm contains a 1H signal from **21b** and a 1H signal from **3**. The signal between 8.31 and 8.25 ppm contains a 1H signal from **23b** and a 1H signals from **3**.

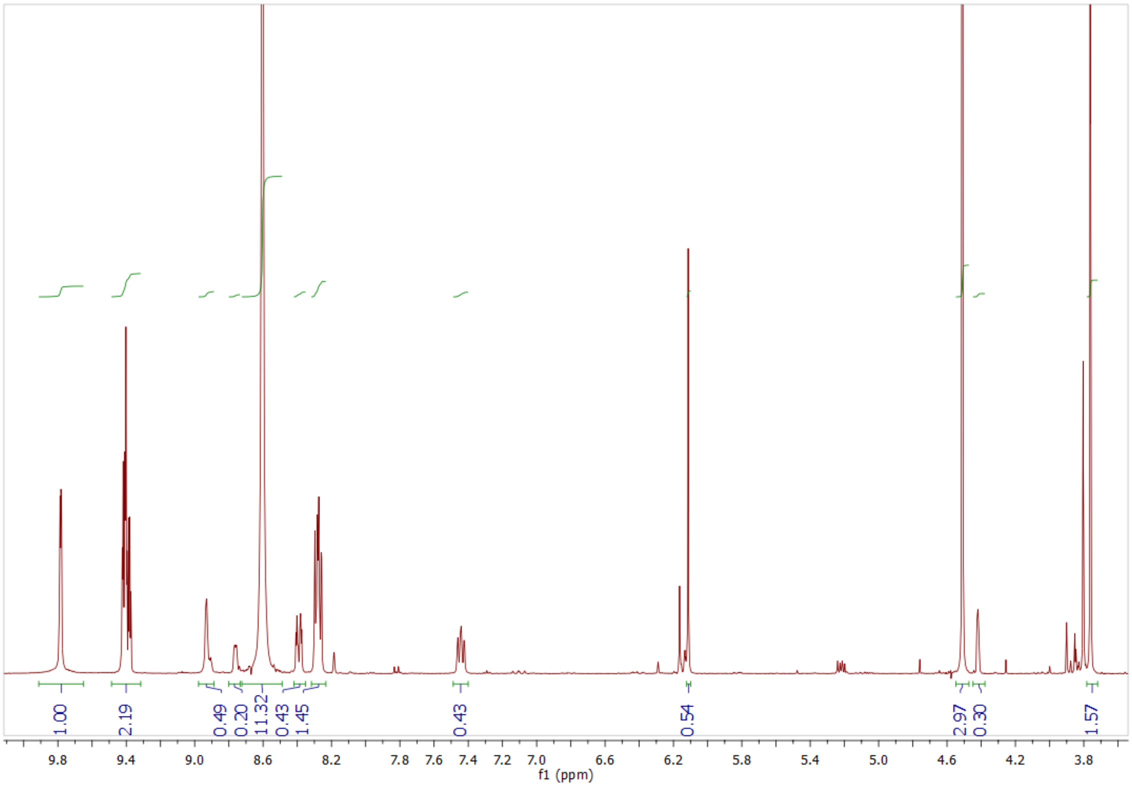

Figure S27: Spectrum B: ^{1}H NMR spectrum in CD_3CN containing signals assigned to **23b**, **7**, crossover product **9b** and **3**. Signals of the internal standard 1,3,5-trimethoxybenzene are also present. No signals of **21b** can be observed. The full spectrum is shown in Section 7.

Ratio of **23b** to **crossover product 9b**:

1H of Compound **23b** = 1.00

2H of crossover product = 0.20 – Therefore 1H = 0.10

$$\text{Ratio} = \frac{1.00}{1.00 + 0.10} \times \ 100 = 91 : 9$$

Ratio of major product to internal standard:

1H of Compound **23b** = 1.00

3H of internal standard = 0.54 – Therefore 1H = 0.18

$$\text{Ratio} = \frac{1.00}{1.00 + 0.18} \times \ 100 = 85 : 15$$

^{1}H NMR (400 MHz, CD_3CN, 20 second relaxation delay) Spectrum C: Relative integrations are given relative to 1H of crossover product **9b** since no baseline-separated signals of the internal standard are available.

No signals characteristic of **23b** are present.

Signals assigned to starting material **3**: δ 9.02 (s, 1H), 8.52 – 8.46 (m, 1H), 8.44 – 8.36 (m, 1H), 7.57 – 7.49 (m, 1H). Relative to 1H of **9b**, 1H of **3** integrates for 1.45H.

Signals assigned to **7**: δ 8.61 (s, 4H). Relative to 1H of **9b**, 1H of **7** integrates for 3.02H.

Signals assigned to **crossover product 9b**: δ 9.43 – 9.35 (m, 2H), 8.75 – 8.70 (m, 2H), 4.38 (s, 3H).

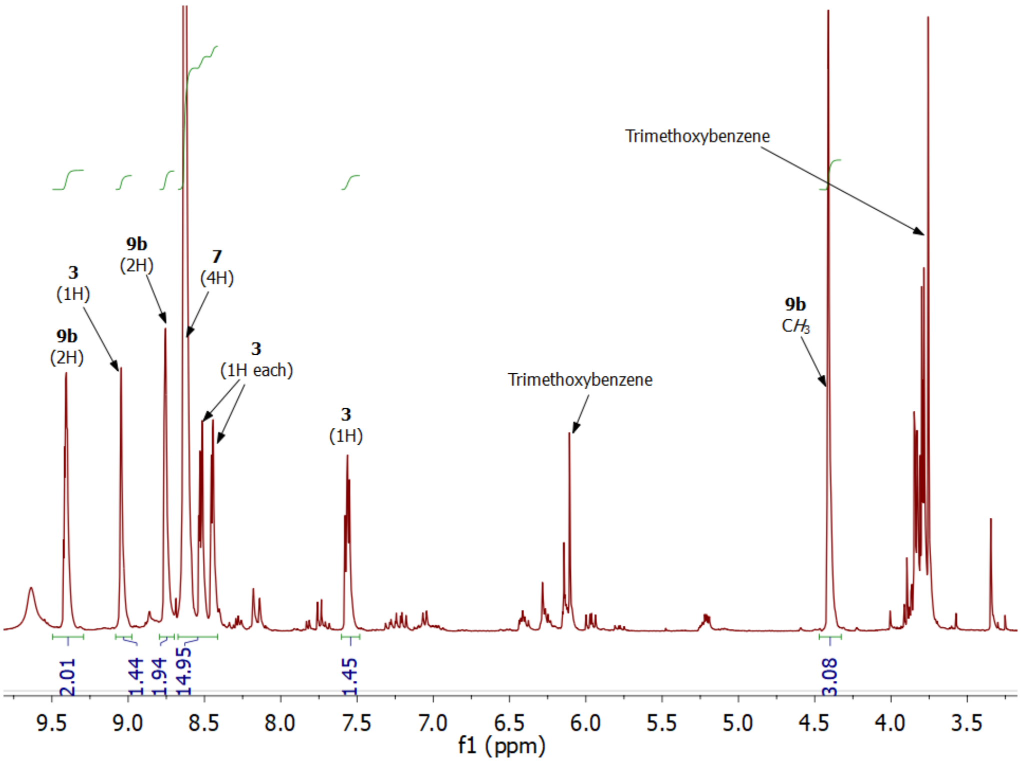

Figure S28: Spectrum C: ^{1}H NMR spectrum in CD_3CN containing signals assigned to **7**, crossover product **9b** and starting material **3**. Signals of the internal standard, 1,3,5-trimethoxybenzene, are also present, but are obscured by signals of decomposition products. No signals assigned to **23b** are observed. The full spectrum is shown in Section 7.

The signals of the internal standard (1,3,5-trimethoxybenzene) are obscured by signals of decomposition products (see ^{1}H NMR spectrum below). The relative proportion of (**7** + **9b**) to **3** is similar to the relative proportion of (**7** + **9b**) to (**3** + **23b**) in spectrum B (above), but reflects the occurrence of some decomposition of **23b** that was independent of the process of formation of crossover product **9b** by methylation of **7**.

Formation of crossover product (**9b**) derived from both N-methylation and O-methylation products (**21b** and **23b**) may indicate that **21b** and **23b** form reversibly from **3** + MeOTf, or instead that **21b** and **23b** each undergo S_N2 reactions with 2nd nucleophile **7**.

5.4 Crossover experiment – 4-methylpyrazinium-*N*-oxide iodide (13a) with MeOTf and methyl nicotinate (25)

The following reagents were combined in the process described in General Procedure D. **13a** (0.031 g, 0.13 mmol) was dissolved in $(CD_3)_2SO$ (0.7 ml) in a vial. To this solution was added 1,3,5-trimethoxybenzene (3 mg, 0.02 mmol). The mixture was transferred to an NMR tube and analyzed by NMR spectroscopy in $(CD_3)_2SO$ (Spectrum A). The tube was removed from the spectrometer and a solution of methyl nicotinate (**25**) (0.026 g, 0.19 mmol) in $(CD_3)_2SO$ (0.15 ml) was injected into the tube through the septum by syringe. The mixture was agitated and re-analyzed by NMR spectroscopy immediately, and again after one day (Spectrum B).

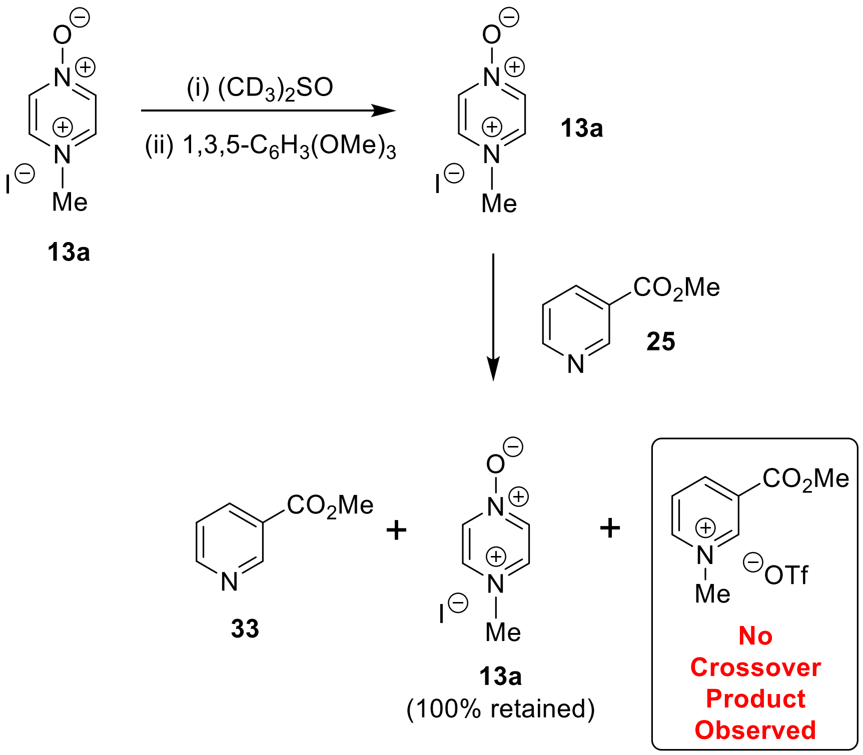

^{1}H NMR (300 MHz, $(CD_3)_2SO$, 10 second relaxation delay) Spectrum A:

Signals assigned to **13a**: δ 8.92 (dd, *J* = 13.3, 5.7 Hz, 4H), 4.16 (s, 3H).

Signals assigned to internal standard **trimethoxybenzene**: δ 6.06 (s, 3H), 3.68 (s, 9H). Relative to 1H of **13a**, 1H of **trimethoxybenzene** integrates for 0.18H.

Note: A singlet belonging to a small amount of an unknown contaminant is present at 8.22 ppm.

The singlet at 3.68 ppm could not be accurately integrated due to its proximity to the H_2O signal.

Ratio of major product to internal standard:

4H of Compound **13a** = 4.00 – Therefore 1H = 1.00

3H of internal standard = 0.55 – Therefore 1H = 0.18

$$\text{Ratio} = \frac{1.00}{1.00 + 0.18} \times \ 100 = 85 : 15$$

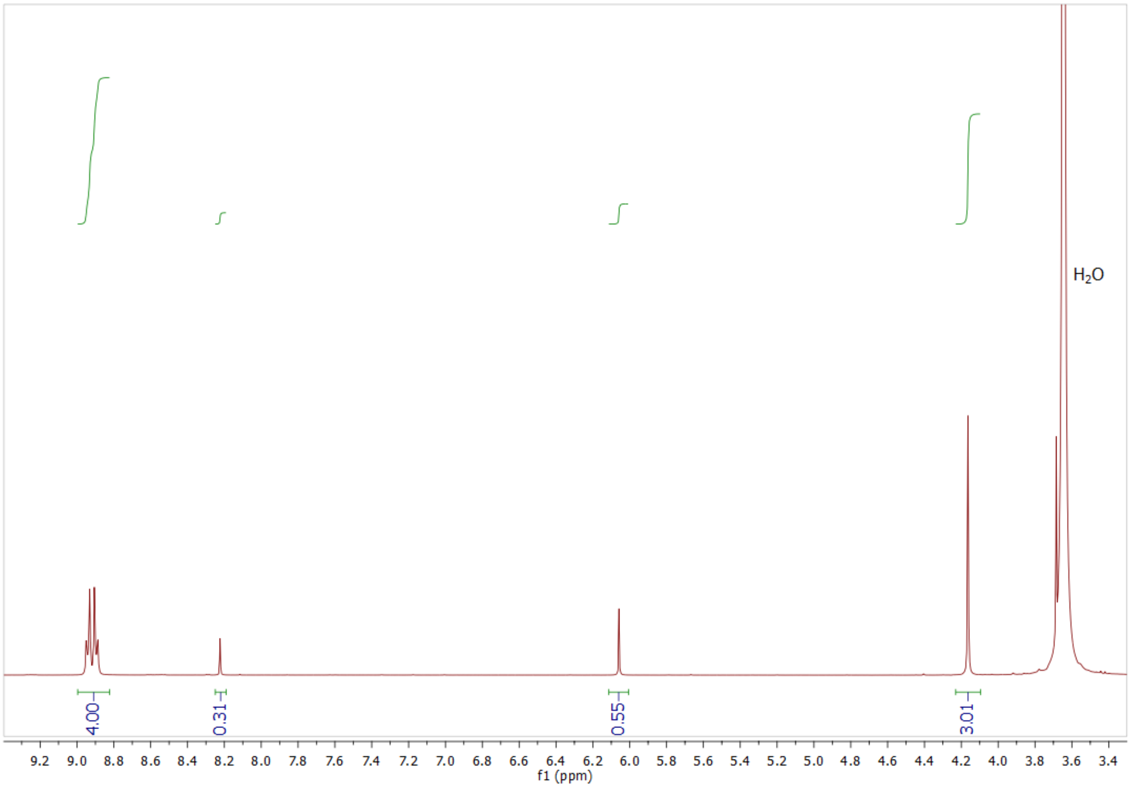

Figure S29: Spectrum A: ^{1}H NMR spectrum in $(CD_3)_2SO$ containing signals assigned to **13a**. Signals of the internal standard 1,3,5-trimethoxybenzene are also present. The full spectrum is shown in Section 7.

^{1}H NMR (300 MHz, $(CD_3)_2SO$, 10 second relaxation delay) Spectrum B:

Signals assigned to **13a**: δ 8.98 – 8.90 (m, 4H), 4.17 (s, 3H).

Signals assigned to internal standard **trimethoxybenzene**: δ 6.04 (s, 3H). Relative to 1H of **13a**, 1H of **trimethoxybenzene** integrates for 0.18H.

Signals assigned to **25**: δ 9.04 (d, *J* = 1.7 Hz, 1H), 8.78 (dd, *J* = 4.8, 1.6 Hz, 1H), 8.27 (app dt, app *J* = 8.0, 1.9 Hz, 1H), 7.56 (ddd, *J* = 8.0, 4.9 Hz (signal resolution not sufficient to determine smallest *J* value – it is of the order of < 1 Hz), 1H), 3.86 (s, 3H). Relative to 1H of **23b**, 1H of **25** integrates for 1.59H.

Note: The 9H singlet of 1,3,5-trimethoxybenzene is obscured by the signal of residual H_2O.

Ratio of major product to internal standard:

4H of Compound **13a** = 4.00 – Therefore 1H = 1.00

3H of internal standard = 0.54 – Therefore 1H = 0.18

$$\text{Ratio} = \frac{1.00}{1.00 + 0.18} \times \ 100 = 85 : 15$$

This experiment shows that **13a** is formed irreversibly from **1** + MeI.

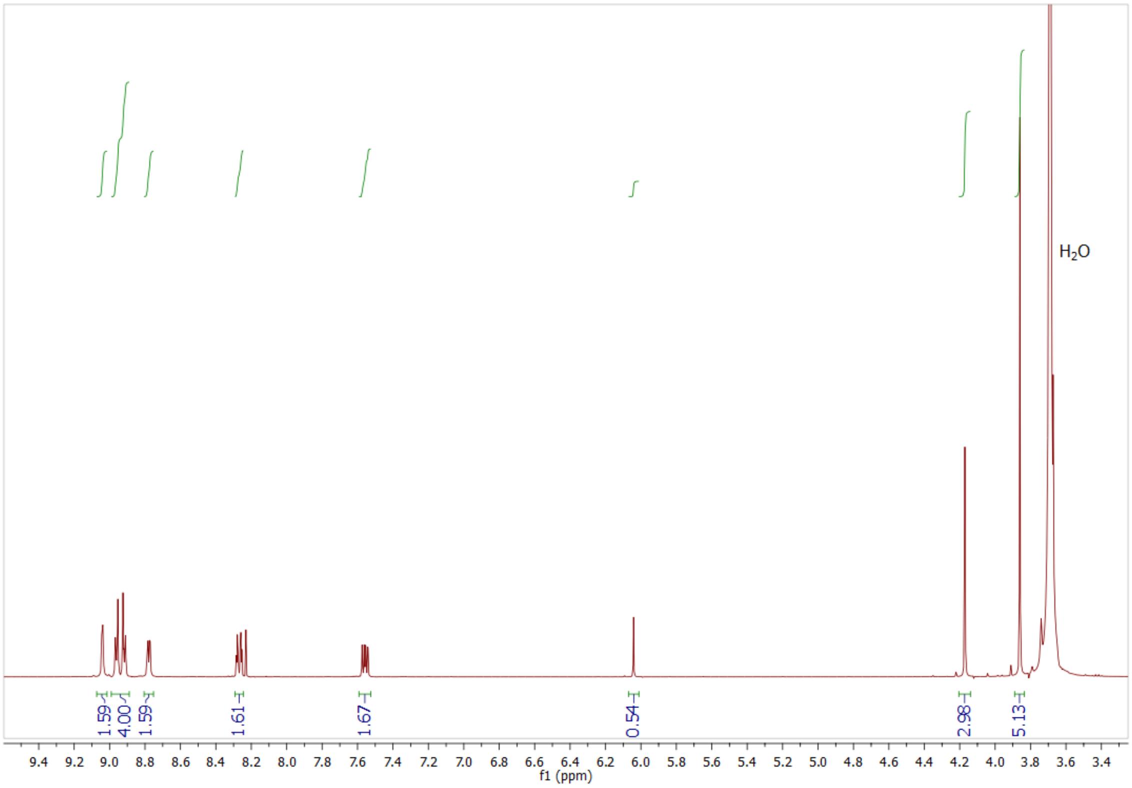

Figure S30: Spectrum B: ^{1}H NMR spectrum in $(CD_3)_2SO$ containing signals assigned to **13a** and **25**. The internal standard 1,3,5-trimethoxybenzene is also present. No signals of crossover product **26** are observed. The full spectrum is shown in Section 7.

As no change in the amount of **13a** present was observed, and no crossover product was formed, we conclude that **13a** is formed irreversibly.

6. Competition experiment: Pyrazine *N*-oxide (1) vs Pyrimidine *N*-oxide (3)

Under an atmosphere of nitrogen in a glove box, bis(methoxy)benzhydryl chloride (0.009 g, 0.03 mmol) was dissolved in dry CD_3CN (0.85 ml). Pyrazine *N*-oxide **1** (6 mg, 0.06 mmol) was then added, followed by pyrimidine *N*-oxide **3** (0.005 g, 0.05 mmol), and then AgOTf (0.013 g, 0.05 mmol), causing the immediate precipitation of AgCl. The reaction vessel was sealed, agitated for 1 – 2 minutes, and filtered (removing AgCl) through a syringe filter into an NMR tube. The NMR tube was then sealed using a rubber septum. The seal was then wrapped with PTFE tape and Parafilm. Finally, the NMR tube was placed in a long Schlenk flask, which was sealed and then removed from the glove box. The sample was brought to the NMR spectrometer inside the long NMR Schlenk flask to protect it from potential ingress of moisture. The sample was removed from this Schlenk flask directly before loading it into the NMR spectrometer.

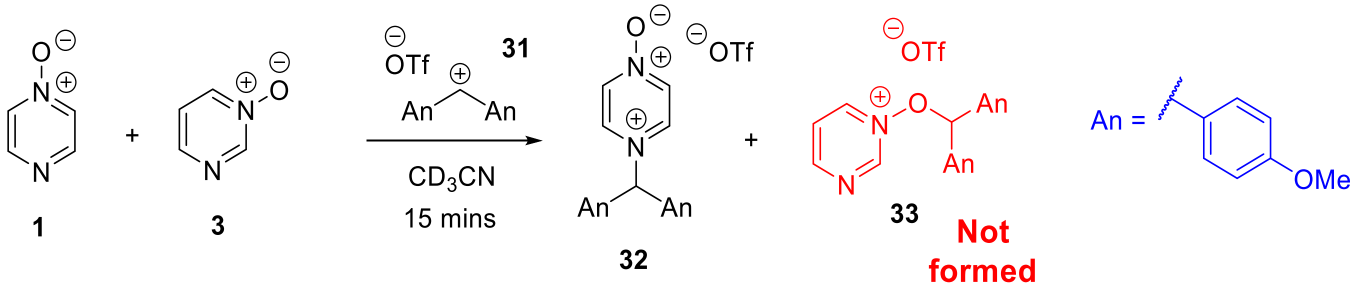

^{1}H NMR (400 MHz, CD_3CN) – Integrations are given relative to 1H of **32**.

Signals assigned to **32**: δ 8.52–8.46 (m, overlaps with signal of **1** at δ 8.46–8.41, contains 4H of **32** (4 × pyrazinium H)), 7.30–7.20 (m, contains 4H of **32** (anisyl protons), overlaps with signal of hydrolysis product), 7.11 – 7.00 (m, 5H, contains Ar_2CH and anisyl protons), 3.84(s, 6H, OCH_3).

Signals assigned to **1**: δ 8.46–8.41 (m, 2H), 8.15–8.08 (m, 2H). 1H of **1** integrates for 0.70 relative to 1H of **32**.

Signals assigned to **3**: δ 8.92 (s, 1H), 8.41–8.32 (app d, app J = 6.3 Hz, 1H), 8.29 – 8.21 (m, 1H), 7.46 – 7.39 (m, 1H). 1H of **3** integrates for 1.58 relative to 1H of **32**.

Small signals arising from the presence of hydrolysis product (bis(4-methoxy)benzhydryl ether) are also present in the ^{1}H NMR spectrum (see Fig. S30 below).

No signals attributable to compound **33** are present, i.e. **32** is the only product formed.

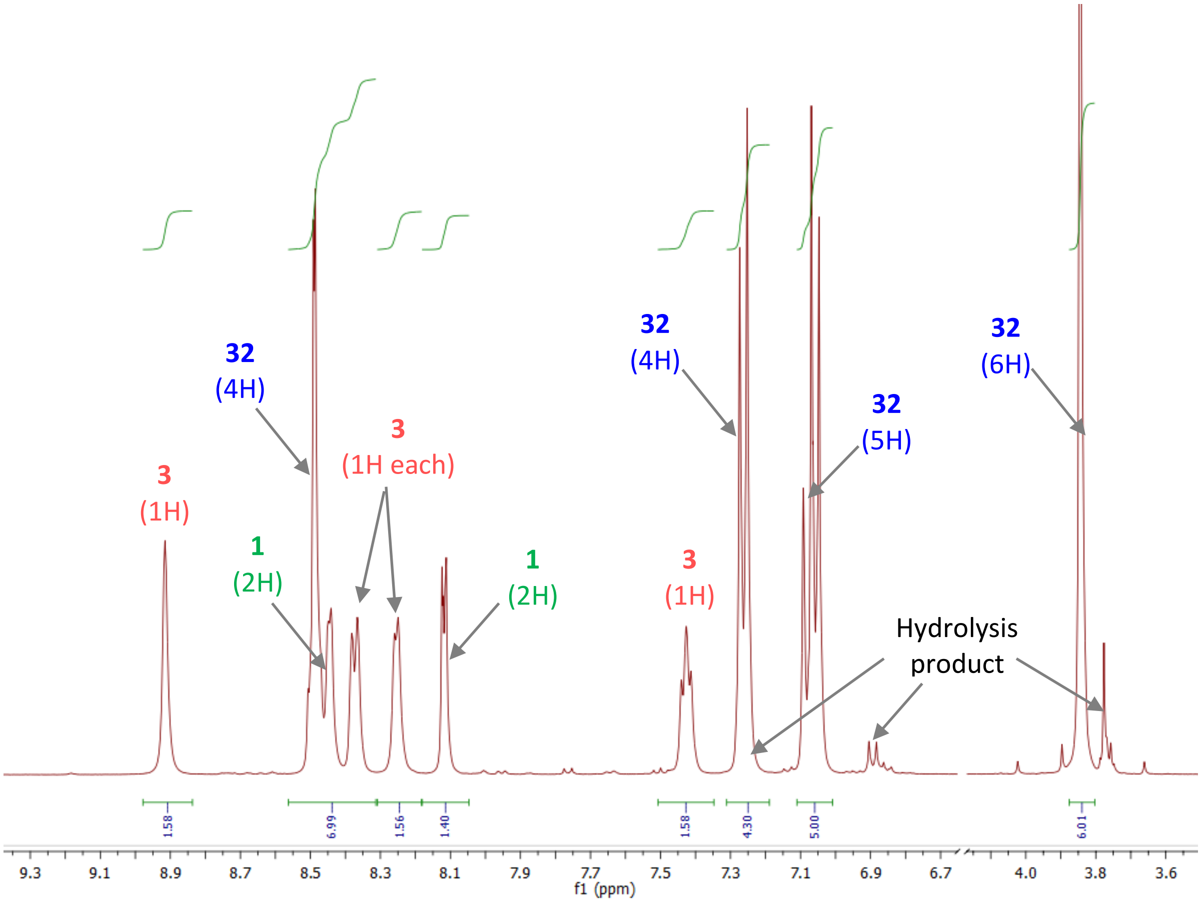

Figure S31: ^{1}H NMR spectrum in CD_3CN containing signals assigned to **1**, **3**, and **32**. No signals assigned to **33** were observed.

7. Full Spectra for compounds produced in Sections 4 – 6

13a in $(CD_3)_2SO$ (From Pyrazine *N*-oxide (1) + MeI)

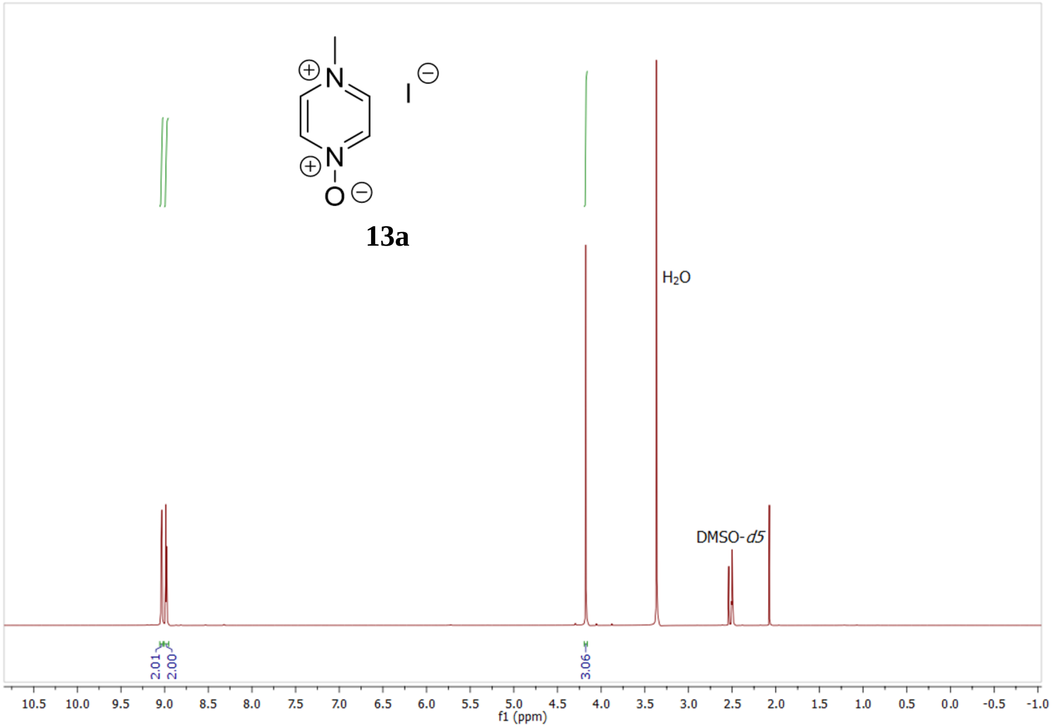

Figure S32 Full ^{1}H NMR spectrum of **13a** in $(CD_3)_2SO$ (600 MHz).

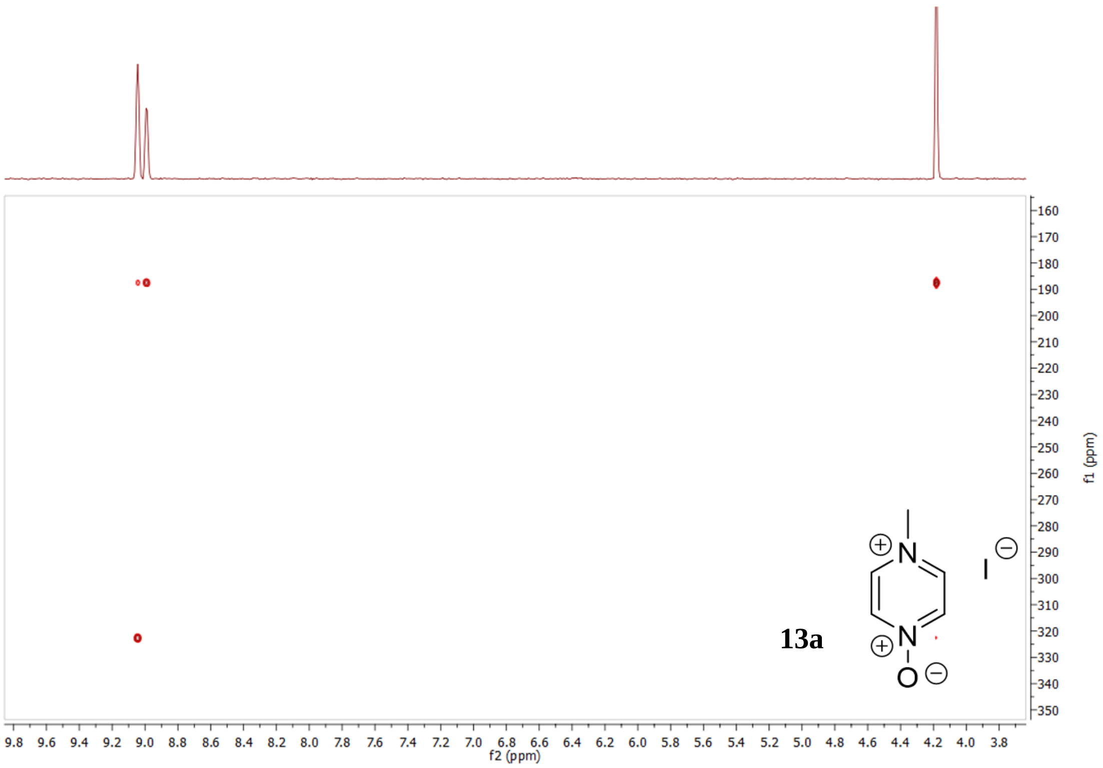

Figure S33: ^{1}H-^{15}N HMBC NMR spectrum of **27a** in $(CD_3)_2SO$.

13a in CD_3CN (From Pyrazine *N*-oxide (1) + MeI)

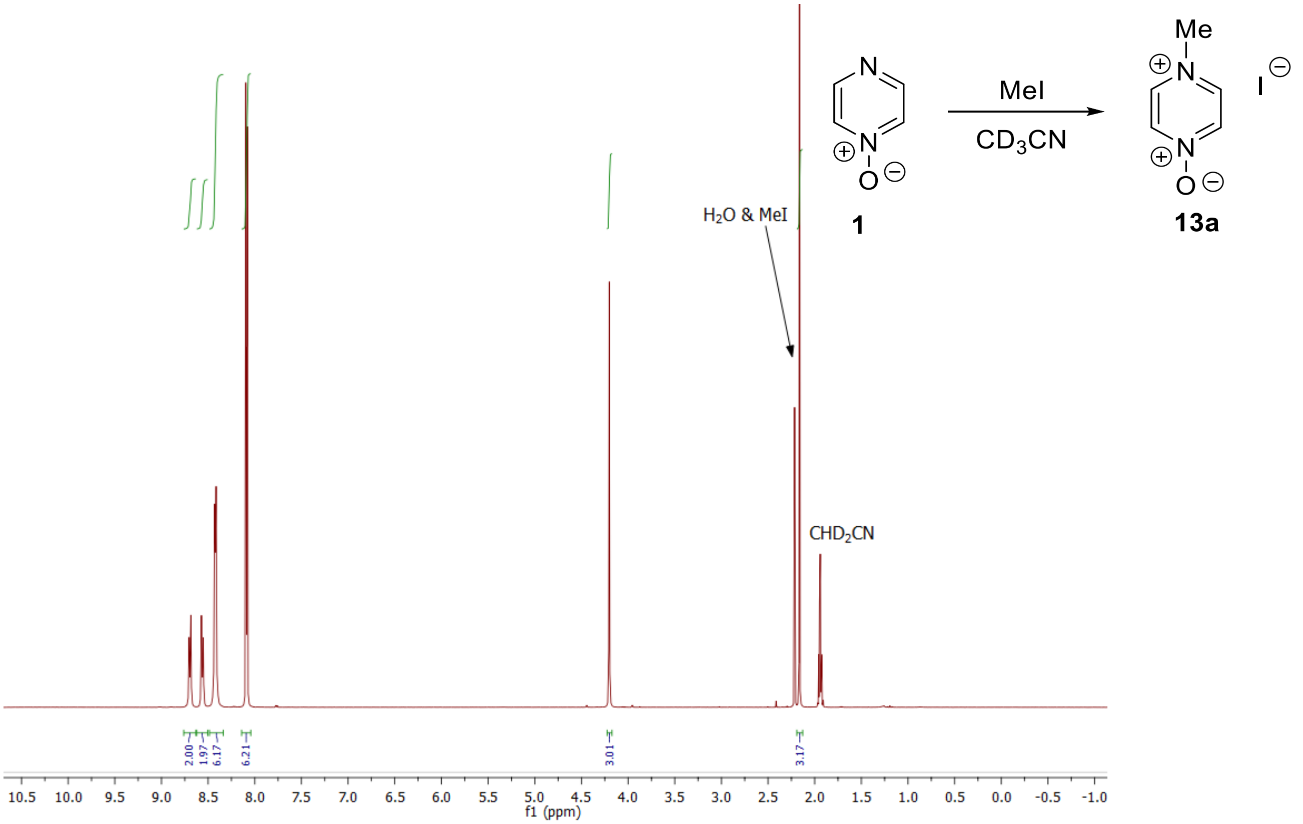

Figure S34 Full ^{1}H NMR spectrum of the reaction of **1** + MeI to give low conversion to **13a** in CD_3CN

13b in $(CD_3)_2SO$ (From Pyrazine *N*-oxide (1) + MeOTf in CH_3CN)

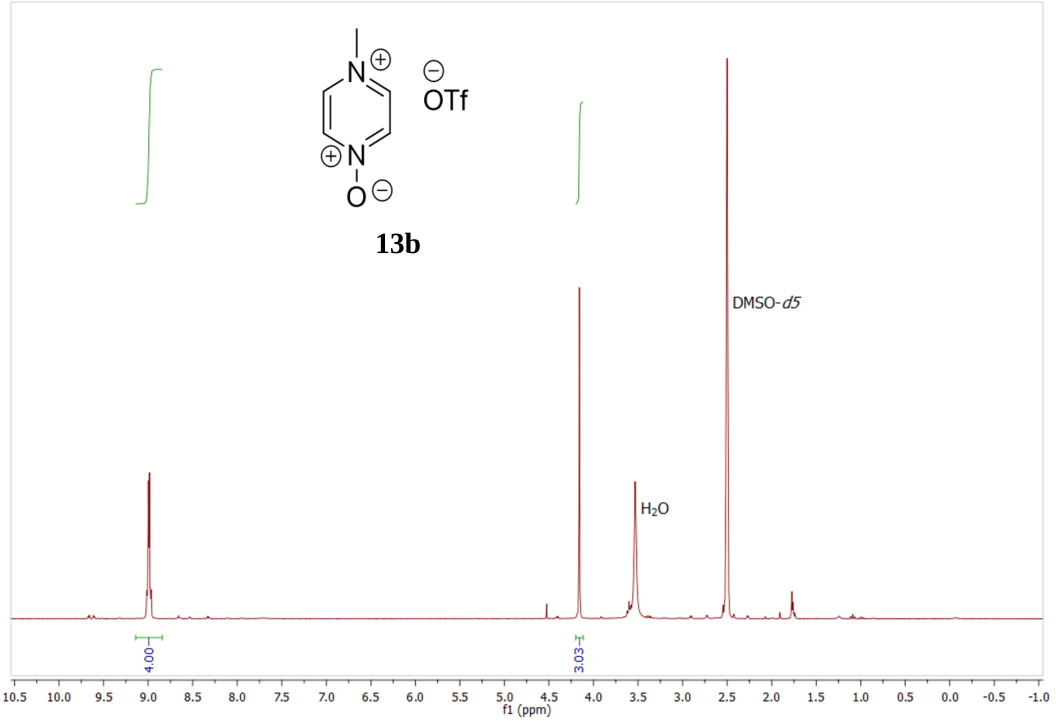

Figure S35: Full ^{1}H spectrum of **13b** in $(CD_3)_2SO$ (300 MHz).

13b and 15b in CD_3CN (From Pyrazine *N*-oxide (1) + MeOTf in CD_3CN)

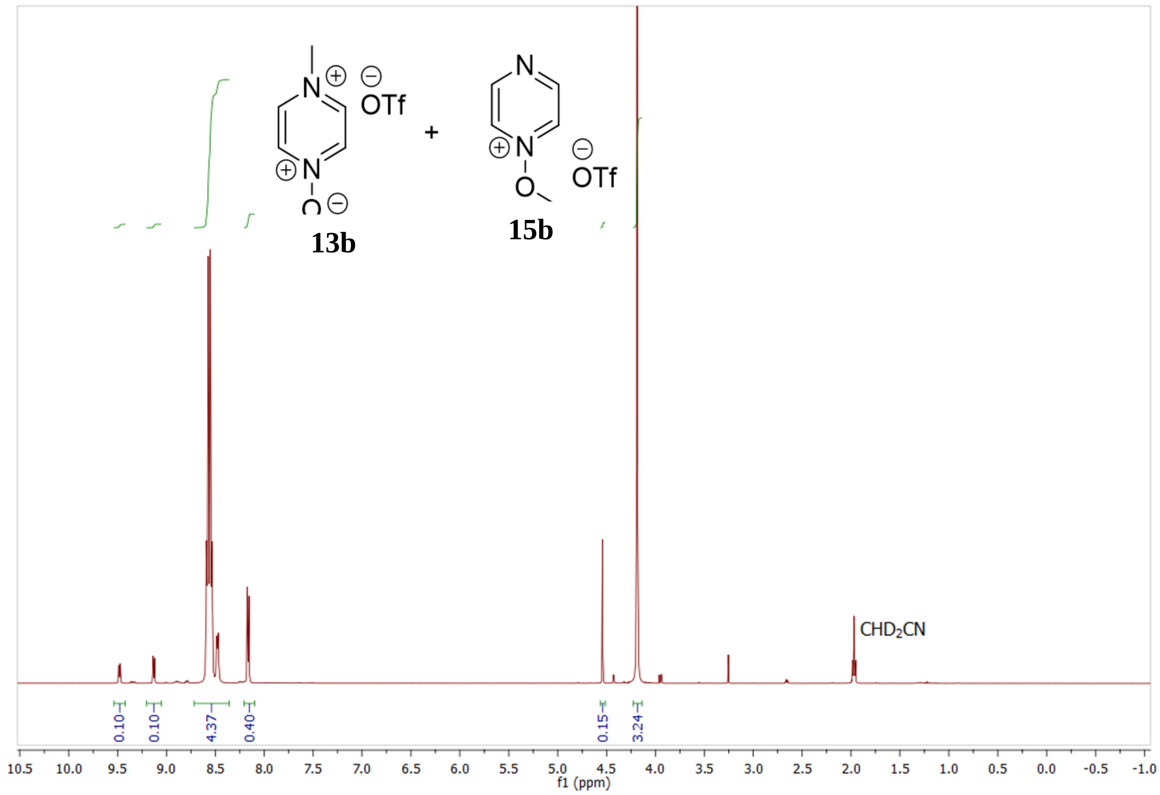

Figure S36: Full ^1H NMR spectrum of **13b**, showing some **15b** and starting material (**1**) in CD_3CN (600 MHz).

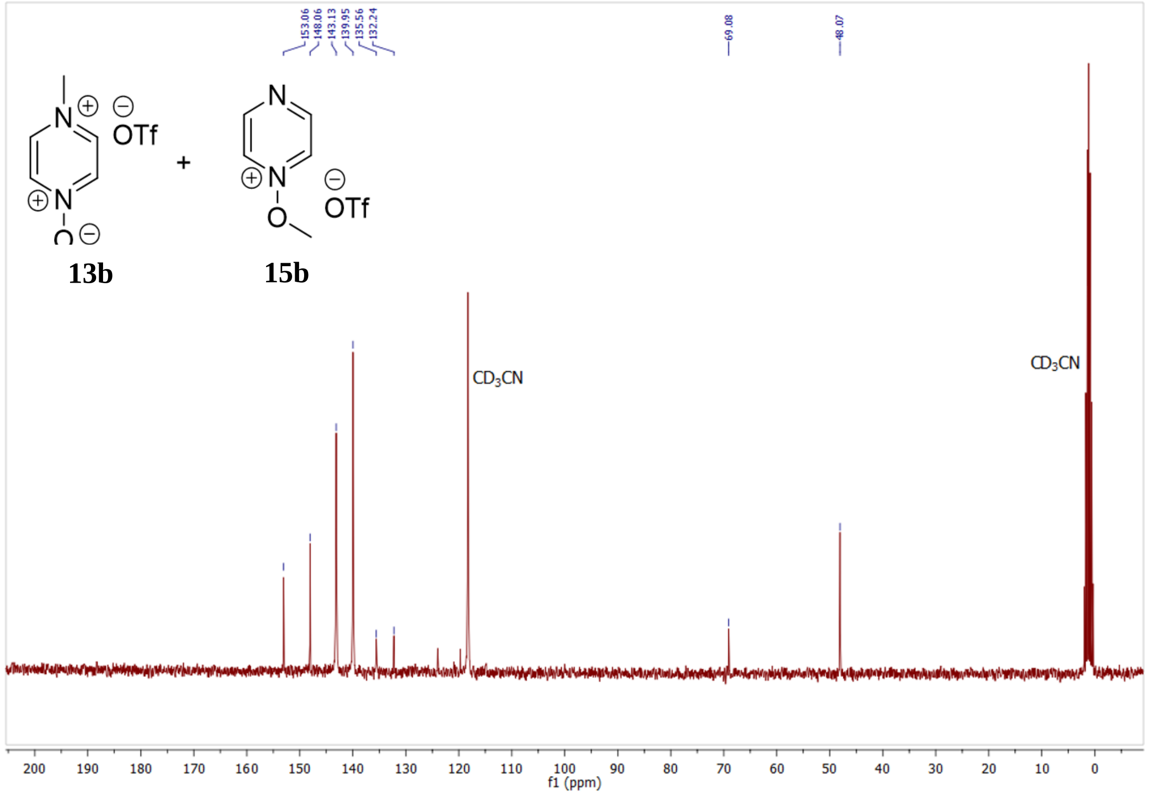

Figure S37: $^{13}C\{^1H\}$ NMR spectrum of **13b**, showing some **15b** and starting material (**1**) in CD_3CN (150 MHz).

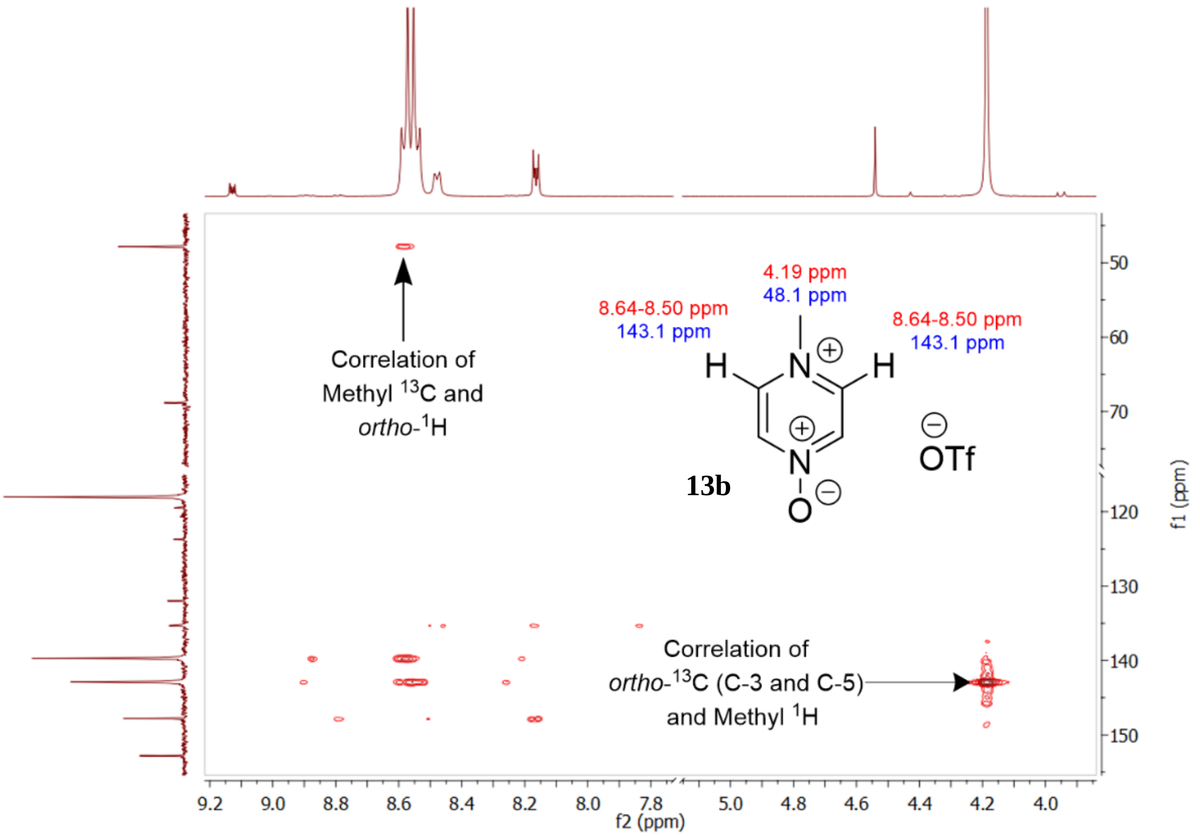

Figure S38: Section of ^{1}H-^{13}C HMBC NMR spectrum of **13b** in CD_3CN.

13b in $(CD_3)_2SO$ (From 1 + MeOTf in CD_3CN, after solvent removal)

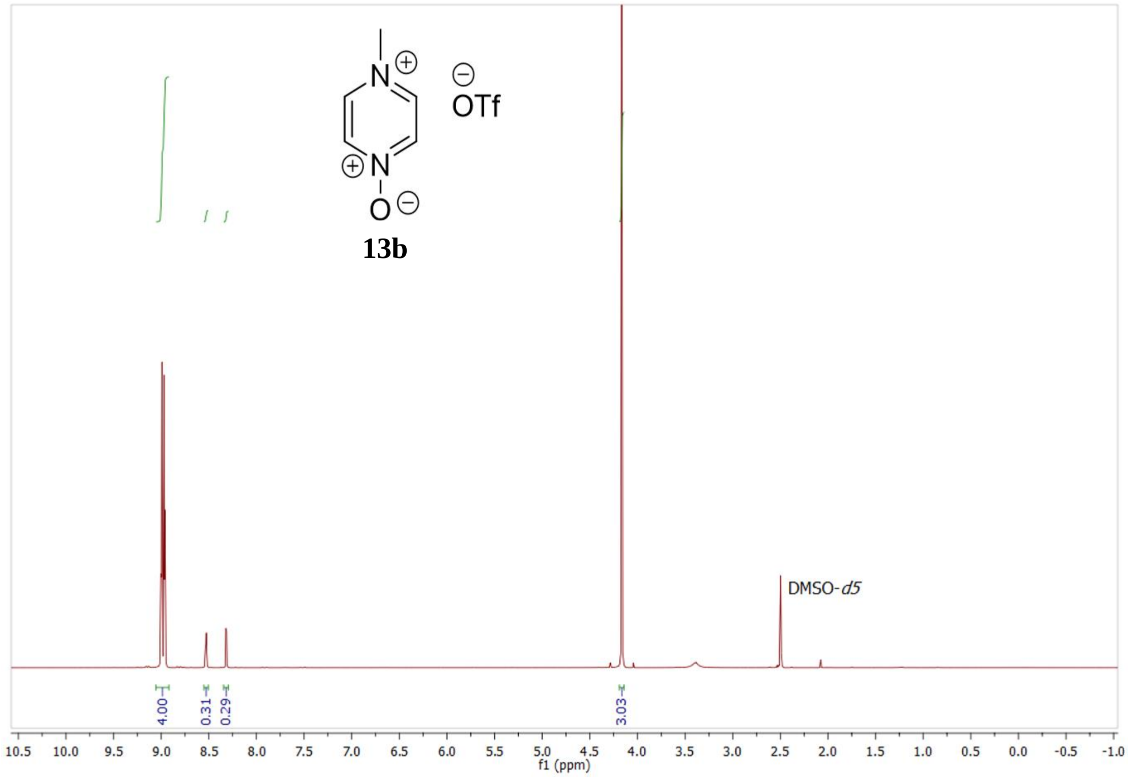

Figure S39: Full ^{1}H NMR spectrum of **13b** and **1** in $(CD_3)_2SO$ after removal of the CD_3CN reaction solvent (600 MHz).

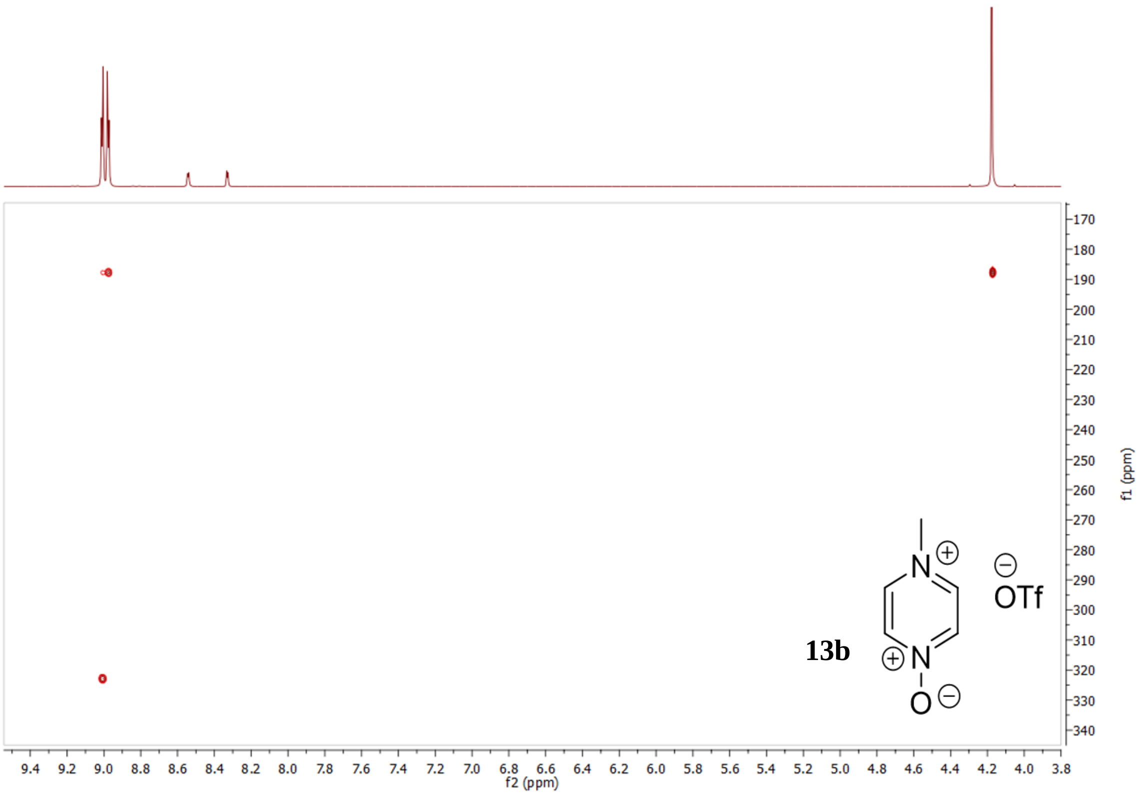

Figure S40: ^{1}H-^{15}N HMBC NMR spectrum of **13b** and **1** in $(CD_3)_2SO$. Removal of the CD_3CN caused the decomposition of **15b**.

13b in $(CD_3)_2SO$ (From Pyrazine *N*-oxide (1) + MeOTf in $(CD_3)_2SO$)

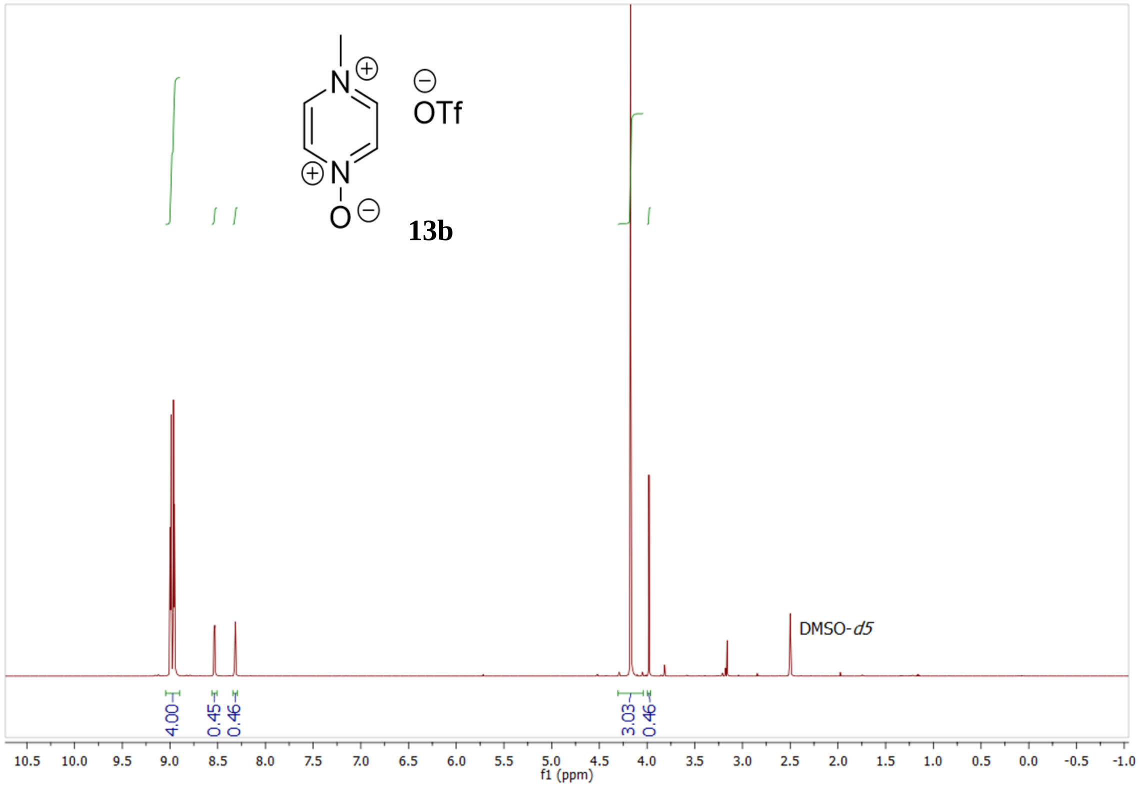

Figure S41: Full ^{1}H NMR spectrum of **13b**, showing some **1** in $(CD_3)_2SO$ (600 MHz). A signal assigned to the methoxydimethylsulfonium salt of $(CD_3)_2SO$ is present at 3.98 ppm.

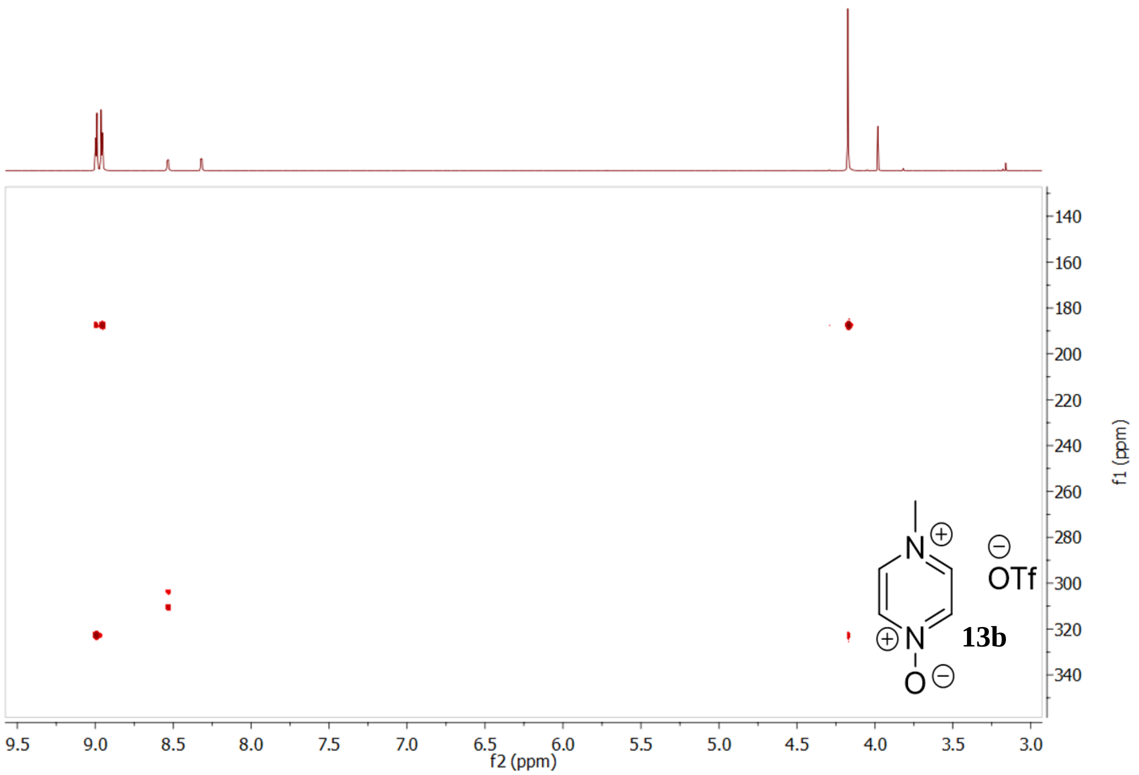

Figure S42: ^{1}H-^{15}N HMBC NMR spectrum of **13b** and **1** in $(CD_3)_2SO$.

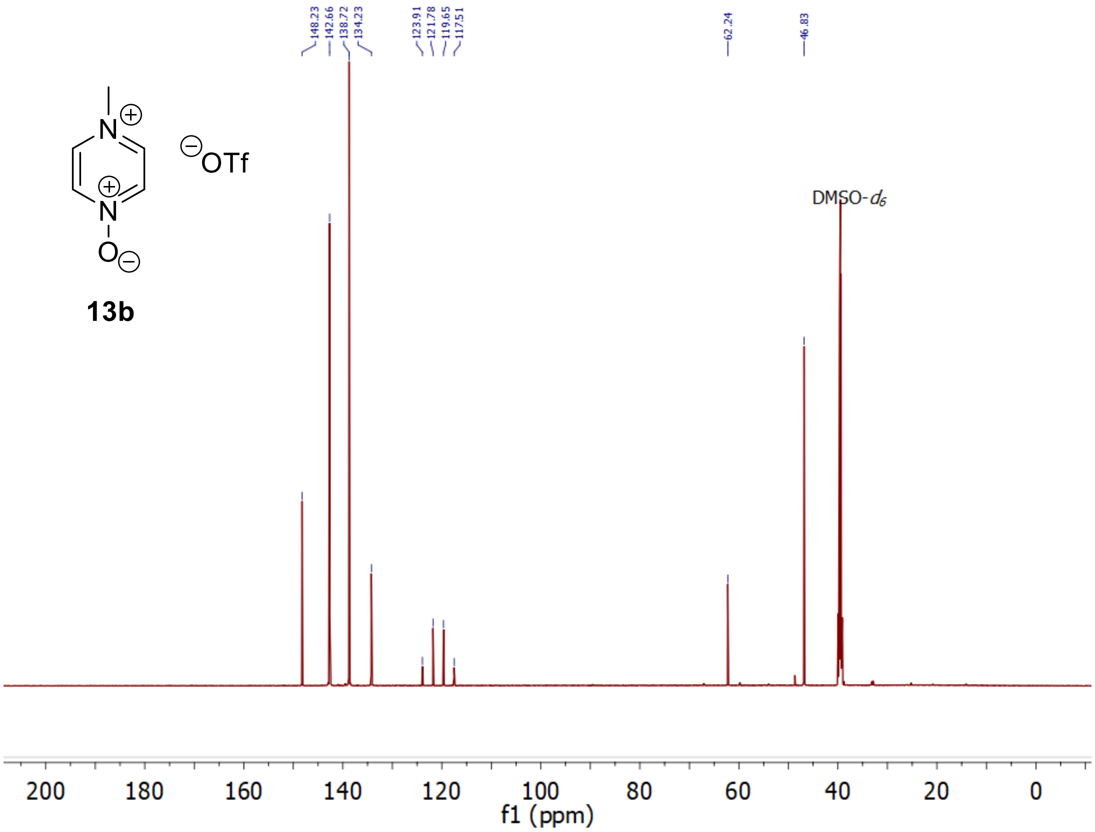

Figure S43: $^{13}C\{^{1}H\}$ NMR spectrum of **13b**, showing some **1** in $(CD_3)_2SO$ (600 MHz). A signal assigned to the methoxydimethylsulfonium salt of $(CD_3)_2SO$ is present at 62.2 ppm.

14 in CD_3CN (From Pyrazine *N*-oxide (1) + benzhydrylium ion 11 in CD_3CN)

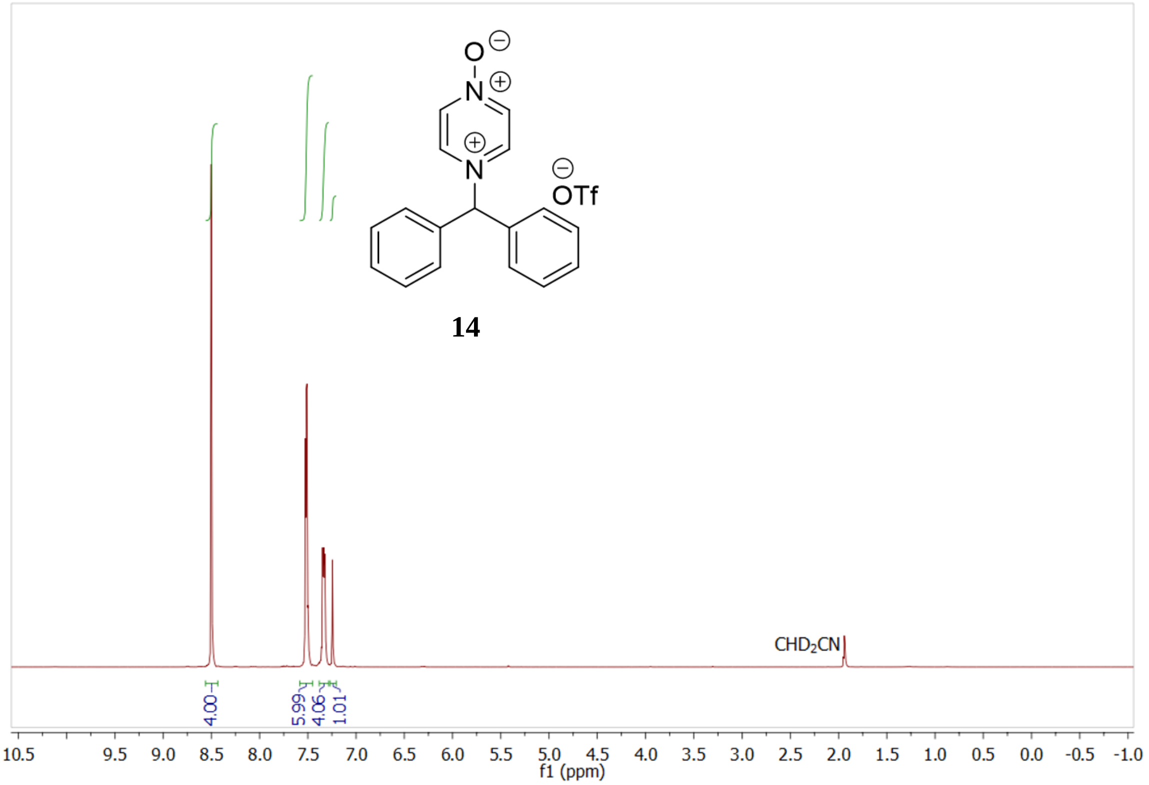

Figure S44: Full ^{1}H NMR spectrum of **14** in CD_3CN (400 MHz)

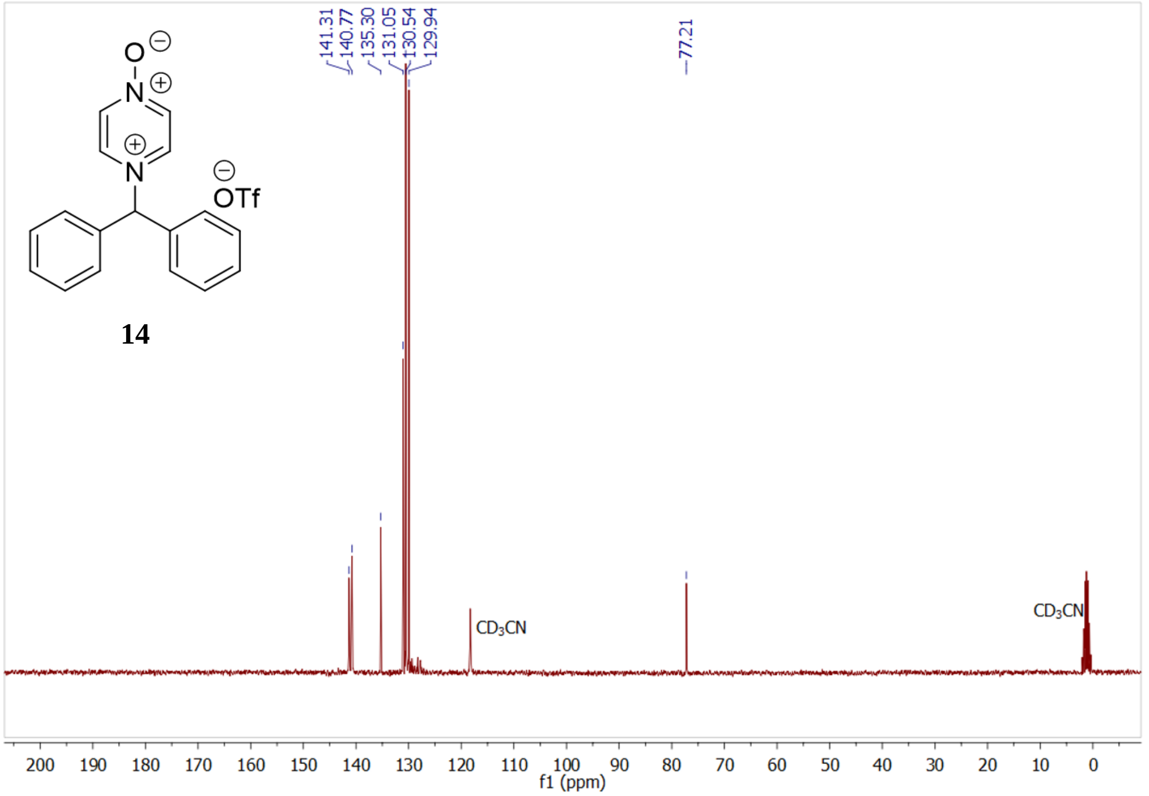

Figure S45: $^{13}C\{^{1}H\}$ NMR spectrum of **14** in CD_3CN (75 MHz)

14 in CH_2Cl_2 (From Pyrazine *N*-oxide (1) + benzhydrylium ion 11 in CH_2Cl_2)

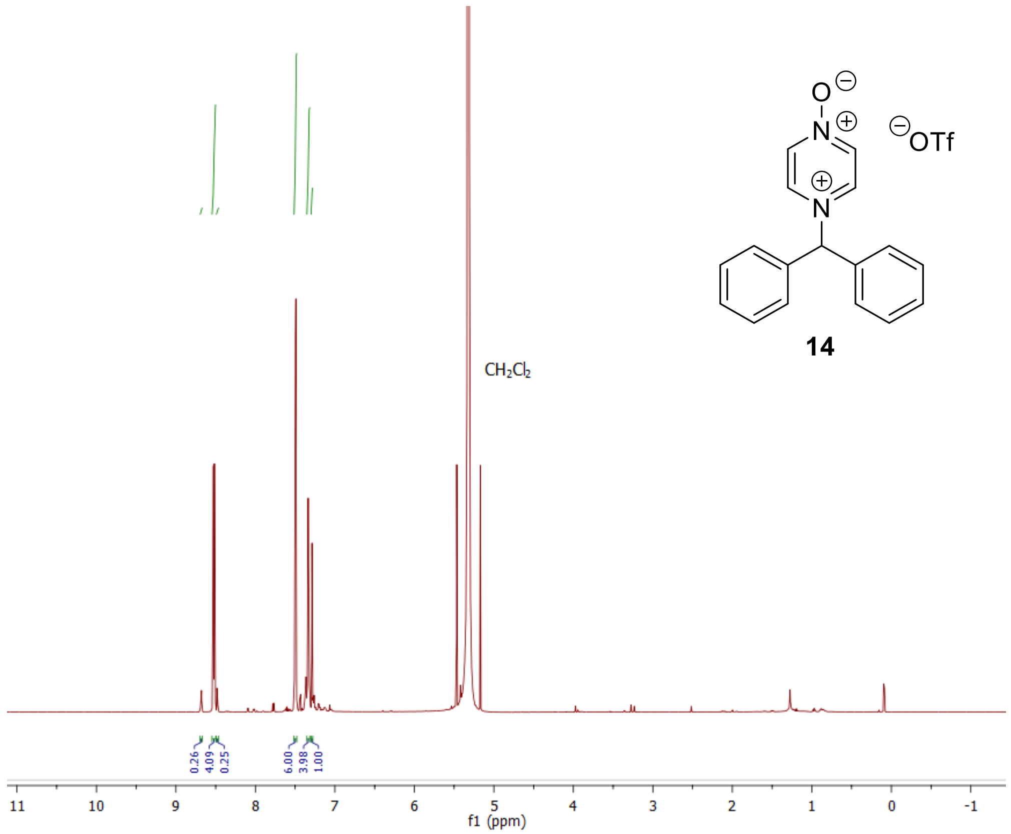

Figure S46: Full ^{1}H NMR spectrum of **14** in CH_2Cl_2 (600 MHz) **acquired with solvent signal suppression**.

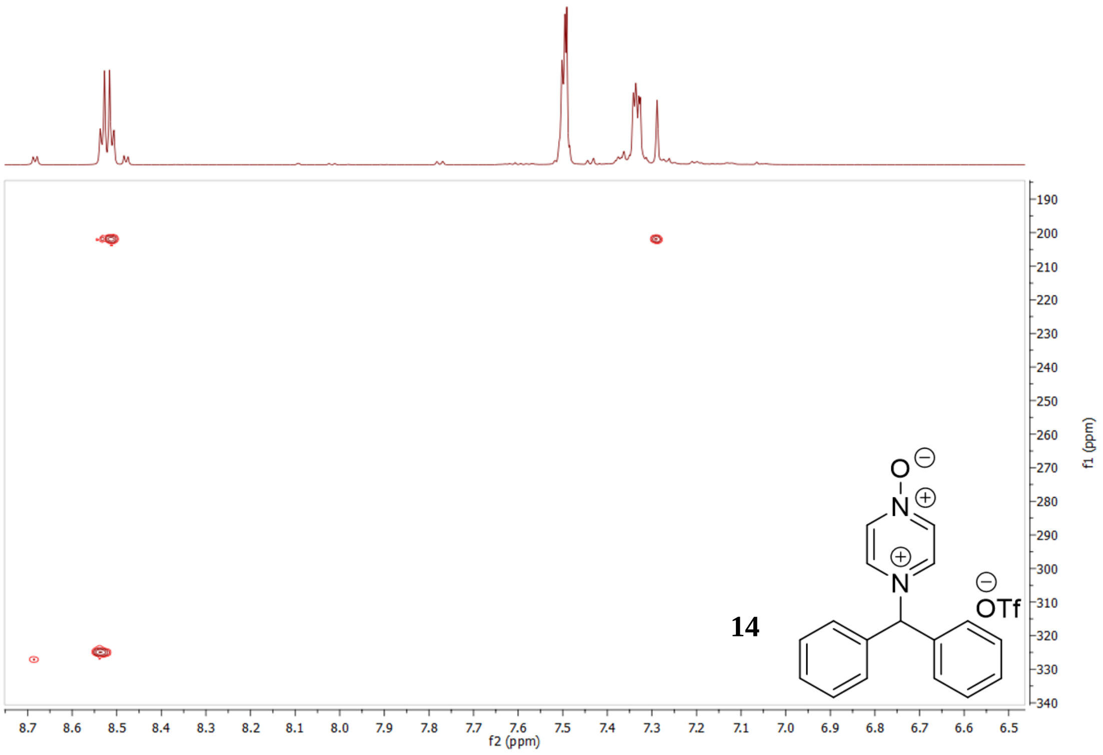

Figure S47: ^{1}H-^{15}N HMBC NMR spectrum of **14** in CH_2Cl_2 acquired with solvent signal suppression.

17a in $(CD_3)_2SO$ (From Quinoxaline *N*-oxide (2) + MeI)

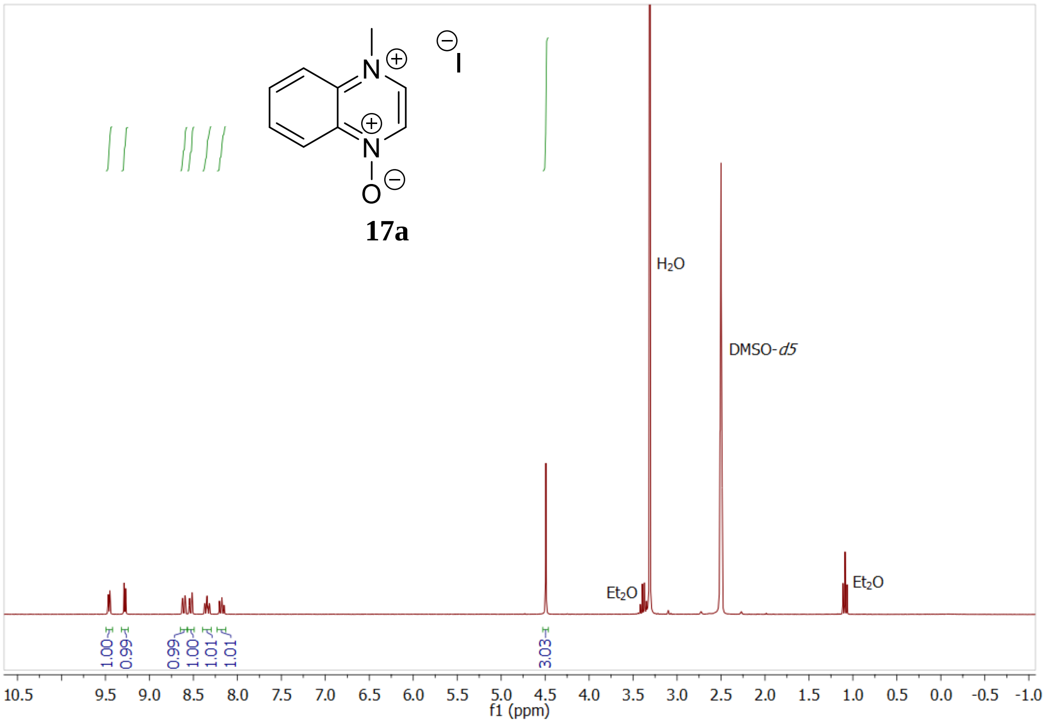

Figure S48: Full ^1H NMR spectrum of **17a** in $(CD_3)_2SO$ (600 MHz).

17a in $(CH_3)_2SO$ (From Quinoxaline *N*-oxide (2) + MeI)

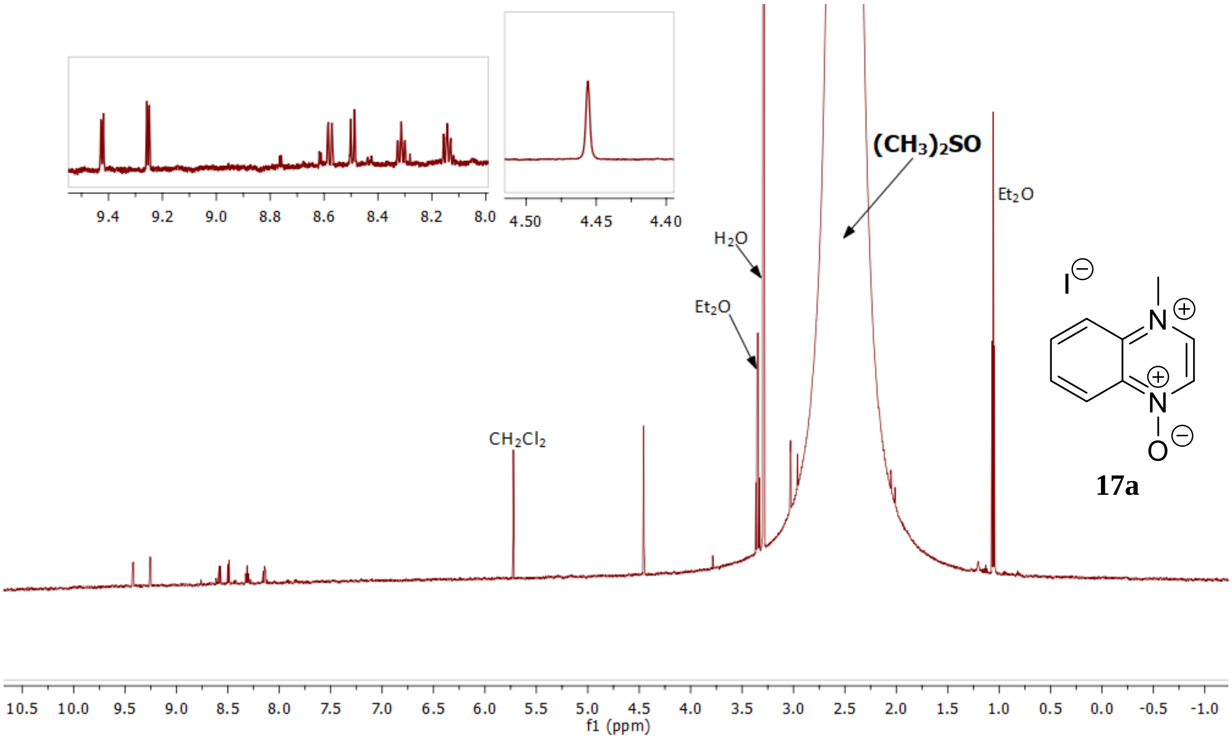

Figure S49: Full ^1H NMR spectrum of **17a** in $(CH_3)_2SO$ (600 MHz) **acquired with solvent signal suppression**.

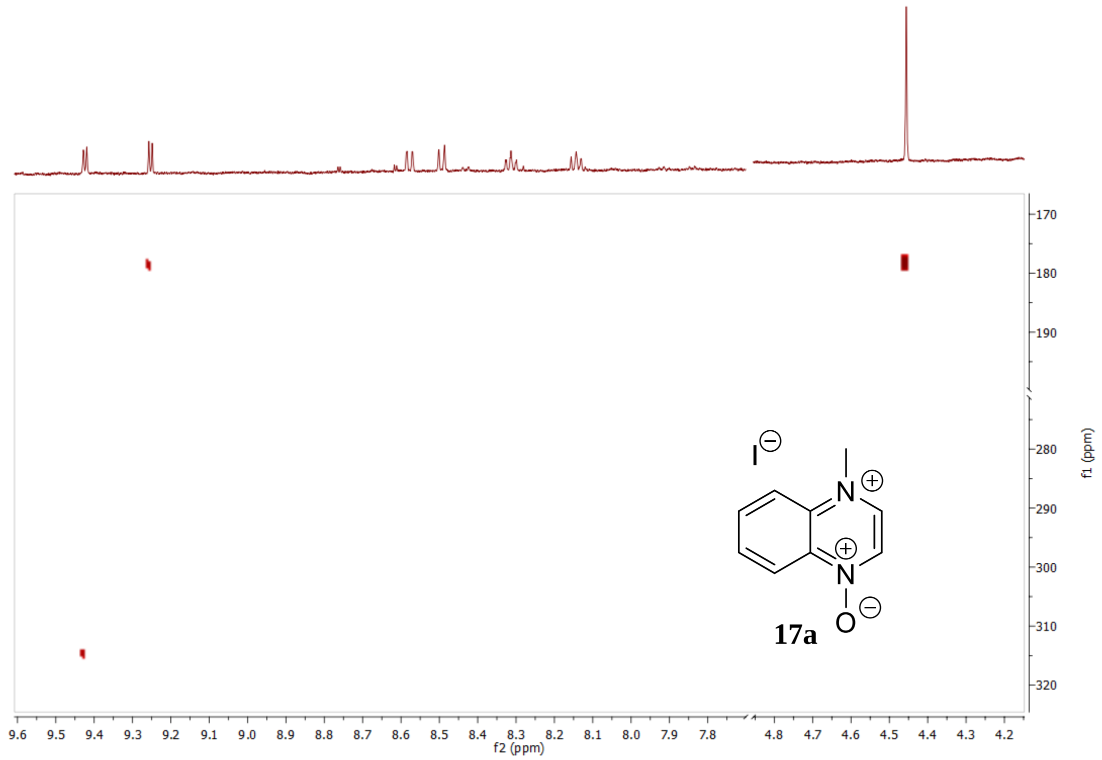

Figure S50: ^{1}H-^{15}N HMBC NMR spectrum of **17a** in $(CH_3)_2SO$ (150 MHz) acquired with solvent signal suppression.

17b in $(CD_3)_2SO$ (From Quinoxaline *N*-oxide (2) + MeOTf in CH_3CN)

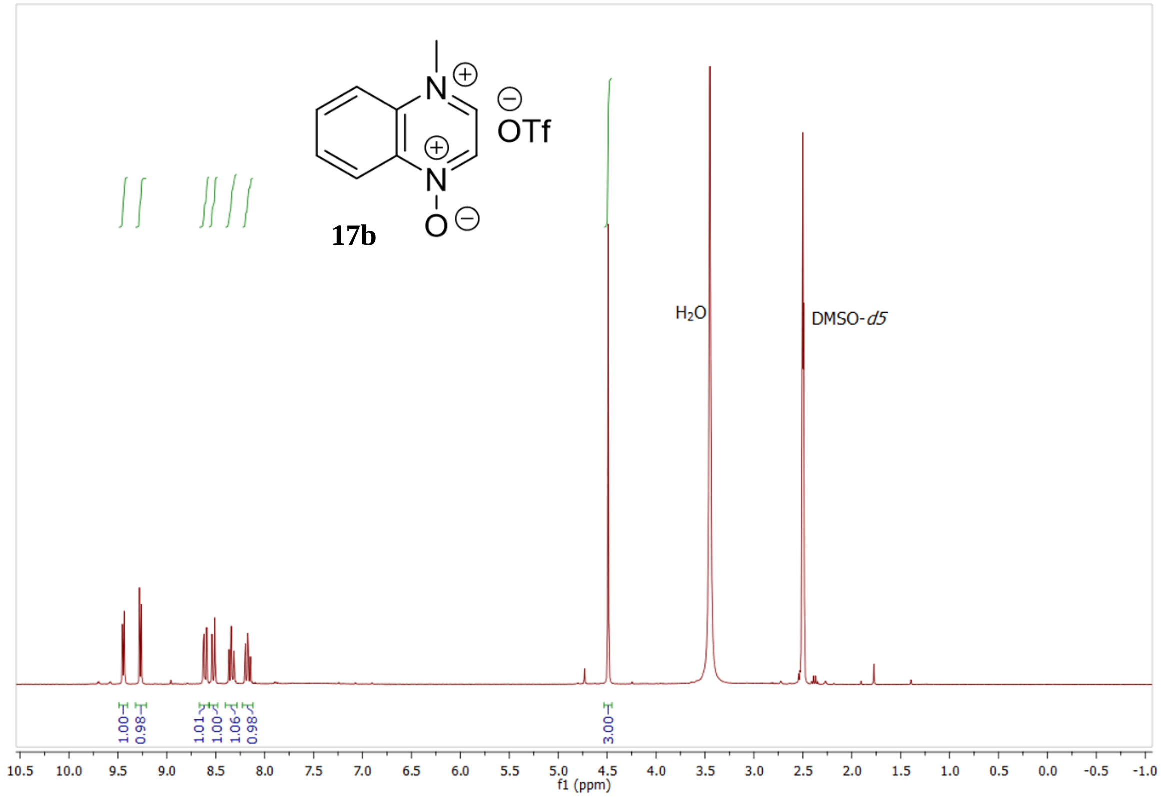

Figure S51 Full ^{1}H NMR spectrum of **17b** in $(CD_3)_2SO$ (300 MHz).

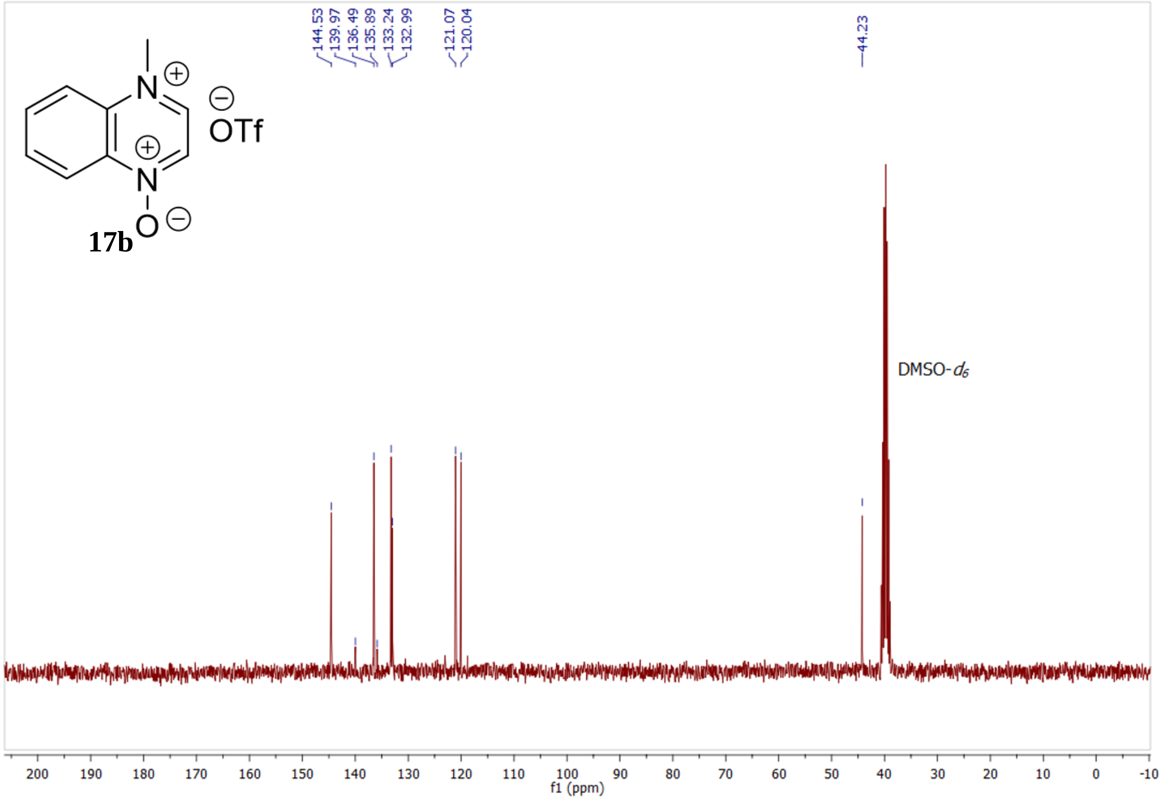

Figure S52: $^{13}C\{^1H\}$ NMR spectrum of **17b** in $(CD_3)_2SO$ (75 MHz).

17b and 19b in CD_3CN (From Quinoxaline *N*-oxide (2) + MeOTf in CD_3CN)

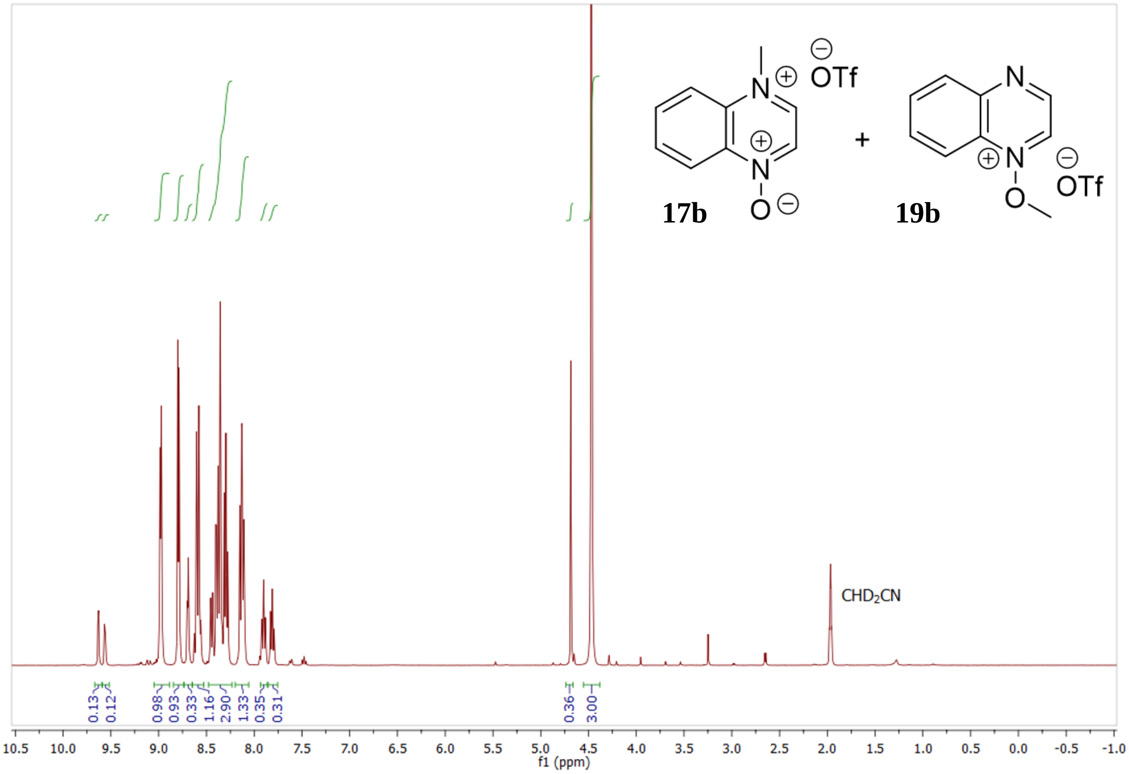

Figure S53: Full ^1H NMR Spectrum of **17b**, **19b** and **2** in CD_3CN (400MHz).

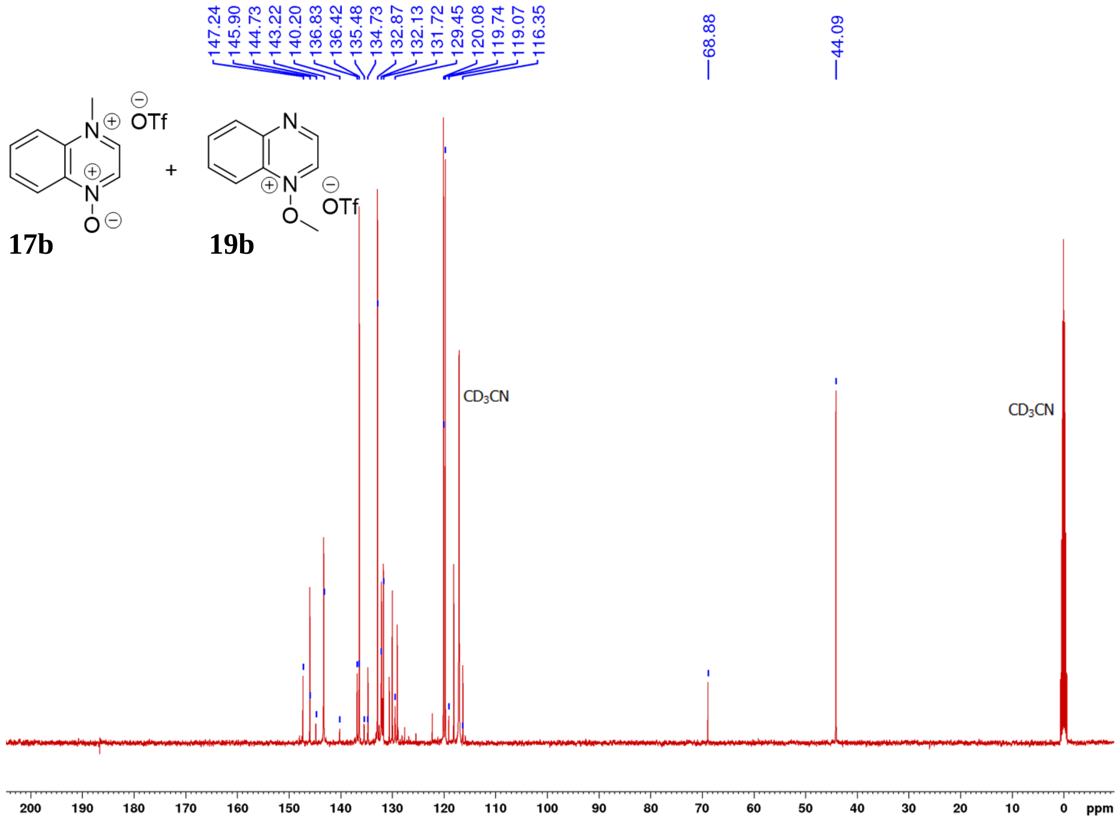

Figure S54: $^{13}C\{^{1}H\}$ NMR Spectrum of **17b**, **19b** and **2** in CD_3CN (100 MHz)

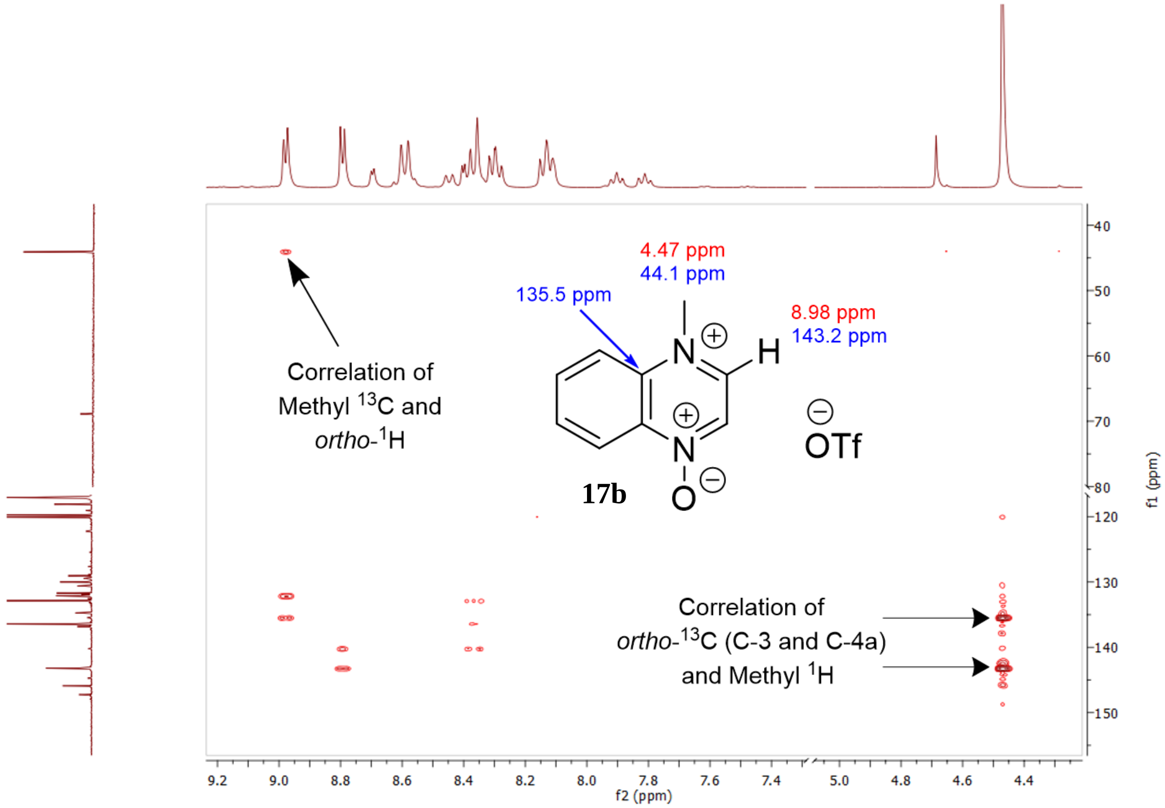

Figure S55: Section of ^{1}H-^{13}C HMBC NMR spectrum of **17b** in CD_3CN.

17b in $(CD_3)_2SO$ (From reaction of 2 + MeOTf in CD_3CN after solvent removal)

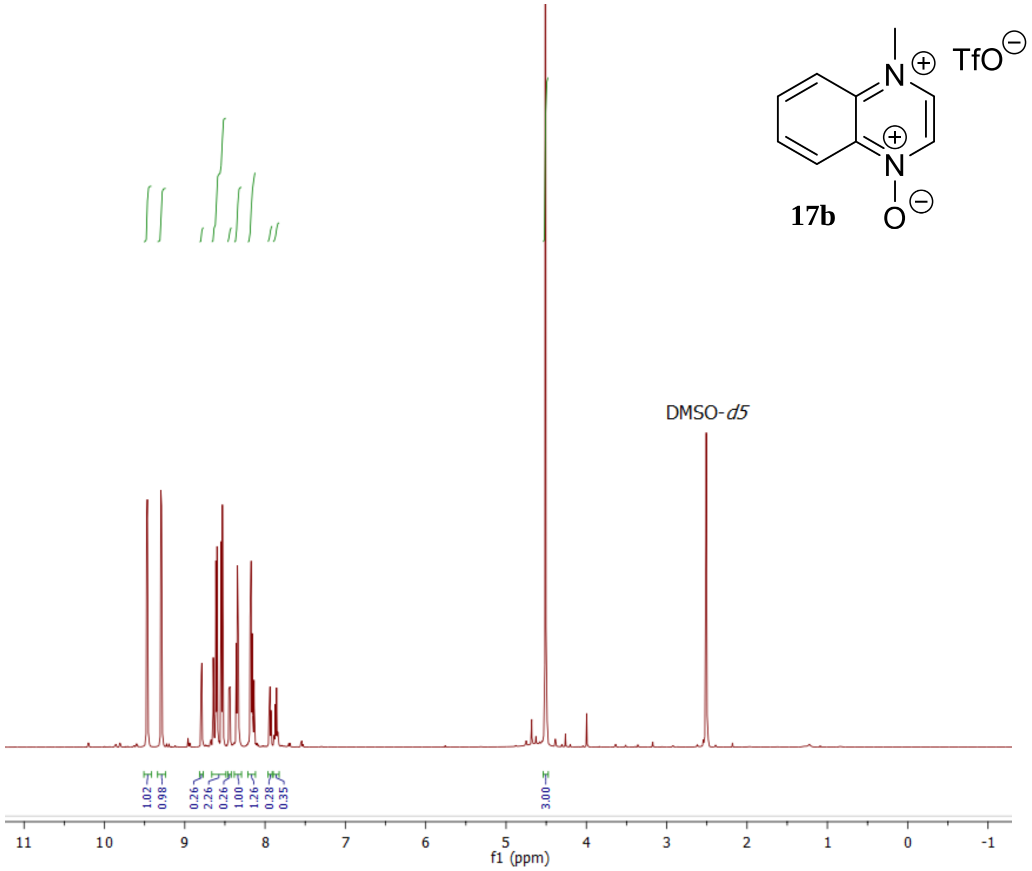

Figure S56: Full ^{1}H spectrum of **17b** and **2** in $(CD_3)_2SO$ (600 MHz). Removal of the CD_3CN caused the decomposition of **19b**.

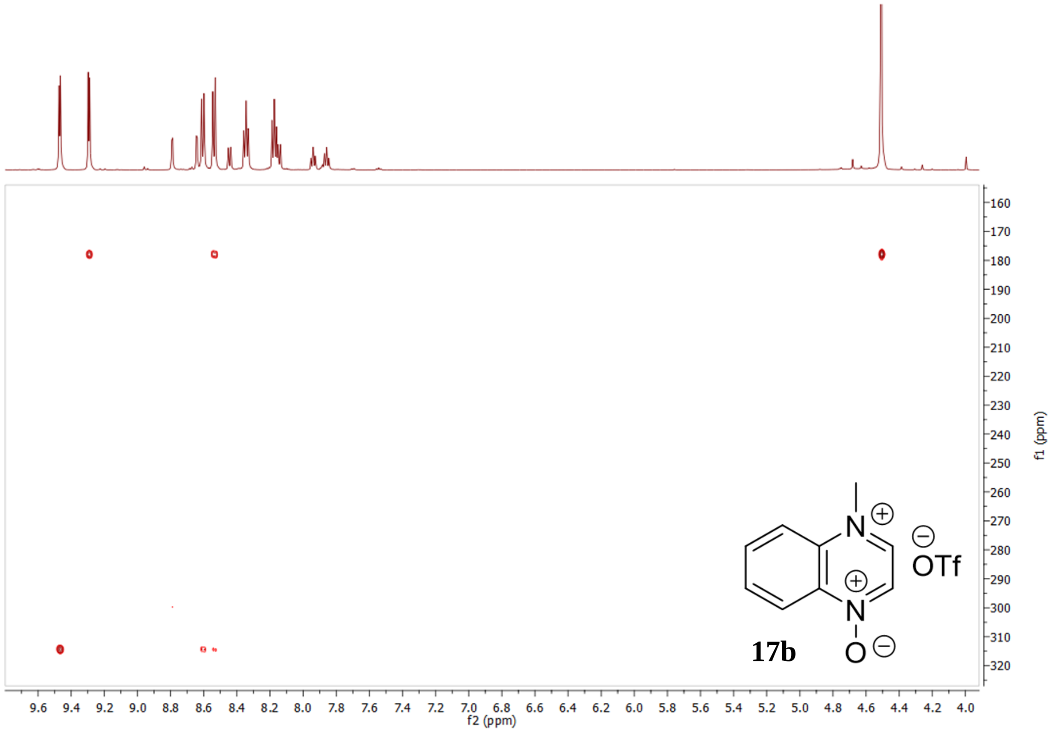

Figure S57: ^{1}H-^{15}N HMBC NMR spectrum of **17b** and **2** in $(CD_3)_2SO$. Removal of the CD_3CN caused the decomposition of **19b**.

17b in $(CD_3)_2SO$ (From Quinoxaline *N*-oxide (2) + MeOTf in $(CD_3)_2SO$)

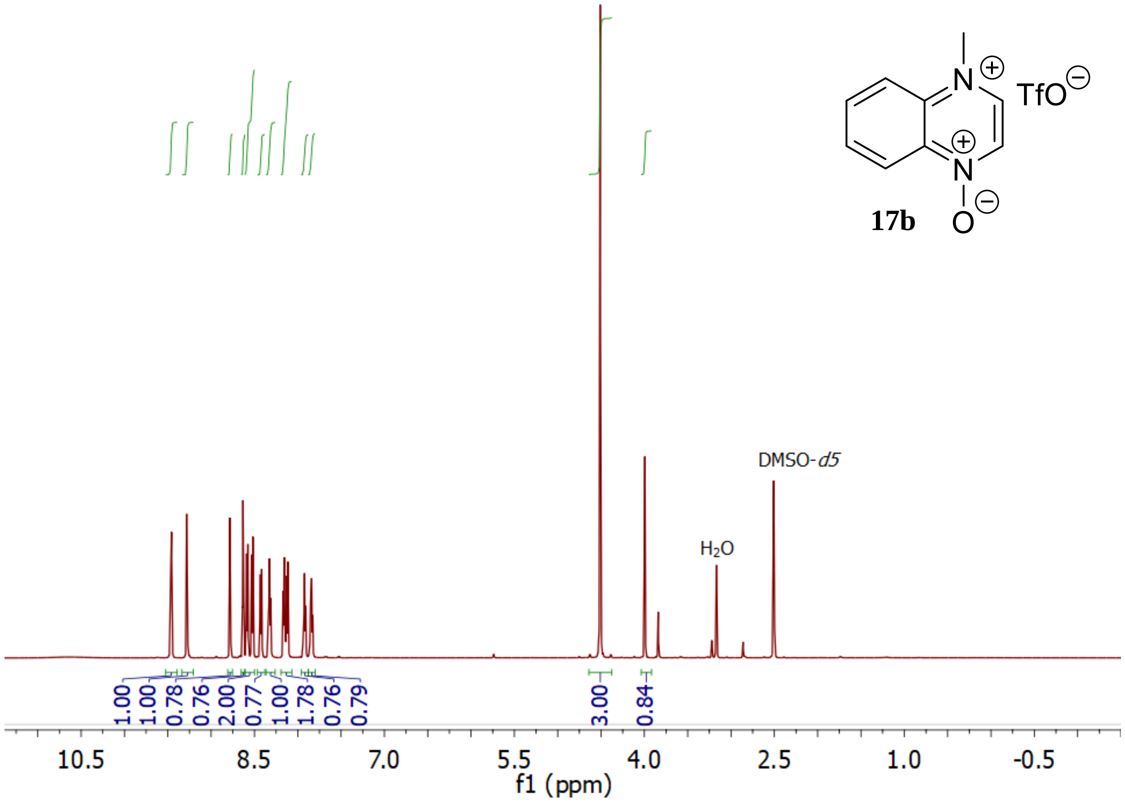

Figure S58: Full ^{1}H NMR spectrum of **17b** + starting material **2** in $(CD_3)_2SO$ (600 MHz). A signal assigned to the methoxydimethylsulfonium salt of $(CD_3)_2SO$ is present at 3.99 ppm.

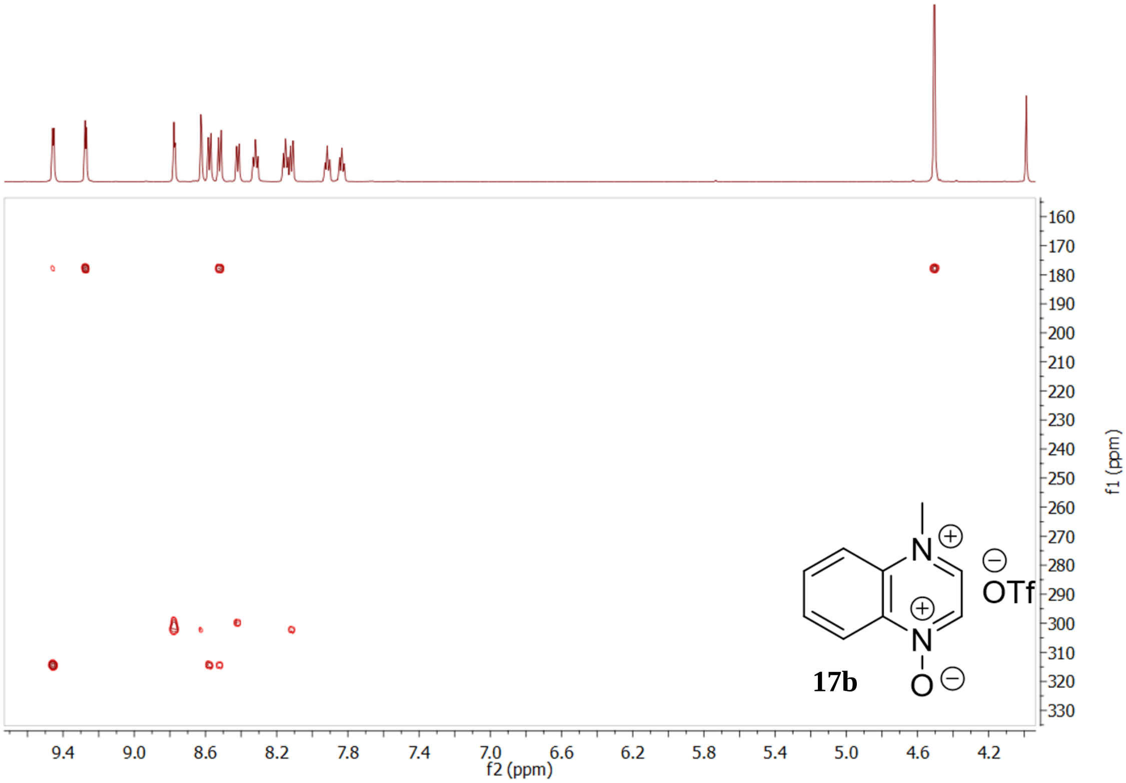

Figure S59: ^{1}H-^{15}N HMBC NMR spectrum of **17b** in $(CD_3)_2SO$, showing some **2**.

18 and 20 in CD_2Cl_2 (From reaction of 2 + 12 in CD_2Cl_2)

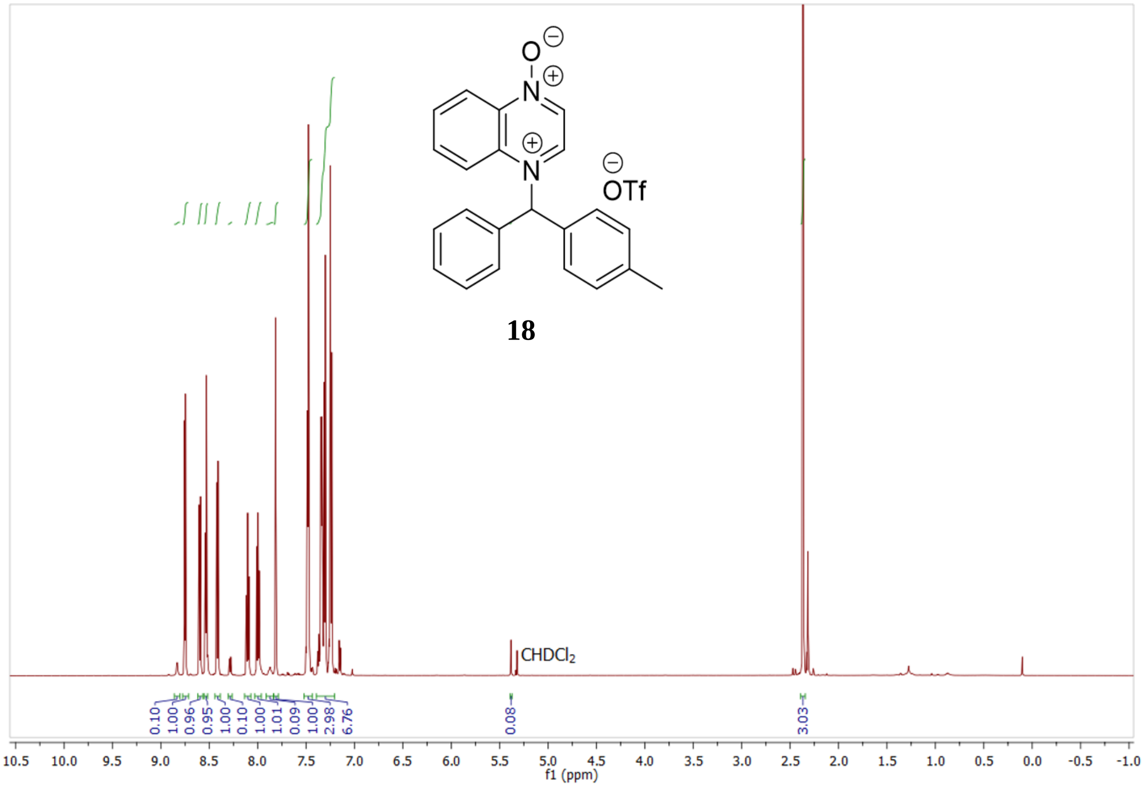

Figure S60: Full ^{1}H NMR spectrum of **18** in CD_2Cl_2 (600 MHz). Small signals assigned to **20** are also present.

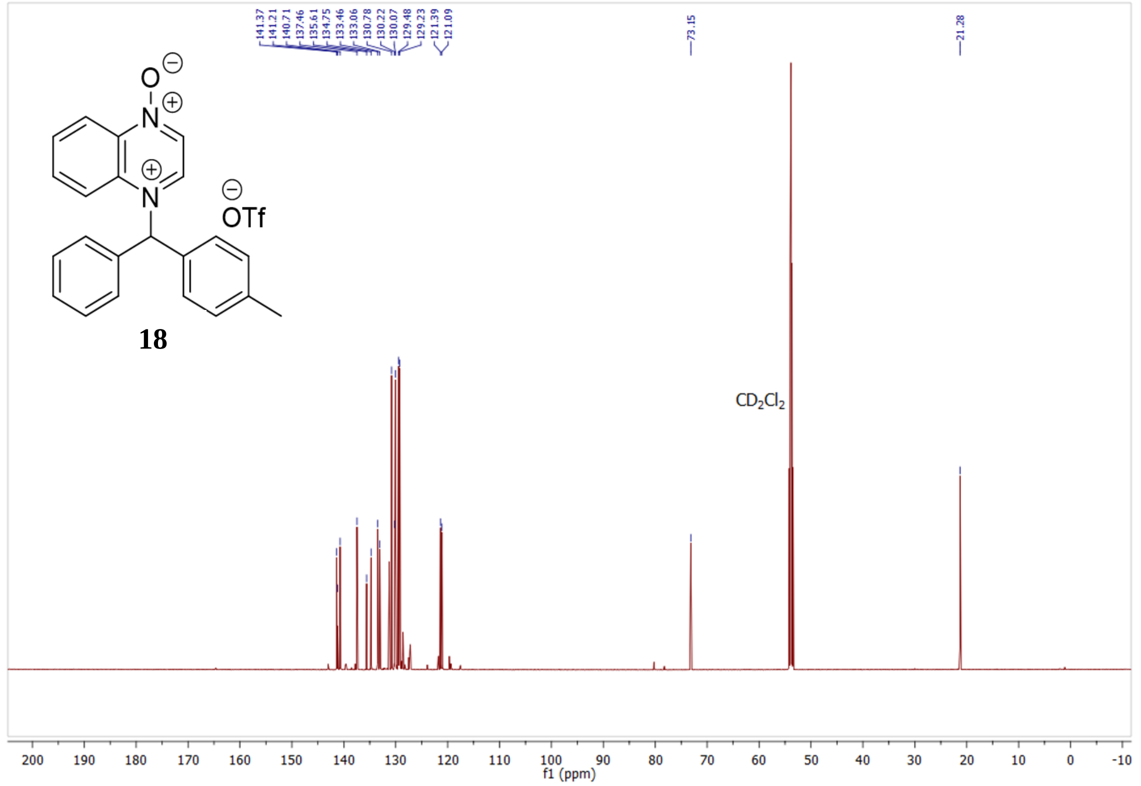

Figure S61: ^{13}C{^{1}H} NMR spectrum of **18** in CD_2Cl_2 (150 MHz). Small signals assigned to **20** are also present. A ^{13}C NMR signal assigned to the $CF_3SO_3^{-}$ ion is present at δ 120.7 ppm.

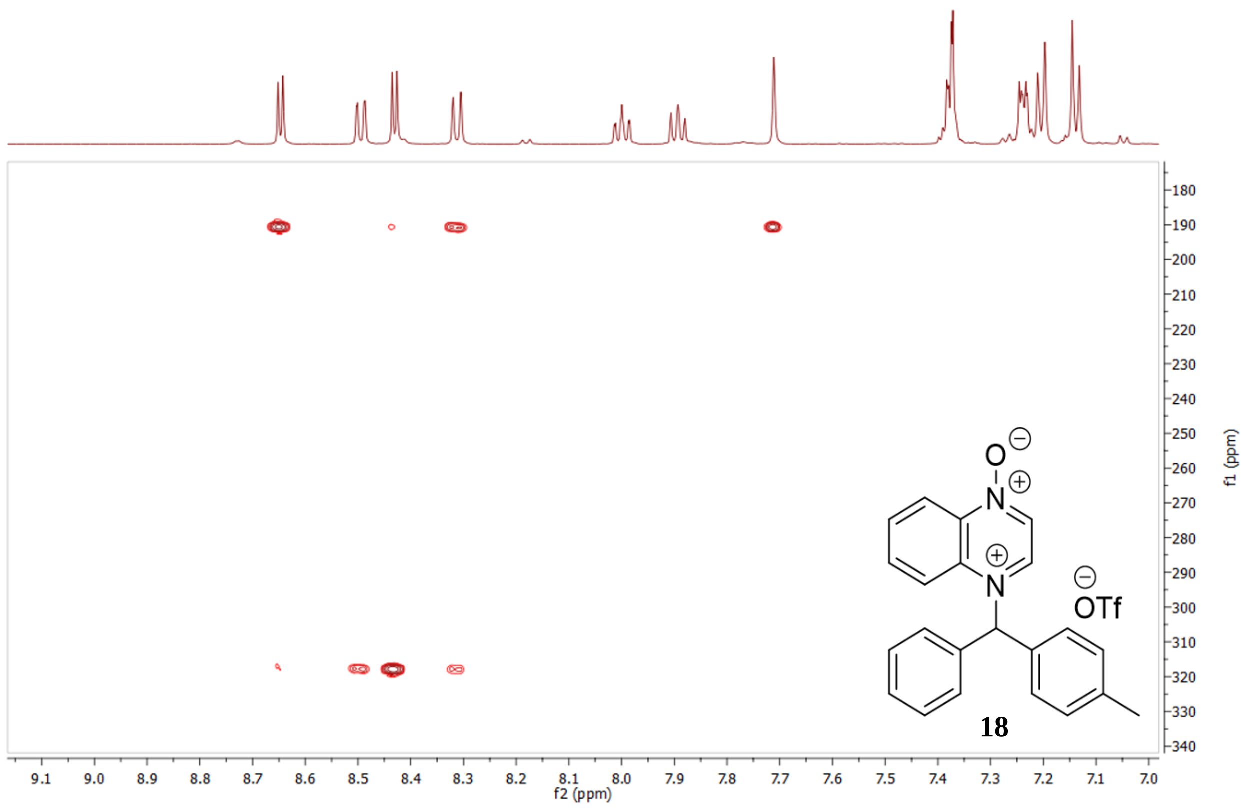

Figure S62: ^{1}H-^{15}N HMBC NMR spectrum of **18** in CD_2Cl_2.

23b in CD_3CN (From Pyrimidine *N*-oxide (3) + MeOTf in CD_3CN)

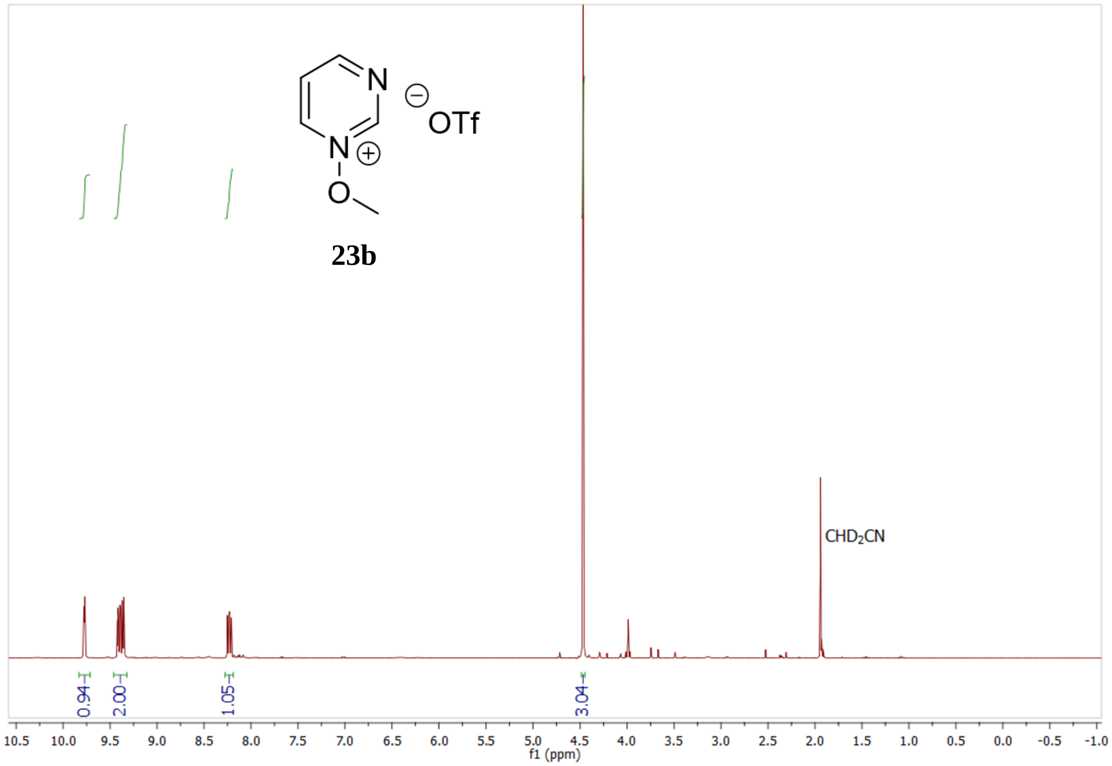

Figure S63: Full ^{1}H spectrum of **23b** in CD_3CN (300 MHz).

23b in $(CH_3)_2SO$ (From Pyrimidine *N*-oxide (3) + MeOTf in CH_3CN)

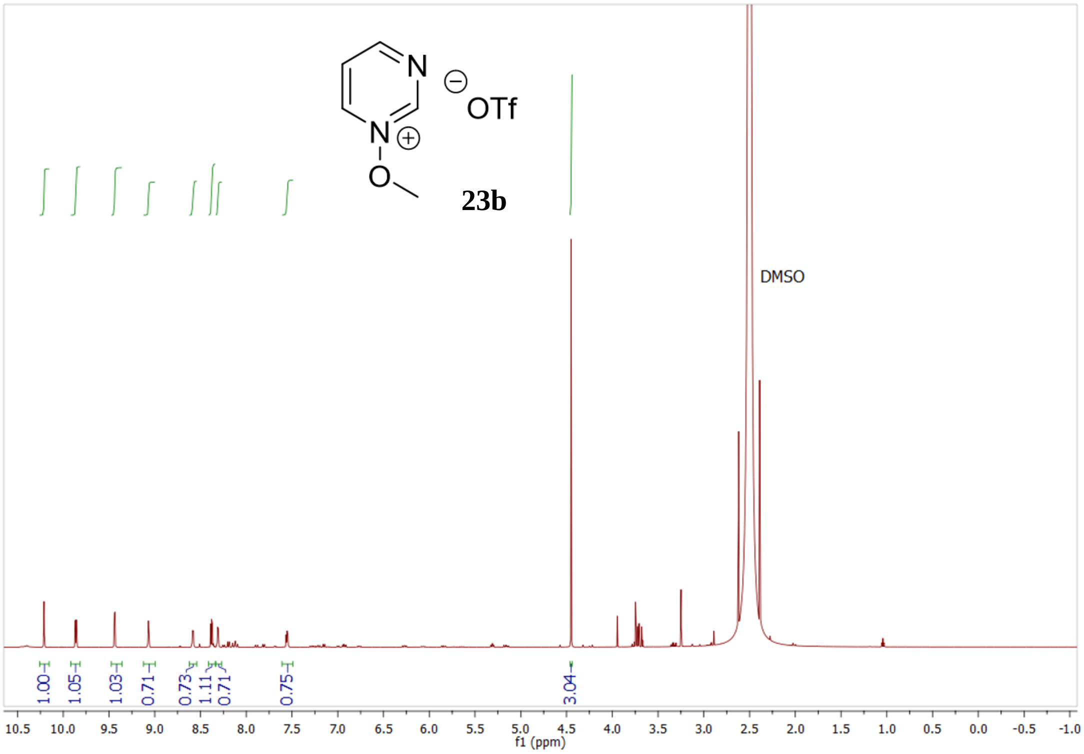

Figure S64: Full ^{1}H spectrum of **23b**, containing signals assigned to **3** in $(CH_3)_2SO$ (600 MHz) **acquired with solvent signal suppression**.

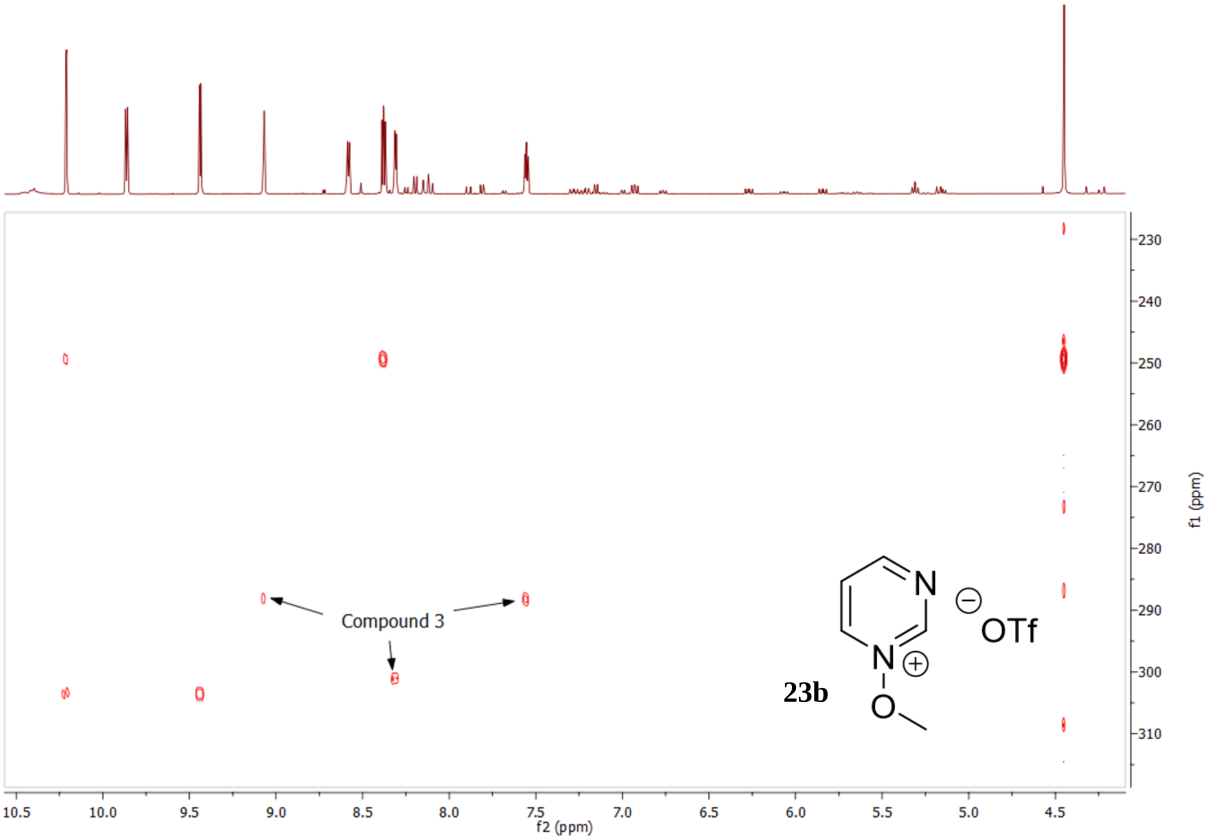

Figure S65: ^{1}H-^{15}N HMBC NMR spectrum of **23b**, containing signals assigned to **3** in $(CH_3)_2SO$ acquired with solvent signal suppression.

21b and 23b in CD_3CN (From Pyrimidine *N*-oxide (3) + MeOTf in CD_3CN)

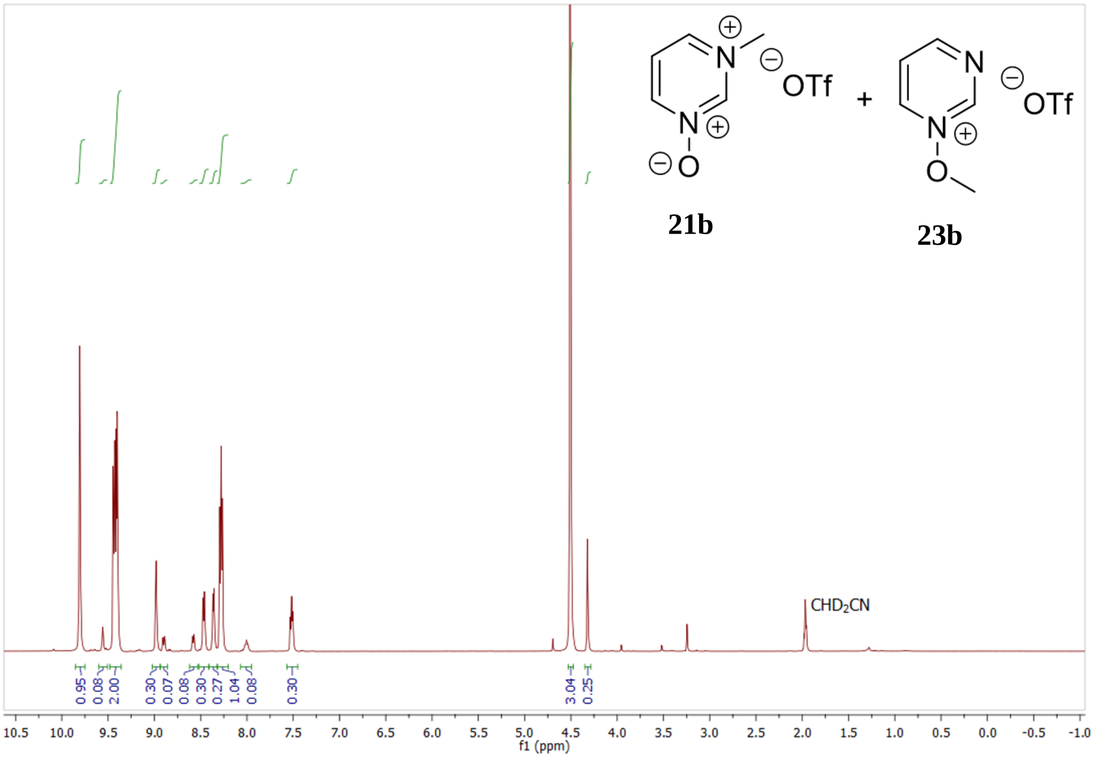

Figure S66: Full ^{1}H NMR spectrum containing signals assigned to **23b**, **21b** and **3** in CD_3CN (400 MHz).

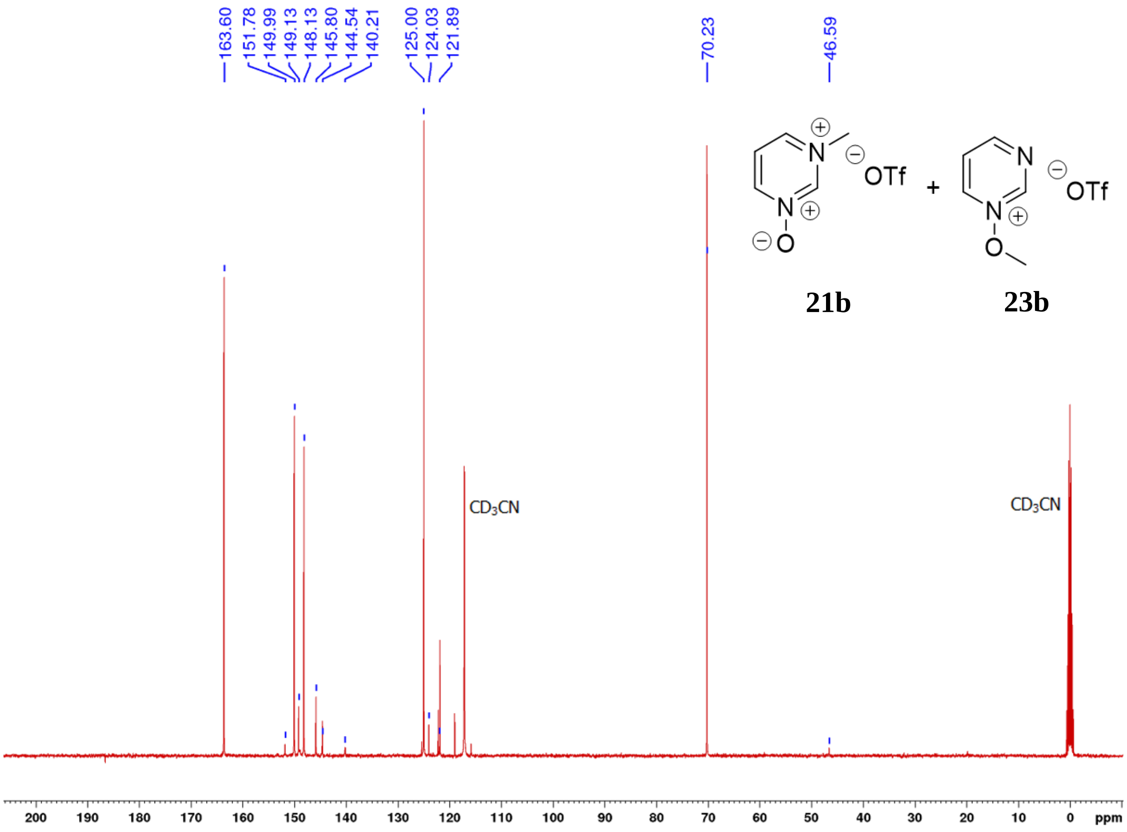

Figure S67: $^{13}C\{^{1}H\}$ NMR Spectrum of **23b**, **21b** and **3** in CD_3CN (100 MHz).

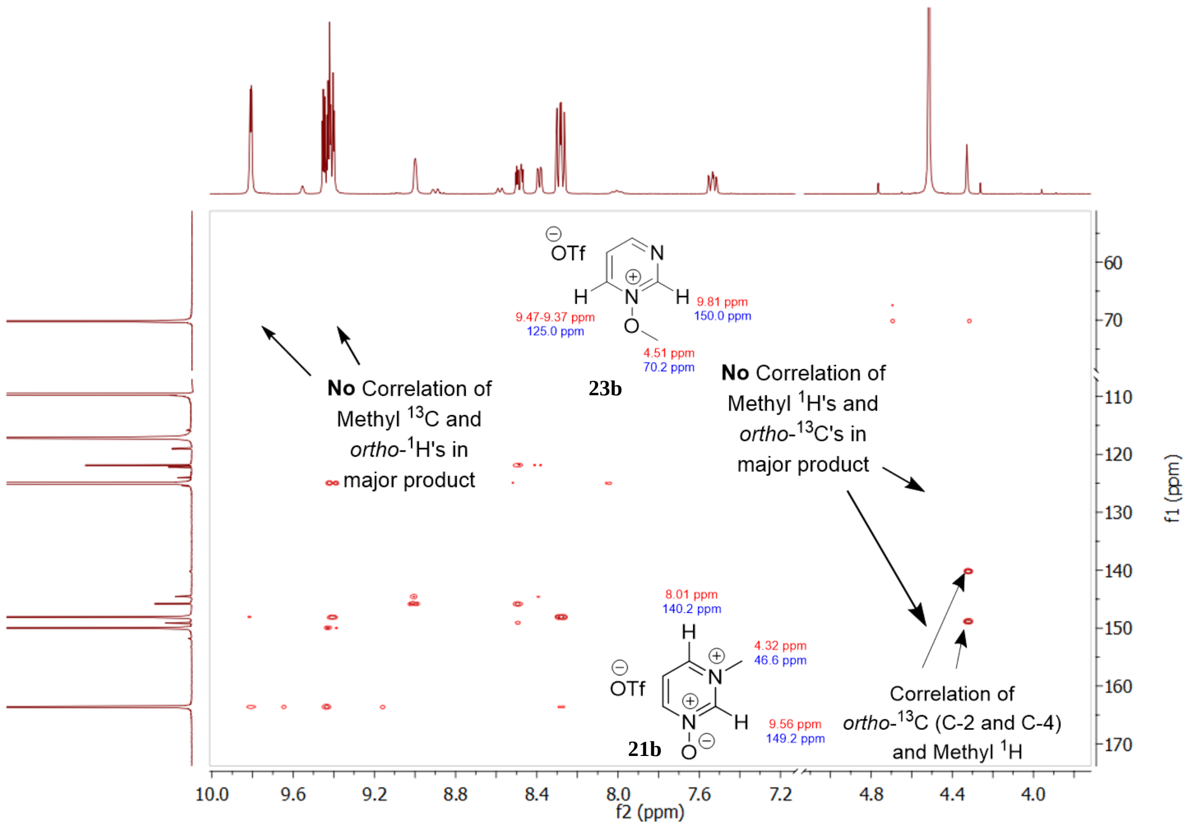

Figure S68: Section of ^{1}H-^{13}C HMBC NMR spectrum of **21b** and **23b** in CD_3CN.

23b in $(CD_3)_2SO$ (From 3 + MeOTf in CD_3CN, after solvent removal)

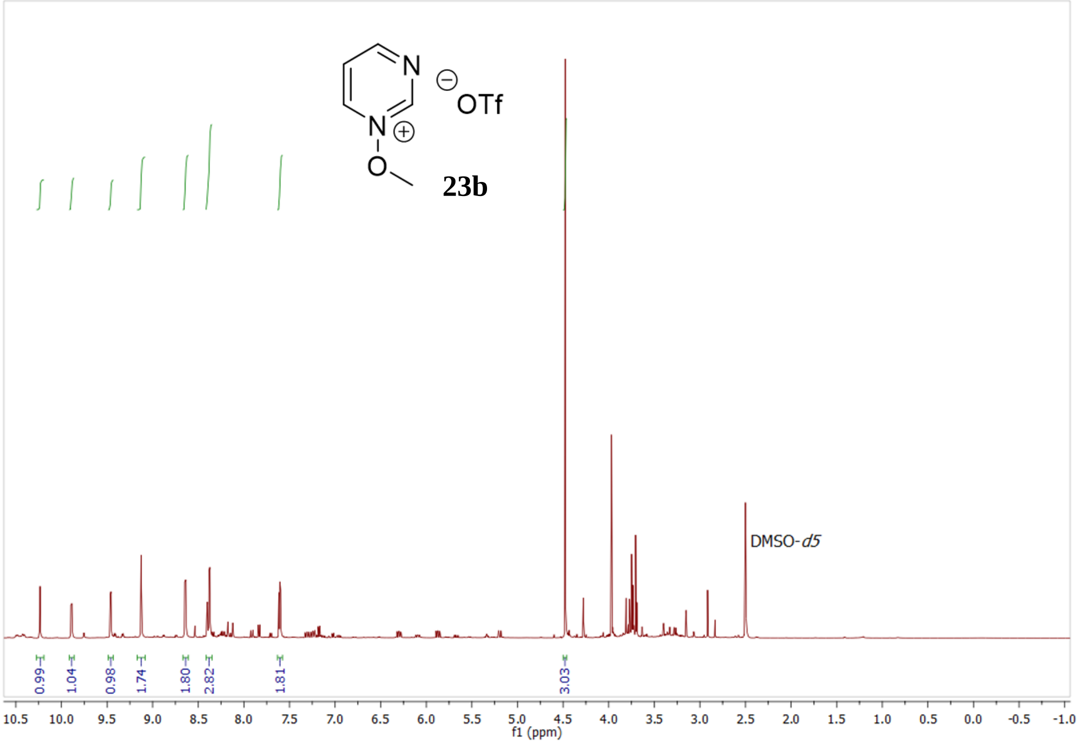

Figure S69: Full ^{1}H spectrum of **23b** and **3** in $(CD_3)_2SO$ (600 MHz). Removal of the CD_3CN caused the decomposition of **21b**. A large amount of decomposition product signals are present on the baseline.

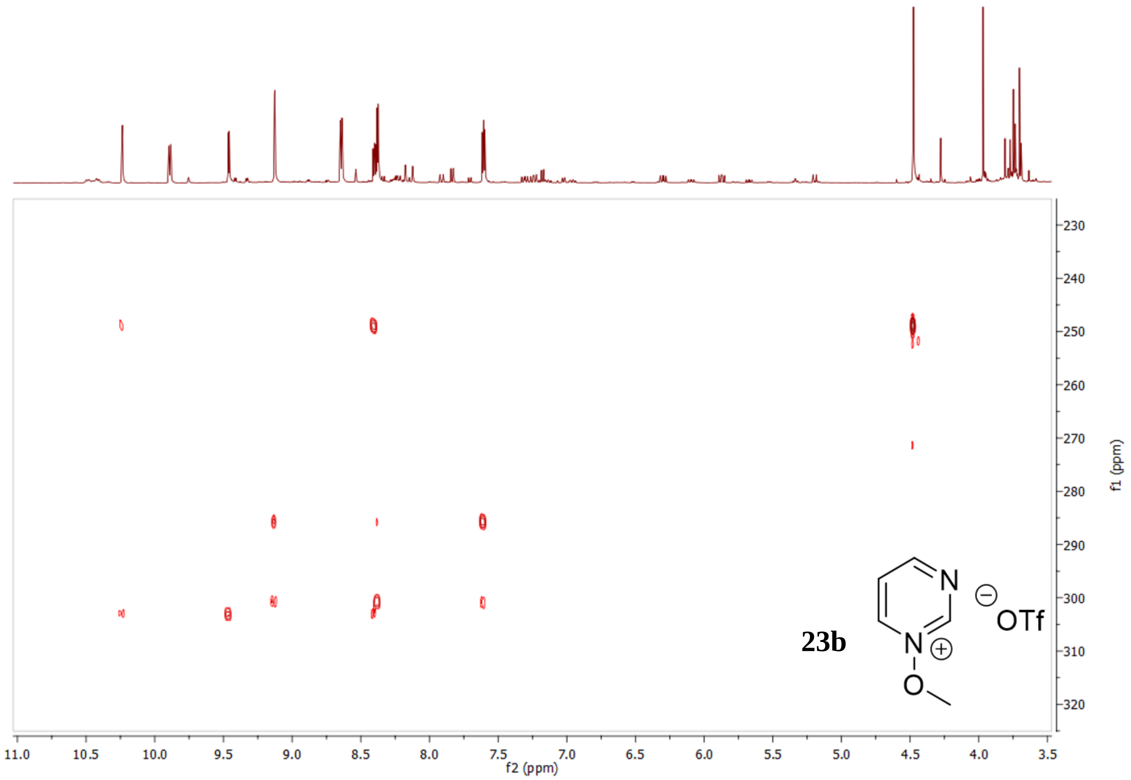

Figure S70: ^{1}H-^{15}N HMBC NMR spectrum of **23b**, containing signals assigned to **3** in $(CD_3)_2SO$.

21b and 23b in $(CD_3)_2SO$ (From Pyrimidine *N*-oxide (3) + MeOTf in $(CD_3)_2SO$)

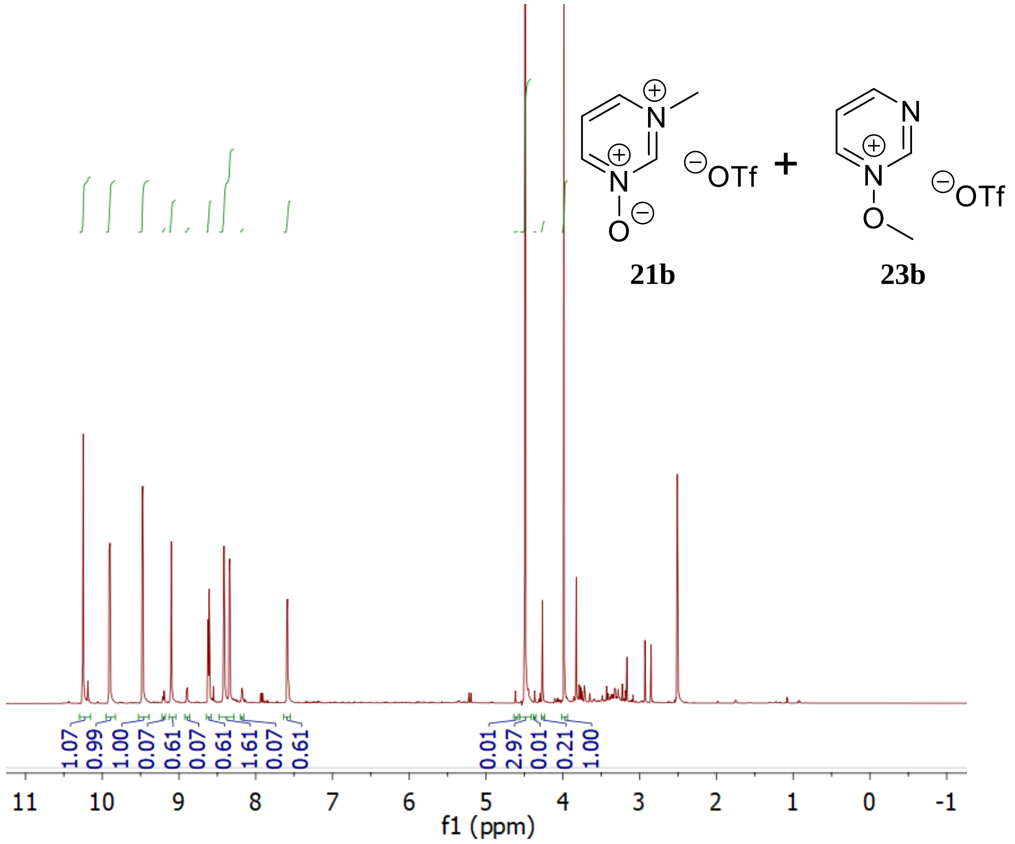

Figure S71: Full ^{1}H spectrum of containing signals assigned to **23b**, **21b** and **3** in $(CD_3)_2SO$ (600 MHz).

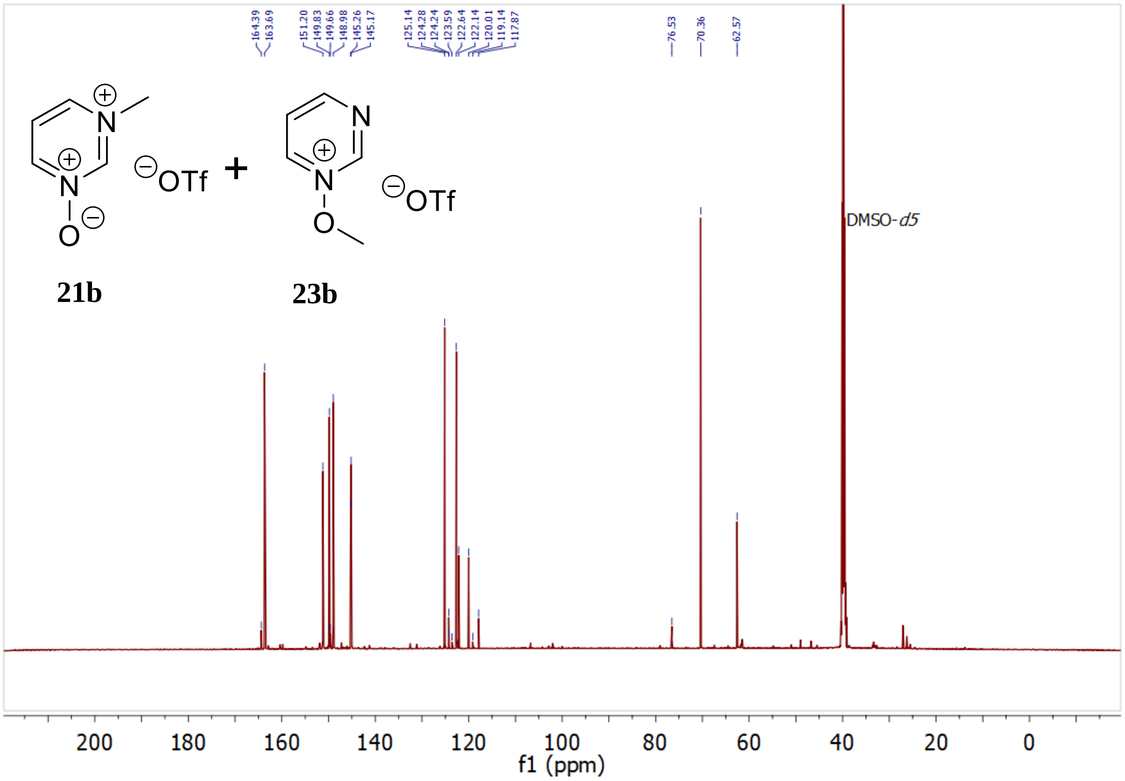

Figure S72: Full $^{13}C\{^{1}H\}$ NMR spectrum containing signals assigned to **23b** and **3** in $(CD_3)_2SO$ (600 MHz). No signals could be unambiguously assigned to the very small amount of **23b** shown to be present by the ^{1}H and ^{1}H-^{15}N HMBC NMR spectra.

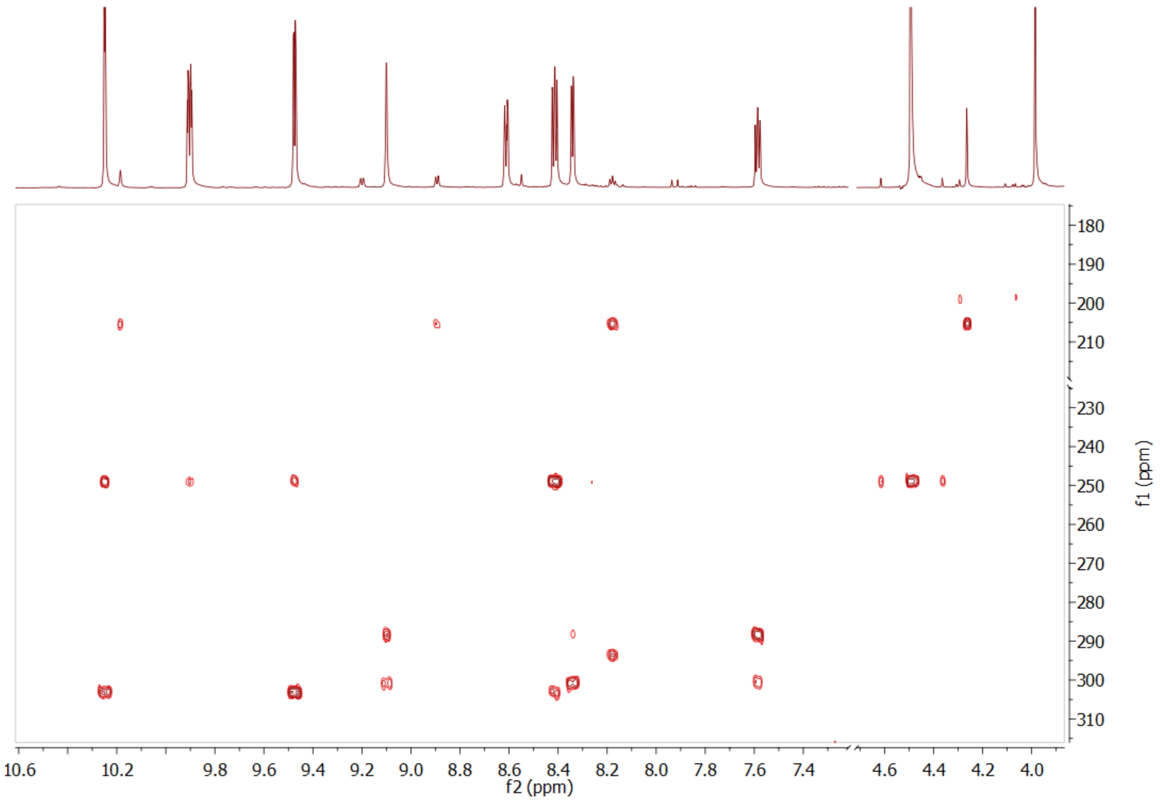

Figure S73: ^{1}H-^{15}N HMBC NMR spectrum containing signals assigned to **23b**, **21b** and **3** in $(CD_3)_2SO$. The two ^{15}N NMR corelations assigned to **21b** indicate ^{15}N resonances at 293.6 and 205.2 ppm.

Attempted Synthesis of 22 and/or 24 in CH_2Cl_2 (From 3 + 26 in CH_2Cl_2)

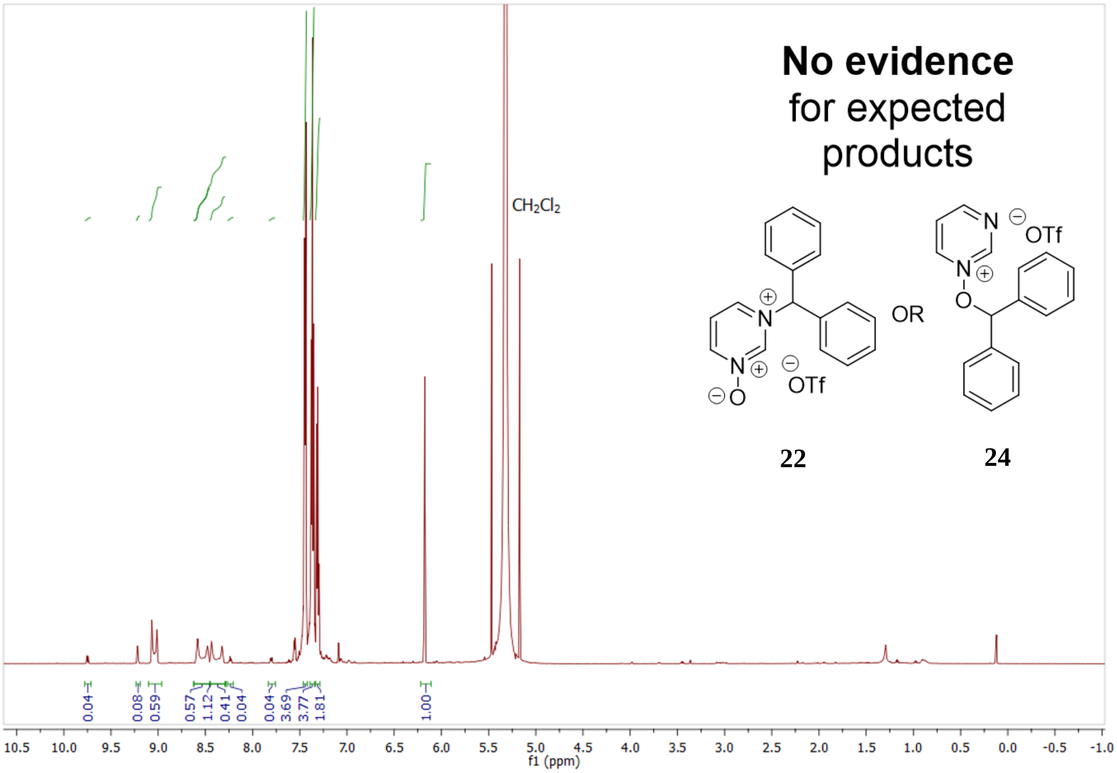

Figure S74: Full ^{1}H NMR spectrum of the crude reaction mixture from the reaction of **3** with **11** in CH_2Cl_2. Signals could not be definitively assigned to product **22** or **24**

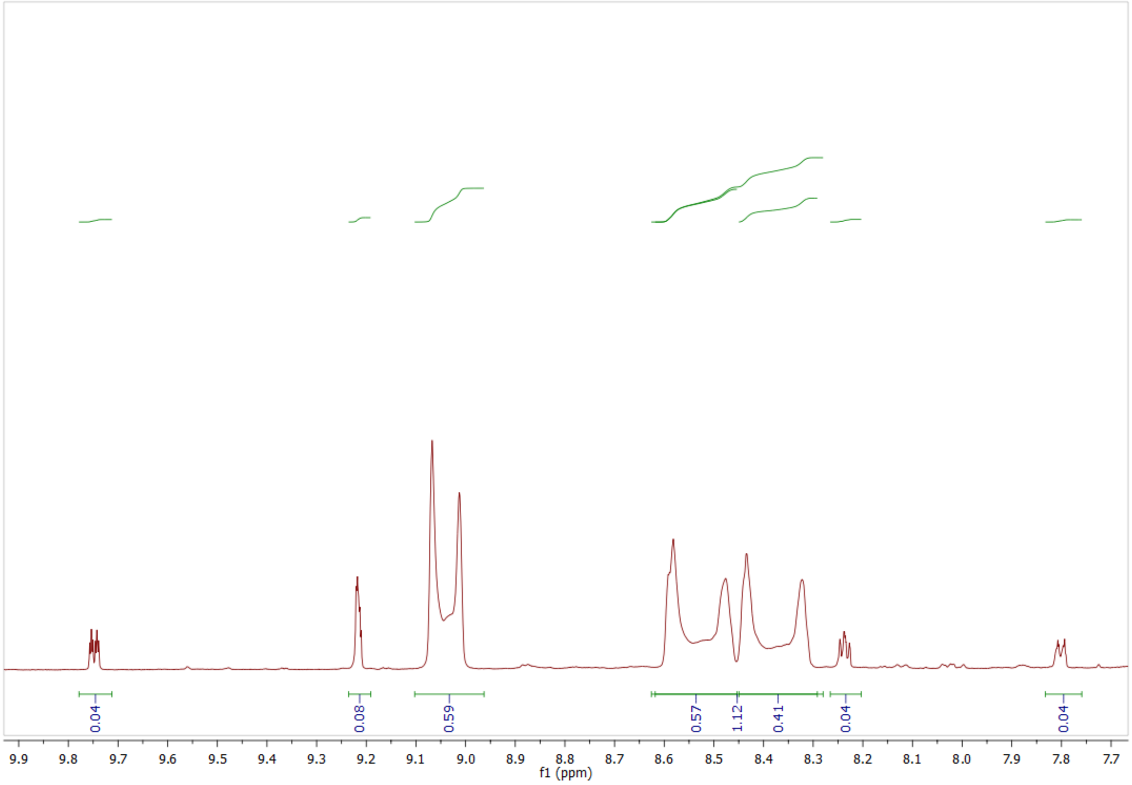

Figure S75: Expanded ^{1}H NMR spectrum of the crude reaction mixture from the reaction of **3** with **11** in CH_2Cl_2 (600 MHz). These unusually broad signals could not be definitively assigned to product **36** or **38**

Crossover experiment: 1 + MeOTf + 25 (reversibility of formation of 13b and 15b)

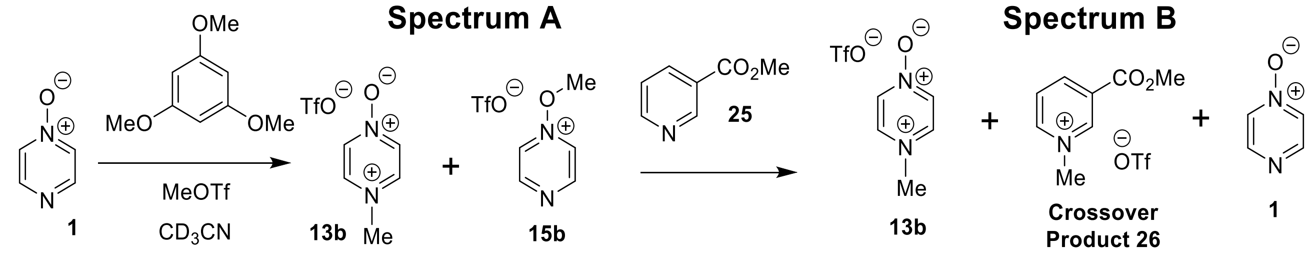

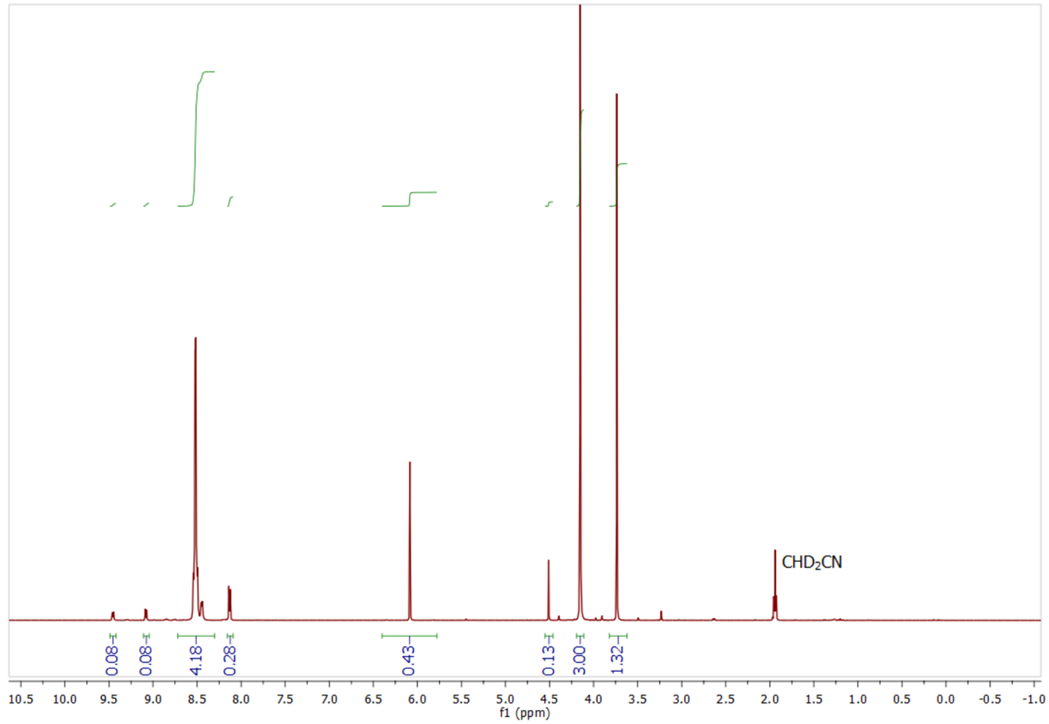

Figure S76: **Spectrum A**: Full ^{1}H NMR spectrum in CD_3CN (300 MHz) containing signals of **13b**, **15b**, **1** and 1,3,5-trimethoxybenzene.

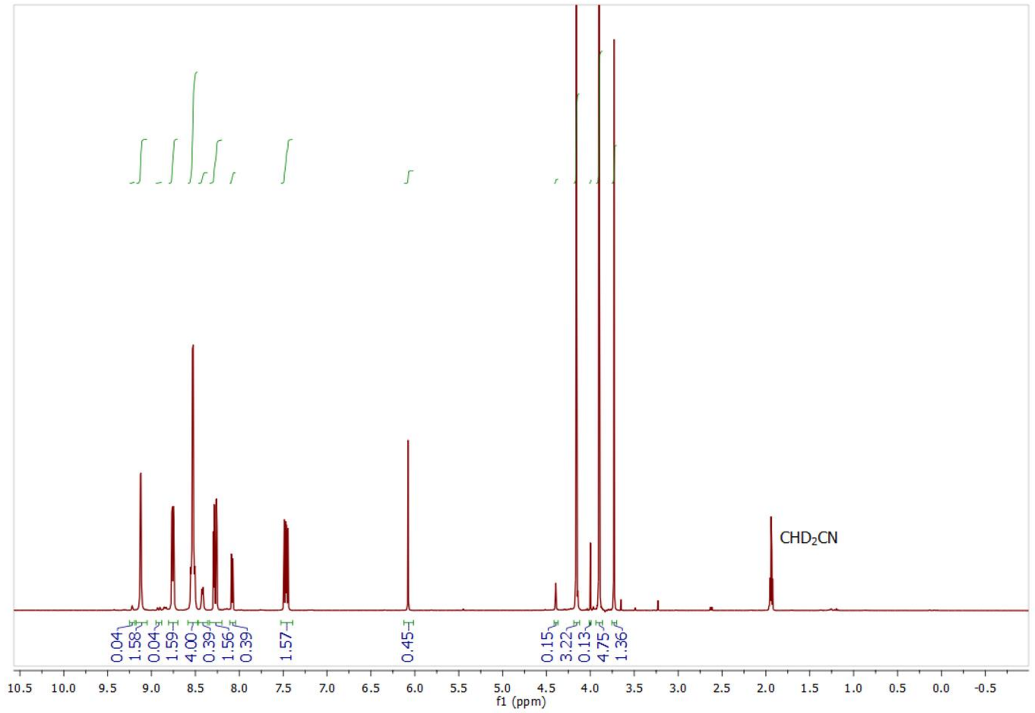

Figure S77: **Spectrum B**: Full ^{1}H NMR spectrum in CD_3CN (300 MHz) containing signals of **13b**, **25**, **26** (crossover product), **1** and 1,3,5-trimethoxybenzene.

Crossover experiment: 2 + MeOTf + 7 (reversibility of formation of 17b and 19b)

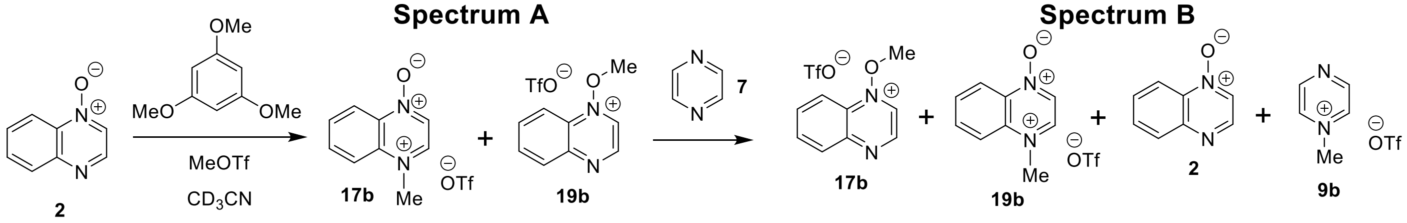

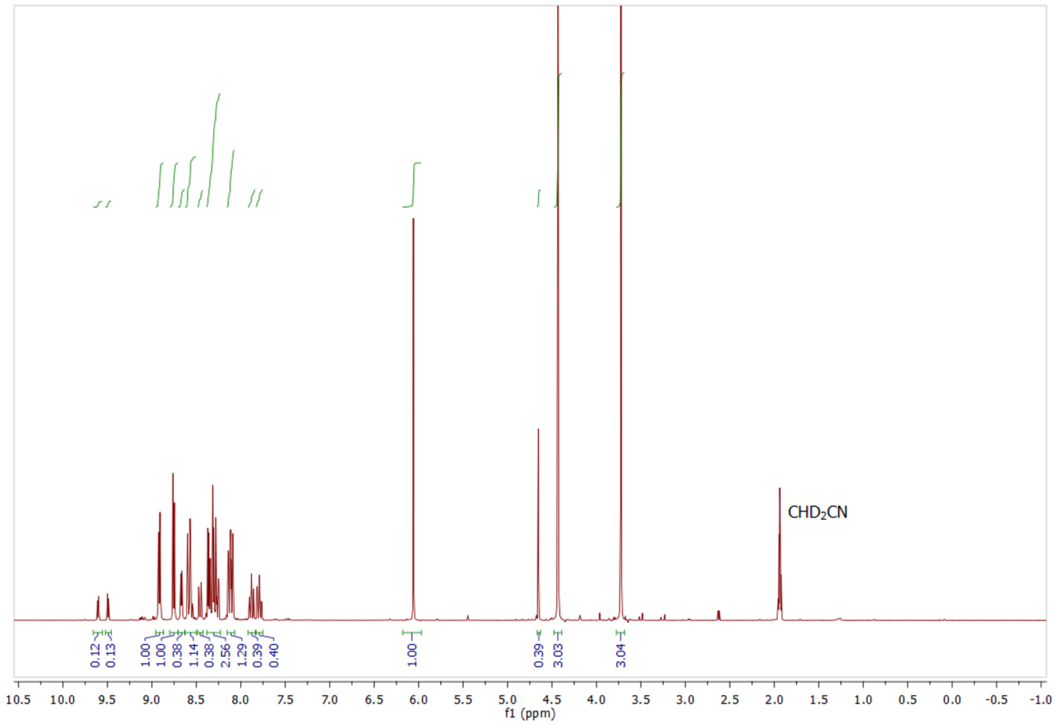

Figure S78: **Spectrum A**: Full ^{1}H NMR spectrum in CD_3CN (300 MHz) containing signals of **17b**, **19b**, **2** and 1,3,5-trimethoxybenzene.

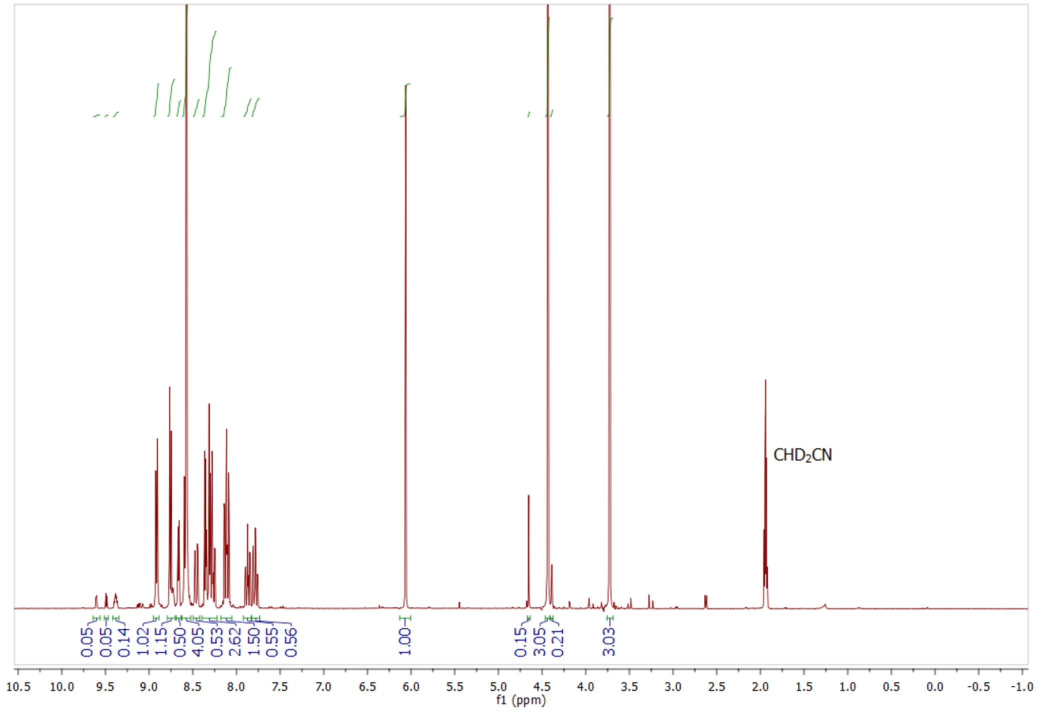

Figure S79: **Spectrum B**: Full ^{1}H NMR spectrum in CD_3CN (300 MHz) containing signals of **17b**, **7**, **9b** (crossover product**)**, **2** and 1,3,5-trimethoxybenzene.

Crossover experiment: 3 + MeOTf + 25 (reversibility of formation of 21b and 23b)

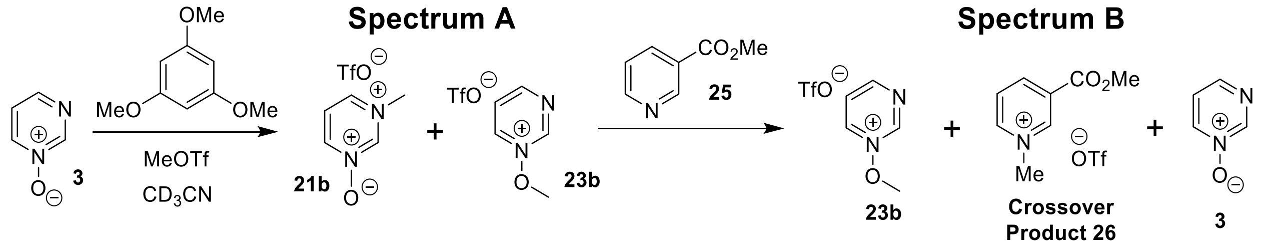

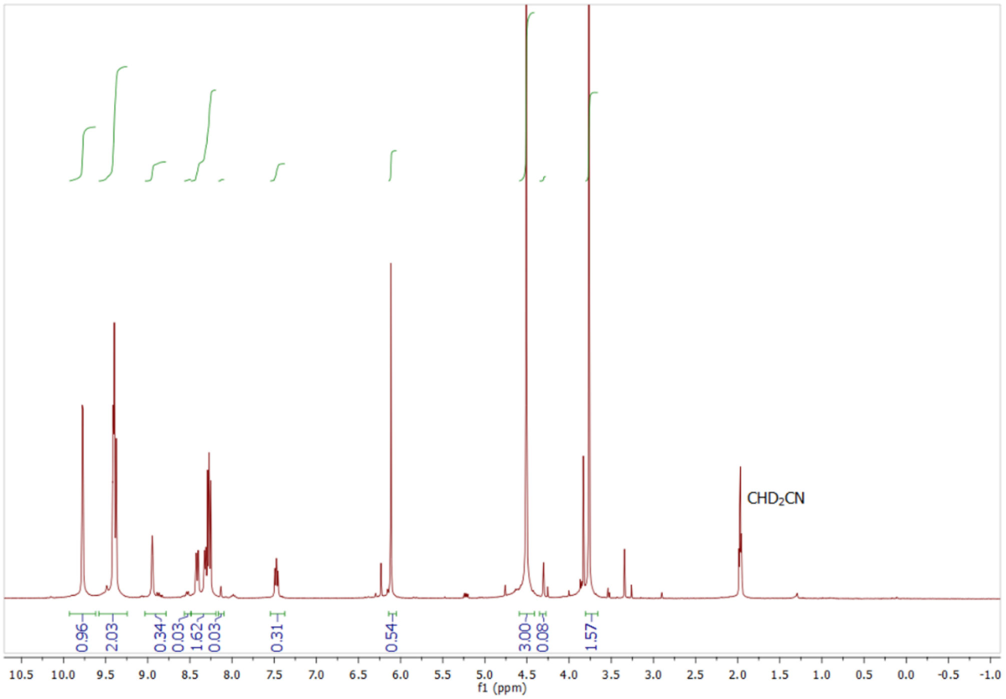

Figure S80: **Spectrum A** – Full ^1H NMR spectrum in CD_3CN (300 MHz) containing signals of **23b**, **21b**, **3** and 1,3,5-trimethoxybenzene.

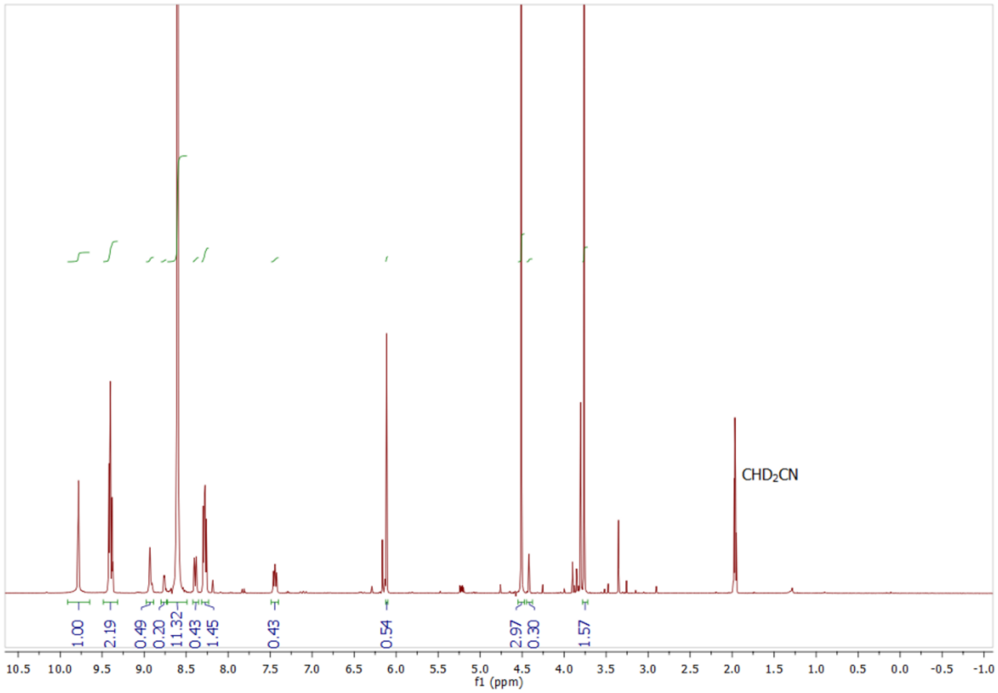

Figure S81: Spectrum B – Full ^1H NMR spectrum in CD_3CN (300 MHz) containing signals of **23b**, **3**, **9b** (crossover product) and 1,3,5-trimethoxybenzene

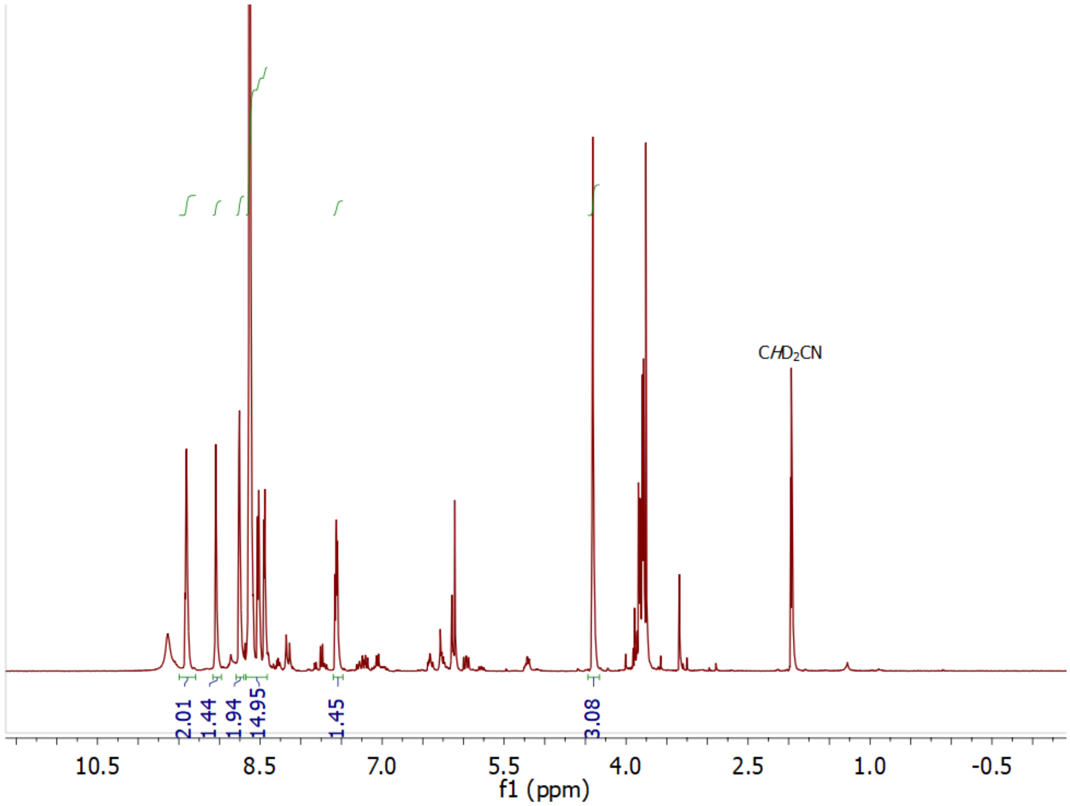

Figure S82: Spectrum C: Full ^{1}H NMR spectrum in CD_3CN (400 MHz) containing signals of **7**, **9b** (crossover product), **3** and 1,3,5-trimethoxybenzene.

Crossover experiment: 13a (From 1 + MeI) + 25 (reversibility of formation of 13a)

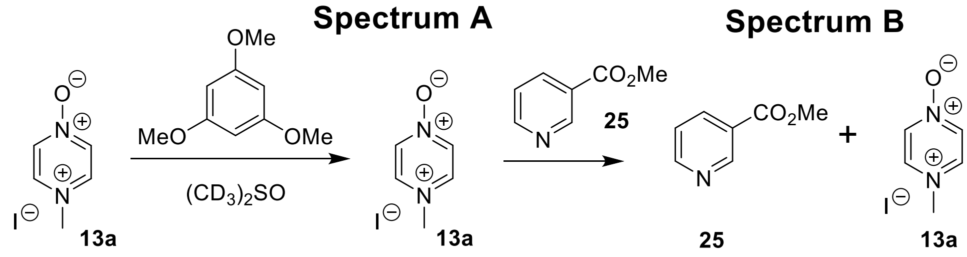

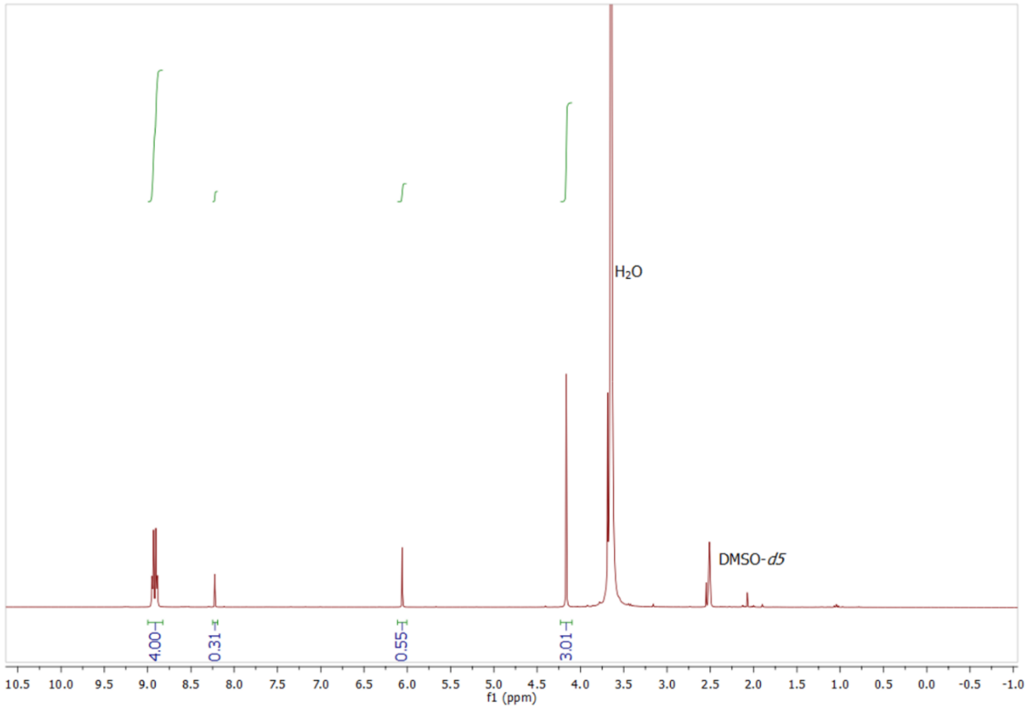

Figure S83: **Spectrum A**: Full ^{1}H NMR spectrum in $(CD_3)_2SO$ (300 MHz) containing signals of **13a** and 1,3,5-trimethoxybenzene.

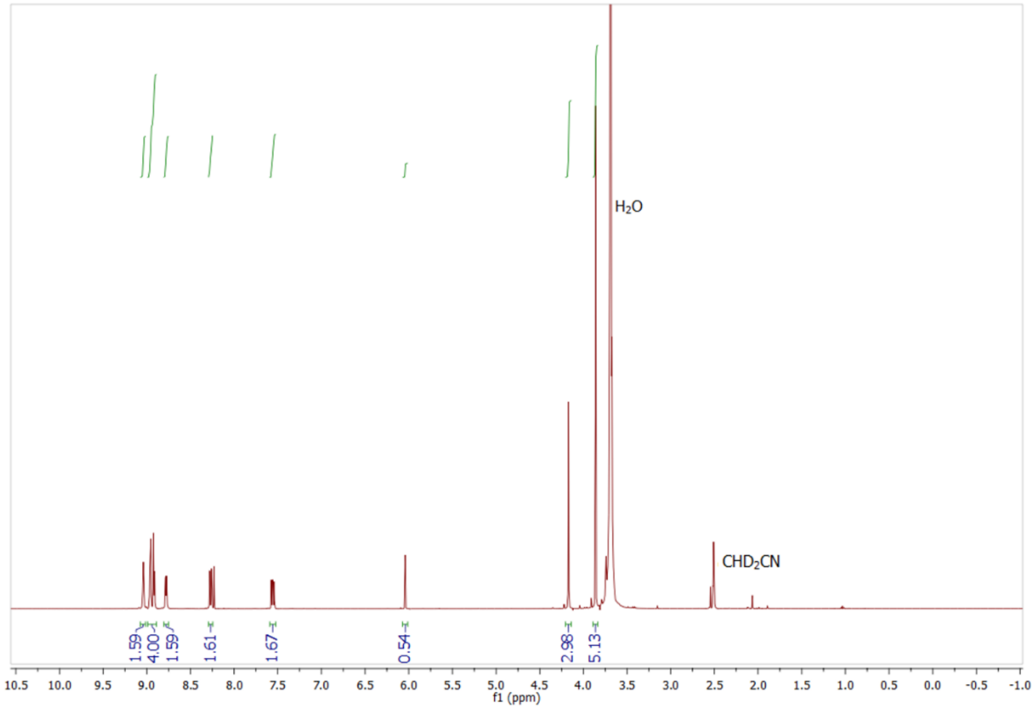

Figure S84: **Spectrum B**: Full ^{1}H NMR spectrum in $(CD_3)_2SO$ (300 MHz) containing signals of **13a**, **25**.and 1,3,5-trimethoxybenzene.

Competition experiment: 1 + 3 + benzhydrylium ion 31

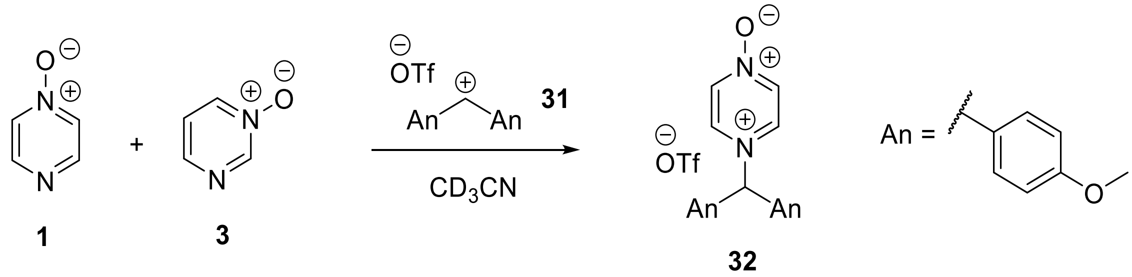

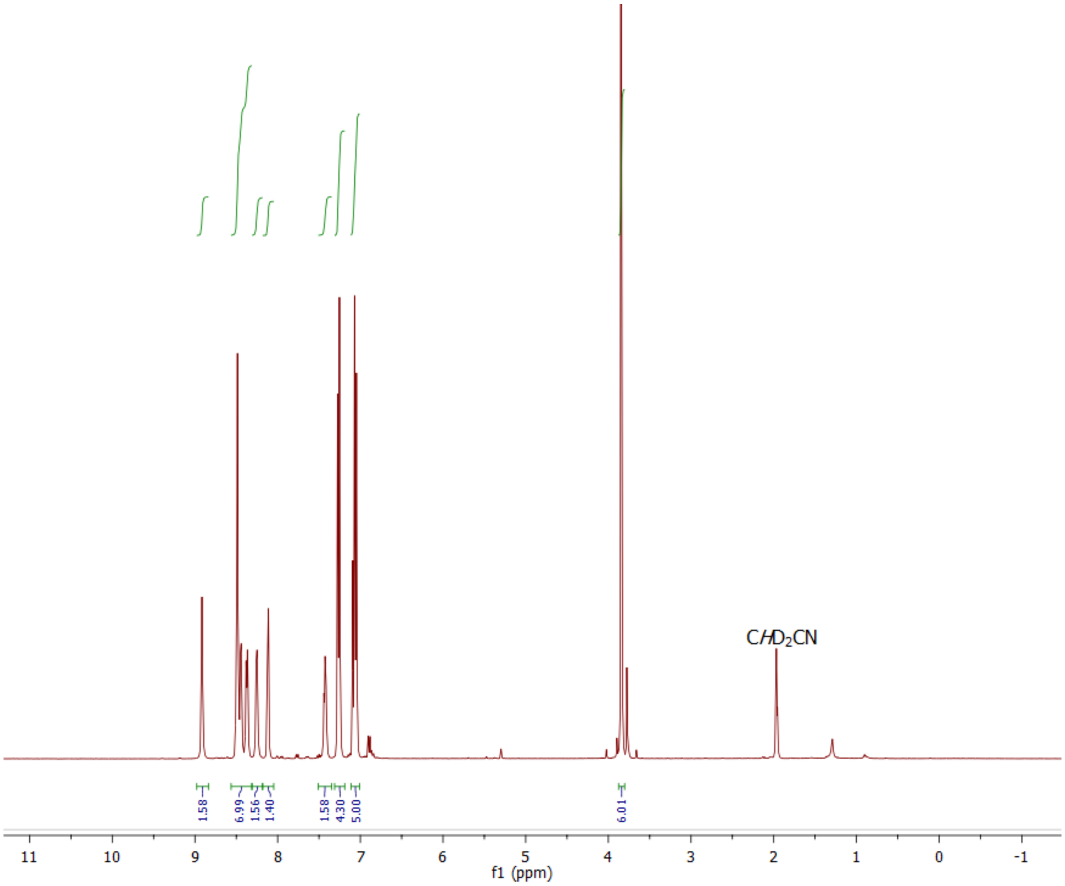

Figure S85: ^{1}H NMR spectrum in CD_3CN (400 MHz) containing signals of **1**, **3**, and **32**.

8. Calculations of Thermodynamic and Activation Parameter Values

Table S1: Activation Enthalpies ($\Delta H^{\ddagger}$, in kJ mol^{-1}), Activation Entropies ($\Delta S^{\ddagger}$, in J K^{-1} mol^{-1}), and Gibbs Energies of Activation ($\Delta G^{\ddagger}$, in kJ mol^{-1}) for Identity Methyl Transfer Reactions.

Reaction	$\Delta H^{\ddagger}$	$\Delta S^{\ddagger}$	$\Delta G^{\ddagger}$
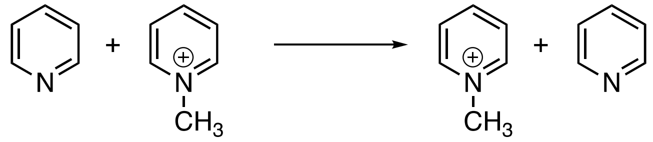	+127	–161	+175
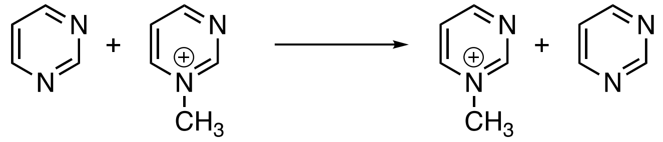	+123	–171	+175
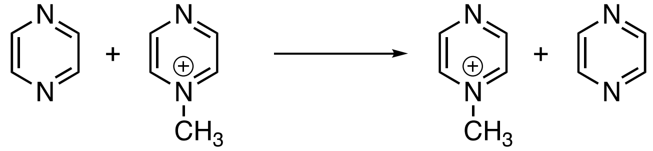	+121	–168	+171
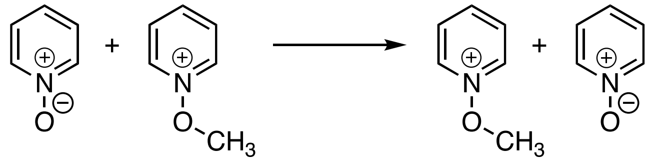	+87	–179	+141
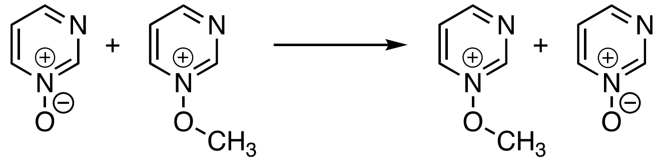	+73	–175	+125
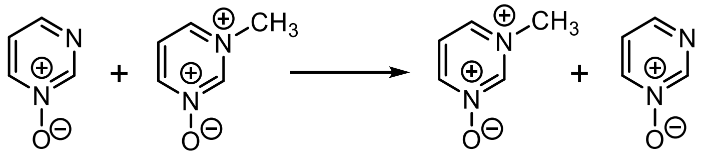	+117	–169	+167
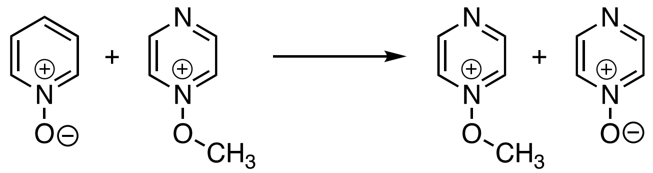	+88	–181	+142
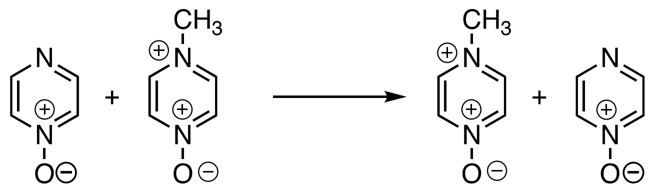	+125	–171	+176

Table S2: Values of Activation Parameters ($\Delta H^{\ddagger}$ (kJ mol^{-1}), $\Delta S^{\ddagger}$ (J K^{-1} mol^{-1})), $\Delta G^{\ddagger}$ (kJ mol^{-1})), and Thermodynamic Parameters ($\Delta_r H°$ (kJ mol^{-1}), $\Delta_r S°$ (J K^{-1} mol^{-1})), $\Delta_r G°$ (kJ mol^{-1})) for Methylation Reactions Using MeI.

Reaction	$\Delta H^{\ddagger}$	$\Delta S^{\ddagger}$	$\Delta G^{\ddagger}$	$\Delta_r H°$	$\Delta_r S°$	$\Delta_r G°$
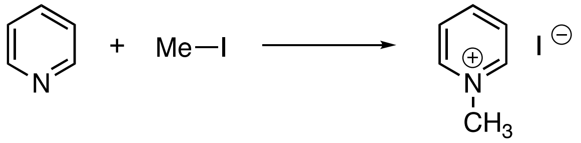	+75	–150	+120	–64	–53	–48
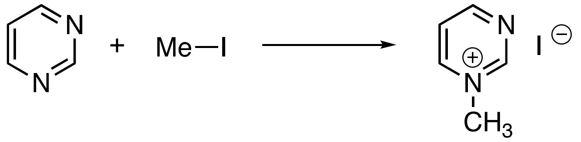	+84	–154	+130	–39	–52	–23
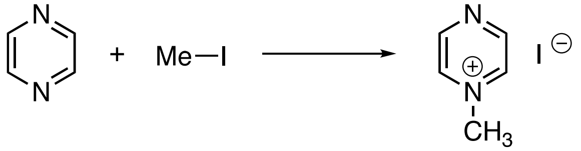	+84	–156	+131	–37	–53	–21
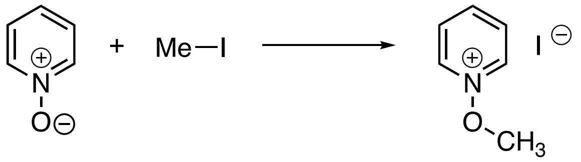	+75	–161	+123	–24	–56	–7
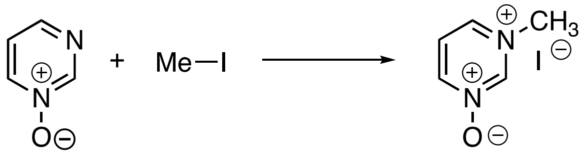	+92	–154	+138	–13	–58	+4
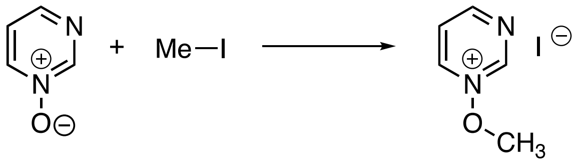	+80	–158	+127	+3	–59	+21
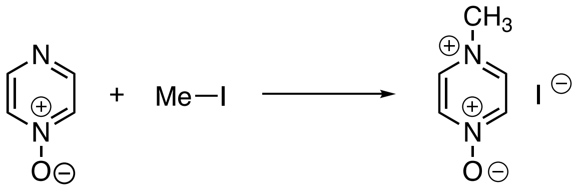	+86	–158	+133	–37	–58	–20
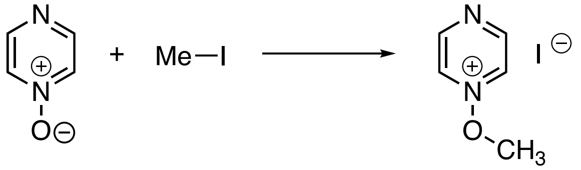	+92	–161	+140	+14	–55	+31

Table S3: Values of Activation Parameters ($\Delta H^{\ddagger}$ (kJ mol^{-1}), $\Delta S^{\ddagger}$ (J K^{-1} mol^{-1})), $\Delta G^{\ddagger}$ (kJ mol^{-1})), and Thermodynamic Parameters ($\Delta_r H°$ (kJ mol^{-1}), $\Delta_r S°$ (J K^{-1} mol^{-1})), $\Delta_r G°$ (kJ mol^{-1})) for Methylation Reactions Using MeOTf.

Reaction	$\Delta H^{\ddagger}$	$\Delta S^{\ddagger}$	$\Delta G^{\ddagger}$	$\Delta_r H°$	$\Delta_r S°$	$\Delta_r G°$
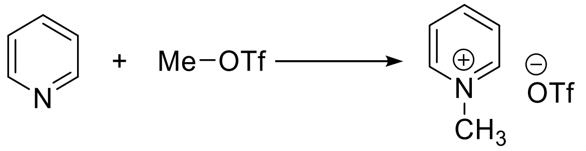	+47	–164	+96	–117	+1	–117
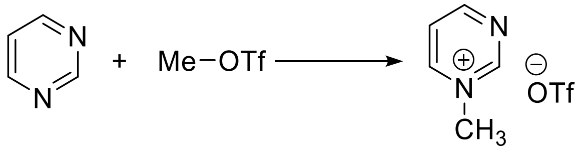	+56	–171	+107	–91	+1	–91
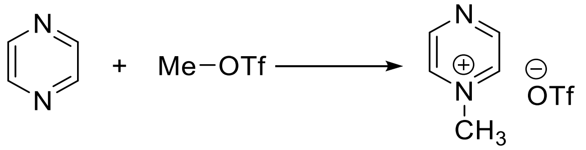	+55	–174	+107	–90	0	–90
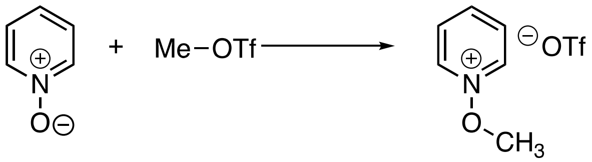	+44	–181	+97	–76	–3	–75
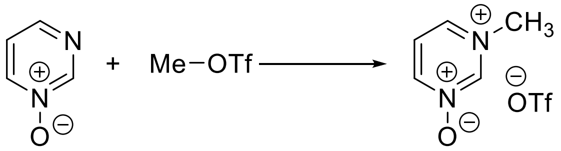	+62	–173	+113	–66	–5	–64
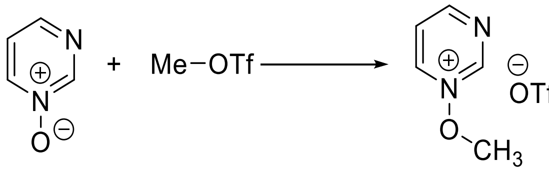	+51	–172	+103	–49	–6	–48
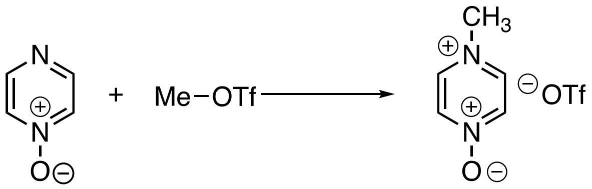	+56	–174	+108	–90	–5	–88
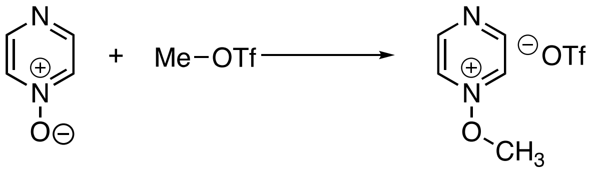	+61	–180	+115	–38	–2	–38

9. Calculation of Marcus Intrinsic Barriers

Let us consider an S_N2 reaction of a nucleophile (Nu) with an alkyl electrophile such as MeX (e.g. X = I, OTf, etc.), with a Gibbs energy of activation $\Delta G^‡$ and a standard Gibbs energy of reaction $\Delta_r G°$. Such a reaction can be thought of as a methyl group transfer from X^- to the nucleophile (Scheme S1a). We wish to calculate $\Delta G^‡$ using the Marcus equation (equation 1 in the main article), reproduced here:

$$\Delta G^{‡} = \Delta G_0^{‡} + \frac{\Delta_r G°}{2} + \frac{(\Delta_r G°)^2}{16\Delta G_0^{‡}} \qquad (1)$$

In order to access the value of the Marcus intrinsic barrier ($\Delta G_0^‡$) for the S_N2 reaction of Nu + MeX, one must first determine the Gibbs energies of activation for the reactions shown in Scheme S1b and S1c. These methyl group transfer reactions are identity reactions since the products and the reactants are the same. They are thermoneutral, i.e. $\Delta_r G° = 0$ for each one. The Gibbs energy of activation for methyl transfer from Me—X to X^- is $\Delta G^‡_B$, and the Gibbs energy of activation for methyl transfer from Nu^+—Me to Nu is $\Delta G^‡_C$.

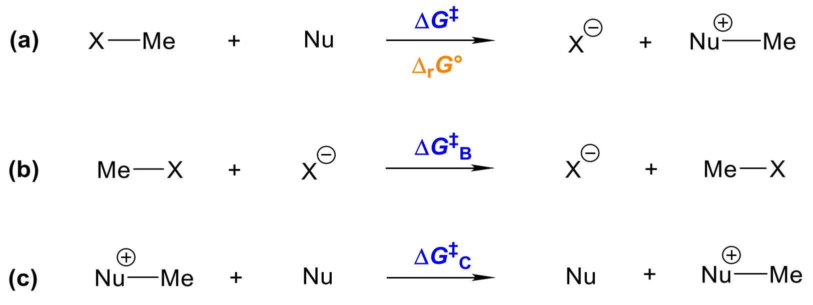

Scheme S1. **(a)** Methyl transfer reaction from MeX to Nu, with Gibbs energy of activation = $\Delta G^‡$ and $\Delta_r G° \neq 0$; **(b)** Methyl transfer identity reaction from MeX to X^-, with Gibbs energy of activation = $\Delta G^‡_B$ and $\Delta_r G° = 0$; **(c)** Methyl transfer identity reaction from Nu^+—Me to Nu, with Gibbs energy of activation = $\Delta G^‡_C$ and $\Delta_r G° = 0$.

Using the Gibbs energies of activation of the identity reactions shown in Scheme S1b and S1c, the intrinsic barrier ($\Delta G_0^‡$) for the reaction of the nucleophile (Nu) with MeX (Scheme S1a) can be calculated using equation 2:

$$\Delta G_0^{‡} = \frac{1}{2}\left(\Delta G_B^{‡} + \Delta G_C^{‡}\right) \qquad (2)$$

i.e. $\Delta G_0^‡$ for the methylation of the nucleophile is taken to be the average of the Gibbs energies of activation of the identity reactions shown in Scheme S1b and S1c.

In this study, we have calculated values of Gibbs energies of activation for methyl group transfer identity reactions of the type shown in Scheme S1b and S1c for nucleophiles **1** and **3** and also iodide and triflate (see Table S1). These calculations were done at the DLPNO-CCSD(T)/def2-TZVPPD/SMD//M06-2X-D3/6-311+G(d,p)/SMD level of theory.

Table S4. $\Delta G^{\ddagger}$ values for methyl transfer identity reactions of **1** and **3** at both the N and O nucleophilic sites, and of iodide and triflate. [a]

Reaction	Compound Number	Site of Methylation	$\Delta G^{\ddagger}$ of Identity Reaction
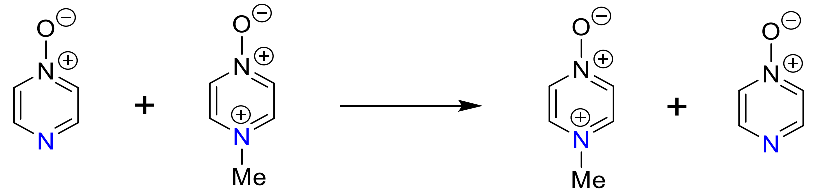	**1**	N	**176**
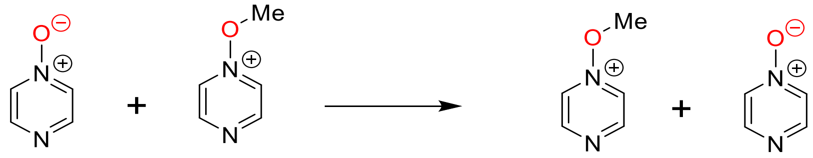	**1**	O	**142**
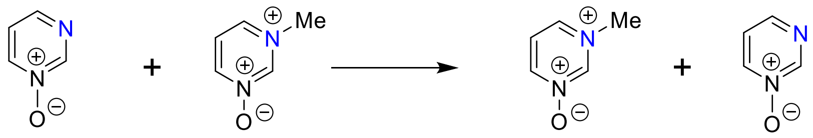	**3**	N	**167**
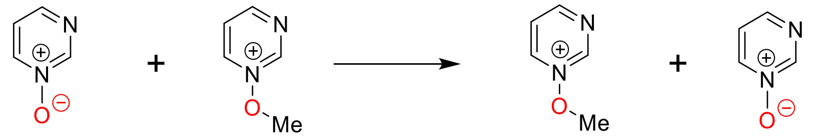	**3**	O	**125**
$I{-}Me + I^{\ominus} \longrightarrow I^{\ominus} + Me{-}I$	Iodide	I	**112**
$TfO{-}Me + {}^{\ominus}OTf \longrightarrow TfO^{\ominus} + Me{-}OTf$	Triflate	O	**123**

[a] Gibbs energy values were calculated at the DLPNO-CCSD(T)/def2-TZVPPD/SMD(CH3CN)//M06-2X-D3/6-311+G(d,p)/SMD(CH3CN) level of theory.

Calculation of $\Delta G^‡$ using $\Delta G_0^‡$ values in the Marcus Equation

Using the $\Delta G^‡$ values calculated for the methyl transfer identity reactions (Table S4), values of the intrinsic barrier ($\Delta G_0^‡$) were calculated for each of the reactions of compounds **1** and **3** with MeI and MeOTf using equation 2. These $\Delta G_0^‡$ values are shown in Table S5 on pg. S90 (these values are also shown in Table 4 of the main article).

The value calculated for $\Delta G_0^‡$ for each reaction was substituted into the Marcus equation (equation 1) along with the $\Delta_r G°$ value calculated for the reaction in question (these values are shown in Table 3 in the main article, and reproduced here in Table S5), enabling calculation of a value for $\Delta G^‡$ according to the Marcus equation for the reaction of Nu + MeX. For ambident nucleophiles **1** and **3**, there are two $\Delta G^‡$ values – one for reaction at each of the nucleophilic sites of the ambident nucleophile. For these nucleophiles, the product ratio predicted by the Marcus calculations just described was obtained using equation 3

$$\frac{k_{\mathrm{N}}}{k_{\mathrm{O}}} = e^{-\left(\frac{\Delta\Delta G^‡}{RT}\right)} = e^{-\left(\frac{\Delta G^‡(\mathrm{N}) - \Delta G^‡(\mathrm{O})}{RT}\right)} \qquad (3)$$

where

k_{N} and $\Delta G^‡(\mathrm{N})$ are the rate constant (L mol^{-1} s^{-1}) and Gibbs energy of activation (kJ mol^{-1}), respectively, for N-methylation,

k_{O} and $\Delta G^‡(\mathrm{O})$ are the rate constant (L mol^{-1} s^{-1}) and Gibbs energy of activation (kJ mol^{-1}), respectively, for O-methylation,

R is the universal gas constant, and the temperature, T, was set to 298 K.

The product ratio calculated in this manner for methylation of **1** by MeOTf was 90:10 ± 2 (in favour of N-methylation), and for **1** + MeI the ratio was 97:3 ± 2 (O-methylation was also calculated to be reversible, i.e. $\Delta_r G° > 0$; experimentally no O-methylation is observed). The product ratio calculated for methylation of **3** by MeOTf was 0.4 : 99.6 ± 2 (in favour of O-methylation), and for **3** + MeI the ratio was calculated to be 1:99 ± 2 (both O and N-methylation were calculated to be reversible, i.e. $\Delta_r G° > 0$, and no product formation was observed experimentally). Further detail on these calculations is given below, in Tables S6 and S7.

Much of the information contained in Table S5 is reproduced from Table 4 in the main article. This was done by design to allow straightforward comparison of the extra results included there (from calculations done using the Zhu equation – see below) with results derived from the Marcus equation, and direct DFT calculations.

Calculation of $\Delta G^{\ddagger}$ using the Zhu Equation

Zhu and co-workers have developed an alternative to the Marcus equation to rationalize the outcomes of hydride transfer reactions.[28] Here, we have adapted the Zhu equation to apply to methyl group transfer reactions. Our adaptation of the Zhu equation is shown in equation 4:

$$\Delta G^{\ddagger} = \frac{1}{2}\left(\Delta G^{\ddagger}_{\text{XMe/X}} + \Delta G^{\ddagger}_{\text{NuMe/Nu}}\right) + \frac{\Delta_r G^{\circ}}{2} \qquad (4)$$

where $\Delta G^{\ddagger}_{\text{XMe/X}} = \Delta G^{\ddagger}_{\text{B}}$ from Scheme S1b on pg. S86, $\Delta G^{\ddagger}_{\text{NuMe/Nu}} = \Delta G^{\ddagger}_{\text{C}}$ from Scheme S1c on pg. S86 (i.e. $\Delta G^{\ddagger}_{\text{XMe/X}}$ and $\Delta G^{\ddagger}_{\text{NuMe/Nu}}$ are the Gibbs energies of activation for the methyl group transfer identity reactions shown in Scheme S1b and S1c, for which $\Delta_r G^{\circ} = 0$), and $\Delta G^{\ddagger}$ and $\Delta_r G^{\circ}$ are, respectively, the Gibbs energy of activation and standard Gibbs energy of reaction for the methyl group transfer reaction shown in Scheme S1a on pg. S86.

The first term in equation 4 (involving the activation barriers for the identity reactions) is identical to the expression for the Marcus intrinsic barrier shown in equation 2, and the second term is identical to the second term of the Marcus equation (equation 1 in the main article). So the Marcus equation and Zhu equation differ only in the exclusion of the quadratic term of the Marcus equation from the latter equation. We have calculated $\Delta G^{\ddagger}$ values using the adapted Zhu equation (equation 4) using our computational data from the methyl transfer identity reactions (values from Table S4) along with our directly calculated $\Delta_r G^{\circ}$ values for the methylation reactions of **1** and **3** (shown in Table S5). These $\Delta G^{\ddagger}$ values, calculated according to the adapted Zhu equation (shown in Table S5), are essentially identical to the values calculated using equation 1. This is because the quadratic term of equation 1 is very small in all reactions investigated here due to the relatively small $\Delta_r G^{\circ}$ values of these reactions. Consequently, there is close agreement between the $\Delta G^{\ddagger}$ values calculated using equation 4 (Zhu equation) and equation 1 (Marcus equation) and those calculated directly at the DLPNO-CCSD(T)/def2-TZVPPD/SMD//M06-2X-D3/6-311+G(d,p)/SMD level of theory. Naturally, therefore, the product ratios determined using these three different methods of calculation agree quite closely. All of these methods of determining the product ratios are close to the true values observed experimentally, as discussed in the main article.

Table S5. Values of intrinsic barriers ($\Delta G_0^‡$) for methylation reactions of nucleophiles **1** and **3**, and derived values of $\Delta G^‡$ for methylation reactions of these nucleophiles (Scheme S1a with Nu = N or O nucleophilic site of **1** or **3**) calculated using Marcus equation (equation 1) and Zhu equation (equation 4) by employing values of $\Delta_r G°$ from Table 4 of the main article (and reproduced here). The site of methylation of each nucleophile is indicated by an arrow. The Gibbs energy values have units of kJ mol^{-1}.

$$\text{Nu} \quad + \quad \text{Me—X} \longrightarrow [\text{Nu–Me}]\ \text{X}$$

Nucleophile (Nu)	#	Product . No.	X	$\Delta G^‡$ Me transfer MeX + X$^-$	$\Delta G^‡$ Me transfer MeNu$^+$ + Nu	$\Delta G_0^‡$	$\Delta_r G°$	Marcus $\Delta G^‡$	Zhu $\Delta G^‡$	DFT $\Delta G^‡$
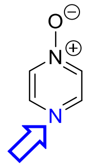	(i)	**13b**	OTf	123	176	+149.5	−88	+108.7	+105.5	+108
	(ii)	**13a**	I	112	176	+144	−20	+134.2	+134	+133
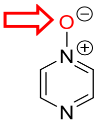	(iii)	**15b**	OTf	123	142	+132.5	−38	+114.3	+113.5	+115
	(iv)	**15a**	I	112	142	+127.0	+31	+143.0	+142.5	+140
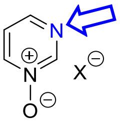	(v)	**21b**	OTf	123	167	+145.0	−64	+114.8	+113	+113
	(vi)	**21a**	I	112	167	+139.5	+4	+141.5	+141.5	+138
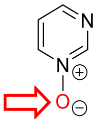	(vii)	**23b**	OTf	123	125	+124.0	−48	+101.2	+100	+103
	(viii)	**23a**	I	112	125	+118.5	+21	+129.2	+129	+127

Example calculation to obtain the product ratio predicted by the Marcus calculations

Table S6. Values of activation barriers ($\Delta G^{\ddagger}$) for methylation reactions of nucleophiles **1** and **3** with MeOTf, with calculations of the terms used in equation 3. The Gibbs energy values have units of kJ mol^{-1}.

Nucleophile (Nu) and Product Number		Entry	Site of Methylation	Marcus $\Delta G^{\ddagger}$	$\Delta\Delta G^{\ddagger}$	$\left(\frac{\Delta\Delta G^{\ddagger}}{RT}\right)$
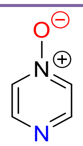	**1**	(i)	N	+108.7	−5.44	−2.20
		(ii)	O	+114.3		
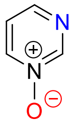	**3**	(iii)	N	+114.8	+13.6	+5.49
		(iv)	O	+101.2		

In the case of **1** + MeOTf:

$$\frac{k_{\mathrm{N}}}{k_{\mathrm{O}}} = e^{-(-2.20)} = 9.03$$

$$\frac{k_{\mathrm{N}}}{k_{\mathrm{O}}} = \frac{9.03}{1 + 9.03} = 90.0\% \;\; \text{N-methylation}$$

Table S7. Calculated ratios of N vs O methylation for reactions of nucleophiles **1** and **3** with MeOTf and MeI. The Gibbs energy values have units of kJ mol^{-1}.

Nucleophile (Nu) and Product Number	Entry	Method	Electrophile	$\Delta\Delta G^{\ddagger}$	$\left(\frac{\Delta\Delta G^{\ddagger}}{RT}\right)$	N/O Ratio
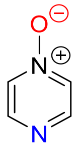 **1**	(i)	Marcus	MeOTf	−5.4	−2.2	90 : 10
	(ii)	Marcus	MeI	−8.8	−3.6	97 : 3
	(iii)	Zhu	MeOTf	−8.0	−3.2	96 : 4
	(iv)	Zhu	MeI	−8.5	−3.4	97 : 3
	(v)	DFT	MeOTf	−7.0	−2.8	94 : 6
	(vi)	DFT	MeI	−7.0	−2.8	94 : 6
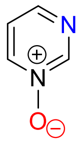 **3**	(vii)	Marcus	MeOTf	+13.6	+5.5	0.4 : 99.6
	(viii)	Marcus	MeI	+12.3	+5.0	0.7 : 99.3
	(ix)	Zhu	MeOTf	+13.0	+5.3	0.5 : 99.5
	(ix)	Zhu	MeI	+12.5	+5.0	0.6 : 99.4
	(x)	DFT	MeOTf	+10.0	+4.0	1.7 : 98.3
	(xi)	DFT	MeI	+11.0	+4.4	1.2 : 98.8

10. Charge Density Calculations

Table S8. Charge density calculations using different computational methods (NBO,[13] Merz-Singh-Kollman,[14] ChelpG,[15] AIM[16]) at the M06-2X-D3/6-311+G(d,p)/SMD(CH_3CN) level of theory.

Nucleophile	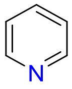 **28**	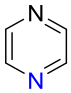 **7**	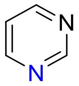 **27**	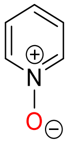 **8**	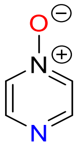 **1**		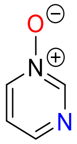 **3**	
Nucleophilic Site	N	N	N	O	N	O	N	O
NBO	−0.514	−0.460	−0.528	−0.656	−0.490	−0.589	−0.484	−0.648
Merz-Singh-Kollman	−0.842	−0.564	−0.917	−0.719	−0.732	−0.679	−0.704	−0.300
ChelpG	−0.719	−0.558	−0.921	−0.710	−0.745	−0.700	−0.706	−0.665
AIM	−1.245	−1.215	−1.238	−0.669	−1.205	−0.616	−1.228	−1.215

11. Calculation of Activation Barriers for Methyl Transfer Identity Reactions

11.1 Methyl Transfer Involving Iodide

11.1.1 Methyl iodide

SCF energy:	–336.980594 hartree
Zero-point correction:	+0.036760 hartree
Enthalpy correction:	+0.040854 hartree
Free energy correction:	+0.011036 hartree
Truhlar's Delta G correction:	+0.011036 hartree
Grimme's Delta G correction:	+0.011036 hartree

Cartesian Coordinates

C	0.00000	-1.81320	0.00000
H	-1.03708	-2.13422	0.00000
H	0.51854	-2.13422	0.89813
H	0.51854	-2.13422	-0.89813
I	0.00000	0.32607	0.00000

11.1.2 Iodide

SCF energy:	–297.318803 hartree
Zero-point correction:	+0.000000 hartree
Enthalpy correction:	+0.002360 hartree
Free energy correction:	–0.016848 hartree
Truhlar's Delta G correction:	–0.016848 hartree
Grimme's Delta G correction:	–0.016848 hartree

Cartesian Coordinates

I	0.00000	0.00000	0.00000

11.1.3 Transition State Identity Reaction

SCF energy:	–634.267049 hartree
Zero-point correction:	+0.036306 hartree
Enthalpy correction:	+0.042259 hartree
Free energy correction:	+0.004451 hartree
Truhlar's Delta G correction:	+0.004451 hartree
Grimme's Delta G correction:	+0.004620 hartree
Imaginary Frequency:	509.4 $i$cm^{-1}

Cartesian Coordinates

C	-0.00002	-0.00726	-0.00073
H	0.00014	0.55232	-0.92086
H	-0.00002	-1.08424	-0.02511
H	-0.00018	0.51002	0.94379
I	-2.64394	0.00062	0.00006
I	2.64394	0.00062	0.00006

11.2 Methyl Transfer Involving Pyrimidine *N*-Oxide (3)

11.2.1 Pyrimidine *N*-Oxide (**3**)

SCF energy:	–338.948361 hartree
Zero-point correction:	+0.081611 hartree
Enthalpy correction:	+0.087483 hartree
Free energy correction:	+0.052978 hartree
Truhlar's Delta G correction:	+0.052978 hartree
Grimme's Delta G correction:	+0.052974 hartree

Cartesian Coordinates

C	0.26487	-1.15272	0.00000
N	0.94851	0.02630	-0.00000
C	0.23736	1.17892	-0.00000
N	-1.04443	-1.23702	-0.00000
C	-1.14071	1.13449	0.00000
C	-1.75816	-0.10771	-0.00000
H	0.89179	-2.03487	0.00000
H	0.82933	2.08392	0.00000
H	-1.70979	2.05411	0.00000
O	2.23462	0.03240	0.00000
H	-2.83702	-0.20519	0.00000

11.2.2 *N*-Methyl Pyrimidinium *N*-Oxide Ion (**21**$^+$)

SCF energy:	–378.619925 hartree
Zero-point correction:	+0.123063 hartree
Enthalpy correction:	+0.130750 hartree
Free energy correction:	+0.091756 hartree
Truhlar's Delta G correction:	+0.092170 hartree
Grimme's Delta G correction:	+0.092167 hartree

Cartesian Coordinates

C	-0.01615	-0.94658	-0.00865
N	1.22271	-0.41278	0.00011
C	1.35312	0.94382	0.00578
N	-1.09916	-0.17328	-0.01202
C	0.23842	1.74862	0.00269
C	-1.01290	1.16655	-0.00731
H	-0.10598	-2.02271	-0.01230
H	2.37320	1.30223	0.01158
H	0.34526	2.82390	0.00599
O	2.24047	-1.17740	0.00201
H	-1.94320	1.71722	-0.01098
C	-2.43513	-0.81592	0.01102
H	-2.31843	-1.86849	-0.22917
H	-2.84743	-0.69125	1.01057
H	-3.05610	-0.31825	-0.72955

11.2.3 O-Methyl Pyrimidinium *N*-Oxide Ion (**23**$^+$)

SCF energy:	–378.613655 hartree
Zero-point correction:	+0.123086 hartree
Enthalpy correction:	+0.130726 hartree
Free energy correction:	+0.091645 hartree
Truhlar's Delta G correction:	+0.092374 hartree
Grimme's Delta G correction:	+0.092257 hartree

Cartesian Coordinates

C	0.50632	-1.25048	-0.00002
N	-0.39237	-0.23937	-0.00001
C	-0.01000	1.04487	-0.00002
N	1.79283	-1.03301	0.00000
C	1.34576	1.30953	-0.00000
C	2.22073	0.23658	0.00002
H	0.08592	-2.24882	-0.00001
H	-0.76781	1.81597	-0.00004
H	1.69061	2.33361	0.00000
O	-1.68674	-0.66793	0.00001
H	3.29330	0.38890	0.00003
C	-2.68892	0.36351	0.00001
H	-2.61912	0.96986	-0.90453
H	-3.61660	-0.20344	0.00019
H	-2.61892	0.97003	0.90443

11.2.4 Transition State Identity Reaction N → N

SCF energy:	–717.523828 hartree
Zero-point correction:	+0.204107 hartree
Enthalpy correction:	+0.218130 hartree
Free energy correction:	+0.161539 hartree
Truhlar's Delta G correction:	+0.164702 hartree
Grimme's Delta G correction:	+0.164263 hartree
Imaginary Frequency:	621.4 *i*cm^{-1}

Cartesian Coordinates

C	0.00368	-0.25554	0.01894
N	-1.95733	-0.26162	-0.11354
C	-2.67025	0.70881	0.41227
C	-2.54612	-1.27727	-0.74632
N	-4.02639	0.73300	0.34367
H	-2.20240	1.53757	0.92773
C	-3.92668	-1.31416	-0.85191
H	-1.89987	-2.04368	-1.15736
C	-4.65680	-0.28782	-0.29314
H	-4.43332	-2.12393	-1.35828
N	1.96541	-0.29454	0.14773
C	2.59356	-1.30528	0.74728
C	2.64293	0.70089	-0.38143
C	3.97821	-1.31126	0.81527
H	1.98085	-2.09523	1.16444
N	3.99821	0.75617	-0.34892
H	2.13911	1.52524	-0.86963
C	4.66918	-0.26052	0.25464

H	4.51601	-2.11735	1.29468
H	-0.01922	0.65635	0.59685
H	0.07119	-0.21079	-1.05734
H	-0.04091	-1.21209	0.51782
H	-5.73542	-0.21415	-0.31586
H	5.74603	-0.16196	0.24923
O	4.61778	1.74416	-0.87646
O	-4.68124	1.69857	0.8704

11.2.5 Transition State Identity Reaction O → O

SCF energy:	–717.534306 hartree
Zero-point correction:	+0.204429 hartree
Enthalpy correction:	+0.218231 hartree
Free energy correction:	+0.162267 hartree
Truhlar's Delta G correction:	+0.165731 hartree
Grimme's Delta G correction:	+0.165175 hartree
Imaginary Frequency:	669.6 $i$cm^{-1}

Cartesian Coordinates

C	0.00001	1.11739	-0.00005
O	1.51505	1.13662	-1.14294
O	-1.51504	1.13663	1.14291
N	2.48404	0.41906	-0.60057
C	2.61022	-0.88536	-0.93581
C	3.33311	0.97778	0.28306
N	3.54349	-1.66117	-0.43794
H	1.88437	-1.24885	-1.65249
C	4.32902	0.19885	0.83146
H	3.16906	2.02583	0.49681
C	4.40055	-1.13104	0.44012
H	5.02521	0.62421	1.54066
N	-2.48406	0.41908	0.60061
C	-3.33314	0.97779	-0.28303
C	-2.61027	-0.88532	0.93589
C	-4.32900	0.19883	-0.83149
H	-3.16911	2.02584	-0.49676
N	-3.54349	-1.66116	0.43796
H	-1.88447	-1.24880	1.65263
C	-4.40049	-1.13107	-0.44017
H	-5.02518	0.62418	-1.54070
H	-0.00013	0.03614	-0.00021
H	0.53455	1.65965	0.76593
H	-0.53444	1.66001	-0.76584
H	-5.16228	-1.79106	-0.83793
H	5.16241	-1.79099	0.83781

11.3 Methyl Transfer Involving Pyrazine *N*-Oxide (1)

11.3.1 Pyrazine *N*-Oxide (**1**)

SCF energy:	–338.948037 hartree
Zero-point correction:	+0.081835 hartree
Enthalpy correction:	+0.087670 hartree
Free energy correction:	+0.053220 hartree
Truhlar's Delta G correction:	+0.053220 hartree
Grimme's Delta G correction:	+0.053215 hartree

Cartesian Coordinates

C	0.26352	1.16424	0.00000
N	0.96464	-0.00000	0.00000
C	0.26352	-1.16424	0.00000
C	-1.11658	1.12880	0.00000
C	-1.11658	-1.12879	-0.00000
N	-1.82670	-0.00000	0.00000
H	-1.65916	2.06716	-0.00000
H	0.85431	2.06847	-0.00000
H	0.85431	-2.06847	0.00000
H	-1.65916	-2.06716	-0.00000
O	2.23512	0.00000	-0.00000

11.3.2 *N*-Methyl Pyrazinium *N*-Oxide (**13**$^{+}$)

SCF energy:	–378.628807 hartree
Zero-point correction:	+0.123428 hartree
Enthalpy correction:	+0.131048 hartree
Free energy correction:	+0.092405 hartree
Truhlar's Delta G correction:	+0.092411 hartree
Grimme's Delta G correction:	+0.092584 hartree

Cartesian Coordinates

C	-0.77370	-1.17472	-0.00042
N	-1.47508	0.00000	0.00347
C	-0.77370	1.17472	-0.00042
C	0.59252	-1.15896	-0.00910
C	0.59253	1.15896	-0.00910
N	1.27293	0.00001	-0.01579
H	1.16492	-2.07690	-0.01408
H	-1.36003	-2.08123	0.00032
H	-1.36004	2.08123	0.00033
H	1.16492	2.07690	-0.01410
O	-2.72232	-0.00000	0.00797
C	2.74757	-0.00001	0.01595
H	3.10059	0.89266	-0.49342
H	3.10059	-0.89237	-0.49397
H	3.07136	-0.00030	1.05594

11.3.3 O-Methyl Pyrazinium *N*-Oxide (**15**$^{+}$)

SCF energy:	–378.608535 hartree
Zero-point correction:	+0.122591 hartree
Enthalpy correction:	+0.130352 hartree
Free energy correction:	+0.091208 hartree
Truhlar's Delta G correction:	+0.091495 hartree
Grimme's Delta G correction:	+0.091554 hartree

Cartesian Coordinates

C	0.20982	-1.17502	-0.16268
N	-0.41529	-0.00005	-0.28265
C	0.20974	1.17497	-0.16274
C	1.57163	-1.14105	0.10083
C	1.57154	1.14112	0.10078
N	2.24151	0.00006	0.23065
H	2.11521	-2.07185	0.20156
H	-0.38356	-2.07150	-0.28497
H	-0.38365	2.07144	-0.28511
H	2.11506	2.07196	0.20145
O	-1.74032	-0.00013	-0.60425
C	-2.56366	0.00008	0.59187
H	-3.58194	-0.00005	0.21160
H	-2.36828	0.90208	1.17241
H	-2.36820	-0.90170	1.17276

11.3.4 Transition State Identity Reaction N → N

SCF energy:	–717.529061 hartree
Zero-point correction:	+0.204595 hartree
Enthalpy correction:	+0.218532 hartree
Free energy correction:	+0.162293 hartree
Truhlar's Delta G correction:	+0.165360 hartree
Grimme's Delta G correction:	+0.164983 hartree
Imaginary Frequency:	639.2 $i$cm^{-1}

Cartesian Coordinates

C	0.00011	-0.03639	0.00449
N	1.95349	-0.04288	0.02048
C	2.66242	-1.12875	-0.28129
C	2.61320	1.07790	0.31038
C	4.03868	-1.12143	-0.29861
H	2.12139	-2.03818	-0.51788
C	3.98708	1.13968	0.30656
H	4.65047	-1.97873	-0.53676
N	-1.95319	-0.02845	-0.02524
C	-2.65434	-1.11935	0.27794
C	-2.62110	1.08765	-0.31327
C	-4.03020	-1.12198	0.29846
H	-2.10530	-2.02458	0.51231
C	-3.99573	1.13956	-0.30644

N	-4.71089	0.02127	0.00376
H	-4.63564	-1.98357	0.53744
N	4.71073	0.02621	-0.00250
O	5.97214	0.05826	-0.01388
O	-5.97246	0.04420	0.01740
H	-4.57519	2.02164	-0.53508
H	-2.04500	1.97273	-0.55904
H	-0.00415	-1.10596	-0.14965
H	-0.00963	0.36396	1.00679
H	4.55998	2.02585	0.53605
H	2.02962	1.95869	0.55432
H	0.01426	0.63397	-0.84151

11.3.5 Transition State Identity Reaction O → O

SCF energy:	–717.523094 hartree
Zero-point correction:	+0.204513 hartree
Enthalpy correction:	+0.218210 hartree
Free energy correction:	+0.163041 hartree
Truhlar's Delta G correction:	+0.165687 hartree
Grimme's Delta G correction:	+0.165467 hartree
Imaginary Frequency:	687.0 $i$cm^{-1}

Cartesian Coordinates

C	-0.00003	-0.84277	0.00000
O	-1.47072	-0.86673	-1.20899
O	1.47061	-0.86638	1.20908
N	-2.51771	-0.31668	-0.63878
C	-3.36095	-1.07469	0.08617
C	-2.73834	1.00375	-0.77540
C	-4.45426	-0.46528	0.67827
H	-3.12677	-2.12779	0.15422
C	-3.84944	1.55524	-0.16018
H	-2.02415	1.55335	-1.37202
N	-4.70554	0.83662	0.56246
H	-5.14050	-1.06730	1.26128
H	-4.03781	2.61661	-0.26710
N	2.51766	-0.31651	0.63881
C	2.73841	1.00391	0.77524
C	3.36085	-1.07471	-0.08600
C	3.84958	1.55522	0.15998
H	2.02424	1.55367	1.37175
C	4.45425	-0.46549	-0.67814
H	3.12658	-2.12780	-0.15391
N	4.70565	0.83640	-0.56251
H	4.03805	2.61658	0.26675
H	5.14045	-1.06766	-1.26104
H	-0.56412	-1.38609	0.74425
H	0.56382	-1.38696	-0.74379
H	0.00021	0.23837	-0.00047

12 Calculations on Reactions with Methyl Iodide and Methyl Triflate

12.1 Methylation of Pyridine (28)

12.1.1 Methyl Triflate

SCF energy:	–1000.213262 hartree
Zero-point correction:	+0.068039 hartree
Enthalpy correction:	+0.078169 hartree
Free energy correction:	+0.032986 hartree
Truhlar's Delta G correction:	+0.033685 hartree
Grimme's Delta G correction:	+0.033748 hartree

Cartesian Coordinates

O	0.14961	1.83242	-0.76112
S	0.41956	0.67876	0.04845
O	0.80716	0.78320	1.43023
O	1.40565	-0.24861	-0.75382
C	-1.11715	-0.36119	-0.00014
F	-2.07844	0.27303	0.64814
F	-0.88290	-1.52575	0.58292
F	-1.48201	-0.56039	-1.25300
C	2.27344	-1.18381	-0.03564
H	1.67126	-1.89244	0.52961
H	2.82132	-1.68966	-0.82497
H	2.94757	-0.62619	0.61004

12.1.2 Triflate Ion

SCF energy:	–960.567597 hartree
Zero-point correction:	+0.027777 hartree
Enthalpy correction:	+0.035760 hartree
Free energy correction:	–0.004526 hartree
Truhlar's Delta G correction:	–0.004057 hartree
Grimme's Delta G correction:	–0.004078 hartree

Cartesian Coordinates

O	-1.23298	-1.37549	0.38099
S	-0.90907	-0.00016	0.00006
O	-1.23319	1.01766	1.00037
O	-1.23278	0.35740	-1.38158
C	0.94417	-0.00001	0.00010
F	1.42517	-0.31444	1.20518
F	1.42405	1.20128	-0.33017
F	1.42541	-0.88617	-0.87498

12.1.3 Transition State for Methyl Iodide

SCF energy:	–584.799693 hartree
Zero-point correction:	+0.127449 hartree

Enthalpy correction:	+0.136820 hartree
Free energy correction:	+0.090730 hartree
Truhlar's Delta G correction:	+0.092049 hartree
Grimme's Delta G correction:	+0.092055 hartree
Imaginary Frequency:	547.9 $i$cm^{-1}

Cartesian Coordinates

C	0.13742	0.01418	-0.01218
H	0.08277	-0.38754	0.98579
H	0.10008	1.08084	-0.16385
H	0.09180	-0.64956	-0.85970
I	2.65999	-0.00134	0.00346
C	-2.68248	1.16435	-0.01176
C	-2.64062	-1.14048	-0.01245
C	-4.07140	1.18497	0.00838
H	-2.10187	2.08151	-0.01960
C	-4.02730	-1.21263	0.00760
H	-2.02535	-2.03503	-0.02039
C	-4.75335	-0.02655	0.01809
H	-4.59847	2.13048	0.01633
H	-4.51914	-2.17695	0.01491
N	-1.99232	0.02433	-0.02238
H	-5.83684	-0.04637	0.03393

12.1.4 Transition State for Methyl Triflate

SCF energy:	–1248.041686 hartree
Zero-point correction:	+0.157223 hartree
Enthalpy correction:	+0.172913 hartree
Free energy correction:	+0.111304 hartree
Truhlar's Delta G correction:	+0.116184 hartree
Grimme's Delta G correction:	+0.115162 hartree
Imaginary Frequency:	609.9 $i$cm^{-1}

Cartesian Coordinates

C	0.59052	-0.78365	-0.36165
H	0.51364	-0.57423	0.69468
H	0.93741	-1.75400	-0.68029
H	0.55833	0.02389	-1.07479
C	3.62842	-1.12267	-0.40148
C	2.93930	0.96797	0.27716
C	4.96680	-0.77889	-0.25811
H	3.33192	-2.11203	-0.73594
C	4.24872	1.39693	0.44785
H	2.09699	1.62401	0.47701
C	5.27992	0.50463	0.17450
H	5.73871	-1.50409	-0.48179
H	4.44833	2.40552	0.78666
N	2.64291	-0.26449	-0.13796
H	6.31374	0.80598	0.29692
O	-1.13569	-1.25271	-0.61487
S	-2.22823	-0.80612	0.32915
C	-2.53909	0.92263	-0.25376

F	-3.48071	1.49202	0.48845
F	-1.42293	1.63989	-0.15361
F	-2.93294	0.92494	-1.52092
O	-1.76545	-0.64845	1.69541
O	-3.46712	-1.51371	0.08754

12.2 Methylation of Pyrimidine (27)

12.2.1 Transition State for Methyl Iodide

SCF energy:	–600.829663 hartree
Zero-point correction:	+0.115790 hartree
Enthalpy correction:	+0.125116 hartree
Free energy correction:	+0.078790 hartree
Truhlar's Delta G correction:	+0.080442 hartree
Grimme's Delta G correction:	+0.080332 hartree
Imaginary Frequency:	558.2 *i*cm^{-1}

Cartesian Coordinates

C	0.09976	-0.02447	0.00012
H	0.06600	0.51333	0.93315
H	0.06581	0.51364	-0.93272
H	0.08339	-1.10271	-0.00007
I	2.65135	0.00453	-0.00004
C	-2.65071	1.13772	0.00018
C	-2.71138	-1.14956	0.00014
C	-4.03480	1.17525	-0.00011
H	-2.04400	2.03794	0.00030
H	-2.15390	-2.08039	0.00022
C	-4.69108	-0.04865	-0.00023
H	-4.57612	2.11133	-0.00026
N	-2.00001	-0.02603	0.00030
H	-5.77483	-0.09958	-0.00043
N	-4.03694	-1.21327	-0.00011

12.2.2 Transition State for Methyl Triflate

SCF energy:	–1264.071957 hartree
Zero-point correction:	+0.145638 hartree
Enthalpy correction:	+0.161203 hartree
Free energy correction:	+0.099993 hartree
Truhlar's Delta G correction:	+0.104642 hartree
Grimme's Delta G correction:	+0.103700 hartree
Imaginary Frequency:	616.2 *i*cm^{-1}

Cartesian Coordinates

C	0.62496	-0.79366	-0.32206
H	0.52486	-0.53283	0.72097
H	0.95324	-1.78601	-0.58826
H	0.58922	-0.02748	-1.07930
C	3.65098	-1.05993	-0.48735

C	2.95555	0.92367	0.41197
N	4.94119	-0.76058	-0.40261
H	3.37769	-2.02434	-0.90305
C	4.27311	1.32816	0.54271
H	2.12261	1.55008	0.71715
C	5.24457	0.43380	0.11268
H	4.53098	2.29262	0.95826
N	2.65286	-0.26954	-0.10208
H	6.29888	0.68027	0.18237
O	-1.12680	-1.26806	-0.56905
S	-2.21693	-0.78914	0.35780
C	-2.54166	0.91038	-0.29835
F	-3.47808	1.50995	0.42692
F	-1.42727	1.63570	-0.24172
F	-2.94815	0.85545	-1.56053
O	-1.74946	-0.56714	1.71426
O	-3.45480	-1.51226	0.15734

12.3 Methylation of Pyrazine (7)

12.3.1 Transition State for Methyl Iodide

SCF energy:	–600.820701 hartree
Zero-point correction:	+0.115661 hartree
Enthalpy correction:	+0.124908 hartree
Free energy correction:	+0.078918 hartree
Truhlar's Delta G correction:	+0.080343 hartree
Grimme's Delta G correction:	+0.080317 hartree
Imaginary Frequency:	541.4 *i*cm^{-1}

Cartesian Coordinates

C	-0.08206	0.00012	0.02625
H	-0.07076	-0.85202	0.68612
H	-0.04869	-0.14657	-1.04064
H	-0.07307	0.99806	0.43311
I	-2.65195	0.00003	-0.00728
C	2.65978	-1.14071	0.02674
C	2.67047	1.14733	0.02659
C	4.05031	-1.13956	-0.01914
H	2.08575	-2.06060	0.04421
C	4.06124	1.13264	-0.01973
H	2.10600	2.07302	0.04453
N	4.74994	-0.00664	-0.04263
H	4.60005	-2.07349	-0.03718
H	4.61974	2.06136	-0.03862
N	1.98945	0.00662	0.04976

12.3.2 Transition State for Methyl Triflate

SCF energy:	–1264.063294 hartree
Zero-point correction:	+0.145460 hartree
Enthalpy correction:	+0.160955 hartree

Free energy correction:	+0.100467 hartree
Truhlar's Delta G correction:	+0.104420 hartree
Grimme's Delta G correction:	+0.103812 hartree
Imaginary Frequency:	614.4 *i*cm^{-1}

Cartesian Coordinates

C	0.61921	-0.76025	-0.38232
H	0.95470	-1.72286	-0.73525
H	0.56766	0.06813	-1.07047
H	0.52987	-0.58925	0.68022
N	2.64423	-0.24920	-0.15028
C	3.63093	-1.10860	-0.38282
C	2.95439	0.98036	0.25055
C	4.95709	-0.72367	-0.20805
H	3.36448	-2.10784	-0.70941
C	4.28366	1.35266	0.42094
H	2.13734	1.66941	0.43673
N	5.28392	0.50305	0.19283
H	5.76187	-1.42461	-0.39743
H	4.53646	2.35476	0.74769
O	-1.12747	-1.23221	-0.64981
S	-2.21756	-0.81761	0.30817
O	-3.45560	-1.52410	0.05663
O	-1.75048	-0.69121	1.67689
C	-2.54033	0.92389	-0.22795
F	-3.47594	1.47269	0.53731
F	-2.94687	0.95748	-1.49084
F	-1.42496	1.64216	-0.12119

12.4 Methylation of Pyridine *N*-Oxide (8)

12.4.1 Transition State for Methyl Iodide

SCF energy:	–659.875011 hartree
Zero-point correction:	+0.131927 hartree
Enthalpy correction:	+0.141913 hartree
Free energy correction:	+0.094439 hartree
Truhlar's Delta G correction:	+0.095943 hartree
Grimme's Delta G correction:	+0.095738 hartree
Imaginary Frequency:	582.5 *i*cm^{-1}

Cartesian Coordinates

C	-0.40236	0.85146	0.00027
H	-0.16830	0.36790	0.93520
H	-0.16825	0.36817	-0.93478
H	-0.84408	1.83395	0.00041
I	-2.73001	-0.24897	-0.00007
O	1.38142	1.73258	0.00038
C	2.76647	0.31523	-1.17623
C	2.76634	0.31459	1.17637
C	3.72902	-0.67432	-1.19761
H	2.32958	0.76767	-2.05561

C	3.72889	-0.67497	1.19732
H	2.32934	0.76653	2.05596
H	4.08128	-1.03616	-2.15436
H	4.08104	-1.03734	2.15391
N	2.30844	0.79269	0.00018
C	4.22201	-1.18012	-0.00026
H	4.97738	-1.95578	-0.00042

12.4.2 Transition State for Methyl Triflate

SCF energy:	–1323.118407 hartree
Zero-point correction:	+0.161735 hartree
Enthalpy correction:	+0.178020 hartree
Free energy correction:	+0.115592 hartree
Truhlar's Delta G correction:	+0.120598 hartree
Grimme's Delta G correction:	+0.119490 hartree
Imaginary Frequency:	662.1 $i$cm^{-1}

Cartesian Coordinates

C	-0.12073	1.75711	0.53653
H	-0.20297	1.49019	-0.50658
H	-0.32510	1.03808	1.31362
H	0.00019	2.79504	0.80178
O	1.68877	1.45706	0.63364
S	2.36265	0.52759	-0.34536
O	1.80479	0.60930	-1.68340
O	3.80189	0.53118	-0.18948
C	1.81545	-1.13163	0.26899
F	2.33970	-2.09203	-0.48271
F	0.48791	-1.22192	0.21151
F	2.19794	-1.31569	1.52652
O	-2.11379	2.07976	0.47804
N	-2.65313	0.91379	0.17329
C	-3.01567	0.07175	1.16395
C	-2.80624	0.57298	-1.12377
C	-3.56114	-1.16130	0.86552
H	-2.84807	0.44348	2.16546
C	-3.34631	-0.65161	-1.46389
H	-2.48256	1.32005	-1.83534
H	-3.84499	-1.81442	1.67995
H	-3.45808	-0.89702	-2.51165
C	-3.73124	-1.53506	-0.46237
H	-4.15676	-2.49859	-0.71245

12.5 Methylation of Pyrimidine *N*-Oxide (3)

12.5.1 Transition State for N-Alkylation by Methyl Iodide

SCF energy:	–675.895370 hartree
Zero-point correction:	+0.119727 hartree
Enthalpy correction:	+0.129865 hartree
Free energy correction:	+0.081050 hartree

Truhlar's Delta G correction:	+0.083450 hartree
Grimme's Delta G correction:	+0.083041 hartree
Imaginary Frequency:	565.7 $i$cm^{-1}

Cartesian Coordinates

C	-0.40825	0.10800	-0.02109
H	-0.40752	0.39037	1.01899
H	-0.32897	-0.93107	-0.29811
H	-0.45853	0.86632	-0.78588
I	-2.98134	-0.07450	0.00577
C	2.40596	-0.79600	-0.02549
C	2.19303	1.49356	-0.01549
N	3.76323	-0.73092	0.00429
H	1.97784	-1.78985	-0.03990
C	3.57155	1.63338	0.01505
H	1.51523	2.33888	-0.02387
C	4.34738	0.49444	0.02449
O	4.46222	-1.80463	0.01305
H	4.04147	2.60702	0.03113
N	1.64921	0.27670	-0.03649
H	5.42839	0.48323	0.04787

12.5.2 Transition State for O-Alkylation by Methyl Iodide

SCF energy:	–675.900119 hartree
Zero-point correction:	+0.119877 hartree
Enthalpy correction:	+0.129857 hartree
Free energy correction:	+0.081965 hartree
Truhlar's Delta G correction:	+0.083777 hartree
Grimme's Delta G correction:	+0.083499 hartree
Imaginary Frequency:	610.2 $i$cm^{-1}

Cartesian Coordinates

C	0.40228	-0.79009	-0.21577
H	0.82738	-1.71587	-0.56671
H	0.21074	-0.65241	0.83628
H	0.14842	-0.01449	-0.92109
I	2.75010	0.22783	0.06714
O	-1.39251	-1.62034	-0.45066
C	-2.75801	0.08714	-1.20203
C	-2.85350	-0.61665	1.02717
C	-3.74083	1.01247	-0.92382
H	-2.29081	-0.05872	-2.16708
N	-3.79011	0.24908	1.33322
H	-2.44728	-1.30387	1.75852
C	-4.23782	1.06189	0.37117
H	-4.10359	1.67118	-1.70046
N	-2.32712	-0.72535	-0.21624
H	-5.01213	1.76712	0.64826

12.5.3 Transition State for N-Alkylation by Methyl Triflate

SCF energy:	–1339.138179 hartree
Zero-point correction:	+0.149359 hartree

Enthalpy correction:	+0.165756 hartree
Free energy correction:	+0.102444 hartree
Truhlar's Delta G correction:	+0.107541 hartree
Grimme's Delta G correction:	+0.106444 hartree
Imaginary Frequency:	622.1 $i$cm^{-1}

Cartesian Coordinates

C	0.33838	-0.66903	-0.32745
H	0.20728	-0.46948	0.72565
H	0.71008	-1.63130	-0.64280
H	0.23950	0.12443	-1.05066
N	2.29943	-0.02935	-0.08249
C	2.49918	1.24284	0.26531
C	3.31812	-0.83822	-0.26393
C	3.78824	1.72127	0.43622
H	1.61951	1.86116	0.40203
N	4.60763	-0.43539	-0.11383
H	3.17463	-1.87332	-0.54573
C	4.84120	0.85450	0.23873
H	3.97885	2.74784	0.71718
O	5.56922	-1.26093	-0.30344
O	-1.40587	-1.24438	-0.60205
S	-2.52061	-0.85126	0.33208
O	-3.71958	-1.63282	0.11235
O	-2.06836	-0.63926	1.69596
C	-2.93609	0.84563	-0.27822
F	-3.89925	1.37799	0.46460
F	-3.34427	0.80391	-1.54059
F	-1.86005	1.62640	-0.20554
H	5.88510	1.11670	0.34255

12.5.4 Transition State for O-Alkylation by Methyl Triflate

SCF energy:	–1339.142371 hartree
Zero-point correction:	+0.149567 hartree
Enthalpy correction:	+0.165930 hartree
Free energy correction:	+0.102106 hartree
Truhlar's Delta G correction:	+0.108124 hartree
Grimme's Delta G correction:	+0.106580 hartree
Imaginary Frequency:	664.2 $i$cm^{-1}

Cartesian Coordinates

C	-0.32305	1.33268	0.90702
H	-0.27637	0.28348	1.15519
H	-0.18415	2.06606	1.68491
H	-0.64349	1.64980	-0.07254
O	1.47546	1.45085	0.47348
S	2.03963	0.60603	-0.64029
O	1.01250	0.06432	-1.51268
O	3.21778	1.19752	-1.24002
C	2.67411	-0.85871	0.29682
F	3.17649	-1.75688	-0.54281

F	1.68364	-1.42429	0.98235
F	3.62401	-0.49345	1.14906
O	-2.25055	1.29688	1.44770
N	-2.89420	0.46662	0.65536
C	-2.95011	-0.84906	0.94215
C	-3.50009	0.94036	-0.45921
C	-3.63428	-1.69072	0.09135
H	-2.44333	-1.15365	1.84845
N	-4.16218	0.17483	-1.29396
H	-3.40225	2.00735	-0.61530
C	-4.23478	-1.13301	-1.02858
H	-3.69320	-2.74900	0.30419
H	-4.78618	-1.74485	-1.73256

12.6 Methylation of Pyrazine *N*-Oxide (1)

12.6.1 Transition State for N-Alkylation by Methyl Iodide

SCF energy:	–675.897471 hartree
Zero-point correction:	+0.119953 hartree
Enthalpy correction:	+0.129995 hartree
Free energy correction:	+0.082008 hartree
Truhlar's Delta G correction:	+0.083784 hartree
Grimme's Delta G correction:	+0.083620 hartree
Imaginary Frequency:	549.1 *i*cm^{-1}

Cartesian Coordinates

C	-0.50833	-0.00072	0.03533
H	-0.50406	-0.85522	0.69210
H	-0.47253	-0.14285	-1.03195
H	-0.50476	0.99535	0.44667
I	-3.08243	-0.00037	-0.01323
C	2.23958	-1.13643	0.04718
C	2.24724	1.14517	0.04675
C	3.61517	-1.17019	-0.00135
H	1.67630	-2.06259	0.06556
C	3.62328	1.16932	-0.00205
H	1.69097	2.07550	0.06518
N	4.31689	-0.00271	-0.02645
H	4.20437	-2.07497	-0.02153
H	4.21845	2.07017	-0.02301
N	1.55719	0.00682	0.07269
O	5.57998	-0.00718	-0.07132

12.6.2 Transition State for O-Alkylation by Methyl Iodide

SCF energy:	–675.894942 hartree
Zero-point correction:	+0.120147 hartree
Enthalpy correction:	+0.129989 hartree
Free energy correction:	+0.082700 hartree
Truhlar's Delta G correction:	+0.084231 hartree
Grimme's Delta G correction:	+0.084004 hartree
Imaginary Frequency:	594.7 *i*cm^{-1}

Cartesian Coordinates

C	-0.35380	0.85037	0.00167
H	-0.80973	1.82663	0.00415
H	-0.15547	0.34868	0.93598
H	-0.15619	0.35307	-0.93512
I	-2.74387	-0.23868	-0.00047
O	1.39219	1.69632	0.00276
C	2.78722	0.29184	-1.16689
C	2.78646	0.28734	1.16789
C	3.75896	-0.69342	-1.13546
H	2.36934	0.72085	-2.06650
C	3.75821	-0.69782	1.13329
H	2.36805	0.71293	2.06887
N	4.24835	-1.19156	-0.00188
H	4.14400	-1.08163	-2.07051
H	4.14260	-1.08966	2.06709
N	2.31773	0.77140	0.00128

12.6.3 Transition State for N-Alkylation by Methyl Triflate

SCF energy:	–1339.140028 hartree
Zero-point correction:	+0.149547 hartree
Enthalpy correction:	+0.165943 hartree
Free energy correction:	+0.102870 hartree
Truhlar's Delta G correction:	+0.107578 hartree
Grimme's Delta G correction:	+0.106785 hartree
Imaginary Frequency:	620.5 *i*cm^{-1}

Cartesian Coordinates

C	0.20351	-0.81501	-0.38943
H	0.50154	-1.80197	-0.70652
H	0.16978	-0.01446	-1.11055
H	0.12513	-0.59961	0.66591
N	2.24238	-0.36825	-0.19261
C	3.20559	-1.25890	-0.42067
C	2.60972	0.85591	0.18396
C	4.54176	-0.95391	-0.27999
H	2.91214	-2.25650	-0.72741
C	3.92936	1.21582	0.34059
H	1.82668	1.58275	0.37025
N	4.90913	0.29889	0.10627
H	5.34886	-1.64935	-0.45594
H	4.26459	2.19639	0.64411
O	-1.56481	-1.24069	-0.62872
S	-2.63073	-0.78173	0.33512
O	-3.88826	-1.46515	0.11813
O	-2.14053	-0.63612	1.69393
C	-2.92393	0.95451	-0.23387
F	-3.83921	1.53833	0.53017
F	-3.34452	0.97021	-1.49261
F	-1.79324	1.65221	-0.15511
O	6.12848	0.60429	0.24378

12.6.4 Transition State for O-Alkylation by Methyl Triflate

SCF energy:	–1339.138211 hartree
Zero-point correction:	+0.149981 hartree
Enthalpy correction:	+0.166123 hartree
Free energy correction:	+0.103765 hartree
Truhlar's Delta G correction:	+0.108854 hartree
Grimme's Delta G correction:	+0.107708 hartree
Imaginary Frequency:	671.4 *i*cm^{-1}

Cartesian Coordinates

C	-0.18773	-1.71515	-0.53991
H	-0.22393	-1.46338	0.50982
H	-0.36654	-0.97053	-1.29960
H	-0.06562	-2.74630	-0.82978
O	1.66585	-1.43996	-0.66131
S	2.37448	-0.56119	0.33403
O	1.80100	-0.63282	1.66724
O	3.81481	-0.62645	0.19573
C	1.91235	1.13006	-0.25997
F	2.45343	2.05583	0.52314
F	0.58784	1.27242	-0.23473
F	2.33401	1.32326	-1.50375
O	-2.14571	-2.02655	-0.46694
N	-2.70306	-0.88259	-0.16687
C	-3.11544	-0.05651	-1.14803
C	-2.84328	-0.51818	1.12268
C	-3.68350	1.15682	-0.79955
H	-2.97456	-0.40513	-2.16137
C	-3.41655	0.70924	1.40769
H	-2.49474	-1.22395	1.86347
N	-3.83642	1.54851	0.46362
H	-4.01907	1.82419	-1.58406
H	-3.53110	1.00753	2.44279

13 Determination of 2nd Order Rate Constant

Pyrazine *N*-oxide (**1**) (0.010 g, 0.10 mmol) was dissolved in dry CD_3CN (0.20 ml) in a glove box under nitrogen atmosphere. This solution was taken up in a syringe and the syringe was placed in a long Schlenk flask inside the glove box, and the Schlenk flask was sealed. Methyl iodide (0.147 g, 1.04 mmol, 10 equivalents) was dissolved in 0.65 ml dry CD_3CN in the glove box. This solution was placed in an NMR tube, which was sealed using a rubber septum. The seal was then wrapped with PTFE tape and Parafilm. Finally, the NMR tube was placed in a long Schlenk flask, which was then sealed inside the glove box. Both Schlenk flasks were removed from the glove box and brought to the NMR spectrometer (500 MHz instrument).

The Schlenk tubes were placed in the NMR spectrometer room for 20 minutes to allow them to equilibrate to the controlled room temperature of 25 °C. The NMR tube containing the MeI solution was removed from the Schlenk flask and placed in the NMR spectrometer. The probe of the spectrometer was also kept at 25 °C. After obtaining the first ^{1}H NMR spectrum and the correct shim for this sample, the NMR tube was ejected. The pyrazine *N*-oxide solution (0.18 ml, containing 0.090 mmol pyrazine *N*-oxide) in its syringe was removed from its Schlenk flask and added to the NMR tube by injection through the rubber septum. The septum was re-wrapped with parafilm after removal of the syringe. The NMR tube was inverted and then rapidly returned to the spectrometer to obtain NMR spectra of the ongoing reaction at certain intervals.

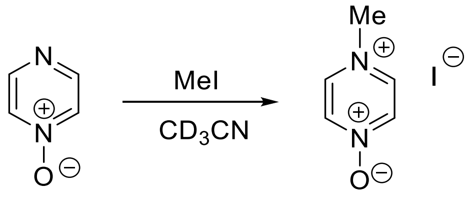

1 **13a**

Each spectrum was obtained using 4 scans, a 5 second relaxation delay and a 30° pulse. The time ascribed to each spectrum was when the spectrum measurement ended. In the obtained spectra, the C*H*D_2CN signal at δ 1.968 was set at a constant integral value throughout and the other signals are given relative to this value.

The following signals were observed in the spectra after addition.

^{1}H NMR (500 MHz, CD_3CN, 298K)

Assigned to **1**: δ 8.49 – 8.38 (m, 2H), 8.14 – 8.06 (m, 2H).

Assigned to MeI: δ 2.20 (s, 3H).

Assigned to **13a**: δ δ 8.65 – 8.59 (m, 2H), 8.57 – 8.52 (m, 2H), 4.19 (s, 3H, NC*H*$_3$).

<u>Note</u>: ^{13}C satellite peaks of the 2H signal of **1** at δ 8.10 appear at δ 8.30 – 8.28 and 7.92 – 7.90. These signals were included in the integration value for that signal. The aromatic signals of **13a** showed a variable chemical shift, moving downfield as the reaction progressed. The signal also initially appeared as a singlet, before splitting into two doublets as it moved downfield.

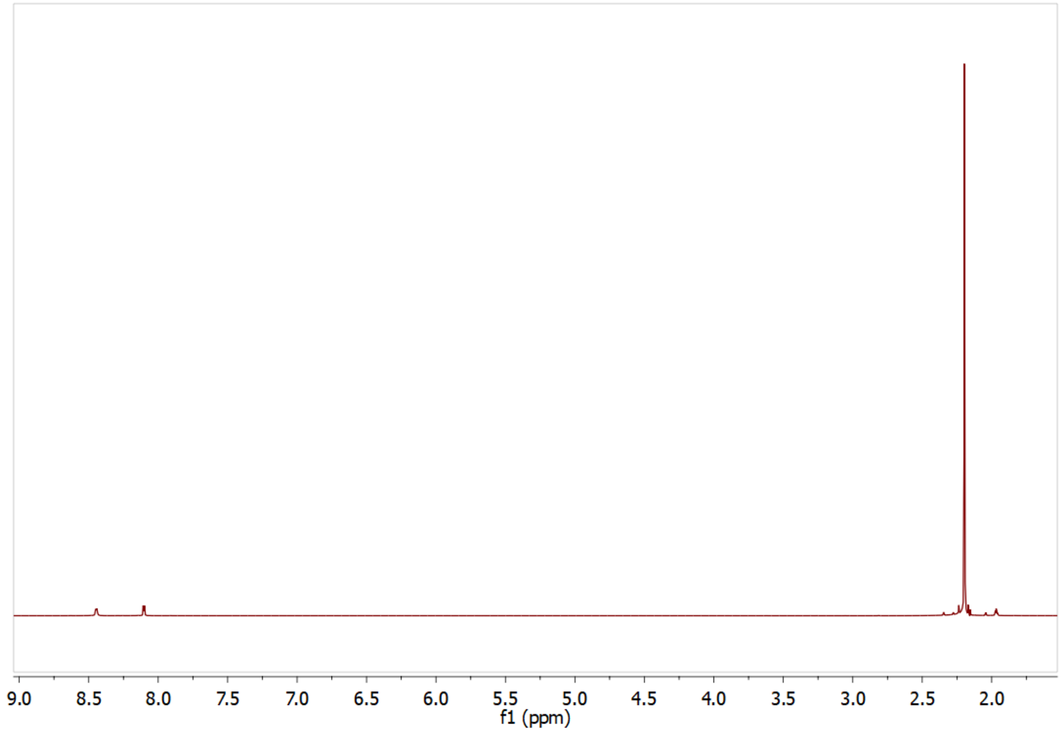

Figure S86: ^{1}H NMR spectrum in CD_3CN of the reaction of **1** and MeI. The MeI signal is disproportionately large compared to the signals of **1** and **13a** as there are 10 equivalents of MeI relative to **1**.

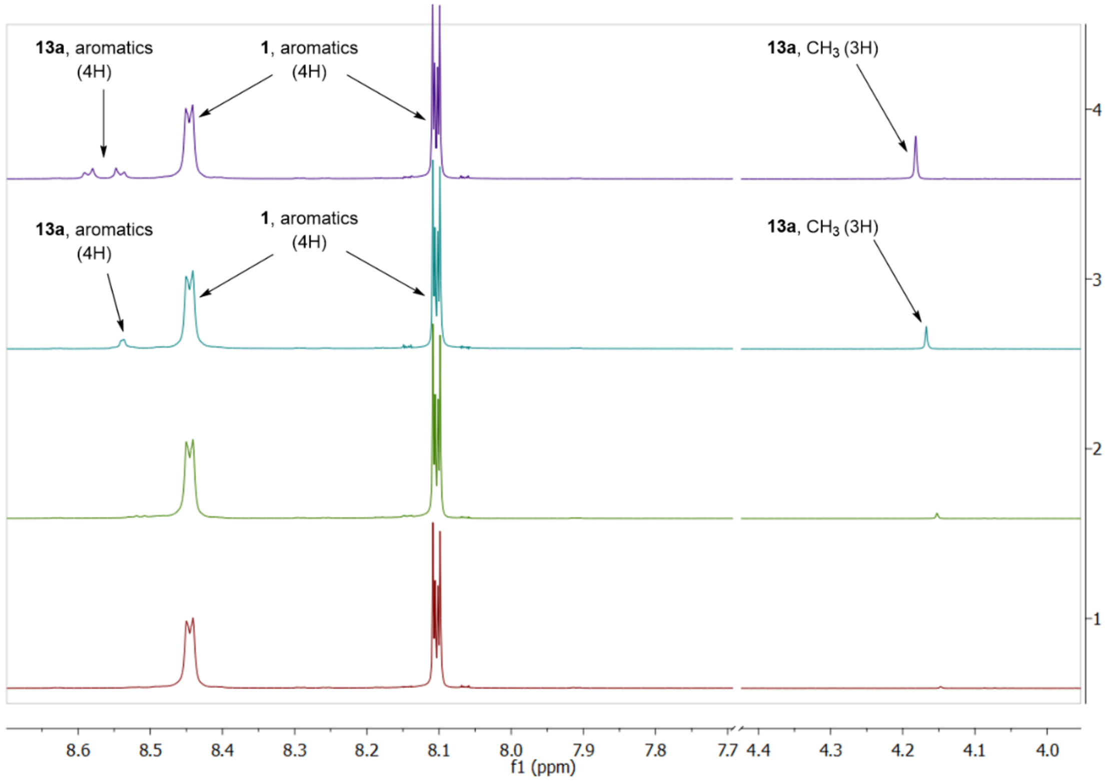

Figure S87: Stacked ^{1}H NMR spectra in CD_3CN of the reaction of **1** and MeI. The signals of **13a** appear as the reaction progresses. The aromatic signals associated with **13a** showed a variable chemical shift, in addition to being observed as both a singlet in earlier spectra, and a multiplet in later spectra.

The consumption of pyrazine *N*-oxide (**1**) was monitored by observing the decrease in the integration value at 8.10 ppm relative to the signal of C*H*D_2CN. The integration value was assigned a concentration value ($[\mathbf{1}]_t$) relative to the concentration of (**1**) at t = 0.

At t = 0,

$$[\mathbf{1}]_{t=0} = \frac{\left(\frac{0.090 \text{ g}}{96.089 \text{ g mol}^{-1}}\right)}{0.83 \text{ ml}} = 0.113 \text{ mmol/ml}$$

At t = 713 seconds, the integration value of the signal at 8.10 ppm was 99.7% of its value at t = 0, giving:

$$[\mathbf{1}]_{t=713\text{ s}} = (0.113 \text{ mmol/ml}) \times \ 0.997 \ = 0.1127 \text{ mmol/ml}$$

This procedure was continued at various time points in order to monitor the consumption of pyrazine N-oxide. After 25 hours, conversion was approximately 28%. An approximate value of the 2nd order rate constant was derived based on data recorded for the reaction up to this level of conversion.

For each ^{1}H NMR spectrum (time t), the integrations of **1** and **13a** at time t ($I_{\mathbf{1}}$ and $I_{\mathbf{13a}}$, respectively) relative to the integration of the residual C*H*D_2CN were established (the integration of C*H*D_2CN in each spectrum was set equal to an arbitrary value of 15.2). The integration of **13a** ($I_{\mathbf{13a}}$) was scaled (multiplied by 2/3) to take account of the additional protons contributing to the signal used for the integration.

The total amount of **1** and **13a** present always equals the initial amount of **1** added, i.e.

$$n_{\mathbf{1}} + n_{\mathbf{13a}} = n_{\mathbf{1},t=0}$$

where:

$$n_{\mathbf{1}} = \text{amount of } \mathbf{1} \text{ (mmol) at time } t$$

$$n_{\mathbf{13a}} = \text{amount of } \mathbf{13a} \text{ (mmol) at time } t$$

$$n_{\mathbf{1},t=0} = \text{inital amount of } \mathbf{1} \text{ added (mmol)}$$

Hence, the quantity ($I_{\mathbf{1}}$ + $I_{\mathbf{13a}}$) – the sum of the integrations of the signals of **1** and **13a** (scaled appropriately) – was used to represent the initial amount of **1** added. The consumption of **1** at time t was then established as follows:

$$\text{Consumption of } \mathbf{1} \text{ at time } t = \frac{I_{\mathbf{1}}}{(I_{\mathbf{1}} + I_{\mathbf{13a}})} = \frac{[\mathbf{1}]_t}{[\mathbf{1}]_0}$$

See column 4 of Table S9 below for the quantities calculated in this manner.

Table S9. Recorded integration values (*I*) and calculated concentrations of **1** and **13a** at various time points, with derived values of ln ([**1**]$_t$ / [**1**]$_0$). Note that the integration value of **13a** shown here was scaled to take into account the additional protons contributing to the signal used for the integration.

Time (seconds)	I_1	I_{13a}	[**1**]$_t$ / [**1**]$_0$	ln ([**1**]$_t$ / [**1**]$_0$)
0	25.00	0	1	0
713	24.89	0.07	0.997	-2.7×10^{-3}
1080	24.87	0.09	0.996	-3.8×10^{-3}
1248	24.81	0.13	0.995	-5.1×10^{-3}
1620	24.77	0.15	0.994	-5.9×10^{-3}
1740	24.76	0.17	0.993	-6.7×10^{-3}
2160	24.74	0.19	0.992	-7.8×10^{-3}
2460	24.68	0.21	0.991	-8.6×10^{-3}
3060	24.63	0.25	0.990	-1.0×10^{-2}
3720	24.53	0.31	0.987	-1.3×10^{-2}
5460	24.32	0.45	0.982	-1.9×10^{-2}
7500	24.03	0.64	0.974	-2.6×10^{-2}
8100	23.97	0.69	0.972	-2.9×10^{-2}
11760	23.51	0.99	0.959	-4.1×10^{-2}
22560	22.48	1.68	0.930	-7.2×10^{-2}
29760	21.58	2.29	0.904	-1.0×10^{-1}
36960	20.83	2.79	0.882	-1.3×10^{-1}
54960	19.00	4.00	0.826	-1.9×10^{-1}
72960	16.96	5.36	0.760	-2.8×10^{-1}
90960	15.71	6.17	0.718	-3.3×10^{-1}

For the 2nd order reaction of **1** with MeI (and rate constant *k*):

$$\text{Rate} = -k[\mathbf{1}][\text{MeI}]$$

By including 10 equivalents of MeI, it can be assumed that:

$$[\text{MeI}]_t = [\text{MeI}]_0$$

Thus, for this pseudo-1st order reaction:

$$\text{Rate} = -k'[\mathbf{1}]$$

where $k' = k[\text{MeI}]_0$

The integrated rate equation for this reaction (under pseudo first-order conditions) is:

$$\ln\frac{[\mathbf{1}]_t}{[\mathbf{1}]_0} = -k't$$

where *t* is the time since the start of the reaction (s).

A plot of $\ln([\mathbf{1}]_t/[\mathbf{1}]_0)$ vs *t* (using the values shown in Table S9) is linear, as shown below. The slope of the line is $-k'$.

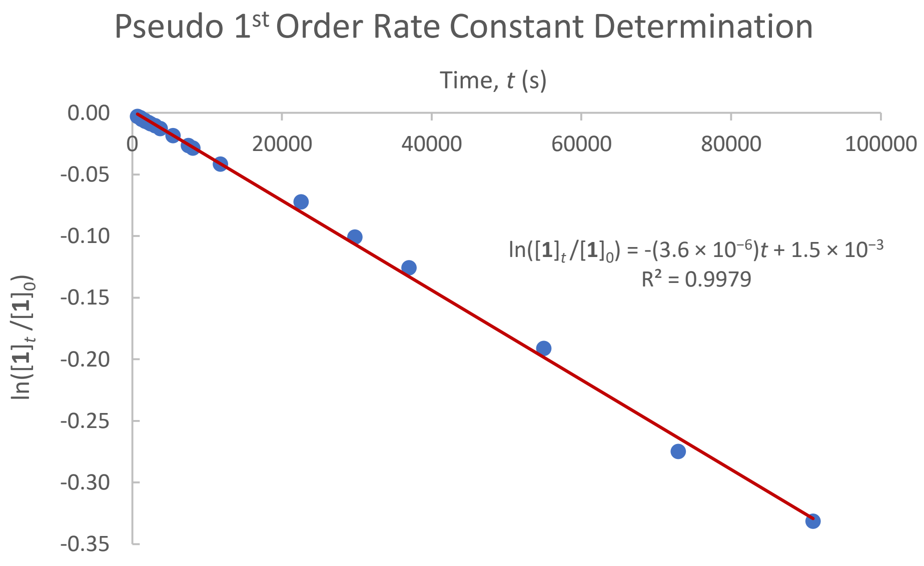

The slope of the plot is -3.6×10^{-6}, so $k' = -3.6 \times 10^{-6}\ \text{s}^{-1}$. Hence, since $[\text{MeI}]_0 = 1.25\ \text{mol L}^{-1}$,

$$k = (3.6 \times 10^{-6})\ \text{s}^{-1} \times (1.25\ \text{mol L}^{-1})$$

$$= 2.9 \times 10^{-6}\ \text{mol L}^{-1}\text{s}^{-1}$$

An identical value of the second order rate constant is also determined by monitoring the growth in the concentration of product **13a**.

This value of k may be related to $\Delta G^{\ddagger}$ by the Eyring equation:

$$k = \boldsymbol{\kappa}\frac{k_B T}{h} \times \frac{RT}{p^{\circ}}\ e^{-\frac{\Delta G^{\ddagger}}{RT}}$$

as seen in Atkins' Physical Chemistry, 9th ed. Section 22.4 pg. 848.[17] The transmission coefficient κ is taken to equal 1.

This equation can be rearranged to:

$$RT\ \ln\left(\left(\frac{1}{k}\right)\left(\frac{k_B T}{h}\right)\left(\frac{RT}{p^{\circ}}\right)\right) = \ \Delta G^{\ddagger}$$

giving:

$$\Delta G^{\ddagger} = 1.4 \times 10^{2}\ \text{kJ mol}^{-1}$$

where:

$R = 3.14\ \text{J K}^{-1}\text{mol}^{-1}$ $\quad T = 298\ \text{K}$

$p^{\circ} = 10^{5}\ \text{N m}^{-2}$ $\quad k_B = 1.38\ \times 10^{-23}\ \text{J K}^{-1}$ $\quad h = 6.63 \times 10^{-34}\ \text{J s}$

14 Additional Literature References from Main Article

S-1. Alkylation of amides (see also reference 19 of main article):

(a) Bredereck, H.; Gompper, R.; Rempfer, H.; Klemm, K.; Keck, H. *Chem. Ber*. **1959**, *92*, 329–337;

(b) Bredereck, H; Effenberger, F.; Simchen, G. *Chem. Ber*. **1963**, *96*, 1350 – 1355;

(c) Bredereck, H.; Gompper, R.; Theilig, G. *Chem. Ber*. **1954**, *87,* 537 – 546;

(d) Challis, B. C.; Challis, J. in *The Chemistry of Amides*; Zabicky, J., Ed.; Interscience: London, UK, **1970**; p. 731–858;

(e) Allouch, F.; Dwadnia, N.; Vologdin, N. V.; Svyaschenko,H. Cattey, Y. V.; Penouilh, M.-J.; Roger, J.; Naoufal, D.; Ben Salem, R.; Pirio, N.; Hierso, J.-C. *Organometallics* **2015**, *34*, 5015–5028;

(f) Smith, M. B.; Shroff, H. N. *J. Org. Chem*. **1984**, *49*, 2900–2906;

(g) Deslongchemps, P.; Caron, M. *Can. J. Chem*. **1980**, *58*, 2061 – 2068;

(h) Stirling, C. J. M. *J. Chem. Soc*. **1960**, 255 – 262;

(i) Ates, A.; Curran, D. P. *J. Am. Chem. Soc.* **2001**, *123*, 5130 – 5131.

S-2. Alkylation of amide anions (see also reference 21 of main article):

(a) See ref. S-1h above;

(b) See ref. S-1i above;

(c) Stein, A. R.; Tan, S. H. *Can. J. Chem.* **1974**, *52*, 4050 – 4061;

(d) Ragnarsson, U.; Grehn, L. *Acc. Chem. Res.* **1991**, *24*, 285 – 289.

S-3. Alkylation of anions of pyridone or quinolone (see also reference 22 of main article):

(a) Chung, N. M.; Tieckelmann, H. *J. Org. Chem*. **1970**, *35*, 2517–2520;

(b) Räth, C. *Liebigs Ann. Chem.* **1931**, *489*, 107–118;

(c) Effenberger, F.; Brodt, W.; Zinczuk, J. *Chem. Ber*. **1983**, *116*, 3011 – 3026;

(d) Nishiwaki, N.; Hisaki, M.; Ono, M.; Ariga, M. *Tetrahedron* **2009**, *65*, 7403-7407.

S-4. Alkylation of imide anions (see also reference 23 of main article):

(a) Gibson, M. S.; Bradshaw, R.W. *Angew. Chem. Int. Ed*. **1968**, *7*, 919 – 930;

(b) See ref. S-2d above.

S-5. Examples of reactions of anionic ambident nucleophiles containing N- and O-centred nucleophilic sites in which coordination to a counter-cation influences site-selectivity, i.e. the selectivities are dependent on the identity and nature of the cation employed (see also reference 24 of main article):

(a) See ref. S-2c above.

(b) See ref. S-3b above.

15 Supporting Information References

1. Williams, D. B. G.; Lawton, M. *J. Org. Chem.* **2010**, *75*, 8351–8354.
2. Shriver, D. F.; Drezdzon, M.A *The Manipulation of Air-Sensitive Compounds, 2nd Edition*; John Wiley & Sons: New York, 1986.
3. Kokatla, H. P.; Thomson, P. F.; Bae, S.; Doddi, V. R.; Lakshman, M. K. *J. Org. Chem.* **2011**, *76*, 7842–7848.
4. Procedure for synthesis of aromatic *N*-oxides: Leclerc, J. P.; Fagnou, K. *Angew. Chem. Int. Ed*. **2006**, *45*, 7781–7786
5. Głaszczka, R.; Jaźwiński, J.. *J. Mol. Struct.* **2014**, *1061*, 150–159.
6. Freire Franco, M. S.; de Paula, M. H.; Glowacka, P. C.; Fumagalli, F.; Clososki, G. C.; da Silva Emery, F. *Tetrahedron Lett.* **2018**, *59*, 2562–2566.
7. Larionov, O. V.; Stephens, D.; Mfuh, A. M.; Arman, H. D.; Naumova, A. S.; Chavez, G.; Skenderi, B. *Org. Biomol. Chem.* **2014**, *12*, 3026–3036.
8. Denegri, B.; Streiter, A.; Jurić, S.; Ofial, A. R.; Kronja, O.; Mayr, H. *Chem. Eur. J.* **2006**, *12*, 1648–1656
9. Jovanovic, M. V. **1985**, 23, 2299–2315
10. Forrester, J.; Jones, R. V. H.; Preston, P. N.; Simpson, E. S. C. *Perkin Trans.* **1995**, 2289–2291.
11. Ma, X.; Dang, H.; Rose, J. A.; Rablen, P.; Herzon, S. B. *J. Am. Chem. Soc.* **2017**, *139*, 5998–6007.
12. King, J. A.; Bryant, G. L. *Synthetic Commun.* **1994**, *24*, 1923–1935.
13. Breneman, C. M.; Wiberg, K. B. *J. Comput. Chem.* **1990**, *11*, 361–373.
14. (a) Singh, U. C.; Kollman, P. A. *J. Comput. Chem.* **1984**, *5*, 129–145; (b) Besler, B. H.; Merz Jr., K. M.; Kollman, P. A. *J. Comput. Chem.* **1990**, *11*, 431–439.
15. Glendening, E. D.; Badenhoop, J. K.; Reed, A. E.; Carpenter, J. E.; Bohmann, J. A.; Morales, C. M.; Landis, C. R.; Weinhold, F. *NBO 6.0*; Theoretical Chemistry Institute, University of Wisconsin, Madison, WI, 2013.
16. Bader, R. F. W. *Atoms in Molecules, A Quantum Theory*; Clarendon Press: Oxford, 1994.
17. Atkins, P. W.; De Paula, J. *Physical Chemistry, 9th Edition;* Oxford University Press: Oxford, 2010
