# Supplementary material for: Competition between N and O: use of diazine N-oxides as a test case for the Marcus theory rationale for ambident reactivity
Source: Chem Sci. 2020 Jul 23;11(35):9630–47. doi: 10.1039/d0sc02834g (PMC8162281; doi:10.1039/d0sc02834g)
Supplement: SC-011-D0SC02834G-s023 [file SC-011-D0SC02834G-s023.docx]

Compound **14** - from Pyrazine *N*-oxide + benzhydryl chloride + AgOTf in CH_2_Cl_2_. See Supporting Information, pg. S17.

NMR Spectrometer: Bruker Avance III 600

Acquisition Software: Bruker Topspin version 3.5.7

Program used to process software: MestreNova

Reference Frequency for ^1^H NMR: 600 MHz

Reference Frequency for ^15^N NMR: 60.8 MHz

Unprocessed NMR spectra from the above reaction are provided in JCAMP-DX format. Since saving in this format does not allow preservation of correct integration curves (1D NMR spectra) or insertion of spectral traces (2D spectra), the spectra are provided in their original, unprocessed state, with the exception of application of t1 noise reduction to ^1^H-^15^N HMBC NMR spectra.

**Note**: Both of the ^1^H and ^1^H-^15^N HMBC NMR spectra produced upon opening the raw NMR files are mis-referenced in the ^1^H dimension (f2) – it is necessary to re-reference the CH_2_Cl_2_ signal to 5.32 ppm.
