# Supplementary material for: Competition between N and O: use of diazine N-oxides as a test case for the Marcus theory rationale for ambident reactivity
Source: Chem Sci. 2020 Jul 23;11(35):9630–47. doi: 10.1039/d0sc02834g (PMC8162281; doi:10.1039/d0sc02834g)
Supplement: SC-011-D0SC02834G-s025 [file SC-011-D0SC02834G-s025.docx]

Compounds **23b** and **21b** - from Pyrimidine *N*-oxide + MeOTf in CD_3_CN. NMR spectra recorded in CD_3_CN, then solvent swapped to DMSO-*d*_6_. Supporting Information, pg. S31 – S33.

**NMR Spectra in CD_3_CN**

NMR Spectrometer: Bruker Avance I 400

Acquisition Software: Bruker Topspin version 2.1

Program used to process software: MestreNova (^1^H, HSQC, HMBC, COSY)

Topspin (^13^C{^1^H})

Reference Frequency for ^1^H NMR: 400 MHz

Reference Frequency for ^13^C NMR: 100 MHz

**NMR Spectra in DMSO-*d*_6_**

NMR Spectrometer: Bruker Avance III 600

Acquisition Software: Bruker Topspin version 3.5.7

Program used to process software: MestreNova

Reference Frequency for ^1^H NMR: 600 MHz

Reference Frequency for ^15^N NMR: 60.8 MHz

**Note**: The ^1^H-^15^N HMBC NMR spectrum produced from the raw data is mis-referenced in the f2 dimension (^1^H dimension) and needs to be re-referenced upon opening.

Unprocessed NMR spectra from the above reaction are provided in JCAMP-DX format. Since saving in this format does not allow preservation of correct integration curves (1D NMR spectra) or insertion of spectral traces (2D spectra), the spectra are provided in their original, unprocessed state, with the exception of application of t1 noise reduction to ^1^H-^15^N HMBC NMR spectra.
