# Supplementary material for: Competition between N and O: use of diazine N-oxides as a test case for the Marcus theory rationale for ambident reactivity
Source: Chem Sci. 2020 Jul 23;11(35):9630–47. doi: 10.1039/d0sc02834g (PMC8162281; doi:10.1039/d0sc02834g)
Supplement: SC-011-D0SC02834G-s039 [file SC-011-D0SC02834G-s039.docx]

Compound **23b** - from Pyrimidine *N*-oxide + MeOTf in MeCN, NMR solvent = CD_3_CN. Supporting Information, pg. S28 – S29.

NMR Spectrometer: Bruker Avance III 300

Acquisition Software: Bruker Topspin version 3.2

Program used to process software: MestreNova

Reference Frequency for ^1^H NMR: 600 MHz

Unprocessed NMR spectra from the above reaction are provided in JCAMP-DX format. Since saving in this format does not allow preservation of correct integration curves, the spectra are provided in their original, unprocessed state.
