# Supplementary material for: Competition between N and O: use of diazine N-oxides as a test case for the Marcus theory rationale for ambident reactivity
Source: Chem Sci. 2020 Jul 23;11(35):9630–47. doi: 10.1039/d0sc02834g (PMC8162281; doi:10.1039/d0sc02834g)
Supplement: SC-011-D0SC02834G-s046 [file SC-011-D0SC02834G-s046.docx]

Compound **17a** - from quinoxaline *N*-oxide + MeI (no solvent) – product dissolved in DMSO-*d*_6_ (^1^H NMR) or DMSO (non-deuterated, ^1^H-^15^N HMBC NMR). See Supporting Information, pg. S18 – S19.

NMR Spectrometer: Bruker Avance III 600

Acquisition Software: Bruker Topspin version 3.5.7

Program used to process software: MestreNova

Reference Frequency for ^1^H NMR: 600 MHz

Reference Frequency for ^15^N NMR: 60.8 MHz

Unprocessed NMR spectra from the above reaction are provided in JCAMP-DX format. Since saving in this format does not allow preservation of correct integration curves (1D NMR spectra) or insertion of spectral traces (2D spectra), the spectra are provided in their original, unprocessed state, with the exception of application of t1 noise reduction to ^1^H-^15^N HMBC NMR spectra. The signal intensity in the ^1^H-^15^N HMBC NMR spectrum is low due to the low conversion in the reaction and the use of non-deuterated solvent for this spectroscopic experiment.
